# Supplementary material for: Factors associated with pneumococcal nasopharyngeal carriage: A systematic review
Source: PLOS Glob Public Health. 2022 Apr 11;2(4):e0000327. doi: 10.1371/journal.pgph.0000327 (PMC10021834; doi:10.1371/journal.pgph.0000327)
Supplement: S1 Text — (DOCX) [file pgph.0000327.s002.docx]

# **S1 Text. Search strategies**

## Medline (Ovid) search strategy

1. Streptococcus pneumoniae/
2. exp Pneumococcal Infections/
3. Carrier State/
4. Nasopharynx/
5. (1 or 2) and 3 and 4
6. ((pneumococcal adj carriage) or (nasopharyngeal adj carriage)).tw,kf,hw.
7. (1 or 2) and 6
8. 5 or 7 9. exp animals/ not human*.sh.
9. 8 not 9

## Embase (Ovid) search strategy

1. Streptococcus pneumoniae/
2. pneumococcal infection/
3. bacterium carrier/
4. bacterial colonization/
5. prevalence/
6. risk factor/
7. nasopharynx/
8. ((pneumococcal adj carriage) or (nasopharyngeal adj carriage)).tw,kw,hw.
9. exp ANIMAL/ not human*.sh.
10. (1 or 2) and (3 or 4) and (5 or 6 or 7)
11. (1 or 2) and 8
12. (10 or 11) not 9

## Cochrane library search strategy

ID Search

#1 MeSH descriptor: [Streptococcus pneumoniae] this term only

#2 "Streptococcus pneumoniae" or pneumococcal or pneumococci (Word variations have been searched)

#3 MeSH descriptor: [Pneumococcal Infections] explode all trees

#4 MeSH descriptor: [Carrier State] this term only

#5 Carrier or carriage or colonization or colonisation (Word variations have been searched)

#6 MeSH descriptor: [Nasopharynx] this term only

#7 nasopharynx or nasopharyngeal (Word variations have been searched)

#8 (#1 or #2 or #3) and (#4 or #5) and (#6 or #7)

#9 "pneumococcal carriage" or "nasopharyngeal carriage" (Word variations have been searched)

#10 (#1 or #2 or #3) and #9

#11 #8 or #10

#12 MeSH descriptor: [Humans] explode all trees

#13 MeSH descriptor: [Animals] explode all trees

#14 human or humans (Word variations have been searched)

#15 animal or animals or mouse or mice (Word variations have been searched)

#16 (#13 or #15) not (#12 or #14)

#17 #11 not #16

## PubMed search strategy

(("Streptococcus pneumoniae"[All Fields] OR ("pneumococcal vaccines"[MeSH Terms] OR ("pneumococcal"[All Fields] AND "vaccines"[All Fields]) OR "pneumococcal vaccines"[All Fields] OR "pneumococcal"[All Fields]) OR pneumococci[All Fields]) AND (("carrier state" [MeSH Terms] OR ("carrier"[All Fields] AND "state"[All Fields]) OR "carrier state"[All Fields] OR "carrier"[All Fields]) OR carriage[All Fields] OR colonization[All Fields] OR colonisation[All Fields]) AND (("nasopharynx"[MeSH Terms] OR "nasopharynx"[All Fields]) OR ("nasopharynx"[MeSH Terms] OR "nasopharynx"[All Fields] OR "nasopharyngeal"[All Fields])) AND (NOTNLM[All Fields] OR publisher[sb] OR inprocess[sb] OR pubmednotmedline[sb] OR indatareview[sb] OR pubstatusaheadofprint[All Fields])) NOT (("Streptococcus pneumoniae"[All Fields] OR ("pneumococcal vaccines"[MeSH Terms] OR ("pneumococcal"[All Fields] AND "vaccines"[All Fields]) OR "pneumococcal vaccines"[All Fields] OR "pneumococcal"[All Fields]) OR pneumococci[All Fields]) AND (("carrier state" [MeSH Terms] OR ("carrier"[All Fields] AND "state"[All Fields]) OR "carrier state"[All Fields] OR "carrier"[All Fields]) OR carriage[All Fields] OR colonization[All Fields] OR colonisation[All Fields]) AND (("nasopharynx"[MeSH Terms] OR "nasopharynx"[All Fields]) OR ("nasopharynx"[MeSH Terms] OR "nasopharynx"[All Fields] OR "nasopharyngeal"[All Fields])) AND (NOTNLM[All Fields] OR publisher[sb] OR inprocess[sb] OR pubmednotmedline[sb] OR indatareview[sb] OR pubstatusaheadofprint[All Fields]) AND "animals" [MeSH Terms:noexp])
