# Supplementary material for: Factors associated with pneumococcal nasopharyngeal carriage: A systematic review
Source: PLOS Glob Public Health. 2022 Apr 11;2(4):e0000327. doi: 10.1371/journal.pgph.0000327 (PMC10021834; doi:10.1371/journal.pgph.0000327)
Supplement: S1 Table — (DOCX) [file pgph.0000327.s003.docx]

**S1 Table. Summary of studies reporting risk factors for pneumococcal carriage, stratified by World Bank income status, WHO region, and country**

| **WHO region^a^** | **Country** | **Ref** | **Study timeframe** | **Study design** | **Setting** | **Sample size** | **Age groups included in the study** | **Risk factor analysis method** | **Variable selection^b^** |
| --- | --- | --- | --- | --- | --- | --- | --- | --- | --- |
| **Low-income countries^c^ (n = 17)** | | | | | | | | | |
| Africa | Ethiopia | [1] | Feb-May 2012 | Cross-sectional | Rural, urban community | 234 | ≤ 10 years | Multivariable logistic regression | Empirical, stepwise selection (P < 0.05) |
|  |  | [2] | Jun-Sep 2014 | Cross-sectional | Jimma town, children attending well-chid or sick care visits | 361 | < 5 years | Multivariable logistic regression | Empirical (P < 0.25) |
|  |  | [3] | Sep–Dec 2016 | Cross-sectional | Community (within Sodo Zuria Woreda of Wolaita Zone, Ethiopia) | 710 | 3–13 years | Multivariable logistic regression | NR |
|  |  | [4] | Mar–May 2018 | Cross-sectional | Urban community | 317 | 3–6 years | Multivariable logistic regression | NR |
|  | Kenya | [5] | 2-24 Mar 2004,  2 Jun-Jul 2004 | Cross-sectional | Community (within the Kilifi Health and Demographic Surveillance System) | 864 | All ages | Random effects logistic regression to adjust for within-subject correlation | a priori (age); empirical via likelihood ratio tests, P < 0.05 |
|  |  | [6] | Oct 2006-Dec 2008 | Cross-sectional | Semi-urban communities (within the Kilifi Health and Demographic Surveillance System) | 2840 | 3-59 months | Multivariable logistic regression | Empirical (P > 0.5), backward stepwise exclusion |
|  | Niger | [7] | Jun 2007-May 2008 | Cross-sectional | Community, health center for routine immunization | 1200 | 0-2 years | Multivariable logistic regression | NR |
|  | The Gambia | [8] | Nov 2006-Jun 2008 | Nested longitudinal | Rural villages in western Gambia | 636 | > 30 months | Multivariable logistic regression | a priori (age, trial arm) |
|  |  | [9] | May-Aug 2009 | Cross-sectional | Rural communities | 847 | All ages | Multivariable logistic regression with robust standard errors to adjust for village-level clustering | a priori (age and sex) |
|  |  | [10] | Apr 2013-Apr 2014 | Retrospective nested cohort | Peri-urban health facility | 374 | 0-28 days | Multivariable logistic regression | a priori (birth weight, other children in the household, mother’s age, mother’s education, and season) |
|  |  | [11] | NR | Cross-sectional | Rural villages, community | 2972 | All ages | Multivariable logistic regression with robust standard errors to adjust for village-level clustering | a priori (sex, age) |
|  | Uganda | [12] | Jan-Mar 2014 | Cross-sectional | Four rural sub-counties | 566 | All ages | Age-adjusted log-binomial model with robust variance estimator | Empirical (P < 0.1) |
|  |  | [13] | 2008, 2009, 2011 | Cross-sectional | Mainly rural, some peri-urban | 1761 | < 5 years | Multivariable logistic regression | Empirical (P < 0.2) |
|  |  | [14] | Jan-Mar 2014 | Cross-sectional | Four rural sub-counties | 1346 | All ages | Multivariable logistic regression | Age-adjusted sex, antibiotic use, symptoms of respiratory tract infection |
| South-East Asia | India | [15] | Oct 1998-Jun 1999 | Nested longitudinal | Rural south Indian | 464 | 2-2.5, 4, and 6 months | Multivariable logistic regression | Empirical (P < 0.1) |
|  | Nepal | [16] | Oct 2001-Jan 2006 | Nested case-control | Rural district in Southern Nepal | 550 healthy controls | 1-35 months | Multivariable logistic regression | Empirical (P ⩽ 0.10) |
| Western Pacific | Vietnam | [17] | Apr 2008-Mar 2009 | Cross-sectional | Urban, community | 883 | < 5 years | Multivariable logistic regression | NR |
| **Lower-middle-income countries^c^ (n = 11)** | | | | | | | | | |
| Africa | Angola | [18] | Nov–Dec 2017 | Cross-sectional | Community (city of Luanda, villages surrounding Saurimo) | 940 | 4–12 years | Multivariable logistic regression | NR |
|  | Nigeria | [19] | NR | Cross-sectional | Community, peri-urban | 1025 | All ages | Logistic regression with random effects to adjust for location-level clustering | a priori (sex and age); adjusted odds ratios for the presence of carriage obtained separately for each risk factor |
| Americas | Bolivia | [20] | May–Jun 2007 | Cross-sectional | Urban, community | 601 | Children (ages not specified) mean 9.5 years (SD 3.5) | Mixed-effects multivariable logistic regression models to account for clustering of samples within schools | Backward deletion based on likelihood ratio test |
|  | Brazil | [21] | Nov 2002-Jul 2003 | Cross-sectional | Public schools among 11 Sanitary Districts of Brazil | 1013 | 10-19 years | Multivariable logistic regression | Empirical (95% CI not crossing null value) and stepwise adjustment |
| Eastern Mediterranean | West Bank and Gaza | [22] | Mar-Jul 2009 | Cross-sectional | Community based | 379 | < 5.5 years | Multivariable logistic regression | Empirical (P < 0.2) |
| South East Asia | Indonesia | [23] | Feb-Apr 2010 | Cross-sectional | Urban community | 243 | 6-60 months | Multivariable logistic regression | Empirical (backward stepwise with P < 0.2) |
|  |  |  |  |  |  | 253 | 45-70 years |  |  |
|  |  | [24] | Nov 2014–Jan 2015 | Longitudinal | Urban and semi-rural community | 200 | 2–12 months | Multivariable logistic regression, incorporating generalized estimating equations with robust 95% Cis and an unstructured working correlation matrix to account for repeated sampling of individuals | a priori (age, antibiotic exposure, and presence of upper respiratory tract infection symptoms) |
| Western Pacific | China | [25] | Apr-May, & Oct-Nov 2009 | Cross-sectional | Urban, Shanghai immunization clinics | 614 | 12-18 months | Multivariable logistic regression | a priori (age, season, “urbanicity”); empirical (P < 0.05) |
|  | Fiji | [26] | Oct 2003-Apr 2004 | Cross-sectional | Rural, urban community | 774 | 3-13 months | Multivariable logistic regression | Empirical (P < 0.25) |
|  | Mongolia | [27] | May–Jul 2015  May–Jul 2017 | Cross-sectional | Urban, community | 961 | 5–8 weeks | Multivariable logistic regression | Empirical (P < 0.2; then stepwise with P < 0.05) |
|  |  |  |  |  |  | 989 | 12–23 months |  |  |
|  | Lao People’s Democratic Republic | [28] | Nov 2013–Feb 2014  Nov 2015–Feb 2016 | Cross-sectional | Rural, urban community | 999 | 5–8 weeks | Multivariable logistic regression | a priori (symptoms of upper respiratory tract infection, > 2 children < 5 years in the household, poverty) and empirical (P < 0.2) |
|  |  |  |  |  |  | 1010 | 12–23 months |  |  |
| **Upper-middle-income countries^c^ (n = 18)** | | | | | | | | | |
| Africa | South Africa | [29] | Jan 2007-May 2009 | Cohort | Urban, community | 251 | 6-12 weeks to 2 years and mothers of infants aged 6–12 weeks to 2 years | GEE multivariable logistic regression | NR |
|  |  | [30] | Mar 2012–Jul 2015 | Nested cohort | Peri-urban community | 986 | Birth–12 months | Multivariable Poisson regression | a priori (weight-for-age-z-score at birth, prematurity, ethnicity, sex, HIV exposure, time on exclusive breastfeeding, the average number of people sleeping per sleeping room, dwelling category, recent respiratory infection, childcare attendance, vaccination, number of other children < 5 years living in the household, and antibiotic use) and exploration of potential confounding by bacterial co-colonization by using indicator variables for each pathogen |
|  |  |  |  |  |  | 982 | Mothers of infants |  |  |
|  |  | [31] | Jun-Dec 2014  June–Dec 2016 | Cross-sectional | Community, routine immunizations | 202 | ~6–14 weeks | Multivariable logistic regression | Empirical (details not provided) |
|  |  |  |  |  |  | 99 | 9 months |  |  |
|  |  |  |  |  |  | 93 | 18 months |  |  |
|  |  |  |  |  |  | 60 | 5 years |  |  |
| Americas | Brazil | [32] | Jul–May 2001 | Cross-sectional | Favela (slum) community | 262 | All ages | GEE multivariable logistic regression to adjust for household-level clustering | a priori (age, sex) and empirical (P < 0.05) |
|  |  | [33] | Jan 2008–Jan 2009 | Cohort | Peri-urban | 203 | 1–48 months | Multivariable logistic regression with bootstrapping | Empirical (P < 0.05 and 95% CI did not include 1) |
|  |  | [34] | Sep–Dec 2014 | Cross-sectional | Community, routine check-up / sick visits | 522 | < 6 years | Multivariable logistic regression | NR |
|  | Cuba | [35] | Oct–Dec 2013 | Cross-sectional | Rural, urban, community | 1050 | 2–18 months | Multivariable logistic regression | Empirical (P < 0.05) |
|  | Venezuela | [36] | Dec 2006–Jan 2008 | Cross-sectional | Rural, urban community | 1004 | 3–65 months | Multivariable logistic regression | Empirical (P < 0.10) |
|  |  | [37] | NR | Cross-sectional | Isolated rural communities | 504 | 0–4 years | GEE multivariable logistic regression to adjust for community-level clustering | NR |
|  |  |  |  |  |  | 227 | 5–10 years |  |  |
|  |  |  |  |  |  | 333 | 13–62 years |  |  |
| Eastern Mediterranean | Iran | [38] | Feb–Apr 2016 | Cross-sectional | Urban, community | 532 | 7–14 years | Multivariable logistic regression | NR |
| Europe | Poland | [39] | Nov–Dec 2002 (Autumn).  Feb–Mar 2003 (Winter).  May–Jun 2003 (Spring) | Cohort | Community | 311 each season | 3–5 years | Multivariable logistic regression | a priori (age, recent RTI, antibiotic use); Empirical (P < 0.1) |
|  | Turkey | [40] | Nov 2003–Feb 2004 | Cross-sectional | Rural, urban, community; well-child clinics | 564 | 0–2 years | Multivariable logistic regression | Empirical (P < 0.25) |
|  |  | [41] | Feb–Mar 2004 | Cross-sectional | Urban | 301 | 9 days–67 months | Multivariable logistic regression | NR |
|  |  | [42] | Apr–Jun 2011 | Cross-sectional | Community, via well-child outpatient clinical/general pediatric outpatient clinic | 1101 | 1 months–18 years | Multivariable logistic regression | Empirical (P < 0.1) |
|  |  | [43] | Sep–Dec 2014 | Cross-sectional | Urban, community | 150 | 0–6 years | Multivariable logistic regression | NR |
| Western Pacific | Fiji | [44] | Sep-Dec 2012  Jul–Nov 2013  Jul–Dec 2014  Aug–Nov 2015 | Cross-sectional | Rural, urban community | 2006 | 5–8 weeks | Multivariable logistic regression | a priori (survey year, residential location, low family income, > 2 children < 5 years living in the household) selected via directed acyclic graph |
|  |  | [45] | Aug–Nov 2015 | Cross-sectional | Rural, urban community | 496 | 5–8 weeks | Multivariable logistic GEE regression | a priori (ethnicity and residential location) and empirical (P < 0.05) |
|  |  |  |  |  |  | 498 | 12–23 months |  |  |
|  |  |  |  |  |  | 510 | 2–6 years |  |  |
|  |  |  |  |  |  | 510 | Caregivers of infant and child participants |  |  |
|  |  | [46] | Sep-Dec 2012  Jul–Nov 2013  Jul–Dec 2014  Aug–Nov 2015 | Cross-sectional | Rural, urban community | 2006 | 5–8 weeks | Multivariable logistic regression | a priori (PCV10 vaccination, survey year, ethnicity, participant group, and symptoms of upper respiratory tract infection) and empirical (P < 0.2) |
|  |  |  |  |  |  | 2004 | 12–23 months |  |  |
|  |  |  |  |  |  | 2052 | 2–6 years |  |  |
|  |  |  |  |  |  | 2047 | Caregivers of infant and child participants |  |  |
| **High-income countries^c^ (n = 36)** | | | | | | | | | |
| Americas | Canada | [47] | 2003-2006  2010-2012 | Cross-sectional | Community | 6149 | 10 months-5 years | Multivariable logistic regression | a priori, then backward elimination |
|  | United States of America | [48] | Nov-Dec 1997 | Cross-sectional | Isolated rural communities | 737 | < 8 years | Multivariable logistic regression | Empirical (Likelihood-ratio test P < 0.05) or change in effect estimate of >15% for independent risk factors; and a priori |
|  |  | [49] | Apr 1997-Oct 2000 | Cohort, nested within a randomized controlled trial | Community, Navajo, and Apache reservations | 410 | < 6 years | GEE multivariable logistic regression to adjust for within-subject correlation | Empirical (P ≤ 0.10) |
|  |  | [50] | Jan 1998-Jan 1999 | Cross-sectional | Urban, healthy attendees at routine well-child checks at a university-based pediatric outpatient clinic | 291 | < 5 years | Multivariable logistic regression | Empirical (P ≤ 0.10) |
|  |  | [51] | Mar-May 2001 | Cross-sectional | Rural and urban Massachusetts | 766 | < 7 years | GEE multivariable logistic regression to adjust for community-level clustering | Empirical (P < 0.10), then stepwise removal of non-significant variables |
|  |  | [52] | Mar-May 2001 | Cross-sectional | Rural, urban Massachusetts communities | 710 | < 7 years | GEE multivariable logistic regression to adjust for census-tract- level clustering | a priori (recent antibiotic use), empirical (P < 0.05) |
|  |  | [53] | Mar-May 2001 | Cross-sectional | Rural, urban Massachusetts communities during well or sick child visits | 678 | 3 months-7 years | Generalized linear mixed models to adjust for community-level clustering | NR |
|  |  |  | Nov-Apr 2004 |  |  | 988 |  |  |  |
|  |  |  | Oct 2006-Apr 2007 |  |  | 972 |  |  |  |
|  |  | [54] | Feb-Mar 2000  Feb-Mar 2001  Feb-Mar 2002 | Cross-sectional | Alaskan community clinic for well-child or acute care | 1275 | 3-59 months | Multivariable logistic regression | a priori (age, clinic, up to date vaccination status), empirical |
|  |  | [55] | Feb-Mar 2000  Feb–Mar 2001  Feb-Mar 2002  Feb-Mar 2003  Feb-Mar 2004 | Cross-sectional | Urban Anchorage, Alaska | 2061 | 3-59 months | Multivariable logistic regression | Empirical (P < 0.25) |
|  |  | [56] | 2001  2004  2007  2009  2011 | Cross-sectional | Rural, urban Massachusetts communities | 5380 overall | 0-7 years | Multivariable logistic regression | a priori (sex, ethnicity, number of siblings ≤ 6 years, childcare attendance), |
|  |  | [57] | 2006-2007 | Cross-sectional (secondary analysis) | Rural, urban Massachusetts communities | 543 from urban Boston | < 7 years | Multivariable logistic regression, accounting for clustering by census tract | NR |
|  |  |  |  |  |  | 794 from outside Boston |  |  |  |
|  |  | [58] | Mar-Apr 2008  Mar-Apr 2009  Mar-Apr 2010  Mar-Apr 2011 | Cross-sectional | Rural Alaskan villages | 3417 | < 10 years | Multivariable logistic regression | Empirical (non-automatic backward selection) |
|  |  |  |  |  |  | 2663 | 10-17 years |  |  |
|  |  |  |  |  |  | 6455 | ≥ 18 years |  |  |
|  |  | [59] | 2006-2007  2008-2009 | Cross-sectional | Urban community | 1982 | 3 months-< 7 years | Multivariable logistic regression | NR |
| Eastern Mediterranean | Cyprus | [60] | Nov 2007-May 2008 | Cross-sectional | Urban, community | 402 | 6 months-5 years | Multivariable logistic regression | a priori (all variables) |
|  | Kingdom of Saudi Arabia | [61] | Hajj season 2011 and 2012 | Cross-sectional | Mass gathering of pilgrims | 3203 | > 18 years | Multivariable logistic regression | Empirical (95% CI did not cross null value) |
| Europe | France | [62] | Nov 2006-June 2009 | Cross-sectional | Rural, urban, distributed throughout France | 3507 | 6-24 months | Multivariable logistic regression | Empirical (P < 0.10) |
|  | France and the Kingdom of Saudi Arabia | [63] | Hajj season 2018 | Cohort | Gathering of pilgrims from Marseille, France | 121 | 26–83 years | Multivariable logistic mixed models, with random effects to account for repeated measures for pathogen carriage for each participant | Empirical (P < 0.2) |
|  | Greenland | [64] | Oct-Dec 2011 | Cross-sectional | Rural, urban, community | 352 | 0-6 years | Multivariable logistic regression | Empirical (P < 0.05) |
|  | Italy | [65] | Oct-Dec 2010 | Cross-sectional | Urban, community | 669 | 0–59 months | Binary regression model, with log link | NR |
|  |  | [66] | Nov 2011-Apr 2012 | Cross-sectional | Urban | 571 | < 5 years | Random effects logistic regression, with recruitment center set as a random effect | NR |
|  |  | [67] | 2012 | Cross-sectional | Urban in north Italy | 301 | < 6 years | General nonlinear structural equation modeling | Empirical (Log-likelihood ratio test P < 0.05) |
|  |  | [68] | Sep-Dec 2011 | Cross-sectional | Urban, community | 1250 | 3-59 months | Multivariable Poisson regression | a priori (all variables) |
|  | Portugal | [69] | Apr 2010-Dec 2012 | Cross-sectional | Urban, rural, community | 3361 | > 60 years | Multivariable logistic regression | Empirical (P <0.05) |
|  | Spain | [70] | Jun–Aug 2014  Jan–Mar 2015 | Cross-sectional | Community, southeast Spain, children attending Health Child Program | 906 | 10–14 months | Multivariable logistic regression | NR |
|  |  |  |  |  |  | 915 | 3.5–4.5 years |  |  |
|  | The Netherlands | [71] | Jun-Jul 2002  Sep-Nov 2002 | Cross-sectional | National population-based | 3198 | 1-19 years | Multivariable logistic regression | Empirical (P < 0.10), backward stepwise |
|  |  | [72] | Jun 2003-Nov 2006 | Nested cohort | Rural, urban, population-based | 1079 | 1.5 months; 6 months; 14 months | GEE multivariable logistic regression to adjust for within-subject correlation | a priori (birth weight, parity, gestation age, sex, siblings, maternal education level, childcare attendance, smoking duration of breastfeeding) |
|  |  | [73] | Jul 2005–Feb 2008 | Randomised controlled trial | Population-based | 326 | 6-24 months | GEE multivariable logistic regression to control for repeat measurements | a priori (sex, age, childcare attendance, symptoms of URTI, presence of siblings in household, antibiotic consumption < 2 months before swab, use of pacifier, season of sampling); empirical (P < 0.10) |
| Europe and Eastern Mediterranean | Israel /  West Bank and Gaza^d^ | [74] | 2009  2010  2011 | Cross-sectional | Population-based in East Jerusalem under Israeli Health law, and under Palestinian Authority; | 2570 | < 5 years | Multivariable logistic regression | Empirical (P < 0.2) |
| Western Pacific | Australia | [75] | Aug-Nov 2002.  Mar-May 2004 | Cross-sectional | Remote rural, Indigenous Australian communities, | 214 | ⩾ 2 years-< 16 years | Multivariable logistic regression | Empirical (P ≤ 0.20) |
|  |  |  |  |  |  | 324 | > 16 years |  |  |
|  | Hong Kong, British Protectorate | [76] | Sep 1992-Feb 1993 | Longitudinal | Urban Hong Kong; refugee detention camp | 621 Chinese children | < 5 years | Multivariable logistic regression | NR |
|  |  |  |  |  |  | 300 Vietnamese refugees in detention |  |  |  |
|  | Hong Kong SAR, China | [77] | Jun 2013-June 2014 | Cross-sectional | Four major regions in Hong Kong (Hong Kong Island, Kowloon, New Territories East, and New Territories West) | 1541 | 2, 12, and 18 months | Multivariable logistic regression | Empirical (P < 0.05) with consideration of clinical relevance |
|  | Japan | [78] | Jan 2008-Dec 2011 | Cohort | Sado Island community | 349 | 0-36 months | Multivariable Cox proportional hazards regression | Empirical (P < 0.05) |
|  |  | [79] | Jul 2010-Mar 2012 | Cross-sectional | Rural, urban, community | 229 | 2 months-6 years | Multivariable logistic regression | Empirical |
|  | Japan | [80] | Jan–Dec 2015 | Cross-sectional | Okinawa main island community, infants and children presenting for vaccination | 769 | 2–24 months | Multivariable logistic regression | NR |
|  | Taiwan (China) | [81] | Jul 2005-Dec 2010 | Cross-sectional | North, central, and south Taiwan, children presenting for regular vaccination or acute visits | 7747 PCV7 vaccinated (⩾ 1 dose) | 2-5 years | Multivariable logistic regression | Empirical (P < 0.05) |
|  |  |  |  |  |  | 1958 PCV7 unvaccinated (0 doses) |  |  |  |
|  |  | [82] | Jul 2005-Jul 2007 | Cohort | Community, via childcare centers, well-child or outpatient clinics | 6060 | 2 months-5 years | Multivariable logistic regression | Empirical (P < 0.05) |

Abbreviations: GEE–generalized estimating equation; NR–not reported; PCV–pneumococcal conjugate vaccine; PCV7–seven-valent pneumococcal conjugate vaccine; PCV10–ten-valent pneumococcal conjugate vaccine; RTI–respiratory tract illness; URTI–upper respiratory tract infection; WHO–World Health Organization. Footnotes: ^a^ As per countries listed under WHO regional offices[83]; ^b^ Where reported, P-values refer to threshold associated with a hypothesis test for a single variable to be included in a multivariable model, if the P-value is smaller than that threshold, unless otherwise specified; ^c^ World Bank Income status at the time the study was undertaken[84]; ^d^ This study was conducted in high-income Israel (WHO European region) and lower-middle-income West Bank and Gaza (WHO Eastern Mediterranean regions)[74, 83, 84].

# References

1. Assefa A, Gelaw B, Shiferaw Y, Tigabu Z. Nasopharyngeal carriage and antimicrobial susceptibility pattern of *Streptococcus pneumoniae* among pediatric outpatients at Gondar University Hospital, North West Ethiopia. PEDN. 2013;54(5):315-21. doi: <https://dx.doi.org/10.1016/j.pedneo.2013.03.017>. PubMed PMID: 23680262.

2. Gebre T, Tadesse M, Aragaw D, Feye D, Beyene HB, Seyoum D, et al. Nasopharyngeal carriage and antimicrobial susceptibility patterns of *Streptococcus pneumoniae* among children under five in Southwest Ethiopia. Children. 2017;4(4). doi: 10.3390/children4040027. PubMed PMID: 28422083.

3. Wada FW, Tufa EG, Berheto TM, Solomon FB. Nasopharyngeal carriage of Streptococcus pneumoniae and antimicrobial susceptibility pattern among school children in South Ethiopia: post-vaccination era. BMC research notes. 2019;12(1):306. doi: <https://dx.doi.org/10.1186/s13104-019-4330-0>.

4. Haile AA, Gidebo DD, Ali MM. Colonization rate of Streptococcus pneumoniae, its associated factors and antimicrobial susceptibility pattern among children attending kindergarten school in Hawassa, southern Ethiopia. BMC Res Notes. 2019;12(1):344. Epub 2019/06/19. doi: 10.1186/s13104-019-4376-z. PubMed PMID: 31208447; PubMed Central PMCID: PMCPMC6580519.

5. Abdullahi O, Nyiro J, Lewa P, Slack M, Scott JA. The descriptive epidemiology of *Streptococcus pneumoniae* and *Haemophilus influenzae* nasopharyngeal carriage in children and adults in Kilifi district, Kenya. Ped Infect Dis J. 2008;27(1):59-64. doi: <https://dx.doi.org/10.1097/INF.0b013e31814da70c>. PubMed PMID: 18162940.

6. Abdullahi O, Karani A, Tigoi CC, Mugo D, Kungu S, Wanjiru E, et al. The prevalence and risk factors for pneumococcal colonization of the nasopharynx among children in Kilifi District, Kenya. PLoS One. 2012;7(2):e30787. doi: <https://dx.doi.org/10.1371/journal.pone.0030787>. PubMed PMID: 22363489.

7. Ousmane S, Diallo BA, Ouedraogo R, Sanda AA, Soussou AM, Collard JM. Serotype distribution and antimicrobial sensitivity profile of *Streptococcus pneumoniae c*arried in healthy toddlers before PCV13 introduction in Niamey, Niger. PLoS One. 2017;12(1):e0169547. doi: 10.1371/journal.pone.0169547. PubMed PMID: 28103262.

8. Bojang A, Jafali J, Egere U, Hill P, Antonio M, Jeffries D. Seasonality of pneumococcal nasopharyngeal carriage in rural Gambia determined within the context of a cluster randomized pneumococcal vaccine trial. PLoS One. 2015;10(7):13. PubMed PMID: CN-01130937.

9. Usuf E, Badji H, Bojang A, Jarju S, Ikumapayi UN, Antonio M, et al. Pneumococcal carriage in rural Gambia prior to the introduction of pneumococcal conjugate vaccine: a population-based survey. Trop Med Int Health. 2015;20(7):871-9. doi: <https://dx.doi.org/10.1111/tmi.12505>. PubMed PMID: 25778937.

10. Usuf E, Bojang A, Camara B, Jagne I, Oluwalana C, Bottomley C, et al. Maternal pneumococcal nasopharyngeal carriage and risk factors for neonatal carriage after the introduction of pneumococcal conjugate vaccines in The Gambia. Clin Microbiol Infect. 2018;24(4):389-95. Epub 2017/07/27. doi: 10.1016/j.cmi.2017.07.018. PubMed PMID: 28743545.

11. Hill PC, Akisanya A, Sankareh K, Cheung YB, Saaka M, Lahai G, et al. Nasopharyngeal carriage of *Streptococcus pneumoniae* in Gambian villagers. Clin Infect Dis 2006;43(6):673-9. doi: <https://dx.doi.org/10.1086/506941>. PubMed PMID: 16912937.

12. le Polain de Waroux O, Flasche S, Kucharski AJ, Langendorf C, Ndazima D, Mwanga-Amumpaire J, et al. Identifying human encounters that shape the transmission of *Streptococcus pneumoniae* and other acute respiratory infections. Epidemics. 2018;25:72-9. Epub 2018/07/29. doi: 10.1016/j.epidem.2018.05.008. PubMed PMID: 30054196; PubMed Central PMCID: PMCPMC6227246.

13. Lindstrand A, Kalyango J, Alfven T, Darenberg J, Kadobera D, Bwanga F, et al. Pneumococcal carriage in children under five years in Uganda-will present pneumococcal conjugate vaccines be appropriate? PLoS One. 2016;11(11):e0166018. doi: 10.1371/journal.pone.0166018. PubMed PMID: 27829063.

14. Nackers F, Cohuet S, le Polain de Waroux O, Langendorf C, Nyehangane D, Ndazima D, et al. Carriage prevalence and serotype distribution of *Streptococcus pneumoniae* prior to 10-valent pneumococcal vaccine introduction: A population-based cross-sectional study in South Western Uganda, 2014. Vaccine. 2017;35(39):5271-7. Epub 2017/08/09. doi: 10.1016/j.vaccine.2017.07.081. PubMed PMID: 28784282; PubMed Central PMCID: PMCPMC6616034.

15. Coles CL, Kanungo R, Rahmathullah L, Thulasiraj RD, Katz J, Santosham M, et al. Pneumococcal nasopharyngeal colonization in young South Indian infants. Ped Infect Dis J. 2001;20(3):289-95. PubMed PMID: 11303832.

16. Coles CL, Sherchand JB, Khatry SK, Katz J, Leclerq SC, Mullany LC, et al. Nasopharyngeal carriage of *S. pneumoniae* among young children in rural Nepal. Trop Med Int Health. 2009;14(9):1025-33. Epub 2009/07/01. doi: 10.1111/j.1365-3156.2009.02331.x. PubMed PMID: 19563428; PubMed Central PMCID: PMCPMC2770711.

17. Nguyen HAT, Fujii H, Vu HTT, Parry CM, Dang AD, Ariyoshi K, et al. An alarmingly high nasal carriage rate of Streptococcus pneumoniae serotype 19F non-susceptible to multiple beta-lactam antimicrobials among Vietnamese children. BMC Infect Dis. 2019;19(1):241. Epub 2019/03/15. doi: 10.1186/s12879-019-3861-2. PubMed PMID: 30866853; PubMed Central PMCID: PMCPMC6416861.

18. Uddén F, Filipe M, Slotved HC, Yamba-Yamba L, Fuursted K, Pintar Kuatoko P, et al. Pneumococcal carriage among children aged 4 - 12 years in Angola 4 years after the introduction of a pneumococcal conjugate vaccine. Vaccine. 2020;38(50):7928-37. Epub 2020/11/05. doi: 10.1016/j.vaccine.2020.10.060. PubMed PMID: 33143954.

19. Adetifa IM, Antonio M, Okoromah CA, Ebruke C, Inem V, Nsekpong D, et al. Pre-vaccination nasopharyngeal pneumococcal carriage in a Nigerian population: epidemiology and population biology. PLoS One. 2012;7(1):e30548. doi: <https://dx.doi.org/10.1371/journal.pone.0030548>. PubMed PMID: 22291984.

20. Inverarity D, Diggle M, Ure R, Johnson P, Altstadt P, Mitchell T, et al. Molecular epidemiology and genetic diversity of pneumococcal carriage among children in Beni State, Bolivia. Trans R Soc Trop Med Hyg. 2011;105(8):445-51. doi: <https://dx.doi.org/10.1016/j.trstmh.2011.04.013>. PubMed PMID: 21714978.

21. Cardozo DM, Nascimento-Carvalho CM, Andrade AL, Silvany-Neto AM, Daltro CH, Brandao MA, et al. Prevalence and risk factors for nasopharyngeal carriage of Streptococcus pneumoniae among adolescents. Journal of Medical Microbiology. 2008;57(Pt 2):185-9. doi: <https://dx.doi.org/10.1099/jmm.0.47470-0>. PubMed PMID: 18201984.

22. Regev-Yochay G, Raz M, Dagan R, Porat N, Shainberg B, Pinco E, et al. Nasopharyngeal carriage of *Streptococcus pneumoniae* by adults and children in community and family settings. Clin Infect Dis 2004;38(5):632-9. doi: <https://dx.doi.org/10.1086/381547>. PubMed PMID: 14986245.

23. Farida H, Severin JA, Gasem MH, Keuter M, Wahyono H, van den Broek P, et al. Nasopharyngeal carriage of *Streptococcus pneumoniae* in pneumonia-prone age groups in Semarang, Java Island, Indonesia. PLoS One. 2014;9(1):e87431. doi: <https://dx.doi.org/10.1371/journal.pone.0087431>. PubMed PMID: 24498104.

24. Murad C, Dunne EM, Sudigdoadi S, Fadlyana E, Tarigan R, Pell CL, et al. Pneumococcal carriage, density, and co-colonization dynamics: A longitudinal study in Indonesian infants. International journal of infectious diseases : IJID : official publication of the International Society for Infectious Diseases. 2019;86:73-81. doi: <https://dx.doi.org/10.1016/j.ijid.2019.06.024>.

25. Hu J, Sun X, Huang Z, Wagner AL, Carlson B, Yang J, et al. *Streptococcus pneumoniae* and *Haemophilus influenzae* type b carriage in Chinese children aged 12-18 months in Shanghai, China: a cross-sectional study. BMC Infect Dis. 2016;16:149. doi: <https://dx.doi.org/10.1186/s12879-016-1485-3>. PubMed PMID: 27080523.

26. Russell FM, Carapetis JR, Ketaiwai S, Kunabuli V, Taoi M, Biribo S, et al. Pneumococcal nasopharyngeal carriage and patterns of penicillin resistance in young children in Fiji. Ann Trop Paediatr 2006;26(3):187-97. doi: <https://dx.doi.org/10.1179/146532806X120273>. PubMed PMID: 16925955.

27. von Mollendorf C, Dunne EM, La Vincente S, Ulziibayar M, Suuri B, Luvsantseren D, et al. Pneumococcal carriage in children in Ulaanbaatar, Mongolia before and one year after the introduction of the 13-valent pneumococcal conjugate vaccine. Vaccine. 2019;37(30):4068-75. doi: <https://dx.doi.org/10.1016/j.vaccine.2019.05.078>.

28. Dunne EM, Choummanivong M, Neal EFG, Stanhope K, Nguyen CD, Xeuatvongsa A, et al. Factors associated with pneumococcal carriage and density in infants and young children in Laos PDR. PLoS One. 2019;14(10):e0224392. doi: <https://dx.doi.org/10.1371/journal.pone.0224392>.

29. Shiri T, Nunes MC, Adrian PV, Van Niekerk N, Klugman KP, Madhi SA. Interrelationship of *Streptococcus pneumoniae*, *Haemophilus influenzae* and *Staphylococcus aureus* colonization within and between pneumococcal-vaccine naive mother-child dyads. BMC Infect Dis. 2013;13:483. doi: <https://dx.doi.org/10.1186/1471-2334-13-483>. PubMed PMID: 24134472.

30. Vanker A, Nduru PM, Barnett W, Dube FS, Sly PD, Gie RP, et al. Indoor air pollution and tobacco smoke exposure: impact on nasopharyngeal bacterial carriage in mothers and infants in an African birth cohort study. ERJ Open Res. 2019;5(1). Epub 2019/02/12. doi: 10.1183/23120541.00052-2018. PubMed PMID: 30740462; PubMed Central PMCID: PMCPMC6360211.

31. Skosana Z, Von Gottberg A, Olorunju S, Mohale T, Du Plessis M, Adams T, et al. Non-vaccine serotype pneumococcal carriage in healthy infants in South Africa following introduction of the 13-valent pneumococcal conjugate vaccine. S Afr Med J. 2021;111(2):143-8. Epub 2021/05/05. doi: 10.7196/SAMJ.2021.v111i2.14626. PubMed PMID: 33944725.

32. Reis JN, Palma T, Ribeiro GS, Pinheiro RM, Ribeiro CT, Cordeiro SM, et al. Transmission of Streptococcus pneumoniae in an urban slum community. Journal of Infection. 2008;57(3):204-13. doi: <https://dx.doi.org/10.1016/j.jinf.2008.06.017>. PubMed PMID: 18672297.

33. Menezes AP, Azevedo J, Leite MC, Campos LC, Cunha M, Carvalho Mda G, et al. Nasopharyngeal carriage of *Streptococcus pneumoniae* among children in an urban setting in Brazil prior to PCV10 introduction. Vaccine. 2016;34(6):791-7. doi: <https://dx.doi.org/10.1016/j.vaccine.2015.12.042>. PubMed PMID: 26742946.

34. Neves FPG, Cardoso NT, Snyder RE, Marlow MA, Cardoso CAA, Teixeira LM, et al. Pneumococcal carriage among children after four years of routine 10-valent pneumococcal conjugate vaccine use in Brazil: The emergence of multidrug resistant serotype 6C. Vaccine. 2017;35(21):2794-800. doi: 10.1016/j.vaccine.2017.04.019. PubMed PMID: 28431817.

35. Toledo ME, Casanova MF, Linares-Perez N, Garcia-Rivera D, Torano Peraza G, Barcos Pina I, et al. Prevalence of pneumococcal nasopharyngeal carriage among children 2-18 months of age: baseline study pre-introduction of pneumococcal vaccination in Cuba. Ped Infect Dis J. 2017;36(1):e22-e8. doi: 10.1097/inf.0000000000001341. PubMed PMID: 27649366.

36. Rivera-Olivero IA, del Nogal B, Sisco MC, Bogaert D, Hermans PW, de Waard JH. Carriage and invasive isolates of *Streptococcus pneumoniae* in Caracas, Venezuela: the relative invasiveness of serotypes and vaccine coverage. Eur J Clin Microbiol Infect Dis. 2011;30(12):1489-95. doi: <https://dx.doi.org/10.1007/s10096-011-1247-5>. PubMed PMID: 21499972.

37. Verhagen LM, Hermsen M, Rivera-Olivero IA, Sisco MC, de Jonge MI, Hermans PW, et al. Nasopharyngeal carriage of respiratory pathogens in Warao Amerindians: significant relationship with stunting. Trop Med Int Health. 2017;22(4):407-14. doi: 10.1111/tmi.12835. PubMed PMID: 28072501.

38. Karami M, Hosseini SM, Hashemi SH, Ghiasvand S, Zarei O, Safari N, et al. Prevalence of nasopharyngeal carriage of Streptococcus pneumoniae in children 7 to 14 years in 2016: A survey before pneumococcal conjugate vaccine introduction in Iran. Human vaccines & immunotherapeutics. 2019;15(9):2178-82. doi: <https://dx.doi.org/10.1080/21645515.2018.1539601>.

39. Korona-Glowniak I, Malm A. Characteristics of *Streptococcus pneumoniae* strains colonizing upper respiratory tract of healthy preschool children in Poland. ScientificWorldJournal. 2012.

40. Ozdemir B, Beyazova U, Camurdan AD, Sultan N, Ozkan S, Sahin F. Nasopharyngeal carriage of Streptococcus pneumoniae in healthy Turkish infants. Journal of Infection. 2008;56(5):332-9. doi: <https://dx.doi.org/10.1016/j.jinf.2008.02.010>. PubMed PMID: 18377994.

41. Uzuner A, Ilki A, Akman M, Gundogdu E, Erbolukbas R, Kokacya O, et al. Nasopharyngeal carriage of penicillin-resistant *Streptococcus pneumoniae* in healthy children. Turk J Pediatr 2007;49(4):370-8. Epub 2008/02/06. PubMed PMID: 18246737.

42. Ozdemir H, Ciftci E, Durmaz R, Guriz H, Aysev AD, Karbuz A, et al. Risk factors for nasopharyngeal carriage of *Streptococcus pneumoniae* in healthy Turkish children after the addition of heptavalent pneumococcal conjugate vaccine (PCV7) to the national vaccine schedule. Turk J Pediatr. 2013;55(6):575-83. Epub 2014/03/01. PubMed PMID: 24577974.

43. Arvas A, Cokugras H, Gur E, Gonullu N, Taner Z, Bahar Tokman H. Pneumococcal nasopharyngeal carriage in young healthy children after pneumococcal conjugate vaccine in Turkey. Balkan Med J. 2017. doi: 10.4274/balkanmedj.2016.1256. PubMed PMID: 28443585.

44. Neal EFG, Nguyen C, Ratu FT, Matanitobua S, Dunne EM, Reyburn R, et al. A comparison of pneumococcal nasopharyngeal carriage in very young Fijian infants born by vaginal or Cesarean delivery. JAMA Netw Open. 2019;2(10):e1913650. Epub 2019/10/19. doi: 10.1001/jamanetworkopen.2019.13650. PubMed PMID: 31626319; PubMed Central PMCID: PMCPMC6813584.

45. Neal EFG, Flasche S, Nguyen CD, Ratu FT, Dunne EM, Koyamaibole L, et al. Associations between ethnicity, social contact, and pneumococcal carriage three years post-PCV10 in Fiji. Vaccine. 2020;38(2):202-11. doi: <https://dx.doi.org/10.1016/j.vaccine.2019.10.030>.

46. Neal EFG, Nguyen CD, Ratu FT, Dunne EM, Kama M, Ortika BD, et al. Factors associated with pneumococcal carriage and density in children and adults in Fiji, using four cross-sectional surveys. PLoS One. 2020;15(4):e0231041. doi: <https://dx.doi.org/10.1371/journal.pone.0231041>.

47. Ricketson LJ, Wood ML, Vanderkooi OG, MacDonald JC, Martin IE, Demczuk WH, et al. Trends in asymptomatic nasopharyngeal colonization with *Streptococcus pneumoniae* after introduction of the 13-valent pneumococcal conjugate vaccine in Calgary, Canada. Ped Infect Dis J. 2014;33(7):724-30. doi: <https://dx.doi.org/10.1097/INF.0000000000000267>. PubMed PMID: 24463806.

48. Samore MH, Magill MK, Alder SC, Severina E, Morrison-De Boer L, Lyon JL, et al. High rates of multiple antibiotic resistance in *Streptococcus pneumoniae* from healthy children living in isolated rural communities: association with cephalosporin use and intrafamilial transmission. Pediatrics. 2001;108(4):856-65. PubMed PMID: 11581436.

49. Millar EV, O'Brien KL, Zell ER, Bronsdon MA, Reid R, Santosham M. Nasopharyngeal carriage of *Streptococcus pneumoniae* in Navajo and White Mountain Apache children before the introduction of pneumococcal conjugate vaccine. Ped Infect Dis J. 2009;28(8):711-6. Epub 2009/07/14. doi: 10.1097/INF.0b013e3181a06303. PubMed PMID: 19593248.

50. Cheng Immergluck L, Kanungo S, Schwartz A, McIntyre A, Schreckenberger PC, Diaz PS. Prevalence of *Streptococcus pneumoniae* and *Staphylococcus aureus* nasopharyngeal colonization in healthy children in the United States. Epidemiol Infect. 2004;132(2):159-66. PubMed PMID: 15061489.

51. Finkelstein JA, Huang SS, Daniel J, Rifas-Shiman SL, Kleinman K, Goldmann D, et al. Antibiotic-resistant *Streptococcus pneumoniae* in the heptavalent pneumococcal conjugate vaccine era: predictors of carriage in a multicommunity sample. Pediatrics. 2003;112(4):862-9. PubMed PMID: 14523178.

52. Huang SS, Finkelstein JA, Rifas-Shiman SL, Kleinman K, Platt R. Community-level predictors of pneumococcal carriage and resistance in young children. Am J Epidemiol. 2004;159(7):645-54. PubMed PMID: 15033642.

53. Huang SS, Hinrichsen VL, Stevenson AE, Rifas-Shiman SL, Kleinman K, Pelton SI, et al. Continued impact of pneumococcal conjugate vaccine on carriage in young children. Pediatrics. 2009;124(1):e1-11. doi: <https://dx.doi.org/10.1542/peds.2008-3099>. PubMed PMID: 19564254.

54. Moore MR, Hyde TB, Hennessy TW, Parks DJ, Reasonover AL, Harker-Jones M, et al. Impact of a conjugate vaccine on community-wide carriage of nonsusceptible Streptococcus pneumoniae in Alaska. Journal of Infectious Diseases. 2004;190(11):2031-8. doi: <https://dx.doi.org/10.1086/425422>. PubMed PMID: 15529269.

55. Park SY, Moore MR, Bruden DL, Hyde TB, Reasonover AL, Harker-Jones M, et al. Impact of conjugate vaccine on transmission of antimicrobial-resistant *Streptococcus pneumoniae* among Alaskan children. Ped Infect Dis J. 2008;27(4):335-40. doi: <https://dx.doi.org/10.1097/INF.0b013e318161434d>. PubMed PMID: 18316986.

56. Lee GM, Kleinman K, Pelton SI, Hanage W, Huang SS, Lakoma M, et al. Impact of 13-Valent Pneumococcal Conjugate Vaccination on *Streptococcus pneumoniae* Carriage in Young Children in Massachusetts. J Pediatric Infect Dis Soc. 2014;3(1):23-32. doi: 10.1093/jpids/pit057. PubMed PMID: 24567842.

57. Hsu KK, Rifas-Shiman SL, Shea KM, Kleinman KP, Lee GM, Lakoma M, et al. Do community-level predictors of pneumococcal carriage continue to play a role in the conjugate vaccine era? Epidemiol Infect. 2014;142(2):379-87. doi: <https://dx.doi.org/10.1017/S0950268813000794>. PubMed PMID: 23731707.

58. Reisman J, Rudolph K, Bruden D, Hurlburt D, Bruce MG, Hennessy T. Risk factors for pneumococcal colonization of the nasopharynx in Alaska native adults and children. J Pediatric Infect Dis Soc. 2014;3(2):104-11. doi: 10.1093/jpids/pit069. PubMed PMID: 26625363.

59. Wroe PC, Lee GM, Finkelstein JA, Pelton SI, Hanage WP, Lipsitch M, et al. Pneumococcal carriage and antibiotic resistance in young children before 13-valent conjugate vaccine. Ped Infect Dis J. 2012;31(3):249-54. doi: <https://dx.doi.org/10.1097/INF.0b013e31824214ac>. PubMed PMID: 22173142.

60. Koliou MG, Andreou K, Lamnisos D, Lavranos G, Iakovides P, Economou C, et al. Risk factors for carriage of *Streptococcus pneumoniae* in children. BMC Pediatr. 2018;18(1):144. Epub 2018/04/28. doi: 10.1186/s12887-018-1119-6. PubMed PMID: 29699525; PubMed Central PMCID: PMCPMC5921789.

61. Memish ZA, Assiri A, Almasri M, Alhakeem RF, Turkestani A, Al Rabeeah AA, et al. Impact of the Hajj on pneumococcal transmission. Clin Microbiol Infect. 2015;21(1):77.e11-8. doi: <https://dx.doi.org/10.1016/j.cmi.2014.07.005>. PubMed PMID: 25636939.

62. Cohen R, Levy C, Bonnet E, Thollot F, Boucherat M, Fritzell B, et al. Risk factors for serotype 19A carriage after introduction of 7-valent pneumococcal vaccination. BMC Infect Dis. 2011;11:95. doi: <https://dx.doi.org/10.1186/1471-2334-11-95>. PubMed PMID: 21501471.

63. Hoang VT, Dao TL, Ly TDA, Belhouchat K, Chaht KL, Gaudart J, et al. The dynamics and interactions of respiratory pathogen carriage among French pilgrims during the 2018 Hajj. Emerg Microbes Infect. 2019;8(1):1701-10. Epub 2019/11/22. doi: 10.1080/22221751.2019.1693247. PubMed PMID: 31749410; PubMed Central PMCID: PMCPMC6882464.

64. Navne JE, Borresen ML, Slotved HC, Andersson M, Melbye M, Ladefoged K, et al. Nasopharyngeal bacterial carriage in young children in Greenland: a population at high risk of respiratory infections. Epidemiol Infect. 2016;144(15):3226-36. doi: 10.1017/s0950268816001461. PubMed PMID: 27405603.

65. Ansaldi F, de Florentiis D, Canepa P, Zancolli M, Martini M, Orsi A, et al. Carriage of *Streptococcus pneumoniae* 7 years after implementation of vaccination program in a population with very high and long-lasting coverage, Italy. Vaccine. 2012;30(13):2288-94. doi: <https://dx.doi.org/10.1016/j.vaccine.2012.01.067>. PubMed PMID: 22306795.

66. Camilli R, Daprai L, Cavrini F, Lombardo D, D'Ambrosio F, Del Grosso M, et al. Pneumococcal carriage in young children one year after introduction of the 13-valent conjugate vaccine in Italy. PLoS One. 2013;8(10):e76309. doi: <https://dx.doi.org/10.1371/journal.pone.0076309>. PubMed PMID: 24124543.

67. Camilli R, Vescio MF, Giufre M, Daprai L, Garlaschi ML, Cerquetti M, et al. Carriage of *Haemophilus influenzae* is associated with pneumococcal vaccination in Italian children. Vaccine. 2015;33(36):4559-64. doi: <https://dx.doi.org/10.1016/j.vaccine.2015.07.009>. PubMed PMID: 26190092.

68. Zuccotti G, Mameli C, Daprai L, Garlaschi ML, Dilillo D, Bedogni G, et al. Serotype distribution and antimicrobial susceptibilities of nasopharyngeal isolates of *Streptococcus pneumoniae* from healthy children in the 13-valent pneumococcal conjugate vaccine era. Vaccine. 2014;32(5):527-34. doi: 10.1016/j.vaccine.2013.12.003. PubMed PMID: 24342249.

69. Almeida ST, Nunes S, Santos Paulo AC, Valadares I, Martins S, Breia F, et al. Low prevalence of pneumococcal carriage and high serotype and genotype diversity among adults over 60 years of age living in Portugal. PLoS One. 2014;9(3):e90974. doi: <https://dx.doi.org/10.1371/journal.pone.0090974>. PubMed PMID: 24604030.

70. Alfayate Miguélez S, Yague Guirao G, Menasalvas Ruíz AI, Sanchez-Solís M, Domenech Lucas M, González Camacho F, et al. Impact of Pneumococcal Vaccination in the Nasopharyngeal Carriage of Streptococcus pneumoniae in Healthy Children of the Murcia Region in Spain. Vaccines (Basel). 2020;9(1). Epub 2021/01/01. doi: 10.3390/vaccines9010014. PubMed PMID: 33379235; PubMed Central PMCID: PMCPMC7823743.

71. Bogaert D, van Belkum A, Sluijter M, Luijendijk A, de Groot R, Rumke HC, et al. Colonisation by *Streptococcus pneumoniae* and *Staphylococcus aureus* in healthy children. Lancet. 2004;363(9424):1871-2. doi: <https://dx.doi.org/10.1016/S0140-6736(04)16357-5>. PubMed PMID: 15183627.

72. Labout JA, Duijts L, Arends LR, Jaddoe VW, Hofman A, de Groot R, et al. Factors associated with pneumococcal carriage in healthy Dutch infants: the generation R study. Journal of Pediatrics. 2008;153(6):771-6. doi: <https://dx.doi.org/10.1016/j.jpeds.2008.05.061>. PubMed PMID: 18621390.

73. Gils E, Veenhoven R, Rodenburg G, Hak E, Sanders E. Effect of 7-valent pneumococcal conjugate vaccine on nasopharyngeal carriage with *Haemophilus influenzae* and *Moraxella catarrhalis* in a randomized controlled trial. Vaccine. 2011;29(44):7595-8. doi: 10.1016/j.vaccine.2011.08.049. PubMed PMID: CN-00806054.

74. Daana M, Rahav G, Hamdan A, Thalji A, Jaar F, Abdeen Z, et al. Measuring the effects of pneumococcal conjugate vaccine (PCV7) on *Streptococcus pneumoniae* carriage and antibiotic resistance: the Palestinian-Israeli Collaborative Research (PICR). Vaccine. 2015;33(8):1021-6. doi: 10.1016/j.vaccine.2015.01.003. PubMed PMID: 25593104.

75. Mackenzie GA, Leach AJ, Carapetis JR, Fisher J, Morris PS. Epidemiology of nasopharyngeal carriage of respiratory bacterial pathogens in children and adults: cross-sectional surveys in a population with high rates of pneumococcal disease. BMC Infect Dis. 2010;10:304. doi: <https://dx.doi.org/10.1186/1471-2334-10-304>. PubMed PMID: 20969800.

76. Sung RY, Ling JM, Fung SM, Oppenheimer SJ, Crook DW, Lau JT, et al. Carriage of *Haemophilus influenzae* and *Streptococcus pneumoniae* in healthy Chinese and Vietnamese children in Hong Kong. Acta Paediatr. 1995;84(11):1262-7. PubMed PMID: 8580623.

77. Chan KC, Subramanian R, Chong P, Nelson EA, Lam HS, Li AM, et al. Pneumococcal carriage in young children after introduction of PCV13 in Hong Kong. Vaccine. 2016;34(33):3867-74. doi: 10.1016/j.vaccine.2016.05.047. PubMed PMID: 27265449.

78. Otsuka T, Chang B, Shirai T, Iwaya A, Wada A, Yamanaka N, et al. Individual risk factors associated with nasopharyngeal colonization with *Streptococcus pneumoniae* and *Haemophilus influenzae*: a Japanese birth cohort study. Ped Infect Dis J. 2013;32(7):709-14. doi: <https://dx.doi.org/10.1097/INF.0b013e31828701ea>. PubMed PMID: 23411622.

79. Ueno M, Ishii Y, Tateda K, Anahara Y, Ebata A, Iida M, et al. Prevalence and risk factors of nasopharyngeal carriage of *Streptococcus pneumoniae* in healthy children in Japan. Jpn J Infect Dis. 2013;66(1):22-5. PubMed PMID: 23429080.

80. Chang B, Akeda H, Nakamura Y, Hamabata H, Ameku K, Toma T, et al. Impact of thirteen-valent pneumococcal conjugate vaccine on nasopharyngeal carriage in healthy children under 24 months in Okinawa, Japan. Journal of infection and chemotherapy : official journal of the Japan Society of Chemotherapy. 2020;26(5):465-70. doi: <https://dx.doi.org/10.1016/j.jiac.2019.12.009>.

81. Hsieh YC, Chiu CH, Chang KY, Huang YC, Chen CJ, Kuo CY, et al. The impact of the heptavalent pneumococcal conjugate vaccine on risk factors for *Streptococcus pneumoniae* carriage in children. Ped Infect Dis J. 2012;31(9):e163-8. doi: <https://dx.doi.org/10.1097/INF.0b013e31825cb9f9>. PubMed PMID: 22592521.

82. Kuo CY, Hwang KP, Hsieh YC, Cheng CH, Huang FL, Shen YH, et al. Nasopharyngeal carriage of *Streptococcus pneumoniae* in Taiwan before and after the introduction of a conjugate vaccine. Vaccine. 2011;29(32):5171-7. doi: <https://dx.doi.org/10.1016/j.vaccine.2011.05.034>. PubMed PMID: 21621578.

83. World Health Organization. WHO/Who we are/Regional offices 2020 [cited 2020 April 3]. Available from: <https://www.who.int/about/who-we-are/regional-offices>.

84. World Bank Country and Lending Groups [Internet]. World Bank. 2020 [cited 2020 Jan 26].
