# Supplementary material for: Factors associated with pneumococcal nasopharyngeal carriage: A systematic review
Source: PLOS Glob Public Health. 2022 Apr 11;2(4):e0000327. doi: 10.1371/journal.pgph.0000327 (PMC10021834; doi:10.1371/journal.pgph.0000327)
Supplement: S2 Table — (DOCX) [file pgph.0000327.s004.docx]

**S2 Table*.* Exposure variables collected among participants included in studies reporting risk factors for pneumococcal carriage, stratified by World Bank income status, WHO region, and country.**

| **WHO Region^a^** | **Country** | **Ref** | **Age group** | **Characteristics** | | **n (%) ^b^** | | | | | | | | | | | | | | | | | | | | | | |
| --- | --- | --- | --- | --- | --- | --- | --- | --- | --- | --- | --- | --- | --- | --- | --- | --- | --- | --- | --- | --- | --- | --- | --- | --- | --- | --- | --- | --- |
| **Low-income countries^c^ (n = 17)** | | | | | | | | | | | | | | | | | | | | | | | | | | | | |
| Africa | Ethiopia | [1] | ≤ 10 years  (n = 234) | Female sex  Urban residence  Rural residence  Age (years)  < 3  3 – 5  5 - < 8  8 – 10  Family size  < 5  5 +  Siblings < 5 y  Siblings > 5 y  Sleeping with parents  Number of rooms  1  2+  Antibiotic use in two weeks of enrolment  Upper respiratory tract infection  No  Tonsillopharyngitis  Sinusitis  Lower respiratory tract infection  No  Pneumonia  Tuberculosis  Otitis media during enrolment | | 121 (51.7)  133 (56.8)  101 (43.2)  57 (24.4)  54 (24.4)  60 (25.6)  63 (26.9)  90 (38.5)  144 (61.5)  102 (43.6)  156 (66.7)  217 (92.7)  148 (63.2)  86 (36.8)  87 (37.2)  195 (83.3)  16 (6.8)  23 (9.8)  175 (74.8)  27 (11.5)  32 (13.7)  12 (5.1) | | | | | | | | | | | | | | | | | | | | | | |
|  |  | [2] | < 5 years  (n = 361) | Female sex  Age (months)  2 – 23  24 – 41  42 – 59  Urban residence  Rural residence  Attending kindergarten/school  Family size  < 5  > 5  > 1 sibling in the house  Siblings < 5 years  Siblings > 5 years  Bed-sharing with parent/guardian  Number of room(s) in house  1  > 2  PCV immunization status  Three doses of PCV  1 or 2 doses of PCV  0 doses  Pneumonia  Otitis media  Sinusitis | | 155 (42.9)  192 (53.2)  93 (25.8)  76 (21.0)  284 (78.7)  77 (21.3)  16 (4.4)  189 (52.4)  172 (47.6)  258 (71.5)  164 (45.4)  194 (53.7)  294 (81.4)  90 (24.9)  271 (75.1)  249 (69)  25 (6.9)  87 (24.1)  79 (21.9)  12 (3.3)  19 (5.3) | | | | | | | | | | | | | | | | | | | | | | |
|  |  | [3] | 3 – 13 years  (n = 710) | Participant sex  Male  Female  Age (years, mean 95% CI)  Parental income (Birr / month)  < 21.7  21.7 – 43.4  43.41 – 65.2  65.21 – 86.9  > 86.9  Family size (mean, SD)  Number of rooms in the house (mean, SD)  Number of siblings (mean, SD)  Having sibling < 6 years  Co-sleeping with siblings  Passive smoker | | 356 (49.9) (as reported in the paper)  358 (50.3) (as reported in the paper)  8.06 (7.88 – 8.24)  264 (37.2)  202 (28.5)  130 (18.3)  49 (6.9)  65 (9.2)  5.08 (1.9)  2.8 (1.6)  2.34 (1.5)  426 (60)  462 (65.1)  105 (14.8) | | | | | | | | | | | | | | | | | | | | | | |
|  |  | [4] | 3 – 6 years  (n = 710) | Age (years)  3 – 4  5 – 6  Participant sex  Male  Female  Siblings < 5 years  Yes  No  Number of siblings per house  1  > 2  Childcare attendance  Yes  No  Smoker in a house  Yes  No  Sharing bed with parents  Yes  No  Cooking in bedroom  Yes  No  Number room per house  1  > 2  Number of family in the house  < 5  > 5  Previous disease  Yes  No  Previous hospitalization  Yes  No  Vaccination status  Yes  No | | 55 (17.4)  262 (82.6)  154 (48.6)  163 (51.4)  216 (68.1)  1010 (31.9)  43 (19.9)  173 (80.1)  104 (32.8)  213 (67.2)  13 (4.1)  304 (95.9)  213 (67.2)  104 (32.8)  168 (53.0)  149 (47.0)  218 (68.8)  99 (31.2)  210 (66.2)  107 (33.8)  218 (68.8)  99 (31.2)  39 (17.9)  179 (82.1)  300 (94.6)  17 (5.4) | | | | | | | | | | | | | | | | | | | | | | |
|  | Kenya | [5] | ≤ 1 - ⩾ 50 years  (n = 450; n = 864 swabs) | Dry season  Number in sample  Female sex  Carriage of *H. influenzae* in the dry season  Carriage of *H. influenzae* in the rainy season  Rainy season  Number resampled  Age (years), (dry and rainy season combined)  <1  1 – 2  3 – 4  5 – 9  10 – 19  20 – 29  30 – 49  > 50 | | 450 (100)  227 (50.4)  54 (12.0)  74 (16.4)  414 (92.0)  98/864 swabs (11.3)  130/864 swabs (15.0)  121/864 swabs (14.0)  109/864 swabs (12.6)  104/864 swabs (12.0)  102/864 swabs (11.8)  93/864 swabs (10.8)  107/864 swabs (12.4)  (note that 32 individuals moved up one age category between the two surveys) | | | | | | | | | | | | | | | | | | | | | | |
|  |  | [6] | 3-59 months  (n = 2840) | Female sex  Age (months)  3 – 23  24 – 41  42 – 59  Location of residence  Banda ra salama  Chasimba  Gedi  Jaribundi  Junju  Kauma  Kilifi Township  Matsangoni  Mtwapa  Ngerenya  Roka  Sokoke  Takaungu-Mavueni  Tezo  Ziana  Month of sampling  January  February  March  April  May  June  July  August  September  October  November  December  Cough in last two week  Coryza, in the last two weeks  Antibiotics in last two weeks  Fansidar in last two weeks  Hospitalized in last month  A child sleeps in the cooking room  Cooking fuel  Firewood  Gas  Charcoal  Paraffin  Smoker in the house  Household member hospitalized in last month | | 1405 (49.5)  1023 (36.0)  886 (31.2)  931 (32.8)  99 (3.5)  208 (7.3)  138 (4.9)  45 (1.6)  280 (9.9)  102 (3.6)  293 (10.3)  240 (8.5)  132 (4.6)  249 (8.8)  253 (8.9)  136 (4.8)  244 (8.6)  245 (8.6)  176 (6.2)  216 (7.6)  278 (9.8%)  225 (7.9)  220 (7.7)  278 (9.8)  283 (10.0)  271 (9.5)  118 (4.2)  178 (6.3)  326 (11.5)  337 (11.9)  110 (3.9)  1420 (50.0)  1684 (59.3)  111 (3.9)  30 (1.1)  8 (0.3)  496 (17.5)  2761 (97.2)  10 (0.4)  60 (2.1)  9 (0.3)  517 (18.2)  18 (0.6) | | | | | | | | | | | | | | | | | | | | | | |
|  | Niger | [7] | 0 - 2 years  (n = 1200) | Age (months) median (95% CI)  Age > 3 months  Birthweight (grams) mean (min – max)  Breastfeeding  Presence of at least 1 sibling aged < 6 years  Smoking environment  Antibiotic treatment three months to 7 days before enrolment  History of infection within the last three months  Attendance at a childcare center or family  Male sex  Being premature  Ethnic group of mothers of study subjects  Zarma-Sonrai  Hausa  Kanouri  Burkinbase  Gurmantche  Fulan  Togolese  Tuareg  Beninoise | | 858 (71.5)  6 (6.9 – 7.5)  2999 (1320 – 4350)  1182 (98.5)  673 (56.1)  469 (39.1)  155 (12.9)  562 (46.8)  126 (10.5)  629 (52.4)  97 (8.1)  589 (49.1)  310 (25.8)  18 (1.5)  36 (3)  16 (1.3)  34 (2.8)  26 (2.2)  95 (7.9)  25 (2.1) | | | | | | | | | | | | | | | | | | | | | | |
|  | The Gambia | [8] | > 30 months  (n = 636) ^d^ | Female sex  Individuals per village, median (IQR)  Age (years, median (IQR)  Age groups (years)  2.5 - < 5  5 - < 15  > 15  Number of years at school  None  < 1 year  1 – 6 years  7 – 10 years  > 10 years  Able to read  Occupation  Farmer  Housewife  Student  Unemployed  Other  Smokes  Smoker in the household | | 323 (50.8)  41 (25, 57)  11.0 (4.6, 25.0)  184 (28.9)  208 (32.7)  244 (38.4)  307/635 (48.3)  81/635 (12.8)  151/635 (23.8)  72/635 (11.3)  24/635 (3.8)  229/635 (36.1)  111 (17.5)  38 (6.0)  257 (40.4)  117 (18.4)  113 (17.8)  34 (5.3)  252/631 (39.9) | | | | | | | | | | | | | | | | | | | | | | |
|  |  | [9] | ≥ 18 years  (n = 847) ^d^ | Age (years)  18 – 24  25 – 39  >40  Participant sex  Male  Female  Smoke (only men)  No  Yes  Smoke (only men)  No  Cigarette  Tobacco  Another smoker in the household  None  Cigarette  Tobacco  Both  Occupation  Farmer  Other  Schooling (attends or attended)  No  Yes  Antibiotic (among those who attended clinic n = 140)  No  Yes  Common cold  No  Yes  Sits at open fire (number of times per day)  No  Once / twice  Three or more | | 257 (30.3)  313 (37.0)  277 (32.7)  265 (31.3)  582 (68.7)  740/842 (87.9)  102/842 (12.1)  740/832 (88.9)  77 / 832 (9.3)  15/832 (1.8)  278/817 (34.0)  431/817 (52.8)  99/817 (12.1)  9/817 (1.1)  639/839 (76.2)  200/839 (23.8)  138/843 (16.4)  705/843 (83.6)  95/140 (67.9)  45/140 (32.1)  570/835 (68.3)  265/835 (31.7)  217/817 (26.6)  292/817 (35.7)  308/817 (37.7) | | | | | | | | | | | | | | | | | | | | | | |
|  |  | [10] | Mother -neonate dyads (n = 374) ^d^ | Neonates  Female sex  Birth weight  < 2.5 kg  > 2.5 kg  Gestational age < 37 weeks  *S. aureus* carriage at day 28  Mothers  Age < 25 years  Pneumococcal carrier at day 28  Pneumococcal carrier at day 14 and day 28  Pneumococcal carrier day 28, non-carrier at day 14  Pneumococcal carrier during the neonatal period  Carrier of *S. aureus* at day 28  Ethnicity  Mandinka  Wollof  Jola  Fula  Others  > 1 year(s) of schooling  Can read  Can write  Household  Smoker in house  Who bathes child?  Mother  Other  Other children in the household  > 1 Number of children at school  Season  Rainy  Dry | | 173/367 (47.1)  22/373 (5.9)  351/373 (94.1)  219/371 (59.0)  132 (35.3)  161/373 (43.2)  91 (24.3)  41 (11.0)  48 (12.8)  173 (46.3)  99 (26.5)  165 (44.1)  42 (11.2)  52 (13.9)  56 (15.0)  59 (15.8)  175/359 (48.7)  129 (34.5)  130 (34.8)  31 (8.3)  203/364 (55.8)  161/364 (44.2)  302 (80.7)  191/372 (51.3)  109 (29.1)  265 (70.9) | | | | | | | | | | | | | | | | | | | | | | |
|  |  | [11] | < 5 & ⩾ 50 years  (n = 2972) ^d^ | Female sex  Age (years), median (range)  Ethnicity  Jola  Mandinka  Other  Visited health center within 30 days before the survey  Antibiotics before the survey | | 1501 (50.5)  15 (0 – 110)  2241 (75.4)  505 (17.1)  226 (7.6)  556 (18.7)  16/556 (2.9) | | | | | | | | | | | | | | | | | | | | | | |
|  | Uganda | [12], with participant characteristics reported in  [13] | All ages (n = 566)^d^ | Female sex  Age (years)  < 2  2 – 4  5 – 9  10 – 14  15 – 24  25 – 34  35 – 44  45 – 54  55 – 64  > 65  Occupation / daily activity  Pre-school child  Student  Office work  Shop worker  Agriculture  Other manual work  At home  Unemployed  Retired  Other/unreported  Do not know  Day of the week  Weekday  Sunday  Travel outside village/town in previous 24 hours  Yes  No  Number of casual contacts  < 10  10 – 19  > 20  Do not know | | 330 (58.3)  61 (10.8)  57 (10.1)  74 (13.1)  51 (9.0)  91 (16.1)  57 (10.1)  55 (9.7)  46 (8.1)  26 (4.6)  48 (8.5)  93/564 (16.5)  166/564 (29.4)  4/564 (0.7)  34/564 (6.0)  106/564 (188)  40/564 (7.1)  60/564 (10.6)  11/564 (2.0)  8/564 (1.4)  41 (7.3)  1 (0.2)  441 (77.9)  125 (22.1)  139 (24.6)  427 (75.4)  315 (55.7)  119 (21.0)  56 (9.9)  76 (13.4) | | | | | | | | | | | | | | | | | | | | | | |
|  |  | [14] | < 5 years  (1611) ^d^ | Female sex  Age (months), mean  Wealth quintile  Poorest  Poorer  Poor  Less poor  Least poor  On schedule for vaccination  Ill in last two weeks  Pneumonia symptoms  Other symptoms  Fever  Cough  Diarrhea  Vomiting  Difficult breathing  Fast breathing  Convulsions  Other  Treatment with any antimalarials  Coartem  Chloroquine  Quinine  Fansidar  Treatment with any oral antibiotics | | 808 (50.2)  34.9  226/1410 (16.0)  315/1410 (22.3)  379/1410 (26.9)  300/1410 (21.3)  190/1410 (13.5)  193/231 (83.5)  1249/1610 (77.6)  155/1533 (10.1)  1138/1525 (74.6)  744/1525 (48.8)  959/1525 (62.9)  301/1525 (19.7)  236/1525 (15.5)  180/1532 (11.7)  155/1533 (10.1)  39/1533 (2.5)  109/1525 (7.1)  617/1611 (38.3)  401/1515 (26.5)  120/1515 (7.9)  112/1515 (7.4)  24/1515 (1.6)  493/1611 (30.6) | | | | | | | | | | | | | | | | | | | | | | |
|  |  | [15] | All ages  (n = 1346) | Female sex  Age group (years)  < 2  2 – 4  5 – 14  > 15  Household size, median (range)  Oral antibiotic taken in past two weeks  Respiratory symptoms in the past two weeks | | 698 (51.9)  387 (28.8)  217 (16.1)  417 (31.0)  325 (24.1)  5 (1 – 18)  168 (12.5)  182 (13.5) | | | | | | | | | | | | | | | | | | | | | | |
| South-East Asia | India | [16] | 2 - 2.5 months (n = 464 at baseline) | Female sex  Infant fed colostrum  History of night blindness during pregnancy  Fuel  Wood  Biogas/kerosene  Total no. of cigarettes smoked/day  < 20  > 20  Religion  Hindu  Muslim/Christian  Caste  Forward caste  Backward caste  Most backward/schedule  Mother’s education > 1 year  Conveyance  None  Bicycle  Followed up at 4 months of age  Follow up at 6 months of age | | 200 (43.3)  380 (81.9)  41 (8.8)  439 (94.6)  25 (5.4)  412 (88.8)  52 (11.2)  420 (90.5)  44 (9.5)  19 (4.1)  125 (26.9)  320 (69.0)  255 (55.0)  277 (59.7)  187 (40.3)  404 (87.1)  352 (75.9) | | | | | | | | | | | | | | | | | | | | | | |
|  | Nepal | [17] | Healthy 1-35 months  (n = 550)^d^ | Cohort  Number in sample  Female sex  Aged < 12 months  Ethnic group  Pahadi  Madeshi  Religion  Hindu  Muslim  Hindu caste  Brahmin/Chhetri  Vaiysha/Shudra  > 1 sibling < 5 years old  Mother with < 1 year of education  Mother smokes cigarettes / bedis  Electricity in household  Low socio-economic index  Family owns land  No latrine in household  The child received zinc supplements | | Healthy children  550  282 (51.3)  149 (27.1)  127 (23.1)  423 (76.9)  490 (89.1)  60 (10.9)  40/490 (12.2)  430/490 (87.8)  395/526 (75.1)  439/548 (80.1)  51/500 (10.2)  108 (19.6)  227 (41.3)  366/549 (66.7)  491 (89.3)  292 (53.1) | | | | | | | | | | | | | | | | | | | | | | |
| Western Pacific | Vietnam | [18] | 6 months - < 5 years  (n = 817) | Female sex  Age group  6 – 11 months  1 – 2 years  > 3 years  Childcare attendance  Acute respiratory infection  Living in a large family (> 5 persons)  Prior antibiotic use  Season  Apr-Jun 2008  Jul – Sep 2008  Oct-Dec 2008  Jan – Mar 2009 | | 345 (42.2)  186 (22.8)  472 (57.8)  159 (19.5)  412 (50.4)  331 (40.5)  378 (46.3)  588 (72.0)  107 (13.1)  447 (54.7)  131 (16.)  132 (16.2) | | | | | | | | | | | | | | | | | | | | | | |
| **Lower-middle-income countries^c^ (n = 11)** | | | | | | | | | | | | | | | | | | | | | | | | | | | | |
|  | Angola | [19] | 4 – 12 years  (n = 940) | Group  Number (n)  Age (years)  Female sex  BMI  Chronic ear / auditory or respiratory tract symptoms  Ongoing infectious symptoms  Antibiotic treatment last month  Vaccinated according to schedule  Number of children in the household  Smoker in the household  Access to grid electricity  Access to running water  Cooking fuel used in household  Coal  Gas  Electricity | Missing  -  0 (0.0)  0 (0.0)  1 (0.1)  167 (17.8)  134 (14.3)  142 15.1)  144 (15.3)  4 (0.4)  2 (0.2)  21 (2.2)  490 (52.1)  166 (17.7)  -  - | Total  940  8 (7 – 10)  444 (47)  15.6 (14.5–16.8)  49 (6.0)  59 (7.0)  62 (8.0)  642 (81.0)  4 (3 – 5)  122 (13.0)  696 (76)  76 (17)  2 (0.3)  643 (99.5)  1 (0.2) | | Luanda (community)  654  9 (7 – 10)  323 (49.0)  15.6 (14.5 – 16.8)  12 (2.0)  8 (1.0)  19 (4.0)  403 (79)  4 (3 – 5)  64 (10.0)  579 (92.0)  40 (13.0)  2 (0.3)  643 (99.5)  1 (0.2) | | | | | | | | | | Luanda (hospital)  126  8 (6 – 10)  44 (35.0)  15.4 (14.2 – 17.2)  21 (19.0)  39 (66.0)  43 (35.0)  114 (93.0)  3 (3 – 5)  14 (11.0)  117 (93.0)  30 (73.0)  0 (0.0)  124 (100.0)  0 (0.0) | | | | | | | Saurimo (community)  160  9 (7 – 11)  69 (43.0)  15.7 (14.5 – 16.7)  16 (11.0)  12 (8.0)  0 (0.0)  125 (78.0)  4 (3 – 5)  44 (28.0)  160 (100.0)  6 (6.0)  0 (0.0)  4 (100.0)  0 (0.0) | | | |
|  | Nigeria | [20] | All ages  (n = 1025) ^d^ | Female sex  Age, median years (IQR)  Children  Adults  Yoruba people  Antibiotic use in the month before the survey (children)  Antibiotic use in the month before the survey (adults)  Self-prescription of antibiotics; drug store purchase (adults) | | 652 (63.6)  4.4 (1.3 – 30)  654 (63.8)  371 (36.2)  952 (92.8)  100/641 (15.6)  76/361 (17.2)  50/75 (66.7) | | | | | | | | | | | | | | | | | | | | | | |
| Americas | Bolivia | [21] | May – Jun 2007  (n = 601) ^d^ | Female sex  Age in Trinidad (years)  0 – 5  > 6  Age in Riberalta (years)  0 – 5  > 6  Household size  2 – 5  6 – 9  > 10  Children aged 2 – 5 years at home  Children aged < 2 years at home  Live with a smoker  Riberalta residence  Trinidad residence | | 248 (41.3)  59/493 (12.0)  434/493 (88.0)  27/107 (25.2)  80/107 (74.8)  142/544 (26.1)  254/544 (46.7)  148/544 (27.2)  249/550 (45.3)  202/549 (36.8)  250/546 (45.8)  107/600 (17.8)  493/600 (82.2) | | | | | | | | | | | | | | | | | | | | | | |
|  | Brazil | [22] | 10-19 years  (n = 1013) | Female sex  Age (years), mean (SD)  Passive exposure to cigarette smoke  Children < 5 years in the household  Children < 5 years in the sleeping room  Upper respiratory tract infection during recruitment  Upper respiratory tract infection in a household contact  Occurrence during the last year of:  Upper respiratory tract infection  Otitis  Rhinitis  Sinusitis  An episode of acute asthma  Pneumonia  Hospitalization  Antimicrobial use during previous three months | | 540 (53.3)  14.6 (2.3)  347 (34.3)  199 (19.6)  70 (6.9)  266 (26.3)  198 (19.5)  837 (82.6)  182 (18.0)  96 (9.5)  91 (9.0)  37 (3.7)  16 (1.6)  53 (5.2)  81 (8.0) | | | | | | | | | | | | | | | | | | | | | | |
| Eastern Mediterranean | West Bank and Gaza | [23] | < 5.5 years  (n = 379) ^d^ | Female sex  Age  < 6 months  6 – 11 months  1 year  2 years  3 – 5.5 years  Pneumococcal carriage by parent  Childcare attendance  Received antibiotics in past six months  Hospitalization in previous six months  Smoker  Current breastfeeding (among children < 2 years) | | 192 (50.7)  30 (7.9)  60 (15.8)  115 (30.3)  75 (19.8)  99 (26.1)  30/375 (8.0)  13 (3.4)  142 (37.5)  52 (13.7)  116 (31.1)  103/198 (52.0) | | | | | | | | | | | | | | | | | | | | | | |
| South East Asia | Indonesia | [24] | 6-60 months (n = 243)^d^ and 45 - 75 years  (n = 253)^d^ | Age cohort  Female sex  Age (years), median (range)  Urban residence  Rural residence  Food hygiene  Good  Poor  Water hygiene  Good  Poor  Crowding  Mosquito coil use  Active smoking  Passive smoking  Contact with toddler(s) | | 6 – 60 months  125 (51.4)  2.1 (0.5 – 5)  180 (74.1)  63 (25.9)  81 (33.3)  162 (66.7)  114 (46.9)  129 (53.1)  100/229 (43.7)  59 (24.3)  0 (0.0)  146 (60.1)  132 (54.3) | | | | | | | | | | | | | | 45 – 75 years  159 (62.8)  55 (45 – 75)  186 (73.5)  67 (26.5)  107 (42.3)  146 (57.7)  119 (47.0)  134 (53.0)  80/237 (33.8)  83 (32.8)  53 (20.9)  147 (58.1)  137 (54.2) | | | | | | | | |
|  |  | [25] | 2 – 12 months  (n – 200) ^d^ | Participant sex  Male  Female  Age (months, median, IQR)  Weight (kg, median, IQR)  Height (cm median, IQR)  Weight-for-length Z score (median, IQR)  Ethnicity  Sudanese  Javanese  Other  Paternal education  Elementary school  Junior high school  Senior high school  University  Maternal education  None  Elementary school  Junior high school  Senior high school  University  Parental monthly income  Declined to answer  < Regional minimum salary  > Regional minimum salary  Number of children < 5 years old in the household  1  2  3  4  Cigarette smoker in the household  No  Yes  Breastfeeding at 12 months  No  Yes | | Total (n = 200)  115 (57.5)  85 (42.5)  2.1 (2.0 – 2.3  5.2 (4.8 – 5.8)  58 (57 – 60)  -0.4 (-1.2 – 0.2)  162 (81.0)  35 (17.5)  3 (1.5)  23 (11.5)  53 (26.5)  99 (49.5)  25 (12.5)  1 (0.5)  23 (11.5)  51 (25.5)  105 (52.5)  20 (10.0)  2 (1.0)  159 (79.5)  39 (19.5)  109 (54.5)  64 (32.0)  18 (19.0)  9 (4.5)  57 (28.5)  143 (71.5)  19/198 (9.6)  179/198 (90.4) | | | | | | | | | Urban (n = 98)  64 (65.3)  34 (35.7)  2.0 (2.0 – 2.3)  5.2 (4.8 – 5.8)  58 (56 – 60)  -0.4 (-1.2 – 0.1)  68 (69.4)  29 (29.6)  1 (1.0)  6 (6.1)  16 (16.3)  59 (60.2)  17 (17.3)  1 (1.0)  4 (4.1)  15 (15.3)  66 (67.3)  12 (12.2)  1 (1.0)  73 (74.5)  24 (24.5)  39 (39.8)  39 (39.8)  13 (13.3)  7 (7.1)  36 (36.7)  62 (63.3)  9/96 (9.4)  87/91 (95.6) | | | | | | | | | Semi-rural (n = 102)  51 (50.0)  51 (50.0)  2.2 (2.0 – 2.5)  5.2 (4.8 – 5.8)  58 (57 – 59)  -0.4 (01.2 – 0.4)  94 (92.2)  6 (5.9)  2 (2.0)  17 (16.7)  37 (36.3)  40 (39.2)  8 (7.8)  0 (0.0)  19 (18.6)  36 (35.3)  39 (38.2)  8 (7.8)  1 (1.0)  86 (84.3)  15 (14.7)  70 (68.6)  25 (24.5)  5 (4.9)  2 (2.0)  21 (20.6)  81 (79.4)  10 (9.8)  92 (90.2) | | | | |
| Western Pacific | China | [26] | 12-18 months  (n = 614) | Female sex  Season of enrolment  Autumn  Spring  Age (months)  12  13 – 14  15 – 16  17 – 18  Age of mother at childbirth (years)  18 – 24  25 – 29  30 – 34  35 – 44  Local household residency  Non-local household residency  Urban residence  Suburban residence  Rural residence  Household income  < ¥2000  ¥2000 - ¥4999  > ¥5000  Size of house  < 18 m^2^  18 – 29 m^2^  30 – 69 m^2^  > 70 m^2^  > 1 sibling  Mother’s education  Primary school or less  Junior high school  High school / vocational  College or more | | 276 (45.0)  306 (49.8)  308 (50.2)  170 (27.7)  184 (30.0)  158 (25.7)  102 (16.6)  179 (29.2)  257 (41.9)  123 (20.0)  55 (9.0)  160 (26.1)  454 (73.9)  422 (68.7)  126 (20.5)  66 (10.7)  232 (37.8)  286 (46.6)  96 (15.6)  145 (23.6)  120 (19.5)  156 (25.4)  193 (31.4)  207 (33.7)  73 (11.9)  286 (46.6)  134 (21.8)  121 (19.7) | | | | | | | | | | | | | | | | | | | | | | |
|  | Fiji | [27] | 3 - 13 months  (n = 440)^d^ | Female sex  Age (months), median (range)  Ethnicity  iTaukei  Fijian of Indian Descent  Other  Urban residence  Rural residence  The family income per annum, median (range)  Low socio-economic status (<FJ $6000 per annum)  Birthweight, median (range)  Low birthweight (< 2500 grams)  Malnourished (Z-score < - 2)  Number children living in the household, median (range)  Number children < 5 years living in the household, median (range)  Cigarette smokers at home  Cooking fuel used  Gas  Electricity  Wood  Kerosene  If wood or kerosene, cooking indoors  Hay fever symptoms  Symptoms of acute respiratory illness  Hospitalization within the last three months  Antibiotics taken in last month  Currently breastfeeding | | 206/438 (47.0)  9.4 (2.5 – 13.3)  283/438 (64.6)  122/438 (27.9)  33/438 (7.5)  343 (77.9)  97 (22.1)  7020 (0 – 91000  158 (35.9)  3250 (500 – 48500)  50 (11.3)  34 (7.7)  2 (1 – 11)  1 (1 – 5)  239 (54.6)  263 (60.1)  212 (48.4)  224 (51.1)  343 (78.3)  224 (51.1)  55 (12.5)  64 (30.2)  13 (2.9)  60 (13.7)  334 (76.3) | | | | | | | | | | | | | | | | | | | | | | |
|  | Mongolia | [28] | 5 – 8 weeks and 12 – 23 months  (n = 1550) ^d^ | Age group  Period  Number  Median age (weeks, median, IQR)  Median age (months, median, IQR)  Female sex  Parent primary caregivers  > two children < 5 years in the household  Exposure to household cigarette smoke  Mother completed university  Crowding (> 3 people per room)  Above minimum income  Born by Caesarean section  Breastfeeding at the time of the survey  Living in informal housing (ger)  Member of household treated for tuberculosis  The main source of cooking fuel  Electricity  Coal  Wood  Gas  Other  Previous admission for pneumonia  Vaccinated with any number of PCV13 doses  Received one dose  Received two doses  Received three doses | | 5 – 8 weeks | | | | | | | | | | | | | | 12 – 23 months | | | | | | | | |
|  |  |  |  |  |  | pre-PCV13  n = 461  6.4 (5.3 – 7.7)  -  222 (48.2)  457 (99.01)  177 (38.4)  28/441 (6.3)  260/455 (57.1)  172 (37.3)  287/435 (66.0)  141/457 (30.9)  439 (95.2)  153 (33.2)  15 (3.3)  184 (39.9)  253 (54.9)  16 (3.5)  5 (1.1)  3 (0.7)  7/457 (1.5)  -  -  - | | | post-PCV13  n = 500  6.1 (5.3 – 7.0)  -  244 (48.8)  495 (99.0)  217 (43.4)  33 (6.6)  287/499 (57.5)  172 (34.4)  188/489 (38.4)  147 (28.4)  482 (96.4)  154 (30.8)  12 (2.4)  228 (45.6)  239 (47.8)  33 (6.6)  0 (0.0)  0 (0.0)  9/7 (1.4)  -  -  - | | | | | | | | | | | pre-PCV13  n = 489  -  16.4 (14.1 – 20.7)  257 (52.6)  410 (83.8)  156 (31.9)  55/488 (11.3)  283/487 (58.1)  206 (42.1)  277/465 (59.6)  142 (29.0)  329 (67.3)  189 (38.7)  19 (3.9)  186 (38.0)  279 (57.1)  16 (3.3)  2 (0.4)  6 (1.2)  124 (25.4)  0 (0.0)  0 (0.0)  0 (0.0) | | | | | | post-PCV13  n = 500  -  15.9 (14.1 – 19.5)  241 (48.2)  423 (84.6)  183 (36.6)  54 (10.8)  296 (59.2)  176 (35.2)  188/486 (38.7)  161 (32.2)  341 (68.2)  156 (31.2)  6 (1.2)  218 (43.6)  230 (46.0)  37 (7.4)  1 (0.2)  14 (2.8)  126 (25.2)  21/439 (4.8)  339/439 (77.2)  79/439 (18.0) | | |
|  | Lao People’s Demographic Republic | [29] | 5 – 8 weeks and 12 – 23 months  (n = 2009) ^d^ | Numbers  Survey  Pre-PCV13  Two years post-PCV13  Age (weeks, median IQR)  Age (months, median IQR)  Male sex  Ethnicity  Lao Loum  Lao Thung  Hmong  Other  Rural residence  Upper respiratory tract infection symptoms  Antibiotic use in the previous two weeks  Exposure to household cigarette smoke  Primary fuel source  Wood  Charcoal  Kerosene  Gas  Electricity  > two children < 5 years in the household  Below poverty line  Delivery by Caesarean section  Current breastfeeding  PCV13 vaccinated | | 5 – 8 week  n = 999  498 (49.9)  501 (50.2)  6.7 (6.5 – 7.0)  -  509/998 (51.0)  964/998 (96.6)  3/998 (0.3)  27/998 (2.7)  4/998 (0.4)  70 (7.0)  162 (16.2)  29/997 (2.9)  366/996 (36.8)  164 (16.4)  510 (51.1)  1 (0.1)  179 (17.9)  145 (14.5)  409/998 (41.0)  109 (10.6)  238 (23.8)  844/998 (84.6)  0 (0.0) | | | | | | | | | | | | | | | 12 – 23 months  n = 1010  503 (49.8)  507 (50.2)  -  16.6 (15.6 – 20.0)  484 (47.9)  963 (95.4)  13 (1.3)  30 (3.0)  4 (0.4)  470 (46.5)  625 (61.9)  405/1007 (40.2)  440/1007 (43.7)  227/1007 (27.5)  482/1007 (47.8)  1/1007 (0.1)  153/1007 (15.2)  95/1007 (9.4)  349 (34.6)  70 (6.9)  179 (17.7)  201 /1009 (19.9)  448/1000 (44.8) | | | | | | | |
| **Upper-middle-income countries^c^ (n = 18)** | | | | | | | | | | | | | | | | | | | | | | | | | | | | |
| Africa | South Africa | [30] | 6-12 weeks to 2 years  (n = 251) | Demographics not reported | | Demographics not reported | | | | | | | | | | | | | | | | | | | | | | |
|  |  | [31] | Birth – 12 months  (n = 986) | Public primary health clinic  Number of infants; sets of twins  Female sex  Gestation at delivery, weeks  Birthweight, grams  Weight-for-age Z score  Birthweight < 2500 grams  Birth < 37 weeks gestation  Feeding at 6 months  Exclusive breastfeeding  Mixed  Not breastfeeding  Duration of exclusive breastfeeding (months)  Childcare attendance  6 months of age  12 months of age  Additional child < 5 years of age in the household  Respiratory infection in the prior month  6 months of age  12 months of age  Antibiotic use in prior six months  6 months of age  12 months of age | | Mbekweni  534 (54); 4  280 (52)  39 (38 – 40)  3180 (2810 – 3460)  -0.4 (-1.3 – 0.2)  55 (10)  16 (3)  76 (14)  89 (17)  369 (69)  1.1 (0.5 – 3.0)  17 (3)  69 (13)  455 (85)  40 (7)  29 (5)  25 (5)  14 (3) | | | | | | | | | Newman  452 (46); 0  170 (45)  39 (37 – 40)  2990 (2630 – 3340)  -0.7 (-1.4 – -0.1)  83 (18)  19 (4)  66 (15)  170 (38)  216 (48)  1.6 (0.9 – 3.0)  28 (6)  90 (20)  396 (88)  37 (8)  22 (88)  38 (8)  13 (3) | | | | | | | | | | Total  986 (100)  484 (49)  39 (38 – 40)  3080 (2770 – 3415)  -0.6 (-1.3 – 0.0)  138 (14)  35 (4)  142 (14)  259 (26)  585 (59)  1.4 (0.7 – 3.0)  45 (5)  159 (16)  851 (86)  77 (8)  51 (5)  63 (6)  27 (3) | | | |
|  |  |  | Mothers of infant participants  (n = 982) | Public primary health clinic  Number of mothers  Age at enrolment (years, median, IQR)  HIV-infected  Unemployed  Socio-economic score (quartile)  Lowest  Low-moderate  Moderate-high  Highest  Household members (median, IQR)  Married / cohabiting | | Mbekweni  530 (54%)  26.9 (22.3 – 31.6)  193 (36)  400 (75)  157 (30)  150 (28)  126 (24)  97 (18)  4 (3 – 6)  188 (35) | | | | | | | | | Newman  452 (46%)  24.6 (21.3 – 29.1)  13 (3)  325 (72)  80 (18)  109 (24)  127 (28)  136 (30)  5 (4 – 7)  194 (43) | | | | | | | | | | Total  982 (100)  25.8 (22.0 – 30.6)  206 (21)  724 (74)  237 (24)  259 (26)  253 (26)  233 (24)  4 (3 – 6)  382 (39) | | | |
|  |  | [32] | 7 – 14 years  (n = 316) | Participant sex  Male sex  Female sex  Not known  Age group  ~6 weeks – 14 weeks  9 months  18 months  5 years  Specimen type (nasopharyngeal swabs)  Vaccination  Yes  No  No vaccination records  Previous illnesses  Yes  No  No record  Childcare attendance  Yes  No  No data  Siblings < 5 years  Yes  No  No data  Exposure to smoking  Yes  No  No data | | 158 (50.0)  147 (46.5)  11 (3.5)  153 (48.4)  61 (19.3)  63 (19.9)  39 (12.3)  All samples  267 (84.5)  47 (14.9)  2 (0.6)  97 (30.7)  193 (61.1)  13 (4.1)  71 (22.4)  232 (73.2)  13 (9.4)  195 (61.7)  101 (32.0)  20 (6.3)  192 (60.7)  115 (36.4)  9 (2.9) | | | | | | | | | | | | | | | | | | | | | | |
| Americas | Brazil | [33] | < 2 years - > 17 years  (n = 262) | Female sex  Age (years)  < 2  2 – 4  5 – 17  >17  Chronic underlying illness  Upper respiratory tract illness in last month  Antibiotic use in the last month  Breastfeeding  Childcare attendance  School attendance  Smoking  Household contact who is a smoker  Number of household contacts  < 5  5 – 10  > 10  Household contact with age < 5 years  Household contact with age < 10 years  Household contact with pneumococcal carriage | | 149 (56.9)  15 (5.7)  35 (13.4)  95 (36.3)  117 (44.7)  44 (16.8)  87 (33.2)  26 (9.9)  8 (3.1)  10 (3.8)  80 (30.5)  21 (8.0)  112 (42.7)  21 (8.0)  157 (59.9)  84 (32.1)  198 (75.6)  238 (90.8)  234 (89.3) | | | | | | | | | | | | | | | | | | | | | | |
|  |  | [34] | 1 - 48 months  (n = 203 children; % reported is for number of swabs for each variable) | Female sex  Age (months)  < 6  6 – 11  12 – 23  24 – 35  36 – 47  48 - 59  Ethnicity  Mixed  White  Black  Childcare attendance  Upper respiratory tract infection in the last month  Exposure to cigarette smoking  Antibiotic use in the last month  Number of children < 2 years living in the same household  None  One contact  Three contacts  Number of residents/number of rooms, mean (SD)  Number of residents/number of beds, mean (SD) | | 371/718 (51.7)  50/721 (6.9)  93/721 (12.9)  145/721 20.1)  132/721 (18.3)  173/721 (24.0)  128/721 (17.8)  505/717 (70.4)  59/717 (8.2)  153/717 (21.3)  28/713 (3.9)  421/708 (59.5)  231/721 (32.0)  58/711 (8.2)  288/713 (40.4)  358/713 (50.2)  67/713 (9.4)  3.3 (+ 1.7)  3.5 (+ 1.8) | | | | | | | | | | | | | | | | | | | | | | |
|  |  | [35] | < 6 years attending private or public clinic for well child or sick visit (n = 522) | Clinic  Sample size  Female sex  Age (years)  0 - < 1  1 - < 2  2 - < 3  3 - < 4  4 - < 5  5 - < 6  Self-reported ethnicity  White  Non-white (Pardo, Black, Asian, or Mestizo)  Blank  Symptoms of fever, coryza, sneezing, cough, expectoration, fatigue, breathlessness, or hypoactivity  Asthma/bronchitis  Rhinitis  Previous pneumonia  Any antibiotic use in the previous two weeks  Hospitalization (> 48 hours) in the past year  Attends childcare  Location of primary residence  Inside a slum  Outside a slum  Not reported  Number of people living in their home  2 or 3  4  5+  Not reported  Has at least one sibling under 6 years old  The family receives governmental support funding  Per-capita household monthly income (US$)  < 130  ≥ 131-350  ≥ 350-875  > 875 | | Private  238 (45.6)  120 (50.4)  142 (59.7)  45 (18.9)  24 (10.1)  14 (5.9)  12 (5.0)  1 (0.4)  166 (69.7)  72 (30.3)  0 (0.0)  106 (44.5)  13 (5.5)  31 (13.0)  8 (3.4)  25 (10.5)  9 (3.8)  62 (26.1)  11 (4.6)  227 (95.4)  0 (0.0)  127 (53.4)  90 (37.8)  21 (8.8)  0 (0.0)  43 (18.1)  2 (0.8)  9 (3.8)  31 (13.0)  96 (40.3)  102 (42.9) | | | | | | | | | | | | | | Public  284 (54.4)  148 (52.1)  67 (23.6)  67 (23.6)  56 (19.7)  42 (14.8)  36 (12.7)  16 (5.6)  99 (34.9)  183 (64.4)  2 (0.7)  108 (38.0)  43 (15.1)  44 (15.5)  33 (11.6)  48 (16.9)  31 (10.9)  31 (10.9)  138 (48.6)  142 (50.0)  4 (1.4)  89 (31.3)  96 (33.8)  96 (33.8)  3 (1.1)  77 (27.1)  76 (26.8)  125 (44.0)  119 (41.9)  30 (10.6)  10 (3.5) | | | | | | | | |
|  | Cuba | [36] | 2-18 months (n = 980) | Female sex  Age (months)  2 – 3  4 – 5  6 – 7  9 – 8  10 -11  12  13 – 18  Geographic area  Urban  Suburban  Rural  Health area  I  II  III  IV  V  VI  VII  VIII  Type of childcare  Home  Private household  Childcare  Sibling  Sibling > 60 months  Sibling < 5 years  Exclusive breastfeeding until 6 months of age  Previous Hospitalization  Previous antibiotic use  Previous respiratory infections  Sharing a bedroom with > 2 persons  Sharing bed with parents  Exposure to cigarette smoke | | 454 (46.3)  81 (1.9)  114 (3.0)  126 (2.0)  133 (3.3)  25 (0.7)  81 (1.6)  420 (9.1)  855 (87.7)  41 (4.2)  79 (8,1)  161 (16.4)  98 (10.0)  143 (14.6)  70 (7.1)  180 (18.4)  88 (9.0)  117 (11.9)  123 (12.6)  843 (86.0)  87 (8.9)  50 (5.1)  227 (23.2)  289 (29.5)  840 (85.7)  125 (12.8)  219 (22.3)  728 (74.3)  973 (99.3)  88 (9.0)  427 (43.6) | | | | | | | | | | | | | | | | | | | | | | |
|  | Venezuela | [37] | 3-65 months  (n = 1004) | Female sex  Age (months) range  1 – 12  13 – 24  25 – 36  37 – 48  >49  Living in an urban area  Low-income families  Childcare attendance  Breastfeeding  Passive smoking  Number of siblings  0  1  > 2  Family size (> 5 persons)  Antibiotic therapy in the last 30 days  History of upper respiratory tract infections  History of lower respiratory tract infections | | 500 (49.8)  413 (41.1)  306 (30.5)  175 (17.4)  62 (6.2)  12 (1.2)  898 (89.4)  616 (61.4)  352 (35)  899 (89.5)  375 (37.4)  670 (71.5)  193 (19.2)  74 (7.4)  521 (52)  149 (15)  218 (21.7)  135 (13.4) | | | | | | | | | | | | | | | | | | | | | | |
|  |  | [38] | 0 – 62 years  (n = 1064)^d^ | Female sex  Age (years)  0 – 4  5 – 10  13 – 63  Cooking method  Wood smoke  Gas  House without walls  Tobacco smoke exposure in household  Crowding (> 5 people/no. rooms in the house) | | 698/1059 (65.9)  504 (47.4)  227 (21.3)  333 (31.3)  894/1054 (84.8)  160/1054 (15.2)  319/1058 (30.2)  491/1055 (46.5)  511/1060 (48.2) | | | | | | | | | | | | | | | | | | | | | | |
|  |  | [38] | < 5 years  (n = 504) | Female sex  Height-for-age Z score > - 2 standard deviations  Weight-for-age Z score > - 2 standard deviations | | 255 (50.6)  311 (61.7)  480 (95.2) | | | | | | | | | | | | | | | | | | | | | | |
| Eastern Mediterranean | Iran | [39] | 7 – 14 years  (n = 532) | Pneumococcal carriage status  The educational level of the mother  Illiterate  Elementary school  Secondary school  High school  The educational level of the father  Illiterate  Elementary school  Secondary school  High school  Participant sex  Male  Female  Children’s sleep mode  With parents  Without parents  Status of parent smoking  In-home  Outside  Age group total  < 10 years  > 10 years  Age group male participants  < 10 years  > 10 years  Age group female participants  < 10 years  > 10 years | | Positive  4 (8.51)  35 (13.31)  22 (12.36)  3 (6.82)  7 (15.22)  36 (14.75)  16 (9.52)  5 (6.86)  27 (13.78)  37 (11.01)  18 (11.92)  46 (12.7)  33 (18.03)  31 (8.88)  32 (12.75)  32 (11.39)  18 (19.78)  9 (8.57)  14 (8.75)  23 (13.07) | | | | | | | | | | | | | | Negative  43 (91.49)  228 (86.69)  156 (87.64)  41 (93.18)  39 (84.78)  208 (85.25)  152 (90.48)  68 (93.15)  169 (86.22)  299 (88.99)  133 (88.08)  335 (87.93)  150 (81.97)  318 (91.12)  219 (87.25)  249 (88.61)  73 (80.22)  96 (91.43)  146 (91.25)  153 (86.93) | | | | | | | | |
| Europe | Poland | [40] | 3 - 5 years  (n = 311) | Female sex  Age (years) at entry  3  4  5  Sibling  0  1  2  Sibling age (years)  < 2  3 – 5  > 6  < 2 and 3 – 5  < 2 and > 6  3 – 5 and > 6  < 2 and 3 – 5 and > 6  Passive smoking | | 158 (50.8)  90 (28.9)  115 (37.0)  106 (34.1)  135 (43.4)  139 (44.7)  37 (11.9)  25 (8.0)  21 (6.7)  115 (37.0)  3 (1.0)  3 (1.0)  8 (2.6)  1 (0.3)  118 (37.9) | | | | | | | | | | | | | | | | | | | | | | |
|  |  | [40] | 3 - 5 years  (n = 311) | Season  Antibiotic consumption in previous 2 months  β-Lactam  Macrolide  Cotrimoxazole  β-Lactam and macrolide  β-Lactam and cotrimoxazole  Macrolide and cotrimoxazole  β-Lactam and macrolide and cotrimoxazole  No data  Respiratory tract infection in previous 3 months  Otitis media  Pharyngitis  Pneumonia  Bronchitis  Sinusitis  Hospitalization | | Autumn  141 (45.3)  73 (23.5)  8 (2.6)  19 (6.1)  12 (3.8)  6 (1.9)  1 (0.3)  3 (1.0)  14 (4.5)  199 (64.0)  13 (4.2)  62 (19.9)  3 (1.0)  48 (15.4)  2 (0.6)  14 (4.5) | | | | | | | | | | Winter  132 (42.4)  76 (24.4)  13 (4.2)  9 (2.9)  13 (4.2)  4 (1.3)  1 (0.3)  0 (0.0)  12 (3.9)  190 (61.1)  15 (4.8)  40 (12.9)  4 (1.3)  27 (8.7)  2 (0.6)  6 (1.9) | | | | | | | | | Spring  105 (33.8)  61 (19.6)  15 (4.8)  8 (2.6)  4 (1.3)  3 (1.0)  0 (0.0)  0 (0.0)  8 (2.6)  137 (44.1)  8 (2.6)  56 (18.0)  1 (0.3)  13 (4.2)  3 (1.0)  7 (2.3) | | | |
|  | Turkey | [41] | 0 - 2 years  (n = 564) | Female sex  Mother’s education  Illiterate  Can read and write  Primary school graduate (5 years)  Middle school graduate (8 years)  High school graduate (11 years)  College (> 11 years)  Father’s education  Illiterate  Can read and write  Primary school graduate (5 years)  Middle school graduate (8 years)  High school graduate (11 years)  College (> 11 years)  Father’s occupation  Unemployed  Farmer  Laborer  Officer  Retired  Owns business  Mother’s occupation  Housewife  Farmer  Laborer  Officer  Retired  Owns business  Socio-economic level (score point)  Very low (0 – 20)  Low (21 – 40)  Medium (41 – 60)  High (61 - 80)  Very high (81 – 100)  Presence of a child within the house who attends a school  A child sleeps with parents or siblings  Antibiotic use in the past 30 days  Smoker in the house | | 281 (49.8)  11 (2.0)  5 (0.9)  242 (42.9)  48 (8.5)  120 (21.3)  138 (24.5)  5 (0.9)  2 (0.4)  174 (30.9)  53 (9.4)  155 (27.5)  174 (30.9)  14 (2.5)  33 (5.9)  45 (8.0)  257 (45.6)  1 (0.2)  214 (37.9)  397 (70.4)  1 (0.2)  2 (0.4)  156 (27.7)  0 (0.0)  8 (1.4)  7 (1.2)  98 (17.4)  102 (18.1)  195 (34.6)  162 (28.7)  165 (29.9)  519 (92.0)  186 (32.9)  326 (57.8) | | | | | | | | | | | | | | | | | | | | | | |
|  |  | [42] | 9 days - 67 months  (n = 301)^d^ | Female sex  Age (months)  0 – 3  > 3 – 6  > 6 – 9  > 9 – 12  > 12  Maternal education > 5 years  Paternal education > 5 years  Monthly income < 600 New Turkish Lira  Social security  1 – 3 rooms in the house  Number of family members  < 3  4  5  > 6  > 1 sibling  Respiratory tract disease in last three months (infants 0-3 months)  > 1 episode acute otitis media in last year (infants)  Hospitalization in the last year (infants 0-12 months)  Antibiotic use in the last two months (infants 0-2 months)  Antibiotic use of the family members in the last three months  Hospitalization of family members in the last three months | | 141 (46.8)  45 (14.9)  74 (24.6)  31 (10.3)  42 (14.0)  109 (36.2)  110 (36.5)  163 (54.2)  193 (64.1)  216 (71.8)  158/278 (56.8)  83 (27.6)  117 (38.9)  47 (15.6)  54 (17.9)  186 (61.8)  8/169 (56.1)  12/31 (38.7)  7/9 (77.8)  2/149 (49.5)  130 (43.2)  190 (63.1) | | | | | | | | | | | | | | | | | | | | | | |
|  |  | [43] | 1 months - 18 years (n = 1101); for most variables, only % was reported | Female sex  Age (months) median  Age group (years)  < 2  < 5  Received PCV7  Non-vaccinated  Respiratory infection within the last year  Respiratory infection within the last month  Antibiotic consumption within the last year  Antibiotic consumption within the last three months  Antibiotic consumption within the last month  Hospitalization within the last year  Hospitalization within the last three months  Hospitalization within the last month  Operation history within last year  Operation history within last six months  Attendance at childcare centers  Attendance at childcare centers (participant and siblings)  History of breastfeeding  Exposure to smoke at home  Presence of a health care provider at home  Month income level of parents  < ₺500  ₺500 - ₺1000  ₺1001 - ₺2000  ₺2001 - ₺5000  >₺5000 | | 504 (45.8)  25 (1 – 18)  (49.6)  (73.5)  679 (61.7)  422 (38.3)  (78.8)  (46.7)  (56.9)  (34.0)  (19.3)  (7.4)  (2.5)  (1.1)  (2.5)  (1.9)  (6.0)  (11.8)  (9,4)  (33.0)  (13.7)  (2.9)  (34.6)  (42.2)  (18.6)  (1.7) | | | | | | | | | | | | | | | | | | | | | | |
|  |  | [44] | 0-6 years  (n = 150) | Female sex  Age (months), mean (SD)  Breastfeeding (months), mean (SD)  Siblings, mean (range)  Number of people living at home, mean (range)  Vaccination status  PCV7 (3 doses)  PCV7 (3 + 1 doses)  PCV13 (3 doses)  PCV13 (3 + 1 doses)  Respiratory infection  Last month  Last three months  Antibiotic usage in the last month  Participant attendance at childcare centers  Sibling attendance at childcare centers  Participant & sibling attendance at childcare centers  Mother’s education < primary school  Father’s education < primary school  Exposure to smoke at home | | 74 (49.3)  23.7 (+ 1.42)  12.5 (+7.8)  1 (0 – 4)  4 (3 – 10)  2 (1.3)  14 (9.3)  51 (34.0)  83 (55.3)  35 (23.3)  41 (27.4)  34 (22.7)  6 (4.0)  43 (28.7)  7 (4.7)  32 (21.3)  36 (24.0)  55 (36.7) | | | | | | | | | | | | | | | | | | | | | | |
| Western Pacific | Fiji | [45] | 5 – 8 weeks  (n = 2006) ^d^ | Infant delivery method group  Numbers  Age (weeks, mean SD)  Female sex  Ethnicity  Fijian of Indian Descent  iTaukei  Other  Breastfeeding at the time of the survey  Upper respiratory tract infection symptoms  Antibiotic use in past two weeks  Residential location  Rural  Urban  Low family income (below basic needs poverty line)  Exposure to household cigarette smoke  > 2 children aged < 5 years in the household  Survey year  Pre-PCV10  1 year post-PCV10  2 years post-PCV10  3 years post-PCV10 | | Total  2006  6.1 (0.02)  976 (48.7)  796 (39.7)  1202 (59.9)  8 (0.4)  1857/2005 (92.6)  311/2005 (15.5)  42/2005 (2.1)  1027 (51.2)  979 (48.8)  994/1954 (50.9)  1011 (50.4)  887 (44.2)  499 (24.9)  510 (25.4)  500 (24.9)  497 (24.8) | | | | | | Vaginal  1742  6.1 (0.02)  865 (49.7)  681 (39.1)  1053 (60.4)  8 (0.5)  1609/1741 (92.4)  260 (14.9)  35 (2.0)  851 (48.9)  891 (51.1)  845/1697 (49.8)  871 (50.0)  797 (45.8)  456 (26.2)  457 (26.2)  405 (23.3)  424 (24.3) | | | | | | | | | | | | | Caesarean section  264  6.1 (0.04)  111 (42.0)  115 (43.6)  149 (56.4)  0 (0.0)  248 (93.9)  51 (19.3)  7 (2.7)  176 (66.7)  88 (33.3)  149/257 (58.0)  140 (53.0)  90 (43.1)  43 (16.3)  53 (20.1)  95 (36.0)  73 (27.7) | | | |
|  |  | [46] | 5 – 8 weeks  12 – 23 months  2 – 6 years  Caregivers of pediatric participants  (n = 2014) | Numbers  Male sex  Participant group  Infants 5 – 8 weeks  Toddlers 12 – 23 months  Young children 2 – 6 years  Caregivers of pediatric participants  Residential location  Urban  Rural  Upper respiratory tract infection symptoms  Antibiotics in past two weeks  Exposure to household cigarette smoke  Poverty  Household size (mean, 95% CI)  All ages  Aged < 18 years  Aged < 5 years  PCV10 vaccinated  Total contacts (mean, 95% CI)  Intensity of contact  Non-physical contacts (mean, 95% CI)  Physical contacts (mean, 95% CI)  Location of contact  Leisure activities  Home  General regularity  < 1 per month  1 – 2 times per month  1 – 2 times per week  Daily  Duration  5 – 14 minutes  15 – 59 minutes  1 – 4 hours  > 4 hours | | Indigenous iTaukei  1212  511 (42.2)  303 (25.0)  301 (24.8)  303 (25.0)  305 (25.2)  641 (52.9)  571 (47.1)  393 (32.4)  43 (3.6)  658 (54.3)  631 (52.1)  8.1 (7.9 – 8.3)  3.8 (3.7 – 4.0)  2.1 (2.1 – 2.2)  375 (30.9)  7.36 (7.14 – 7.59)  1.82 (1.67 – 1.97)  5.55 (5.40 – 5.69)  0.23 (0.18 – 0.29)  7.13 (6.91 – 7.35)  0.04 (0.00 – 0.07)  0.06 (0.04 – 0.09)  0.16 (0.11 – 0.20)  7.10 (6.88 – 7.32)  0.03 (0.01 – 0.04)  0.05 (0.03 – 0.08)  0.20 (0.14 – 0.26)  7.08 (6.86 – 7.29) | | | | | | | | | | | | | | Fijian of Indian Descent  802  319 (39.8)  193 (24.1)  197 (24.6)  207 (25.8)  205 (25.5)  405 (50.5)  397 (49.5)  163 (20.3)  18 (2.2)  429 (53.5)  426 (53.1)  5.7 (5.6 – 5.8)  2.3 (2.2 – 2.4)  1.4 (1.4 – 1.5)  238 (29.7)  4.94 (4.78 – 5.09)  0.83 (0.73 – 0.94)  4.10 (3.98 – 4.23)  0.19 (0.14 – 0.24)  4.74 (4.59 – 4.89)  0.05 (0.01 – 0.09)  0.06 (0.03 – 0.09  0.12 (0.08 – 0.15)  4.71 (4.57 – 4.85)  0.02 (0.01 – 0.03)  0.05 (0.02 – 0.07)  0.11 (0.07 – 0.15)  4.76 (4.61 – 4.90) | | | | | | | | |
|  |  | [47] | 5 – 8 weeks  12 – 23 months  2 – 6 years  Caregivers of pediatric participants  (n = 8109) ^d^ | PCV10 vaccinated  Survey year  Pre-PCV10 (2012)  One year post-PCV10 (2013)  Two years post-PCV10 (2014)  Three years post-PCV10 (2015)  Ethnicity  Fijian of Indian Descent  iTaukei  Other  Participant group  5 -8 weeks  12 – 23 months  2 – 6 years  Caregivers of pediatric participants  Residential location  Rural  Urban  Female sex  > 2 children aged < 5 years in the household  Low family income (below basic needs poverty line)  Upper respiratory tract infection symptoms  Exposure to household cigarette smoke  Antibiotic use in past two weeks | | 1105 (13.6)  2025 (25.0)  2042 (25.2)  2022 (24.9)  2020 (24.9)  3236 (39.9)  4835 (59.6)  38 (0.5)  2006 (24.7)  2004 (24.7)  2052 (25.3)  2047 (25.3)  3944 (48.6)  4165 (51.4)  4683 (57.8)  4004/8106 (49.4)  4599/7831 (58.7)  2092 (25.8)  4353 (53.7)  357/8105 (4.4) | | | | | | | | | | | | | | | | | | | | | | |
| **High-income countries^c^ (n = 36)** | | | | | | | | | | | | | | | | | | | | | | | | | | | | |
| Americas | Canada | [48] | 10 months - 5 years  (n = 6149; % only reported) | Female sex  Age  12 months  18 months  4.5 years  Sibling(s) < 2 years  Sibling(s) > 2 years only  < 10 hours childcare attendance per week  > 10 hours childcare attendance per week  Minor underlying health conditions  Major underlying health conditions  1 episode of otitis media in the last year  > 2 episodes otitis media in the last year  Hospitalized overnight in the last six months  Antibiotics at time of the survey  Antibiotics within two months before the survey | | (48)  (39)  (35)  (26)  (15)  (51)  (6)  (23)  (6)  (2)  (16)  (9)  (2)  (3)  (17) | | | | | | | | | | | | | | | | | | | | | | |
|  | United States of America | [49] | < 2 - ≥ 5 years (n = 737) | Community  Number in sample  Female sex  Age (years),  < 2  2 – 4  > 5  Number of children < 8 years living at home  1  2  3  > 4  Childcare exposure  Infection history, number with > 1 during previous 4 months  Otitis media  Upper respiratory tract infection  Bronchitis  Pneumonia  Other  Number antibiotic courses during previous 12 months  0  1 – 2  3 – 4  > 5  Antibiotic classes, number with > 1 course  Cephalosporin  Previous 4 months  Previous 12 months  Penicillin  Previous 4 months  Previous 12 months  Macrolide  Previous 4 months  Previous 12 months  Sulfanomide  Previous 4 months  Previous 12 months  Other  Previous 4 months  Previous 12 months | | A  368  162 (44.0)  53 (14.4)  128 (34.8)  187 (50.8)  50 (13.6)  133 (36.1)  106 (28.8)  79 (21.5)  16 (4.3)  46 (12.5)  49 (13.3)  23 (6.3)  4 (1.1)  31 (8.4)  109 (29.6)  145 (39.4)  56 (15.2)  58 (15.8)  102 (27.7)  174 (47.3)  76 (20.7)  128 (34.8)  47 (12.8)  88 (23.9)  16 (4.3)  25 (6.8)  5 (1.4)  14 (3.8) | | | | | | | | | | | | | | B  369  190 (51.5)  54 (14.6)  116 (31.4)  199 (53.9)  89 (24.1)  149 (40.4)  76 (20.6)  55 (14.9)  23 (6.2)  59 (16.0)  54 (14.6)  13 (3.5)  10 (2.7)  35 (9.5)  98 (26.6)  151 (40.9)  63 (17.1)  57 (15.4)  44 (11.9)  72 (19.5)  148 (40.1)  216 (58.5)  65 (17.6)  113 (30.6)  39 (10.6)  59 (16.0)  5 (1.4)  10 (2.7) | | | | | | | | |
|  |  | [50] | < 6 years  (n = 410) | Female sex  Vaccinee  Age (months), median (range)  Household member  Age (months), median (range)  Navajo  Had ever been breastfed  Attended childcare during the last month  Had an episode of otitis media during the last month  Received antibiotics during the last month  Currently receiving antibiotics  Hospitalized in last month  Number <6 years of age in household, median (range)  Smoker lives in the household  Wood/Coal-burning stove in household | | 214 (52.2)  269 (65.6)  8.2 (6-20)  141 (34.3)  44.9 (1-81)  330 (80)  303 (73.9)  52 (12.7)  80 (19.5)  84 (20.5)  31 (7.6)  12 (2.9)  2 (1-5)  67 (16.3)  235 (57.3) | | | | | | | | | | | | | | | | | | | | | | |
|  |  | [51] | ≤ 5 years  (n = 291) | Age (months), median  Female sex  Ethnicity  Black  Hispanic  White  Asian/Pacific Islander  Socioeconomic  < $14000  Public assistance  Women and Infant Children assistance program  Private insurance  Hospitalizations  Surgeries  Ear infections  Antibiotics use  Household member using antibiotics  Prolonged antibiotic use  Hospitalization of a household member  Childcare or nursery  Childcare > 5 days per week  Any childhood illnesses  Any care outside the house  Chronic care or correctional facility/shelter  Medication within two weeks  Medication within 2 – 4 weeks  Medication within 1 – 6 months  Medication within 6 – 12 months | | 12  149 (51.2)  177 (60.8)  100 (34.4)  13 (4.5)  1 (0.3)  190 (65.3)  215 (73.9)  196 (67.4)  54 (18.6)  120 (41.2)  83 (28.5)  5 (1.7)  120 (41.2)  66 (22.7)  5 (1.7)  58 (19.9)  38 (13.1)  60 (20.6)  58 (19.9)  89 (30.6)  9 (3.1)  6 (2.1)  33 (11.3)  48 (16.5)  34 (11.7) | | | | | | | | | | | | | | | | | | | | | | |
|  |  | [52] | < 7 years  (n = 742) ^d^ | Age (months)  0 to < 5  5 - < 24  24 - < 36  36  Female sex  Ethnicity  White  Black  Hispanic  Asian  Other  Current respiratory tract infection  Current antibiotic use  Antibiotic use within two months of survey  Vaccination (Prevnar)  0  1  > 1  Childcare participant  Number of siblings < 6 years old  0  1  > 1  Roommates < 6 years old  Smoking in home  Breastfed > 2 months  Prematurity (< 36 weeks) | | 109 (14.7)  270 (36.4)  104 (14.0)  259 (34.9)  340/741 (45.9)  548/707 (77.5)  50/707 (7.1)  53/707 (7.5)  20/707 (2.8)  36/707 (5.1)  195/729 (26.7)  70/735 (9.5)  294/729 (40.3)  398/729 (54.6)  113/729 (15.5)  218/729 (29.9)  303/69543.5)  367/701 (52.4)  266/701 (37.9)  68/701 (9.7)  150/676 (20.7)  209/700 (29.9)  274/679 (40.4)  29/729 (4.0) | | | | | | | | | | | | | | | | | | | | | | |
|  |  | [53] | < 7 years (710) ^d^ | Female sex  Age (months)  0 - < 5  5 - < 24  24 - < 36  > 36  Race  White  Black  Hispanic  Asian  Other  Current respiratory tract infection  Current antibiotic use  Antibiotic use within two months  Pneumococcal vaccination (Prevnar)  0 doses  1 dose  > dose  Childcare participant  Siblings aged < 6 years  0  1  > 1  Roommates aged < 6 years  Smoking in home  Breastfed for > 2 months  Prematurity (< 36 weeks) | | 324 (45.6)  105 (14.8)  258 (36.3)  99 (13.9)  248 (34.9)  525/673 (78.0)  49/673 (7.3)  52/673 (7.7)  18/673 (2.7)  29/673 (4.3)  191 (26.9)  69 (9.7)  285 (40.1)  389 (54.8)  108 (15.2)  213 (30.0)  294/672 (43.8)  353/674 (52.4)  254/674 (37.7)  67/674 (9.9)  134/650 (20.6)  201/674 (29.8)  261/652 (40.0)  29/729 (4.0) | | | | | | | | | | | | | | | | | | | | | | |
|  |  | [54] | 3 months - <7 years (n = 2638) | Year  Number included in sample  Age (months)  3 - < 6  6 - < 24  24 - < 36  36 - < 84  Antibiotic use within 2 months  Childcare attendance  Respiratory tract infection at the time of specimen collection  Number of young siblings  0  1  > 1 | | 2001  678  59 (8.7)  256 (37.8)  104 (15.3)  259 (38.2)  287 (42.3)  301 (44.4)  192 (28.3)  339 (50.0)  239 (35.3)  62 (9.1) | | | | | | | 2004  988  49 (5.0)  349 (35.3)  143 (14.5)  447 (45.2)  288 (29.1)  480 (48.6)  263 (26.6)  722 (73.1)  205 (20.7)  35 (3.5) | | | | | | | | | | | | 2007  972  76 (7.8)  428 (44.0)  130 (13.4)  338 (34.8)  390 (40.1)  458 (47.1)  448 (46.1)  500 (51.4)  345 (35.5)  85 (8.7) | | | |
|  |  | [55] | 3-59 months (n = 1275 children; n = 1350 swabs) | Year  Male sex  Age (years)  < 1  1 - < 2  > 2  Alaskan Native  Hispanic  Household tobacco use  Household contact aged < 5 years  Attended a childcare center  Childcare exposure through a household contact  Up to date for age, for PCV7 vaccination  Received > one dose of PCV7  Received one dose of PCV7  Antibiotic use within 90 days of nasopharyngeal swab  β-Lactam use within 90 days of nasopharyngeal swab  Cotrimoxazole use within 90 days of nasopharyngeal swab | | 2000  213 (47)  107 (24)  127 (28)  216 (48)  146 (32)  88 (20)  170 (38)  134 (30)  158 (35)  15 (3)  0 (0.0)  0 (0.0)  0 (0.0)  152 (38)  128 (32)  26 (6) | | | | | | | 2001  219 (49)  94 (21)  127 (28)  249 (51)  139 (31)  95 (21)  163 (38)  167 (37)  149 (34)  15 (3)  78 (17)  156 (35)  117 (26)  155 (39)  123 (31)  20 (5) | | | | | | | | | | | | 2002  230 (51)  89 (20)  126 (28)  235 (52)  151 (34)  99 (22)  148 (34)  155 (34)  152 (35)  15 (3)  247 (55)  294 (65)  68 (40)  164 (40)  139 (34)  16 (4) | | | |
|  |  | [56] | 3-59 months  (n = 2061) | Year  Female sex  Age group (months)  3 – 11  12 – 23  24 – 59  Alaskan Native  Hispanic  Household contact aged < 5 years  Childcare exposure (participant or sibling)  Household tobacco use  Up to date for age, for PCV7 vaccination  Received > one dose of PCV7  Received one dose of PCV7  Recent Hospitalization  Antibiotic use within 90 days of survey  β-Lactam use within 90 days of survey  Trimethoprim-sulfamethoxazole use within 90 days of survey  Macrolide use within 90 days of nasopharyngeal swab | | 2000  235 (52)  107 (24)  127 (28)  216 (48)  147 (33)  88 (20)  140 (31)  174 (39)  170 (38)  0 (0)  0 (0)  0 (0)  14 (3)  159 (36)  132 (30)  26 (6)  32 (7) | | | | 2001  231 (51)  94 (21)  127 (28)  229 (51)  140 (31)  95 (21)  174 (39)  164 (37)  163 (38)  78 (17)  156 (35)  117 (26)  13 (3)  160 (36)  127 (28)  20 (4)  41 (9) | | | | | | | 2002  219 (49)  89 (20)  126 (28)  235 (52)  152 (34)  98 (22)  165 (37)  168 (39)  148 (34)  247 (55)  294 (65)  68 (15)  18 (4)  169 (38)  141 (32)  16 (4)  30 (7) | | | | | 2003  218 (48)  85 (19)  111 (25)  254 (56)  164 (36)  87 (19)  174 (39)  165 (38)  150 (36)  201 (45)  341 (76)  64 (14)  12 (3)  164 (37)  146 (33)  11 (2)  22 (5) | | | | | 2004  224 (5)  121 (27)  110 (24)  219 (49)  152 (34)  86 (20)  176 (39)  149 (34)  159 (35)  283 (63)  400 (89)  54 (12)  21 (5)  133 (30)  120 (27)  9 (2)  11 (2) | |
|  |  | [57] | 6 - 7 years  (n = 5380; % only reported) | Year  Number in sample  Female sex  Age group  < 6 months  6 - < 12 months  12 - < 24 months  2 - < 5 years  5 - < 7 years  Siblings < 6 years in household  0  1  > 2  Group childcare attendance  None or < 4 hours / week  4 – 10 hours / week  11 – 20 hours / week  > 20 hours / week  Recent antibiotic use  < 2 weeks  2 - < 4 weeks  4 - < 6 weeks  6 - < 8 weeks  > 8 weeks or none recorded  Respiratory tract infection at the time of specimen collection  Cigarette smoke exposure in household  Breastfed for < 3 months  Breastfed for 3 – 6 months  Breastfed for > 6 months  Ethnicity  White non-Hispanic  Black non-Hispanic  Hispanic / Latino  Asian / Pacific Islander  Other | | 2001  678  (45.79)  (8.70)  (16.52)  (21.24)  (36.14)  (17.40)  (52.97)  (37.34)  (9.69)  (55.52)  (14.83)  (5.77)  (23.89)  (10.18)  (8.26)  (7.37)  (6.19)  (67.99)  (28.83)  (29.73)  (18.48)  (24.01)  (18.96)  (77.83)  (6.67)  (7.60)  (3.10)  (4.81) | | | | 2004  987  (52.79)  (4.96)  (12.16)  (23.10)  (41.84)  (17.93)  (50.47)  (39.02)  (10.51)  (48.59)  (11.53)  (13.88)  (26.00)  (10.33)  (8.81)  (5.57)  (4.15)  (71.12)  (27.14)  (20.57)  (16.89)  (21.46)  (26.71)  (84.07)  (3.86)  (5.50)  (1.99)  (4.57) | | | | | | | 2007  1540  (48.12)  (9.35)  (17.27)  (25.65)  (34.81)  (12.92)  (58.06)  (34.05)  (7.89)  (50.17)  (8.44)  (8.37)  (33.02)  (4.61)  (6.88)  (4.55)  (5.45)  (78.51)  (46.04)  (19.83)  (19.21)  (23.83)  (29.59)  (53.19)  (27.98)  (12.44)  (2.22)  (4.17) | | | | | 2009  1011  (48.17)  (7.72)  (17.31)  (23.44)  (37.88)  (13.65)  (53.75)  (36.84)  (9.41)  (43.44)  (12.28)  (11.75)  (32.53)  (7.72)  (6.92)  (3.36)  (3.17)  (78.83)  (39.27)  (25.74)  (20.27)  (22.11)  (28.25)  (82.27)  (5.15)  (7.42)  (4.02)  (1.13) | | | | | 2011  1164  (46.48)  (7.30)  (18.13)  (23.20)  (35.14)  (16.24)  (53.41)  (36.73)  (9.85)  (45.56)  (11.61)  (10.11)  (32.72)  (5.41)  (6.62)  (3.44)  (2.84)  (81.70)  (33.16)  (22.17)  (20.33)  (26.00)  (29.76)  (69.41)  (12.01)  (10.60)  (5.00)  (2.98) | |
|  |  | [58] | < 7 years (n = 1337) | Location  Number in sample  Female sex  Age (months)  0 - < 6  6 - < 24  24 - < 36  > 36  Ethnicity  Black non-Hispanic  Hispanic  Other non-Hispanic  White non-Hispanic  Current respiratory tract infection  Current antibiotic use  Antibiotic use within two months of survey  PCV7 vaccination doses  0  1  > 1  Childcare participant  Number of siblings aged < 6 years  0  1  > 1  Smoking in home  Breastfed for > 2 months  Education  Less than high school graduate  High school graduate  Some college  College graduate or beyond  Household income (US$)  < 35,000  35,000 – 49,000  50,000 – 74,000  75,000 – 99,000  > 100,000 | | Urban Boston  543  275 (50.6)  65 (12.0)  223 (41.1)  86 (15.8)  169 (31.1)  347 (63.9)  111 (20.4)  38 (7.0)  47 (8.7)  252 (46.4)  8 (1.5)  79 (14.6)  42 (7.7)  53 (9.8)  448 (82.5)  274 (50.5)  353 (65.0)  158 (29.1)  32 (5.9)  108 (20.1)  307 (56.5)  78 (14.4)  219 (40.3)  140 (25.8)  106 (19.5)  408 (75.1)  85 (15.7)  30 (5.5)  11 (2.0)  9 (1.7) | | | | | | | | | | | | | | Outside Boston  794  364 (45.8)  57 (7.2)  348 (43.8)  105 (13.2)  284 (35.8)  43 (5.4)  54 (6.8)  34 (4.3)  663 (83.5)  527 (66.4)  47 (5.9)  298 (37.5)  11 (1.4)  53 (6.7)  729 (91.9)  409 (51.1)  420 (52.9)  297 (37.4)  77 (9.7)  145 (19.6)  410 (52.0)  26 (3.3)  111 (14.0)  195 (24.6)  462 (58.2)  125 (15.7)  100 (12.6)  150 (18.9)  137 (17.3)  282 (35.5) | | | | | | | | |
|  |  | [59] | < 10 years  (n = 12535 swabs) | Female sex  Alaska Native  Age  < 6 months  6 – 11 months  12 – 23 months  2 – 4 years  5 – 9 years  10 – 14 years  15 – 17 years  18 – 49 years  50 – 64 years  > 65 years  No in-home running water  Mean number of people per number of rooms in the house  Percent of children aged 3 – 5 months up to date with PCV  Region  Bristol Bay  Yukon Delta  Norton Sound  Respiratory infection in previous 90 days  Skin infection in previous 90 days  Urinary tract infection in previous 90 days  Recent systemic antibiotic use | | 6133 (49)  12,421 (99.3)  126 (1.0)  187 (1.5)  358 (2.9)  1032 (8.2)  1714 (13.7)  1721 (13.7)  942 (7.5)  4839 (38.6)  1136 (9.1)  480 (3.8)  6023 (48)  1.5  1389 (83.9)  1649 (13.2)  7724 (61.7)  3162 (25.2)  4454 (35.5)  438 (3.5)  249 (2.0)  3188 (25.4) | | | | | | | | | | | | | | | | | | | | | | |
|  |  | [60] | 3 months - < 7 years  (n = 1982) | Survey years  Number in sample  Female sex  Age  3 to <6 months  6 to <24 months  24 to <36 months  36 to <84 months  Group childcare attendance > 4 hours per week  Young siblings in household  0  1  > 1  Respiratory tract infection at the time of specimen collection  Recent antibiotic use | | 2006 – 7  971  455 (47)  76 (8)  427 (44)  130 (13)  338 (35)  458 (50)  500 (54)  344 (37)  85 (9)  447 (46)  390 (40) | | | | | | | | | | | | | | 2008 – 9  1011  487 (48)  78 (8)  412 (41)  150 (15)  371 (37)  539 (57)  537 (54)  368 (37)  94 (9)  397 (39)  294 (29) | | | | | | | | |
| Eastern Mediterranean | Cyprus | [61] | 6 months - 5 years  (n = 402) | Female sex  Age, years  < 1  1 – 2  > 2  Nationality  Both parents Cypriots  Others  Missing  Breastfeeding  Exposure to smoking  Childcare attendance  1 sibling  > 2 siblings  Vaccinated  No  Incomplete  Full  Missing  Origin of specimen  Public center  Private center | | 195 (48.5)  130 (32.3)  123 (30.6)  149 (37.1)  273 (67.9)  128 (31.9)  1 (0.2)  300 (74.6)  142 (35.3)  170 (42.3)  140 (35.4)  87 (22.0)  226 (56.2)  53 (13.2)  91 (22.6)  32 (16.0)  241 (60.3)  159 (39.8) | | | | | | | | | | | | | | | | | | | | | | |
|  | Kingdom of Saudi Arabia | [62] | > 18 years  (n = 3203) ^d^ | Period  Number in sample  Female sex  Primary or lower formal education  Age > 50 years  Resident in Africa  Current tobacco cigarette smoking  Previous influenza or pneumococcal vaccination  Chronic respiratory disease  Upper respiratory infection during Hajj  Shared room with > 1 person with an upper respiratory infection  Used antibiotics during Hajj | | Beginning Hajj  1590  662/1568 (42.2)  850/1518 (56.0)  1006/1587 (63.4)  804 (50.6)  205/1568 (13.1)  676/1475 (45.8)  90 (5.7)  -  -  - | | | | | | | | | | | | | | End Hajj  1613  424/1559 (27.2)  759/1471 (51.6)  961/1604 (59.9)  805/1613 (49.9)  344/1491 (23.1)  1106/1442 (76.7)  148/1613 (9.2)  412/1409 (29.2)  750/1613 (46.5)  379/1424 (26.6) | | | | | | | | |
| Europe | France | [63] | 6 - 24 months  (n = 3507) ^d^ | Female sex  Age (months), mean (SD)  PCV7 vaccination  Partial  Complete  Type of care  Childcare center  Childminder  Home  Siblings  Use of antibiotics three months before  Nasopharyngeal carriage of *H. influenzae*  Nasopharyngeal carriage of *M. catarrhalis*  Nasopharyngeal carriage of *S. aureus*  Multiple carriage (at least two species)  No bacteria identified | | 1689 (48.2)  13.6 (+ 5.2)  3448/3506 (98.3)  754/3506 (21.9)  2693/3506 (78.1)  1170 (33.4)  1127 (32.1)  1209 (34.5)  1906 (54.3)  1439 (41.0)  1399 (39.9)  1780 (50.8)  235 (6.7)  1715 (48.9)  563 (16.1) | | | | | | | | | | | | | | | | | | | | | | |
|  | France and the Kingdom of Saudi Arabia | [64] | 26 – 83 years  (n = 121) | Participant sex  Male  Female  Age (years)  Median (IQR)  Min-max  Age > 60 years  Place of birth  France  North Africa  Sub-Saharan Africa  Comorbidities  Diabetes mellitus  Hypertension  Chronic respiratory disease  Chronic heart disease  Chronic kidney disease  Immunodeficiency  Indication for vaccination against IPD  Smoking status  Yes, current  Yes, stopped  Never  BMI  18.5 – 24.9  25.0 – 29.9  > 30.0 | | 52 (43.0)  69 (57.0)  61 (56 – 66)  26 – 83  71 (58.7)  8 (6.6)  81 (66.9)  32 (26.5)  31 (25.6)  31 (25.6)  16 (13.2)  13 (10.7)  3 (2.5)  4 (3.3)  88 (72.7)  6 (5.0)  24 (19.8)  91 (75.2)  31 (25.6)  56 (46.3)  34 (28.1) | | | | | | | | | | | | | | | | | | | | | | |
|  | Greenland | [65] | 0 - 6 years  (n = 352)^d^ | Female sex  Age (years)  0 - < 2  2 - < 4  4 - < 7  Ethnicity  Inuit  Mixed  Other  PCV13 vaccinated  Geographical regions in Greenland  East Coast  West Coast  Childcare attendance  Current breastfeeding  Ever breastfed  Having siblings attend childcare  > 2 people per room in the household  Exposure to tobacco smoke from one or both parents | | 169/350 (48.3)  134 (38.1)  93 (26.4)  125 (35.5)  324 (92.0)  11 (3.1)  17 (4.8)  222 (63.1)  122 (34.7)  230 (65.3)  84 (23.9)  210 (59.7)  98 (27.8)  201 (71.3)  79 (22.4)  269 (76.4) | | | | | | | | | | | | | | | | | | | | | | |
|  | Italy | [66] | 0 - 59 months  (n = 669)^d^ | Female sex  Age (months)  0 – 12  13 – 24  25 – 59  Caucasian  1 sibling  > 2 siblings  Sleeping with siblings  Childcare attendance  > 1 smoker in the household  Respiratory infections  Antimicrobial drug use in the last three months  PCV7 vaccination coverage  4^th^ and 5^th^ month of life, > 1 dose  6^th^ and 7^th^ month of life, > 2 doses  > 13^th^ month, > 3 doses | | 353 (52.8)  236 (35.3)  183 (27.4)  250 (37.4)  599 (89.6)  264 (39.4)  30 (4.5)  147/294 (50)  295 (44.1)  172 (25.7)  242 (36.2)  78 (11.7)  36/36 (100)  123/125 (98.4)  405/416 (97.4) | | | | | | | | | | | | | | | | | | | | | | |
|  |  | [67] | < 5 years  (n = 571) ^d^ | Age group (months)  0 – 5  6 – 23  24 – 35  36 – 71  Childcare attendance  > 1 young sibling  Smoking in household  Vaccination status (*S. pneumoniae*)  Unvaccinated  Partially vaccinated  PCV7  PCV7/13 + PCV13 | | 28 (4.9)  132 (23.1)  124 (21.7)  287 (50.3)  451 (79.0)  176/568 (30.8)  212/568 (37.1)  100/531 (17.5)  33/531 (5.8)  228/531 (39.9)  170/531 (29.8) | | | | | | | | | | | | | | | | | | | | | | |
|  |  | [68] | < 6 years  (n = 301)^d^ | Age group (months)  0 – 5  6 – 11  12 – 23  24 – 35  36 – 71  Childcare attendance  > 1 young sibling  Parent’s smoking  Previous respiratory infections  Use of antibiotics  Vaccination status (*S. pneumoniae*)  Unvaccinated / partially vaccinated  Vaccinated (any PCV)  Vaccinated (PCV7)  Vaccinated (PCV7/PCV13 + PCV13)  Vaccination status (*H. influenzae*)  Unvaccinated  Vaccinated | | 28 (9.3)  31 (10.3)  52 (17.3)  56 (18.6)  134 (44.5)  232 (77.1)  104/299 (34.6)  116/300 (38.5)  75/280 (24.9)  78/285 (25.9)  127 (42.2)  174 (57.8)  91/174 (30.2)  83/174 (27.6)  11/298 (3.6)  287/298 (95.4) | | | | | | | | | | | | | | | | | | | | | | |
|  |  | [69] | 3-59 months (n = 1250) | Female sex  Age (months)  3 – 12  13 – 24  25 – 59  Ethnic group  Non-Caucasian  Caucasian  Siblings  0  1  > 2  Childcare attendance  Indirect smoke  Respiratory tract infection in 90 days preceding the survey  Antibiotics in the previous 90 days  Antibiotics in the previous seven days  PCV uptake by age  None  PCV7-incompletely vaccinated for age  PCV7-appropriately vaccinated for age  PCV13-incompletely vaccinated for age  PCV13-appropriately vaccinated for age  At least two doses in a combination of PCV7 and PCV13 | | 577 (46.2)  561 (44.9)  316 (25.3)  373 (29.8)  169 (13.5)  1081 (86.5)  614 (49.1)  472 (37.8)  164 (13.1)  465 (37.2)  468 (37.4)  486 (38.9)  81 (6.5)  81 (6.5)  246 (19.7)  28 (2.2)  264 (21.1)  447 (35.8)  171 (13.7)  94 (7.5) | | | | | | | | | | | | | | | | | | | | | | |
|  | Portugal | [70] | > 60 years  (n = 3361)^d^ | Female sex  Urban residence  Rural residence  Years of school education  0  1 – 4  > 5  Retirees  Retired  Active  Housing  Family home  Retirement home  Has weekly contact with children < 6 years  Recreational activities  At least one activity  Club  Day center  Senior university  Other  Smoker  Chronic disease  None  COPD  Asthma  Hepatic disease  Renal disease  Diabetes  Hypertension  Heart disease  Hospitalization in the previous year  Respiratory infection in the previous year  None  Asthma/bronchitis  Cold / flu  Rhinosinusitis  Tonsillitis  Pneumonia  Mild symptoms of respiratory disease at sampling  None  Sputum  Cough  Shortness of breath  Sore throat  Runny nose  Fever  Vaccination  Seasonal influenza  23-valent pneumococcal polysaccharide  Antibiotic consumption at sampling  Antibiotic consumption month before sampling  Antibiotic consumption in previous six months | | 1935 (57.6)  1945 (57.9)  1416 (42.1)  315/3360 (9.4)  2799/3360 (83.3)  246/3360 (7.3)  3015 (89.7)  346 (10.3)  3062 (91.1)  299 (8.9)  650 (19.3)  1119 (33.3)  339 (10.1)  652 (19.4)  51 (1.5)  99 (2.9)  126 (3.7)  585 (17.4)  497 (14.8)  218 (6.5)  70 (2.1)  68 (2.0)  951 (28.3)  2058 (61.2)  1082 (32.2)  510 (15.2)  1837 (54.7)  200 (6.0)  997 (29.7)  367 (11.0)  359 (10.7)  64 (1.9)  2062 (61.4)  330 (9.8)  330 (9.8)  278 (8.3)  491 (14.6)  425 (12.6)  19 (0.6)  1969 (58.6)  122 (3.6)  62 (1.8)  191 (5.7)  257 (7.6) | | | | | | | | | | | | | | | | | | | | | | |
|  | Spain | [71] | 10 – 14 months (1 year) and 3.5 – 4.5 years (4 years)  (n = 1821) | Year  Number of children  Age  1 year  4 years  Participant sex  Male  Female  Siblings > 2  School attendance  Childcare attendance among those 1 year old  Antibiotics during last month  1 year  4 years  Breastfeeding > 6 months  Smoke exposure  PCV13 vaccinated > 2 doses | | 2014  812 (44.5)  401 (49.4)  411 (50.6)  350 (43.1)  462 (56.9)  122 (15.0)  484 (59.6)  73 (9.0)  55 (6.8)  26 (3.2)  29 (3.6)  324 (39.9)  323 (39.7)  585 (72.0) | | | | | | | | | 2015  1009 (55.4)  505 (50.0)  504 (50.0)  543 (53.8)  466 (46.2)  131 (13.0)  599 (59.4)  95 (9.4)  114 (11.3)  56 (5.6)  58 (5.7)  333 (33.0)  393 (38.9)  650 (64.4) | | | | | | | | | Total  1821 (100.0)  906 (49.7)  915 (50.3)  893 (49.0)  928 (51.0)  253 (13.9)  1079 (59.2)  168 (9.2)  169 (10.4)  82 (4.5)  87 (4.8)  657 (36.0)  716 (39.3)  1235 (67.8) | | | | |
|  | The Netherlands | [72] | 1 - 19 years  (n = 3198) | Female sex  Family size, mean  Number of children per family, mean  Smoker in the household (% only reported)  Smokes (children aged > 12 years)  Participates in sport (children > 4 years)  Attends youth society / sports club (children > 4 years)  Attends discos > 3 hours per week (children > 10 years)  Childcare for > 3 days per week (children < 4 years) | | 1528 (48)  4.3  2.4  (45)  275 (21)  1317 (42)  909 (29)  311 (21)  262 (56) | | | | | | | | | | | | | | | | | | | | | | |
|  |  | [73] | 1.5 - 14 months (n = 1079) | Female sex  Birth weight, grams, mean (SD)  Gestational age, weeks, median (range)  Parity > 0  Maternal smoking  Maternal education level  Primary school  Secondary school  Higher education  Siblings > 1  Childcare attendance  At age 6 months  At age 12 months  Breast-feeding  Never  < 3 months  3 – 6 months  > 6 months | | 521 (48.3)  3509 (538)  40.3 (27.6 – 43.4)  420 (38.0)  85 (12.7)  21 (2.0)  361 (33.9)  683 (64.1)  256 (36.7)  611 (63.7)  666 (69.0)  98 (12.7)  196 (25.4)  172 (22.3)  306 (39.6) | | | | | | | | | | | | | | | | | | | | | | |
|  |  | [74, 75] | 6 - 24 months  (n = 336) | Male sex  Gestational age, mean (SD)  Premature birth (gestational age < 37 weeks)  Home delivery  Birth weight, grams, mean (SD)  Feeding from birth  Partial breastfed at 6 weeks  Partial breastfeed > 3 months  Partial breastfed > 6 months  Number of siblings, median (IQR)  Childcare attendance  At 12 months  At 24 months  Use of antibiotics during the month before the swab  At 12 months  At 24 months  Passive tobacco smoke exposure indoors  At 12 months  At 24 months | | 160 (48)  38.7 (1.9)  21 (6)  94 (28)  3492 (532)  256 (77)  97 (30)  59 (18)  0 (0 – 1)  192 (60)  221 (68)  20 (6)  10 (3)  21 (7)  26 (8) | | | | | | | | | | | | | | | | | | | | | | |
| Europe and Eastern Mediterranean | Israel and West Bank and Gaza^e^ | [76] | < 5 years  (n = 2750) | East Jerusalem cohort  Year  Number in sample  Male sex  Age, months  < 6  6 – 23  24 – 60  Number of household members  < 4  4 – 6  > 6  Diagnosis of respiratory infection on screen day  Childcare attendance  Antibiotics in last three months  % vaccinated > one dose  % vaccinated > two doses  % of children < 2 years vaccinated > one dose | | 2009  345  208 (59.8)  47 (13.5)  122 (35.1)  179 (51.4)  75 (21.6)  207 (59.5)  66 (19.0)  93 (26.7)  84 (24.2)  187 (53.7)  9 (2.6)  5 (1.4)  6 (3.7) | | | | | | | | 2010  311  173 (55.6)  59 (19.0)  128 (41.1)  124 (39.9)  84 (27.0)  173 (55.6)  54 (17.4)  40 (12.9)  39 (12.5)  153 (49.2)  173 (55.6)  124 (39.9)  137 (74.9) | | | | | | | | | | 2011  324  176 (54.7)  42 (13.0)  154 (47.5)  128 (39.5)  56 (17.3)  192 (59.4)  75 (23.2)  35 (10.8)  33 (10.2)  197 (60.8)  228 (70.4)  196 (60.5)  168 (85.7) | | | | |
|  |  |  |  | Palestinian Authority cohort  Year  Number in sample  Male sex  Age, months  < 6  6 – 23  24 – 60  Number of household members  < 4  4 – 6  > 6  Diagnosis of respiratory infection on screen day  Childcare attendance  Antibiotics in last three months  % vaccinated > 1 dose  % vaccinated > 2 doses  % of children < 2 years vaccinated > 1 dose | | 2009  620  378 (60.9)  140 (22.5)  294 (47.4)  186 (29.9)  143 (23.0)  326 (52.5)  152 (24.5)  339 (54.60 66 (10.7)  348 (56.0)  9 (1.4)  4 (0.6)  9 (2.5) | | | | | | | | 2010  595  376 (64.0)  195 (32.9)  249 (42.1)  148 (25.0)  185 (31.4)  308 (52.5)  97 (16.4)  307 (51.6)  34 (5.8)  263 (44.2)  35 (5.9)  13 (2.2)  15 (4.1) | | | | | | | | | | 2011  555  346 (62.6)  175 (31.5)  243 (43.6)  137 (24.6)  187 (33.7)  261 (46.9)  107 (19.2)  316 (56.7)  27 (4.9)  269 (48.3)  62 (12.9)  36 (6.5)  53 (13.1) | | | | |
| Western Pacific | Australia | [77] | > 2 years to < 16 (n = 350) | Female sex  Runny nose in the previous week  Number of household occupants < 5 years  0  1 – 2  > 3  Number of bedroom occupants < 5 years  0  1 – 2  > 3  Day time location  School  Creche  Home  With carer  Close person contact  School  Creche  Mother  *H. influenzae* detected  *M. catarrhalis* detected | | 170 (48.6)  187 (53.4)  103 (29.4)  191 (54.6)  49 (14.0)  224 (64.0)  40 (11.4)  86 (24.6)  263 (75.1)  14 (4.0)  31 (8.9)  36 (10.3)  268 (76.6)  22 (6.3)  50 (14.3)  201 (57.4)  258 (73.7) | | | | | | | | | | | | | | | | | | | | | | |
|  |  |  | > 16 years (n = 538) | Female sex  Chest infection in the previous week  Runny nose in the previous week  Number of household occupants < 5 years  0  1 – 2  > 3  Sits at an outside fire  Never / monthly  Most days  Every day  Close person contact  Adult  Schoolchild  Creche child  Young child  *H. influenzae* detected  *M. catarrhalis* detected | | 324 (60.2)  200 (37.2)  214 (39.8)  306 (56.9)  187 (34.8)  39 (7.2)  172 (32.0)  347 (64.5)  11 (2.0)  403 (74.9)  25 (4.6)  14 (2.6)  88 (16.4)  123 (22.9)  92 (17.1) | | | | | | | | | | | | | | | | | | | | | | |
|  | Hong Kong | [78] | < 5 years (n = 921) | Group  Number in sample  Male sex  > 5 household members  > 2 siblings  Smoking family member  Age (months)  < 3  3 - < 24  > 24  Living area (m^2^ / person) | | Chinese  621  324 (52.2)  125 (20.1)  27 (4.3)  245 (39.5)  183 (29.5)  281 (45.2)  157 (25.3)  10.20 | | | | | | | | | | | | | Vietnamese  300  145 (48.3)  21 (7.0)  17 (5.7)  247 (82.3)  30 (10.0)  146 (48.7)  124 (41.3)  1.71 | | | | | | | | | |
|  |  | [79] | 2, 12, and 18 months  (n = 1541) | Female sex  Age group (months)  2 months  12 months  18 months  Vaginal delivery at birth  Cesarean delivery at birth  Ever breastfed  Group care attendance  Siblings < 6 years old  Household tobacco exposure  Overcrowding  Respiratory symptoms in recent three days  Respiratory symptoms in recent one month  Doctor visit in recent three months  Hospitalization in recent three months  Antibiotic use in recent three months  Respiratory symptoms in household members in past three days  Respiratory symptoms in household members in the past one month  Antibiotic use by household members in past three months  Household health care worker  Household income > HK$ 20000  Household dog or cat | | 759 (49.3)  477 (31.0)  522 (33.9)  542 (35.2)  1030 (66.8)  511 (33.2)  1220 (79.2)  197 (12.8)  498 (32.3)  485 (31.5)  96 (6.2)  276 (17.9)  515 (33.4)  950 (61.6)  94 (6.1)  298 (19.3)  360 (23.4)  806 (52.3)  352 (22.8)  91 (5.9)  1094 (71.0)  172 (11.2) | | | | | | | | | | | | | | | | | | | | | | |
|  | Japan | [80] | 0 - 36 months  (n = 349) | Male sex  Gestational age (weeks)  < 37  > 37  Birth weight (grams)  < 2500  > 2500  Apgar score at 5 minutes  < 8  > 8  Older siblings and their childcare attendance  No siblings  Siblings not attending childcare  Siblings attending childcare  Missing  Living with an elderly person  No  Yes  Breastfeeding at 6 months  Exclusively  Partial  Bottle milk  Missing  Start of childcare attendance (months)  > 24  12 – 23  < 12  Missing  Clinic visits  < 20  > 21  Antimicrobial prescription  < 2  > 3  Subject’s regular hospital/clinic  Sado General Hospital  Ryotsu Hospital  Others  Teething ring  No  Yes  Missing  Allergy  No  Yes  Missing | | 189 (54.2)  14 (4.0)  335 (96.0)  21 (6.0)  328 (94.0)  3 (0.9)  346 (99.1)  150 (43.0)  56 (16.0)  132 (37.8)  11 (3.2)  188 (53.9)  161 (46.1)  76 (21.8)  145 (41.5)  84 (24.1)  5 (1.4)  121 (34.7)  139 (39.8)  84 (24.1)  5 (1.4)  127 (36.4)  222 (63.6)  181 (51.9)  168 (48.1)  108 (30.9)  79 (22.6)  162 (46.4)  212 (60.7)  120 (34.4)  17 (4.9)  223 (63.9)  108 (30.9)  18 (5.2) | | | | | | | | | | | | | | | | | | | | | | |
|  |  | [81] | 2 months - 6 years  (n = 229) | Female sex  Age (years)  < 1  1 – 2  3 - 6  Childcare attendance  Presence of older siblings  Presence of younger siblings  Other vaccination histories (DPT; Hib; influenza)  Antibiotics in the previous three months  Season  Winter (January – March)  Spring (April – June)  Summer (July – September)  Autumn (October – December) | | 125 (54.6)  97 (42.4)  75 (32.8)  57 (24.9)  53 (23.1)  105 (45.9)  37 (16.2)  195 (85.2)  82 (35.8)  75 (32.8)  30 (13.1)  75 (32.8)  49 (21.4) | | | | | | | | | | | | | | | | | | | | | | |
|  |  | [82] | 2 – 24 months  (n = 756) | Inoculation times of PCVs  Number of participants  Participant sex  Male  Female  Age (month)  Mean + standard deviation  Median  (IQR)  Age distribution  2 – 6 months  6 – 10 months  10 – 14 months  14 – 18 months  18 – 22 months  > 22 months  Childcare attendance  No  Yes  Number of siblings  0  1  2  3  > 4  Survey period  March-May (spring)  June – August (summer)  September – November (autumn)  December – February (winter)  Number of people at pediatric clinics  A  N  S  W  Vaccine types and inoculation times  None  PCV13 once  PCV13 twice  PCV13 three times  PCV13 four times  PCV7 once and PCV13 twice  PCV7 once and PCV13 three times  PCV7 twice and PCV13 once  PCV7 twice and PCV13 twice  PCV7 three times and PCV13 once | | None  78  41 (52.6)  37 (47.4)  3.0 + 2.7  2.0  (2.0–3.0)  73 (93.6)  4 (5.1)  0 (0.0)  0 (0.0)  0 (0.0)  1 (1.3)  74 (94.9)  4 (5.1)  26 (33.3)  24 (30.8)  18 (23.1)  9 (11.5)  1 (1.3)  18 (23.1)  22 (28.2)  19 (24.4)  19 (24.4)  18 (23.1)  13 (16.7)  5 (6.4)  42 (5.38)  78 (100.0)  0 (0.0)  0 (0.0)  0 (0.0)  0 (0.0)  0 (0.0)  0 (0.0)  0 (0.0)  0 (0.0)  0 (0.0) | 1  99  59 (59.6)  40 (40.4)  4.6 + 3.6  3.0  (3.0–4.0)  81 (81.8)  11 (11.1)  3 (3.0)  2 (2.0)  1 (1.0)  1 (1.0)  92 (92.9)  7 (7.1)  34 (34.3)  33 (33.3)  18 (18.2)  11 (11.1)  3 (3.0)  24 (24.2)  22 (22.2)  26 (26.3)  27 (27.3)  20 (20.2)  30 (30.3)  21 (21.2)  28 (28.3)  0 (0.0)  99 (100.0)  0 (0.0)  0 (0.0)  0 (0.0)  0 (0.0)  0 (0.0)  0 (0.0)  0 (0.0)  0 (0.0) | | | | 2  138  88 (63.8)  50 (36.2)  5.2 + 2.6  4.0  (4.0–5.0)  108 (78.3)  20 (14.5)  7 (5.1)  2 (1.4)  1 (0.7)  0 (0.0)  130 (94.2)  8 (5.8)  59 (42.8)  54 (39.1)  13 (9.4)  9 (6.5)  3 (2.2)  30 (21.7)  38 (27.5)  23 (16.7)  47 (34.1)  58 (42.0)  24 (17.4)  30 (21.7)  26 (18.8)  0 (0.0)  0 (0.0)  138 (100.0)  0 (0.0)  0 (0.0)  0 (0.0)  0 (0.0)  0 (0.0)  0 (0.0)  0 (0.0) | | | | | | | 3  302  152 (50.3)  150 (49.7)  11.5 + 4.8  12.0  (7.0–14.0)  34 (11.3)  86 (28.5)  96 (31.8)  48 (15.9)  29 (9.6)  9 (3.0)  203 (67.2)  99 (32.8)  137 (45.4)  94 (31.1)  47 (15.6)  19 (6.3)  5 (1.7)  69 (32.1)  86 (28.5)  61 (20.2)  86 (28.5)  97 (32.1)  29 (9.6)  84 (27.8)  92 (30.5)  0 (0.0)  0 (0.0)  0 (0.0)  290 (96.0)  0 (0.0)  8 (2.6)  0 (0.0)  4 (1.3)  0 (0.0)  0 (0.0) | | | | | 4  139  68 (48.9)  71 (51.1)  19.0 + 3.3  19.0  (17.0–21.0)  0 (0.0)  0 (0.0)  4 (2.9)  40 (28.8)  62 (44.6)  33 (23.7)  63 (45.3)  76 (54.7)  64 (46.0)  44 (31.7)  17 (12.2)  11 (7.9)  3 (2.2)  39 (28.1)  26 (18.7)  16 (11.5)  58 (41.7)  27 (19.4)  13 (9.4)  55 (39.6)  44 (31.7)  0 (0.0)  0 (0.0)  0 (0.0)  0 (0.0)  91 (65.5)  0 (0.0)  13 (9.4)  0 (0.0)  10 (7.2)  25 (18.0) | | | | | Overall  756  408 (54.0)  348 (46.0)  9.9 + 6.6  8.0  (4.0–16.0)  296 (39.2)  121 (16.0)  110 (14.6)  92 (12.2)  93 (12.3)  44 (5.8)  562 (74.3)  194 (25.7)  320 (42.3)  249 (32.9)  113 (14.9)  59 (7.8)  15 (2.0)  180 (23.8)  194 (25.7)  145 (19.2)  237 (31.3)  220 (29.1)  109 (14.4)  195 (25.8)  232 (30.7)  78 (10.3)  99 (13.1)  138 (18.3)  290 (38.4)  91 (12.0)  8 (1.1)  13 (1.7)  4 (0.5)  10 (1.3)  25 (3.3) |
|  | Taiwan (China) | [83] | 0 - < 60 months | Male sex  Age (months)  0 - < 12  12 - < 24  24 - < 60  Site  North  Central  South  Siblings  0  1  2  > 2  Childcare attendance  History of acute otitis media  Upper respiratory infection within two weeks  Antibiotic use within two weeks  Breastfeeding  0  1 month  2 months  > 2 months  Household exposure to smoking  PCV7 vaccination > 1 dose | | 5276 (54.4)  2733 (28.2)  2795 (28.8)  4177 (43.0)  3281 (33.8)  3144 (32.4)  3280 (33.8)  3322 (34.2)  4298 (48.4)  1193 (12.3)  492 (5.1)  1781 (18.3)  894 (9.2)  2940 (30.3)  571 (5.9)  2705 (27.9)  960 (9.9)  1398 (14.4)  4642 (47.8)  4546 (46.8)  3 (0.2) | | | | | | | | | | | | | | | | | | | | | | |
|  |  | [84] | 2 months – 5 years (n = 5977) | Age > 2 years  With underlying disease  History of being breast milk-fed  Number of bathrooms > 2  Having at least one sibling  Sleep with parents or siblings Passive smoking  Childcare attendance  Influenza vaccination  PCV7 vaccination  History of otitis media  Upper respiratory tract infection in the last two weeks  Antibiotic use in the last two weeks  Influenza virus infection  *S. aureus* colonization | | 2605 (43.6)  442 (7.4)  4202 (70.3)  2302 (38.5)  3924 (65.7)  5395 (90.3)  2911 (48.7)  1125 (18.8)  2435 (40.7)  535 (9.0)  623 (10.4)  2092 (35.0)  389 (6.5)  15 (0.3)  1656 (27.7) | | | | | | | | | | | | | | | | | | | | | | |

Abbreviations: 95% CI – 95% confidence interval; BMI – body mass index; HIV – human immunodeficiency virus; IPD – invasive pneumococcal disease; IQR – interquartile range; PCV – pneumococcal conjugate vaccine; PCV7 – seven-valent pneumococcal conjugate vaccine; PCV10 – ten-valent pneumococcal conjugate vaccine; PCV13 – thirteen-valent pneumococcal conjugate vaccine; SD – standard deviation; WHO – World Health Organization. Footnotes: ^a^ As per countries listed under WHO regional offices[85]; **^b^** As available in individual studies; **^c^** World Bank Income status at the time the study was undertaken[86]; ^d^ Unless otherwise indicated; ^e^ This study was undertaken in high-income Israel (WHO European region) and lower-middle-income West Bank and Gaza (WHO Eastern Mediterranean regions)[76, 85, 86].

# References

1. Assefa A, Gelaw B, Shiferaw Y, Tigabu Z. Nasopharyngeal carriage and antimicrobial susceptibility pattern of *Streptococcus pneumoniae* among pediatric outpatients at Gondar University Hospital, North West Ethiopia. PEDN. 2013;54(5):315-21. doi: <https://dx.doi.org/10.1016/j.pedneo.2013.03.017>. PubMed PMID: 23680262.

2. Gebre T, Tadesse M, Aragaw D, Feye D, Beyene HB, Seyoum D, et al. Nasopharyngeal carriage and antimicrobial susceptibility patterns of *Streptococcus pneumoniae* among children under five in Southwest Ethiopia. Children. 2017;4(4). doi: 10.3390/children4040027. PubMed PMID: 28422083.

3. Wada FW, Tufa EG, Berheto TM, Solomon FB. Nasopharyngeal carriage of Streptococcus pneumoniae and antimicrobial susceptibility pattern among school children in South Ethiopia: post-vaccination era. BMC research notes. 2019;12(1):306. doi: <https://dx.doi.org/10.1186/s13104-019-4330-0>.

4. Haile AA, Gidebo DD, Ali MM. Colonization rate of Streptococcus pneumoniae, its associated factors and antimicrobial susceptibility pattern among children attending kindergarten school in Hawassa, southern Ethiopia. BMC Res Notes. 2019;12(1):344. Epub 2019/06/19. doi: 10.1186/s13104-019-4376-z. PubMed PMID: 31208447; PubMed Central PMCID: PMCPMC6580519.

5. Abdullahi O, Nyiro J, Lewa P, Slack M, Scott JA. The descriptive epidemiology of *Streptococcus pneumoniae* and *Haemophilus influenzae* nasopharyngeal carriage in children and adults in Kilifi district, Kenya. Ped Infect Dis J. 2008;27(1):59-64. doi: <https://dx.doi.org/10.1097/INF.0b013e31814da70c>. PubMed PMID: 18162940.

6. Abdullahi O, Karani A, Tigoi CC, Mugo D, Kungu S, Wanjiru E, et al. The prevalence and risk factors for pneumococcal colonization of the nasopharynx among children in Kilifi District, Kenya. PLoS One. 2012;7(2):e30787. doi: <https://dx.doi.org/10.1371/journal.pone.0030787>. PubMed PMID: 22363489.

7. Ousmane S, Diallo BA, Ouedraogo R, Sanda AA, Soussou AM, Collard JM. Serotype distribution and antimicrobial sensitivity profile of *Streptococcus pneumoniae c*arried in healthy toddlers before PCV13 introduction in Niamey, Niger. PLoS One. 2017;12(1):e0169547. doi: 10.1371/journal.pone.0169547. PubMed PMID: 28103262.

8. Bojang A, Jafali J, Egere U, Hill P, Antonio M, Jeffries D. Seasonality of pneumococcal nasopharyngeal carriage in rural Gambia determined within the context of a cluster randomized pneumococcal vaccine trial. PLoS One. 2015;10(7):13. PubMed PMID: CN-01130937.

9. Usuf E, Badji H, Bojang A, Jarju S, Ikumapayi UN, Antonio M, et al. Pneumococcal carriage in rural Gambia prior to the introduction of pneumococcal conjugate vaccine: a population-based survey. Trop Med Int Health. 2015;20(7):871-9. doi: <https://dx.doi.org/10.1111/tmi.12505>. PubMed PMID: 25778937.

10. Usuf E, Bojang A, Camara B, Jagne I, Oluwalana C, Bottomley C, et al. Maternal pneumococcal nasopharyngeal carriage and risk factors for neonatal carriage after the introduction of pneumococcal conjugate vaccines in The Gambia. Clin Microbiol Infect. 2018;24(4):389-95. Epub 2017/07/27. doi: 10.1016/j.cmi.2017.07.018. PubMed PMID: 28743545.

11. Hill PC, Akisanya A, Sankareh K, Cheung YB, Saaka M, Lahai G, et al. Nasopharyngeal carriage of *Streptococcus pneumoniae* in Gambian villagers. Clin Infect Dis 2006;43(6):673-9. doi: <https://dx.doi.org/10.1086/506941>. PubMed PMID: 16912937.

12. le Polain de Waroux O, Flasche S, Kucharski AJ, Langendorf C, Ndazima D, Mwanga-Amumpaire J, et al. Identifying human encounters that shape the transmission of *Streptococcus pneumoniae* and other acute respiratory infections. Epidemics. 2018;25:72-9. Epub 2018/07/29. doi: 10.1016/j.epidem.2018.05.008. PubMed PMID: 30054196; PubMed Central PMCID: PMCPMC6227246.

13. le Polain de Waroux O, Cohuet S, Ndazima D, Kucharski AJ, Juan-Giner A, Flasche S, et al. Characteristics of human encounters and social mixing patterns relevant to infectious diseases spread by close contact: a survey in Southwest Uganda. BMC Infect Dis. 2018;18(1):172. doi: 10.1186/s12879-018-3073-1.

14. Lindstrand A, Kalyango J, Alfven T, Darenberg J, Kadobera D, Bwanga F, et al. Pneumococcal carriage in children under five years in Uganda-will present pneumococcal conjugate vaccines be appropriate? PLoS One. 2016;11(11):e0166018. doi: 10.1371/journal.pone.0166018. PubMed PMID: 27829063.

15. Nackers F, Cohuet S, le Polain de Waroux O, Langendorf C, Nyehangane D, Ndazima D, et al. Carriage prevalence and serotype distribution of *Streptococcus pneumoniae* prior to 10-valent pneumococcal vaccine introduction: A population-based cross-sectional study in South Western Uganda, 2014. Vaccine. 2017;35(39):5271-7. Epub 2017/08/09. doi: 10.1016/j.vaccine.2017.07.081. PubMed PMID: 28784282; PubMed Central PMCID: PMCPMC6616034.

16. Coles CL, Kanungo R, Rahmathullah L, Thulasiraj RD, Katz J, Santosham M, et al. Pneumococcal nasopharyngeal colonization in young South Indian infants. Ped Infect Dis J. 2001;20(3):289-95. PubMed PMID: 11303832.

17. Coles CL, Sherchand JB, Khatry SK, Katz J, Leclerq SC, Mullany LC, et al. Nasopharyngeal carriage of *S. pneumoniae* among young children in rural Nepal. Trop Med Int Health. 2009;14(9):1025-33. Epub 2009/07/01. doi: 10.1111/j.1365-3156.2009.02331.x. PubMed PMID: 19563428; PubMed Central PMCID: PMCPMC2770711.

18. Nguyen HAT, Fujii H, Vu HTT, Parry CM, Dang AD, Ariyoshi K, et al. An alarmingly high nasal carriage rate of Streptococcus pneumoniae serotype 19F non-susceptible to multiple beta-lactam antimicrobials among Vietnamese children. BMC Infect Dis. 2019;19(1):241. Epub 2019/03/15. doi: 10.1186/s12879-019-3861-2. PubMed PMID: 30866853; PubMed Central PMCID: PMCPMC6416861.

19. Uddén F, Filipe M, Slotved HC, Yamba-Yamba L, Fuursted K, Pintar Kuatoko P, et al. Pneumococcal carriage among children aged 4 - 12 years in Angola 4 years after the introduction of a pneumococcal conjugate vaccine. Vaccine. 2020;38(50):7928-37. Epub 2020/11/05. doi: 10.1016/j.vaccine.2020.10.060. PubMed PMID: 33143954.

20. Adetifa IM, Antonio M, Okoromah CA, Ebruke C, Inem V, Nsekpong D, et al. Pre-vaccination nasopharyngeal pneumococcal carriage in a Nigerian population: epidemiology and population biology. PLoS One. 2012;7(1):e30548. doi: <https://dx.doi.org/10.1371/journal.pone.0030548>. PubMed PMID: 22291984.

21. Inverarity D, Diggle M, Ure R, Johnson P, Altstadt P, Mitchell T, et al. Molecular epidemiology and genetic diversity of pneumococcal carriage among children in Beni State, Bolivia. Trans R Soc Trop Med Hyg. 2011;105(8):445-51. doi: <https://dx.doi.org/10.1016/j.trstmh.2011.04.013>. PubMed PMID: 21714978.

22. Cardozo DM, Nascimento-Carvalho CM, Andrade AL, Silvany-Neto AM, Daltro CH, Brandao MA, et al. Prevalence and risk factors for nasopharyngeal carriage of *Streptococcus pneumoniae* among adolescents. J Med Microbiol. 2008;57(Pt 2):185-9. doi: <https://dx.doi.org/10.1099/jmm.0.47470-0>. PubMed PMID: 18201984.

23. Regev-Yochay G, Raz M, Dagan R, Porat N, Shainberg B, Pinco E, et al. Nasopharyngeal carriage of *Streptococcus pneumoniae* by adults and children in community and family settings. Clin Infect Dis 2004;38(5):632-9. doi: <https://dx.doi.org/10.1086/381547>. PubMed PMID: 14986245.

24. Farida H, Severin JA, Gasem MH, Keuter M, Wahyono H, van den Broek P, et al. Nasopharyngeal carriage of *Streptococcus pneumoniae* in pneumonia-prone age groups in Semarang, Java Island, Indonesia. PLoS One. 2014;9(1):e87431. doi: <https://dx.doi.org/10.1371/journal.pone.0087431>. PubMed PMID: 24498104.

25. Murad C, Dunne EM, Sudigdoadi S, Fadlyana E, Tarigan R, Pell CL, et al. Pneumococcal carriage, density, and co-colonization dynamics: A longitudinal study in Indonesian infants. IJID. 2019;86:73-81. doi: <https://dx.doi.org/10.1016/j.ijid.2019.06.024>.

26. Hu J, Sun X, Huang Z, Wagner AL, Carlson B, Yang J, et al. *Streptococcus pneumoniae* and *Haemophilus influenzae* type b carriage in Chinese children aged 12-18 months in Shanghai, China: a cross-sectional study. BMC Infect Dis. 2016;16:149. doi: <https://dx.doi.org/10.1186/s12879-016-1485-3>. PubMed PMID: 27080523.

27. Russell FM, Carapetis JR, Ketaiwai S, Kunabuli V, Taoi M, Biribo S, et al. Pneumococcal nasopharyngeal carriage and patterns of penicillin resistance in young children in Fiji. Ann Trop Paediatr 2006;26(3):187-97. doi: <https://dx.doi.org/10.1179/146532806X120273>. PubMed PMID: 16925955.

28. von Mollendorf C, Dunne EM, La Vincente S, Ulziibayar M, Suuri B, Luvsantseren D, et al. Pneumococcal carriage in children in Ulaanbaatar, Mongolia before and one year after the introduction of the 13-valent pneumococcal conjugate vaccine. Vaccine. 2019;37(30):4068-75. doi: <https://dx.doi.org/10.1016/j.vaccine.2019.05.078>.

29. Dunne EM, Choummanivong M, Neal EFG, Stanhope K, Nguyen CD, Xeuatvongsa A, et al. Factors associated with pneumococcal carriage and density in infants and young children in Laos PDR. PLoS One. 2019;14(10):e0224392. doi: <https://dx.doi.org/10.1371/journal.pone.0224392>.

30. Shiri T, Nunes MC, Adrian PV, Van Niekerk N, Klugman KP, Madhi SA. Interrelationship of *Streptococcus pneumoniae*, *Haemophilus influenzae* and *Staphylococcus aureus* colonization within and between pneumococcal-vaccine naive mother-child dyads. BMC Infect Dis. 2013;13:483. doi: <https://dx.doi.org/10.1186/1471-2334-13-483>. PubMed PMID: 24134472.

31. Vanker A, Nduru PM, Barnett W, Dube FS, Sly PD, Gie RP, et al. Indoor air pollution and tobacco smoke exposure: impact on nasopharyngeal bacterial carriage in mothers and infants in an African birth cohort study. ERJ Open Res. 2019;5(1). Epub 2019/02/12. doi: 10.1183/23120541.00052-2018. PubMed PMID: 30740462; PubMed Central PMCID: PMCPMC6360211.

32. Skosana Z, Von Gottberg A, Olorunju S, Mohale T, Du Plessis M, Adams T, et al. Non-vaccine serotype pneumococcal carriage in healthy infants in South Africa following introduction of the 13-valent pneumococcal conjugate vaccine. S Afr Med J. 2021;111(2):143-8. Epub 2021/05/05. doi: 10.7196/SAMJ.2021.v111i2.14626. PubMed PMID: 33944725.

33. Reis JN, Palma T, Ribeiro GS, Pinheiro RM, Ribeiro CT, Cordeiro SM, et al. Transmission of *Streptococcus pneumoniae* in an urban slum community. J Infect Dis. 2008;57(3):204-13. doi: <https://dx.doi.org/10.1016/j.jinf.2008.06.017>. PubMed PMID: 18672297.

34. Menezes AP, Azevedo J, Leite MC, Campos LC, Cunha M, Carvalho Mda G, et al. Nasopharyngeal carriage of *Streptococcus pneumoniae* among children in an urban setting in Brazil prior to PCV10 introduction. Vaccine. 2016;34(6):791-7. doi: <https://dx.doi.org/10.1016/j.vaccine.2015.12.042>. PubMed PMID: 26742946.

35. Neves FPG, Cardoso NT, Snyder RE, Marlow MA, Cardoso CAA, Teixeira LM, et al. Pneumococcal carriage among children after four years of routine 10-valent pneumococcal conjugate vaccine use in Brazil: The emergence of multidrug resistant serotype 6C. Vaccine. 2017;35(21):2794-800. doi: 10.1016/j.vaccine.2017.04.019. PubMed PMID: 28431817.

36. Toledo ME, Casanova MF, Linares-Perez N, Garcia-Rivera D, Torano Peraza G, Barcos Pina I, et al. Prevalence of pneumococcal nasopharyngeal carriage among children 2-18 months of age: baseline study pre-introduction of pneumococcal vaccination in Cuba. Ped Infect Dis J. 2017;36(1):e22-e8. doi: 10.1097/inf.0000000000001341. PubMed PMID: 27649366.

37. Rivera-Olivero IA, del Nogal B, Sisco MC, Bogaert D, Hermans PW, de Waard JH. Carriage and invasive isolates of *Streptococcus pneumoniae* in Caracas, Venezuela: the relative invasiveness of serotypes and vaccine coverage. Eur J Clin Microbiol Infect Dis. 2011;30(12):1489-95. doi: <https://dx.doi.org/10.1007/s10096-011-1247-5>. PubMed PMID: 21499972.

38. Verhagen LM, Hermsen M, Rivera-Olivero IA, Sisco MC, de Jonge MI, Hermans PW, et al. Nasopharyngeal carriage of respiratory pathogens in Warao Amerindians: significant relationship with stunting. Trop Med Int Health. 2017;22(4):407-14. doi: 10.1111/tmi.12835. PubMed PMID: 28072501.

39. Karami M, Hosseini SM, Hashemi SH, Ghiasvand S, Zarei O, Safari N, et al. Prevalence of nasopharyngeal carriage of Streptococcus pneumoniae in children 7 to 14 years in 2016: A survey before pneumococcal conjugate vaccine introduction in Iran. Human vaccines & immunotherapeutics. 2019;15(9):2178-82. doi: <https://dx.doi.org/10.1080/21645515.2018.1539601>.

40. Korona-Glowniak I, Malm A. Characteristics of *Streptococcus pneumoniae* strains colonizing upper respiratory tract of healthy preschool children in Poland. Sci World J. 2012.

41. Ozdemir B, Beyazova U, Camurdan AD, Sultan N, Ozkan S, Sahin F. Nasopharyngeal carriage of *Streptococcus pneumoniae* in healthy Turkish infants. J Infect Dis. 2008;56(5):332-9. doi: <https://dx.doi.org/10.1016/j.jinf.2008.02.010>. PubMed PMID: 18377994.

42. Uzuner A, Ilki A, Akman M, Gundogdu E, Erbolukbas R, Kokacya O, et al. Nasopharyngeal carriage of penicillin-resistant *Streptococcus pneumoniae* in healthy children. Turk J Pediatr 2007;49(4):370-8. Epub 2008/02/06. PubMed PMID: 18246737.

43. Ozdemir H, Ciftci E, Durmaz R, Guriz H, Aysev AD, Karbuz A, et al. Risk factors for nasopharyngeal carriage of *Streptococcus pneumoniae* in healthy Turkish children after the addition of heptavalent pneumococcal conjugate vaccine (PCV7) to the national vaccine schedule. Turk J Pediatr. 2013;55(6):575-83. Epub 2014/03/01. PubMed PMID: 24577974.

44. Arvas A, Cokugras H, Gur E, Gonullu N, Taner Z, Bahar Tokman H. Pneumococcal nasopharyngeal carriage in young healthy children after pneumococcal conjugate vaccine in Turkey. Balkan Med J. 2017. doi: 10.4274/balkanmedj.2016.1256. PubMed PMID: 28443585.

45. Neal EFG, Nguyen C, Ratu FT, Matanitobua S, Dunne EM, Reyburn R, et al. A comparison of pneumococcal nasopharyngeal carriage in very young Fijian infants born by vaginal or Cesarean delivery. JAMA Netw Open. 2019;2(10):e1913650. Epub 2019/10/19. doi: 10.1001/jamanetworkopen.2019.13650. PubMed PMID: 31626319; PubMed Central PMCID: PMCPMC6813584.

46. Neal EFG, Flasche S, Nguyen CD, Ratu FT, Dunne EM, Koyamaibole L, et al. Associations between ethnicity, social contact, and pneumococcal carriage three years post-PCV10 in Fiji. Vaccine. 2020;38(2):202-11. doi: <https://dx.doi.org/10.1016/j.vaccine.2019.10.030>.

47. Neal EFG, Nguyen CD, Ratu FT, Dunne EM, Kama M, Ortika BD, et al. Factors associated with pneumococcal carriage and density in children and adults in Fiji, using four cross-sectional surveys. PLoS One. 2020;15(4):e0231041. doi: <https://dx.doi.org/10.1371/journal.pone.0231041>.

48. Ricketson LJ, Wood ML, Vanderkooi OG, MacDonald JC, Martin IE, Demczuk WH, et al. Trends in asymptomatic nasopharyngeal colonization with *Streptococcus pneumoniae* after introduction of the 13-valent pneumococcal conjugate vaccine in Calgary, Canada. Ped Infect Dis J. 2014;33(7):724-30. doi: <https://dx.doi.org/10.1097/INF.0000000000000267>. PubMed PMID: 24463806.

49. Samore MH, Magill MK, Alder SC, Severina E, Morrison-De Boer L, Lyon JL, et al. High rates of multiple antibiotic resistance in *Streptococcus pneumoniae* from healthy children living in isolated rural communities: association with cephalosporin use and intrafamilial transmission. Pediatrics. 2001;108(4):856-65. PubMed PMID: 11581436.

50. Millar EV, O'Brien KL, Zell ER, Bronsdon MA, Reid R, Santosham M. Nasopharyngeal carriage of *Streptococcus pneumoniae* in Navajo and White Mountain Apache children before the introduction of pneumococcal conjugate vaccine. Ped Infect Dis J. 2009;28(8):711-6. Epub 2009/07/14. doi: 10.1097/INF.0b013e3181a06303. PubMed PMID: 19593248.

51. Cheng Immergluck L, Kanungo S, Schwartz A, McIntyre A, Schreckenberger PC, Diaz PS. Prevalence of *Streptococcus pneumoniae* and *Staphylococcus aureus* nasopharyngeal colonization in healthy children in the United States. Epidemiol Infect. 2004;132(2):159-66. PubMed PMID: 15061489.

52. Finkelstein JA, Huang SS, Daniel J, Rifas-Shiman SL, Kleinman K, Goldmann D, et al. Antibiotic-resistant *Streptococcus pneumoniae* in the heptavalent pneumococcal conjugate vaccine era: predictors of carriage in a multicommunity sample. Pediatrics. 2003;112(4):862-9. PubMed PMID: 14523178.

53. Huang SS, Finkelstein JA, Rifas-Shiman SL, Kleinman K, Platt R. Community-level predictors of pneumococcal carriage and resistance in young children. Am J Epidemiol. 2004;159(7):645-54. PubMed PMID: 15033642.

54. Huang SS, Hinrichsen VL, Stevenson AE, Rifas-Shiman SL, Kleinman K, Pelton SI, et al. Continued impact of pneumococcal conjugate vaccine on carriage in young children. Pediatrics. 2009;124(1):e1-11. doi: <https://dx.doi.org/10.1542/peds.2008-3099>. PubMed PMID: 19564254.

55. Moore MR, Hyde TB, Hennessy TW, Parks DJ, Reasonover AL, Harker-Jones M, et al. Impact of a conjugate vaccine on community-wide carriage of nonsusceptible *Streptococcus pneumoniae* in Alaska. J Infect Dis. 2004;190(11):2031-8. doi: <https://dx.doi.org/10.1086/425422>. PubMed PMID: 15529269.

56. Park SY, Moore MR, Bruden DL, Hyde TB, Reasonover AL, Harker-Jones M, et al. Impact of conjugate vaccine on transmission of antimicrobial-resistant *Streptococcus pneumoniae* among Alaskan children. Ped Infect Dis J. 2008;27(4):335-40. doi: <https://dx.doi.org/10.1097/INF.0b013e318161434d>. PubMed PMID: 18316986.

57. Lee GM, Kleinman K, Pelton SI, Hanage W, Huang SS, Lakoma M, et al. Impact of 13-Valent Pneumococcal Conjugate Vaccination on *Streptococcus pneumoniae* Carriage in Young Children in Massachusetts. J Pediatric Infect Dis Soc. 2014;3(1):23-32. doi: 10.1093/jpids/pit057. PubMed PMID: 24567842.

58. Hsu KK, Rifas-Shiman SL, Shea KM, Kleinman KP, Lee GM, Lakoma M, et al. Do community-level predictors of pneumococcal carriage continue to play a role in the conjugate vaccine era? Epidemiol Infect. 2014;142(2):379-87. doi: <https://dx.doi.org/10.1017/S0950268813000794>. PubMed PMID: 23731707.

59. Reisman J, Rudolph K, Bruden D, Hurlburt D, Bruce MG, Hennessy T. Risk factors for pneumococcal colonization of the nasopharynx in Alaska native adults and children. J Pediatric Infect Dis Soc. 2014;3(2):104-11. doi: 10.1093/jpids/pit069. PubMed PMID: 26625363.

60. Wroe PC, Lee GM, Finkelstein JA, Pelton SI, Hanage WP, Lipsitch M, et al. Pneumococcal carriage and antibiotic resistance in young children before 13-valent conjugate vaccine. Ped Infect Dis J. 2012;31(3):249-54. doi: <https://dx.doi.org/10.1097/INF.0b013e31824214ac>. PubMed PMID: 22173142.

61. Koliou MG, Andreou K, Lamnisos D, Lavranos G, Iakovides P, Economou C, et al. Risk factors for carriage of *Streptococcus pneumoniae* in children. BMC Pediatr. 2018;18(1):144. Epub 2018/04/28. doi: 10.1186/s12887-018-1119-6. PubMed PMID: 29699525; PubMed Central PMCID: PMCPMC5921789.

62. Memish ZA, Assiri A, Almasri M, Alhakeem RF, Turkestani A, Al Rabeeah AA, et al. Impact of the Hajj on pneumococcal transmission. Clin Microbiol Infect. 2015;21(1):77.e11-8. doi: <https://dx.doi.org/10.1016/j.cmi.2014.07.005>. PubMed PMID: 25636939.

63. Cohen R, Levy C, Bonnet E, Thollot F, Boucherat M, Fritzell B, et al. Risk factors for serotype 19A carriage after introduction of 7-valent pneumococcal vaccination. BMC Infect Dis. 2011;11:95. doi: <https://dx.doi.org/10.1186/1471-2334-11-95>. PubMed PMID: 21501471.

64. Hoang VT, Dao TL, Ly TDA, Belhouchat K, Chaht KL, Gaudart J, et al. The dynamics and interactions of respiratory pathogen carriage among French pilgrims during the 2018 Hajj. Emerg Microbes Infect. 2019;8(1):1701-10. Epub 2019/11/22. doi: 10.1080/22221751.2019.1693247. PubMed PMID: 31749410; PubMed Central PMCID: PMCPMC6882464.

65. Navne JE, Borresen ML, Slotved HC, Andersson M, Melbye M, Ladefoged K, et al. Nasopharyngeal bacterial carriage in young children in Greenland: a population at high risk of respiratory infections. Epidemiol Infect. 2016;144(15):3226-36. doi: 10.1017/s0950268816001461. PubMed PMID: 27405603.

66. Ansaldi F, de Florentiis D, Canepa P, Zancolli M, Martini M, Orsi A, et al. Carriage of *Streptococcus pneumoniae* 7 years after implementation of vaccination program in a population with very high and long-lasting coverage, Italy. Vaccine. 2012;30(13):2288-94. doi: <https://dx.doi.org/10.1016/j.vaccine.2012.01.067>. PubMed PMID: 22306795.

67. Camilli R, Daprai L, Cavrini F, Lombardo D, D'Ambrosio F, Del Grosso M, et al. Pneumococcal carriage in young children one year after introduction of the 13-valent conjugate vaccine in Italy. PLoS One. 2013;8(10):e76309. doi: <https://dx.doi.org/10.1371/journal.pone.0076309>. PubMed PMID: 24124543.

68. Camilli R, Vescio MF, Giufre M, Daprai L, Garlaschi ML, Cerquetti M, et al. Carriage of *Haemophilus influenzae* is associated with pneumococcal vaccination in Italian children. Vaccine. 2015;33(36):4559-64. doi: <https://dx.doi.org/10.1016/j.vaccine.2015.07.009>. PubMed PMID: 26190092.

69. Zuccotti G, Mameli C, Daprai L, Garlaschi ML, Dilillo D, Bedogni G, et al. Serotype distribution and antimicrobial susceptibilities of nasopharyngeal isolates of *Streptococcus pneumoniae* from healthy children in the 13-valent pneumococcal conjugate vaccine era. Vaccine. 2014;32(5):527-34. doi: 10.1016/j.vaccine.2013.12.003. PubMed PMID: 24342249.

70. Almeida ST, Nunes S, Santos Paulo AC, Valadares I, Martins S, Breia F, et al. Low prevalence of pneumococcal carriage and high serotype and genotype diversity among adults over 60 years of age living in Portugal. PLoS One. 2014;9(3):e90974. doi: <https://dx.doi.org/10.1371/journal.pone.0090974>. PubMed PMID: 24604030.

71. Alfayate Miguélez S, Yague Guirao G, Menasalvas Ruíz AI, Sanchez-Solís M, Domenech Lucas M, González Camacho F, et al. Impact of pneumococcal vaccination in the nasopharyngeal carriage of *Streptococcus pneumoniae* in healthy children of the Murcia Region in Spain. Vaccines. 2020;9(1). Epub 2021/01/01. doi: 10.3390/vaccines9010014. PubMed PMID: 33379235; PubMed Central PMCID: PMCPMC7823743.

72. Bogaert D, van Belkum A, Sluijter M, Luijendijk A, de Groot R, Rumke HC, et al. Colonisation by *Streptococcus pneumoniae* and *Staphylococcus aureus* in healthy children. Lancet. 2004;363(9424):1871-2. doi: <https://dx.doi.org/10.1016/S0140-6736(04)16357-5>. PubMed PMID: 15183627.

73. Labout JA, Duijts L, Arends LR, Jaddoe VW, Hofman A, de Groot R, et al. Factors associated with pneumococcal carriage in healthy Dutch infants: the generation R study. J Pediatr. 2008;153(6):771-6. doi: <https://dx.doi.org/10.1016/j.jpeds.2008.05.061>. PubMed PMID: 18621390.

74. Gils E, Veenhoven R, Rodenburg G, Hak E, Sanders E. Effect of 7-valent pneumococcal conjugate vaccine on nasopharyngeal carriage with *Haemophilus influenzae* and *Moraxella catarrhalis* in a randomized controlled trial. Vaccine. 2011;29(44):7595-8. doi: 10.1016/j.vaccine.2011.08.049. PubMed PMID: CN-00806054.

75. van Gils EJ, Veenhoven RH, Hak E, Rodenburg GD, Bogaert D, Ijzerman EP, et al. Effect of reduced-dose schedules with 7-valent pneumococcal conjugate vaccine on nasopharyngeal pneumococcal carriage in children: a randomized controlled trial. Jama. 2009;302(2):159-67. Epub 2009/07/09. doi: 10.1001/jama.2009.975. PubMed PMID: 19584345.

76. Daana M, Rahav G, Hamdan A, Thalji A, Jaar F, Abdeen Z, et al. Measuring the effects of pneumococcal conjugate vaccine (PCV7) on *Streptococcus pneumoniae* carriage and antibiotic resistance: the Palestinian-Israeli Collaborative Research (PICR). Vaccine. 2015;33(8):1021-6. doi: 10.1016/j.vaccine.2015.01.003. PubMed PMID: 25593104.

77. Mackenzie GA, Leach AJ, Carapetis JR, Fisher J, Morris PS. Epidemiology of nasopharyngeal carriage of respiratory bacterial pathogens in children and adults: cross-sectional surveys in a population with high rates of pneumococcal disease. BMC Infect Dis. 2010;10:304. doi: <https://dx.doi.org/10.1186/1471-2334-10-304>. PubMed PMID: 20969800.

78. Sung RY, Ling JM, Fung SM, Oppenheimer SJ, Crook DW, Lau JT, et al. Carriage of *Haemophilus influenzae* and *Streptococcus pneumoniae* in healthy Chinese and Vietnamese children in Hong Kong. Acta Paediatr. 1995;84(11):1262-7. PubMed PMID: 8580623.

79. Chan KC, Subramanian R, Chong P, Nelson EA, Lam HS, Li AM, et al. Pneumococcal carriage in young children after introduction of PCV13 in Hong Kong. Vaccine. 2016;34(33):3867-74. doi: 10.1016/j.vaccine.2016.05.047. PubMed PMID: 27265449.

80. Otsuka T, Chang B, Shirai T, Iwaya A, Wada A, Yamanaka N, et al. Individual risk factors associated with nasopharyngeal colonization with *Streptococcus pneumoniae* and *Haemophilus influenzae*: a Japanese birth cohort study. Ped Infect Dis J. 2013;32(7):709-14. doi: <https://dx.doi.org/10.1097/INF.0b013e31828701ea>. PubMed PMID: 23411622.

81. Ueno M, Ishii Y, Tateda K, Anahara Y, Ebata A, Iida M, et al. Prevalence and risk factors of nasopharyngeal carriage of *Streptococcus pneumoniae* in healthy children in Japan. Jpn J Infect Dis. 2013;66(1):22-5. PubMed PMID: 23429080.

82. Chang B, Akeda H, Nakamura Y, Hamabata H, Ameku K, Toma T, et al. Impact of thirteen-valent pneumococcal conjugate vaccine on nasopharyngeal carriage in healthy children under 24 months in Okinawa, Japan. JIC. 2020;26(5):465-70. doi: <https://dx.doi.org/10.1016/j.jiac.2019.12.009>.

83. Hsieh YC, Chiu CH, Chang KY, Huang YC, Chen CJ, Kuo CY, et al. The impact of the heptavalent pneumococcal conjugate vaccine on risk factors for *Streptococcus pneumoniae* carriage in children. Ped Infect Dis J. 2012;31(9):e163-8. doi: <https://dx.doi.org/10.1097/INF.0b013e31825cb9f9>. PubMed PMID: 22592521.

84. Kuo CY, Hwang KP, Hsieh YC, Cheng CH, Huang FL, Shen YH, et al. Nasopharyngeal carriage of *Streptococcus pneumoniae* in Taiwan before and after the introduction of a conjugate vaccine. Vaccine. 2011;29(32):5171-7. doi: <https://dx.doi.org/10.1016/j.vaccine.2011.05.034>. PubMed PMID: 21621578.

85. World Health Organization. WHO/Who we are/Regional offices 2020 [cited 2020 April 3]. Available from: <https://www.who.int/about/who-we-are/regional-offices>.

86. World Bank Country and Lending Groups [Internet]. World Bank. 2020 [cited 2020 Jan 26].
