# Supplementary material for: Factors associated with pneumococcal nasopharyngeal carriage: A systematic review
Source: PLOS Glob Public Health. 2022 Apr 11;2(4):e0000327. doi: 10.1371/journal.pgph.0000327 (PMC10021834; doi:10.1371/journal.pgph.0000327)
Supplement: S3 Table — (DOCX) [file pgph.0000327.s005.docx]

**S3 Table. Details of laboratory methods in studies reporting risk factors for nasopharyngeal pneumococcal carriage, stratified by World Bank income status, WHO region, and country.**

| **WHO region^a^** | **Country** | **Ref** | **Swab material** | **Sample transport** | **Sample storage** | **Identification method** | **Culture medium** |
| --- | --- | --- | --- | --- | --- | --- | --- |
| **Low–income countries^b^ (n = 17)** | | | | | | | |
| Africa | Ethiopia | [1] | Cotton | Amies | NR | Gram staining, α-hemolysis, optochin susceptibility, bile solubility | Blood agar supplemented with 5 µg/ml gentamicin, incubated at 37° C in a 5% CO_2_ enriched atmosphere for 24 -48 hours |
|  |  | [2] | Rayon | STGG | -20°C | α-hemolysis, optochin susceptibility, bile solubility | Tryptone soy agar supplemented with 5% sheep blood and 5 µg/ml gentamicin, incubated at 37° C in 5-10% CO_2_ enriched atmosphere for 24 hours |
|  |  | [3] | Cotton | STGG | NR | Colony morphology, hemolytic activity, optochin susceptibility, bile solubility | Colombia agar plates with 5% sheep blood and 5 µg/ml gentamicin, incubated at 35-37° C in 5-10% CO_2_ enriched atmosphere for 24 hours |
|  |  | [4] | Rayon | NR | NR | α-hemolysis, optochin susceptibility, bile solubility | Sheep blood agar with 5 µg/ml gentamicin, incubated at 37° C in a 5% CO_2_ enriched atmosphere for 18 – 20 hours |
|  | Kenya | [5] | Rayon | STGG | -80°C up to 2 months | Colony morphology, α-hemolysis, optochin susceptibility, bile solubility | 7% horse blood agar supplemented 2.5 µg/ml and 7% incubated overnight at 37° C in a 5% CO_2_ enriched atmosphere |
|  |  |  |  |  |  |  |  |
|  |  |  |  |  |  |  |  |
|  |  | [6] | Dacron | STGG, transported at ambient temperature | NR | α-hemolysis, optochin susceptibility, presence of a capsule | Blood agar, supplemented with 2.5 mg/ml gentamicin, incubated at 37° C overnight in a 5% CO_2_ enriched atmosphere |
|  | Niger | [7] | Cotton | STGG | Confirmed *S. pneumonia* isolates stored at -80°C | Gram-staining, α-hemolysis, oxidase, and catalase negative colonies with optochin  inhibition zones > 14 mm further confirmed by latex agglutination | Columbia agar containing 15 mg/L Nalidixic acid, supplemented with 7%-10% sheep blood, incubated at 36° + 0.5° C with ~5% CO_2_ enriched atmosphere for 18 + 2 hours; selected colonies purified on new blood agar plates with an optochin disc, and incubated again under the same conditions |
|  | The Gambia | [8] | Calcium alginate | STGG | -70°C | Colony morphology, optochin susceptibility, bile solubility | Blood agar supplemented with gentamicin, incubated at 35° C in a 5% CO_2_ enriched atmosphere |
|  |  | [9] | Calcium alginate | STGG | -70°C | Colony morphology, α-hemolysis, optochin susceptibility, bile solubility | 5.0 µg/ml gentamicin blood agar, incubated overnight at 37° C in a 5% CO_2_ enriched atmosphere |
|  |  |  |  |  |  |  |  |
|  |  |  |  |  |  |  |  |
|  |  |  |  |  |  |  |  |
|  |  | [10] | NR | NR | NR | NR | NR |
|  |  | [11] | NR | STGG, placed in a cold box for transport within 8 hours of collection | -70°C | Colony morphology, optochin susceptibility | Gentamicin blood agar |
|  |  |  |  |  |  |  |  |
|  |  |  |  |  |  |  |  |
|  |  |  |  |  |  |  |  |
|  |  |  |  |  |  |  |  |
|  | Uganda | [12] | Nylon (flocked) | STGG, transported at 4°C in cool boxes to be frozen within 8 hours of collection | -20°C | Colony morphology, optochin susceptibility, bile solubility | Selective agar plate of 5 mg/l gentamicin-Columbia agar with 5% sheep blood, incubated overnight at 37° C in a 5% CO_2_ enriched atmosphere |
|  |  |  |  |  |  |  |  |
|  |  |  |  |  |  |  |  |
|  |  | [13] | Calcium alginate | Amies | Isolates stored at -70°C | Colony morphology, α-hemolysis, optochin susceptibility | Agar plate supplemented with 7% whole sheep blood and chocolate agar, incubated at 37° C in 5-10% CO_2_ enriched atmosphere for 24-48 hours |
|  |  | [14] | Nylon (flocked) | STGG, transported at 4°C in cool boxes to be frozen within 8 hours of collection | -20°C | Colony morphology, optochin susceptibility, bile solubility | Agar plate of 5 mg/L gentamicin-Columbia agar with 5% sheep blood, incubated at 37° C overnight in a 5% CO_2_ enriched atmosphere |
|  |  |  |  |  |  |  |  |
|  |  |  |  |  |  |  |  |
|  |  |  |  |  |  |  |  |
| South-East Asia | India | [15] | Rayon | Amies | Transported within 10 hours of collection | Colony morphology, optochin susceptibility, bile solubility | Blood agar containing 5% sheep blood and 2.5 mg/l gentamicin, incubated at 37° C in a 5% CO_2_ enriched atmosphere for 18-24 hours |
|  |  |  |  |  |  |  |  |
|  |  |  |  |  |  |  |  |
|  | Nepal | [16] | Calcium alginate | STGG, transported on ice within 6 hours | -80°C | Colony morphology, optochin susceptibility, bile solubility | Tryptic soy agar, with 5% sheep blood, and 5 µg/ml gentamicin, incubated at 37° C in a 5% CO_2_ enriched atmosphere for 18 -24 hours |
| Western Pacific | Vietnam | [17] | NR | NR | NR | Colony morphology, α-hemolysis, optochin susceptibility, bile solubility if the optochin test was inconclusive.  Genotypic (*lytA* or *cpsA* PCR) | Trypticase soy agar with 7% defibrinated rabbit blood; serial dilution (1:10^2^ to 10^7^) was performed before streaking to obtain single colonies. Plates incubated in CO_2_ incubators |
| **Lower-middle-income countries^b^ (n = 11)** | | | | | | | |
| Africa | Angola | [18] | Nylon | STGG | Cooler bags with ice-blocks for transport, stored at -80°C | Colony morphology, optochin susceptibility | Blood agar and chocolate blood agar at 35° C in a 5% CO_2_ enriched atmosphere for ~ 40 hours |
|  | Nigeria | [19] | Calcium-alginate | STGG | -30°C for transport | Colony morphology, α-hemolysis, optochin susceptibility, bile solubility | 5% sheep blood agar, with gentamicin, incubated at 37° C in a 5% CO_2_ enriched atmosphere |
| Americas | Bolivia | [20] | Dacron | STGG | -20°C | α-hemolysis, optochin susceptibility | 5% horse blood agar, incubation at 37° C in CO_2_ enriched atmosphere for > 18 hours |
|  | Brazil | [21] | Cotton | Amies | Frozen in skim milk, transported in dry ice | Gram-staining,  Colony morphology, optochin susceptibility, bile solubility | Trypticase soy agar with 5% sheep blood and 5 µg/ml, incubated at 37° C in a 5% CO_2_ enriched atmosphere for 18 – 24 hours |
| Eastern Mediterranean | West Bank and Gaza | [22] | Rayon | Amies | NR | Colony morphology, α-hemolysis, optochin susceptibility | Trypticase soy agar plates supplemented with sheep-blood and 5 µg/ml gentamicin, incubated overnight at 35° C in a 5% CO_2_ enriched air |
| South-East Asia | Indonesia | [23] | Rayon | Amies with charcoal | NR | Optochin susceptibility | 5% sheep blood agar with 5 mg/l gentamicin, incubated at 35° C in a 5% CO_2_ enriched atmosphere for 48 hours |
|  |  | [24] | NR | STGG | Transported in a cool box, then stored at -70°C | *lytA* qPCR positive (threshold cycle value < 35) and *lytA* qPCR equivocal (threshold cycle 35 – 40) | Real-time qPCR targeting the *lytA* gene in STGG samples |
| Western Pacific | China | [25] | Calcium alginate | Plated immediately | Isolates stored at -70°C | Colony morphology, optochin susceptibility, bile solubility | Columbia and chocolate agar, incubated at 37° C in a 5% CO_2_ enriched atmosphere for 24 – 48 hours |
|  | Fiji | [26] | Cotton | STGG, same day transport at < 5°C | -70°C | Colony morphology, α-hemolysis, optochin susceptibility, bile solubility | 2.5 mg/l gentamicin 5% sheep blood Columbia agar plate; incubated at 37° C in a 5% CO_2_ for 18-24 hours |
|  | Lao People’s Democratic Republic | [27] | Flocked nylon | STGG, same-day transport | Stored at -80°C, then transported on dry ice | *lytA* qPCR positive (threshold cycle value < 35) and lytA qPCR equivocal (threshold cycle 35 – 40) | Real-time qPCR targeting the *lytA* gene in STGG samples |
|  | Mongolia | [28] | Flocked nylon | STGG, same-day transport | Stored at -80°C, then transported on dry ice | *lytA* qPCR positive (threshold cycle value < 35) and lytA qPCR equivocal (threshold cycle 35 – 40) | Real-time qPCR targeting the *lytA* gene in STGG samples |
| **Upper-middle-income countries^b^ (n = 18)** | | | | | | | |
| Africa | South Africa | [29] | Dacron | NR | NR | NR | Standard methods (no details provided) |
|  |  | [30] | NR | STGG | Transported on ice, stored at -80°C | Colony morphology, α-hemolysis, optochin susceptibility, confirmed using *lytA* qPCR | Colombia blood agar with 2% agar, 5% horse blood and 4 mg/mL^-1^ gentamicin; incubated at 37° C in a 5% CO_2_ enriched atmosphere overnight |
|  |  | [31] | Calcium alginate | STGG | NR | Colony morphology, α-hemolysis, optochin susceptibility | 5% sheep blood with 5mg/mL gentamicin; incubated at 37° C in a 5% CO_2_ enriched atmosphere |
| Americas | Brazil | [32] | Calcium-alginate | NA plated immediately | NR | Gram-staining, colony morphology, optochin susceptibility, bile solubility | Agar plates with 5% sheep blood and 5.0 µg/mL of gentamicin, incubated at 35° C in a 5% CO_2_ enriched atmosphere < 48 hours |
|  |  |  |  |  |  |  |  |
|  |  | [33] | Calcium-alginate | Modified Stuart’s | One colony per plate stored at -70°C | Colony morphology, α-hemolysis, optochin susceptibility | Agar plates with 5% sheep blood and 5 µg/ml of gentamicin; incubated at 35° C in a 5% CO_2_ enriched atmosphere < 48 hours |
|  |  | [34] | Nylon (flocked) | STGG | -180°C for three months | α-hemolysis, optochin susceptibility, bile solubility | Broth enrichment culture using 2 ml of Todd-Hewett broth, containing 0.5% yeast extract combined with 0.4 ml of rabbit serum |
|  | Cuba | [35] | Nylon (flocked) | STGG | Isolates stored at -70°C | α-hemolysis, optochin susceptibility, bile solubility | Trypticase soy agar, supplemented with 5% sheep blood plus gentamicin, incubated at 37° C for 18-24 hours in a CO_2_ incubator |
|  | Venezuela | [36] | NR | STGG at 4 – 7°C | NR | NR | Blood agar with and without gentamicin, incubated overnight at 36° C in a CO_2_ enriched atmosphere |
|  |  | [37] | NR | STGG | < 3 days at 4°C before storage at – 20°C until analysis | NR | Blood and chocolate agar plates |
|  |  |  |  |  |  |  |  |
|  |  |  |  |  |  |  |  |
|  |  |  |  |  |  |  |  |
| Eastern Mediterranean | Iran | [38] | NR | NR | NR | Colony morphology, α-hemolysis, optochin susceptibility, bile solubility | Enriched chocolate agar plates and selective sheep blood agar plates with 5 mg/mL gentamicin; incubated at 37° C in a 5% CO_2_ enriched atmosphere overnight |
| Europe | Poland | [39] | NR | NR | Isolates stored at -70°C | Colony morphology, α-hemolysis, optochin susceptibility, bile solubility, latex agglutination | Mueller-Hinton agar with 5% sheep blood and 5 mg/l gentamicin, incubated aerobically at 35° C in a CO_2_ enriched atmosphere for 24 – 48 hours |
|  | Turkey | [40] | Calcium-alginate | Stuart’s, transported within the day of collection | Isolates stored at -70°C | Gram stain morphology, colony morphology, optochin susceptibility, bile solubility | Agar plates supplemented with 5% defibrinated sheep blood, incubated overnight at 37° C in a 5% CO_2_ enriched atmosphere |
|  |  | [41] | Cotton | Stuart’s | NR | Gram staining, catalase-negative, α-hemolysis, optochin susceptibility, bile solubility | Columbia agar supplemented with 5% sheep blood, incubated at 35° C for 24 hours |
|  |  | [42] | Calcium-alginate | NR | NR | Gram staining, colony morphology, optochin susceptibility, bile solubility | Agar plates supplemented with 5% defibrinated sheep blood, incubated overnight at 37° C in 5% - 10% CO_2_ enriched atmosphere |
|  |  | [43] | NR | Liquid Amies | NR | Gram staining, colony morphology, optochin susceptibility, bile solubility | 5% sheep blood agar and chocolate agar |
| Western Pacific | Fiji | [44] | Flocked nylon | STGG, same-day transport | Stored at -80°C, then transported on dry ice | *lytA* qPCR positive (threshold cycle value < 35) and *lytA* qPCR equivocal (threshold cycle 35 – 40) | Real-time qPCR targeting the *lytA* gene in STGG samples |
|  |  | [45] | Flocked nylon | STGG, same-day transport | Stored at -80°C, then transported on dry ice | *lytA* qPCR positive (threshold cycle value < 35) and *lytA* qPCR equivocal (threshold cycle 35 – 40) | Real-time qPCR targeting the *lytA* gene in STGG samples |
|  |  | [46] | Flocked nylon | STGG, same-day transport | Stored at -80°C, then transported on dry ice | *lytA* qPCR positive (threshold cycle value < 35) and *lytA* qPCR equivocal (threshold cycle value 35 – 40) | Real-time qPCR targeting the *lytA* gene in STGG samples |
| **High-income- countries^b^ (n = 36)** | | | | | | | |
| Americas | Canada | [47] | Dacron | Medium NR; transported at ambient temperature | NR | Colony morphology, α-hemolysis, optochin susceptibility, bile solubility | NR |
|  | United States of America | [48] | Rayon | Stuart’s | NR | Colony morphology, α-hemolysis, optochin susceptibility, bile solubility | 5% Columbia sheep blood agar and selective gentamicin sheep blood agar; incubated at 37° C in 5% to 10% CO_2_ enriched atmosphere |
|  |  | [49] | Calcium-alginate | STGG | Frozen at -70°C, transported on dry ice | Colony morphology, α-hemolysis, optochin susceptibility, bile solubility | Gentamicin-TSA 5% sheep blood agar plate, incubated overnight at 37° C in a 5% CO_2_ enriched atmosphere |
|  |  | [50] | Calcium-alginate | Stuart’s | NR | Colony morphology, optochin susceptibility | Mannitol salt agar, and colistin, nalidixic acid agar containing 5% sheep blood, incubated at 35° C for 48 hours in ambient air |
|  |  | [51] | Calcium-alginate | STGG | NR | α-hemolysis | Blood agar, blood agar with gentamicin, and chocolate agar plates, incubated at 36° C |
|  |  | [52] | Calcium-alginate | STGG | NR | α-hemolysis | Blood agar, blood agar with gentamicin, and chocolate agar plates, incubated at 36° C |
|  |  | [53] | Calcium-alginate | STGG | NR | α-hemolysis | Blood agar, blood agar with gentamicin, and chocolate agar plates, incubated at 36° C |
|  |  | [54] | NR | NA plated immediately | NR | NR | Sheep blood agar plates supplemented with 10 µg/mL gentamicin, incubated at 35° - 37° C in a 5% CO_2_ within 30 minutes of collection |
|  |  | [55] | Calcium-alginate | Plated immediately | NR | NR | Sheep blood agar plates supplemented with 10 µg/mL gentamicin, incubated at 35° - 37° C in a 5% CO_2_ enriched atmosphere within 30 minutes of collection |
|  |  | [56] | Calcium-alginate | STGG | NR | α-hemolysis | Blood agar, blood agar with gentamicin, and chocolate agar plates, incubated at 36° C |
|  |  | [57] | Calcium-alginate | STGG | NR | α-hemolysis | Blood agar, blood agar with gentamicin, and chocolate agar plates, incubated at 36° C |
|  |  | [58] | NR | STGG, transported on ice packs | NR | Colony morphology, optochin susceptibility, bile solubility | Trypticase soy agar supplemented with 5% sheep blood, and 10 µg/mL gentamicin, incubated at 35° - 37° C in a 5% CO_2_ enriched atmosphere for 18 – 24 hours |
|  |  | [59] | NR | NR | NR | NR | NR |
| Eastern Mediterranean | Cyprus | [60] | Rayon | NR | NR | Optochin susceptibility, bile solubility | Blood agar plates, supplemented with 5% defibrinated horse blood, and chocolate agar plates, incubated at 35° C in a 5% CO_2_ enriched atmosphere for 24 hours |
|  | Kingdom of Saudi Arabia | [61] | Dacron | STGG, placed on wet ice, transported within 4 hours | Single colony selected and stored at -80°C | Optochin susceptibility, bile solubility, with *lytA*, *sodA*, and *16S* rDNA sequencing where ambiguous | Trypticase soy agar and 5% sheep blood with nalidixic acid and colistin; isolates identified as *S. pneumoniae* in Saudi Arabia were transported to Germany and replated on 5% sheep blood agar to test for optochin susceptibility and bile-solubility |
| Europe | France | [62] | Cotton | Transport medium (no other details provided) | NR | Gram staining, colony morphology | Swirled in 200 L brain-heart infusion, 20 L smeared onto Columbia blood agar, supplemented with colistin and nalidixic acid |
|  | France and the Kingdom of Saudi Arabia | [63] | NR | NR | NR | *lytA* qPCR positive (threshold cycle value < 35) | Real-time qPCR targeting the *lytA* gene in samples |
|  | Greenland | [64] | Nylon (flocked) | STGG | 20°C < 3 weeks | Colony morphology, α-hemolysis, optochin susceptibility, bile solubility, Quellung reaction | 5% horse blood agar; added 50 µl of the sample to a 2 ml serum ox broth and incubated at 37° C in CO_2_ enriched atmosphere for 24 hours, before plating again |
|  | Italy | [65] | Calcium-alginate | STGG | -80°C | qPCR targeting the *lytA* gene | Broth enrichment - 200µl aliquots added to 5ml supplemented Todd-Hewitt broth, incubated at 37° C for 4 hours in a CO_2_ incubator |
|  |  |  |  |  |  |  |  |
|  |  | [66] | Nylon (flocked) | STGG | -80°C | Colony morphology, optochin susceptibility, bile solubility | Columbia agar with 5% sheep blood, supplemented with 5 µg/mL gentamicin, incubated overnight at 35° C in a 5% CO_2_ enriched atmosphere |
|  |  |  |  |  |  |  |  |
|  |  |  |  |  |  |  |  |
|  |  | [67] | Nylon (flocked) | NR | NR | Colony morphology | Columbia agar with 5% sheep blood, supplemented with 5 µg/ml gentamicin |
|  |  | [68] | Nylon (flocked) | Liquid Amies | Stored at 4°C - 8°C and plated within 48 hours | Gram staining, α-hemolysis, optochin susceptibility, bile solubility | Columbia horse blood agar containing colistin and nalidixic acid; incubated overnight at 35° C in air with 5% CO_2_ |
|  |  |  |  |  |  |  |  |
|  | Portugal | [69] | Cotton and viscose | Stuart’s then kept at room temperature for 2 – 30 hours | Swabs and pure cultures at -80°C in STGG | Colony morphology, optochin susceptibility, bile solubility | Blood agar with gentamicin, incubated at 37° C in anaerobic jars for 24 hours |
|  |  |  |  |  |  |  |  |
|  | Spain | [70] | NR | Amies | Transport NR; stored at -80°C | Colony morphology, α-hemolysis, optochin susceptibility, bile solubility | Blood agar plates with optoquine disk; incubated at 37° C with 5% CO_2_ enriched atmosphere for 24 hours |
|  | The Netherlands | [71] | NR | Amies | Room temperature | NR | Blood agar supplemented with gentamicin |
|  |  | [72] | Dacron | Amies | NR | NR | Blood agar plates with 5% sheep blood |
|  |  | [73] | Cotton | Modified Amies | Room temperature | Colony morphology | Chocolate agar, Haemophilus chocolate agar, and 5% sheep blood agar, with and without 5 mg/l gentamicin; incubated at 35° C for 48 hours (the blood agar plate without gentamicin aerobically; blood agar plate with gentamicin and chocolate agar with  CO_2_ enrichment |
| Europe and Eastern Mediterranean | Israel and West Bank and Gaza^c^ | [74] | Rayon | Amies | Transported within 24 hours | Colony morphology, α-hemolysis, optochin susceptibility | Tryptic-soy agar plates with sheep blood and 5 µg/ml gentamicin, incubated overnight at 35°C in a 5% CO_2_ enriched atmosphere |
|  |  |  |  |  |  |  |  |
| Western Pacific | Australia | [75] | Cotton | Skimmed milk glucose glycerol broth | -20°C for transport to the laboratory; -70°C in laboratory | Colony morphology, optochin susceptibility | Bacitracin containing horse blood agar and chocolate agar; incubated overnight at 37° C in a 5% CO_2_ |
|  |  |  |  |  |  |  |  |
|  |  |  |  |  |  |  |  |
|  |  |  |  |  |  |  |  |
|  |  |  |  |  |  |  |  |
|  |  |  |  |  |  |  |  |
|  |  |  |  |  |  |  |  |
|  |  |  |  |  |  |  |  |
|  | Hong Kong, British Protectorate | [76] | Calcium-alginate | Amies; sent to the microbiological laboratory within 2 hours, transferred to tubes containing 1.0 ml of 0.9% sodium chloride | NR | Colony morphology, α-hemolysis, optochin susceptibility, bile solubility | Columbia agar base with 5% chocolate horse blood, incubated at 37° C in a 5% CO_2_ for 24 – 48 hours |
|  | Hong Kong SAR, China | [77] | Flocked (type NR) | STGG | Samples at 4°C for transport within 4 hours; isolates at -70°C in brain-heart infusion for longer storage | Gram staining, colony morphology, optochin susceptibility, bile solubility | 2.5ml Todd Hewitt broth with 5% yeast extract, 0.5 ml rabbit serum; inoculated onto 5% horse blood agar, incubated at 37° C in a 5% CO_2_ enriched atmosphere for 18-24 hours |
|  |  |  |  |  |  |  |  |
|  |  |  |  |  |  |  |  |
|  |  |  |  |  |  |  |  |
|  | Japan | [78] | NR | NR | *S. pneumonia* isolates stored at -80°C | NR | NR |
|  |  | [79] | NR | Amies with charcoal | NR | Colony morphology, optochin susceptibility; with detection by *lyt*A real-time PCR detection where ambiguous | Medium containing 5% sheep blood, incubated overnight at 35° C |
|  |  | [80] | Cotton | NR | Via a swab transport system | α-hemolysis, optochin susceptibility | Columbia agar plates with 5% sheep; cultured at 37° C in a 5% CO_2_ overnight |
|  | Taiwan (China) | [81] | NR | Amies | -70°C | Colony morphology, optochin susceptibility, bile solubility | Blood agar plates with 5% sheep blood |
|  |  | [82] | NR | Transport medium (no details provided) | NR | NR | NR |

Abbreviations: °C – degrees Celsius; CO_2_ – carbon dioxide; NR – not reported; PCR – polymerase chain reaction; qPCR – quantitative polymerase chain reaction; rDNA – ribosomal deoxyribonucleic acid; SAR – special administrative region; STGG – skim milk, tryptone, glucose, and glycerine; WHO - World Health Organization. Footnotes: ^a^ As per countries listed under WHO regional offices[83]; ^b^ World Bank Income status at the time the study was undertaken[84]; ^c^ This study was undertaken in high-income Israel (WHO European region) and lower-middle-income West Bank and Gaza (WHO Eastern Mediterranean regions)[74, 83, 84]

# References

1. Assefa A, Gelaw B, Shiferaw Y, Tigabu Z. Nasopharyngeal carriage and antimicrobial susceptibility pattern of *Streptococcus pneumoniae* among pediatric outpatients at Gondar University Hospital, North West Ethiopia. PEDN. 2013;54(5):315-21. doi: <https://dx.doi.org/10.1016/j.pedneo.2013.03.017>. PubMed PMID: 23680262.

2. Gebre T, Tadesse M, Aragaw D, Feye D, Beyene HB, Seyoum D, et al. Nasopharyngeal carriage and antimicrobial susceptibility patterns of *Streptococcus pneumoniae* among children under five in Southwest Ethiopia. Children. 2017;4(4). doi: 10.3390/children4040027. PubMed PMID: 28422083.

3. Wada FW, Tufa EG, Berheto TM, Solomon FB. Nasopharyngeal carriage of Streptococcus pneumoniae and antimicrobial susceptibility pattern among school children in South Ethiopia: post-vaccination era. BMC research notes. 2019;12(1):306. doi: <https://dx.doi.org/10.1186/s13104-019-4330-0>.

4. Haile AA, Gidebo DD, Ali MM. Colonization rate of Streptococcus pneumoniae, its associated factors and antimicrobial susceptibility pattern among children attending kindergarten school in Hawassa, southern Ethiopia. BMC Res Notes. 2019;12(1):344. Epub 2019/06/19. doi: 10.1186/s13104-019-4376-z. PubMed PMID: 31208447; PubMed Central PMCID: PMCPMC6580519.

5. Abdullahi O, Nyiro J, Lewa P, Slack M, Scott JA. The descriptive epidemiology of *Streptococcus pneumoniae* and *Haemophilus influenzae* nasopharyngeal carriage in children and adults in Kilifi district, Kenya. Ped Infect Dis J. 2008;27(1):59-64. doi: <https://dx.doi.org/10.1097/INF.0b013e31814da70c>. PubMed PMID: 18162940.

6. Abdullahi O, Karani A, Tigoi CC, Mugo D, Kungu S, Wanjiru E, et al. The prevalence and risk factors for pneumococcal colonization of the nasopharynx among children in Kilifi District, Kenya. PLoS One. 2012;7(2):e30787. doi: <https://dx.doi.org/10.1371/journal.pone.0030787>. PubMed PMID: 22363489.

7. Ousmane S, Diallo BA, Ouedraogo R, Sanda AA, Soussou AM, Collard JM. Serotype distribution and antimicrobial sensitivity profile of *Streptococcus pneumoniae c*arried in healthy toddlers before PCV13 introduction in Niamey, Niger. PLoS One. 2017;12(1):e0169547. doi: 10.1371/journal.pone.0169547. PubMed PMID: 28103262.

8. Bojang A, Jafali J, Egere U, Hill P, Antonio M, Jeffries D. Seasonality of pneumococcal nasopharyngeal carriage in rural Gambia determined within the context of a cluster randomized pneumococcal vaccine trial. PLoS One. 2015;10(7):13. PubMed PMID: CN-01130937.

9. Usuf E, Badji H, Bojang A, Jarju S, Ikumapayi UN, Antonio M, et al. Pneumococcal carriage in rural Gambia prior to the introduction of pneumococcal conjugate vaccine: a population-based survey. Trop Med Int Health. 2015;20(7):871-9. doi: <https://dx.doi.org/10.1111/tmi.12505>. PubMed PMID: 25778937.

10. Usuf E, Bojang A, Camara B, Jagne I, Oluwalana C, Bottomley C, et al. Maternal pneumococcal nasopharyngeal carriage and risk factors for neonatal carriage after the introduction of pneumococcal conjugate vaccines in The Gambia. Clin Microbiol Infect. 2018;24(4):389-95. Epub 2017/07/27. doi: 10.1016/j.cmi.2017.07.018. PubMed PMID: 28743545.

11. Hill PC, Akisanya A, Sankareh K, Cheung YB, Saaka M, Lahai G, et al. Nasopharyngeal carriage of *Streptococcus pneumoniae* in Gambian villagers. Clin Infect Dis 2006;43(6):673-9. doi: <https://dx.doi.org/10.1086/506941>. PubMed PMID: 16912937.

12. le Polain de Waroux O, Flasche S, Kucharski AJ, Langendorf C, Ndazima D, Mwanga-Amumpaire J, et al. Identifying human encounters that shape the transmission of *Streptococcus pneumoniae* and other acute respiratory infections. Epidemics. 2018;25:72-9. Epub 2018/07/29. doi: 10.1016/j.epidem.2018.05.008. PubMed PMID: 30054196; PubMed Central PMCID: PMCPMC6227246.

13. Lindstrand A, Kalyango J, Alfven T, Darenberg J, Kadobera D, Bwanga F, et al. Pneumococcal carriage in children under five years in Uganda-will present pneumococcal conjugate vaccines be appropriate? PLoS One. 2016;11(11):e0166018. doi: 10.1371/journal.pone.0166018. PubMed PMID: 27829063.

14. Nackers F, Cohuet S, le Polain de Waroux O, Langendorf C, Nyehangane D, Ndazima D, et al. Carriage prevalence and serotype distribution of *Streptococcus pneumoniae* prior to 10-valent pneumococcal vaccine introduction: A population-based cross-sectional study in South Western Uganda, 2014. Vaccine. 2017;35(39):5271-7. Epub 2017/08/09. doi: 10.1016/j.vaccine.2017.07.081. PubMed PMID: 28784282; PubMed Central PMCID: PMCPMC6616034.

15. Coles CL, Kanungo R, Rahmathullah L, Thulasiraj RD, Katz J, Santosham M, et al. Pneumococcal nasopharyngeal colonization in young South Indian infants. Ped Infect Dis J. 2001;20(3):289-95. PubMed PMID: 11303832.

16. Coles CL, Sherchand JB, Khatry SK, Katz J, Leclerq SC, Mullany LC, et al. Nasopharyngeal carriage of *S. pneumoniae* among young children in rural Nepal. Trop Med Int Health. 2009;14(9):1025-33. Epub 2009/07/01. doi: 10.1111/j.1365-3156.2009.02331.x. PubMed PMID: 19563428; PubMed Central PMCID: PMCPMC2770711.

17. Nguyen HAT, Fujii H, Vu HTT, Parry CM, Dang AD, Ariyoshi K, et al. An alarmingly high nasal carriage rate of Streptococcus pneumoniae serotype 19F non-susceptible to multiple beta-lactam antimicrobials among Vietnamese children. BMC Infect Dis. 2019;19(1):241. Epub 2019/03/15. doi: 10.1186/s12879-019-3861-2. PubMed PMID: 30866853; PubMed Central PMCID: PMCPMC6416861.

18. Uddén F, Filipe M, Slotved HC, Yamba-Yamba L, Fuursted K, Pintar Kuatoko P, et al. Pneumococcal carriage among children aged 4 - 12 years in Angola 4 years after the introduction of a pneumococcal conjugate vaccine. Vaccine. 2020;38(50):7928-37. Epub 2020/11/05. doi: 10.1016/j.vaccine.2020.10.060. PubMed PMID: 33143954.

19. Adetifa IM, Antonio M, Okoromah CA, Ebruke C, Inem V, Nsekpong D, et al. Pre-vaccination nasopharyngeal pneumococcal carriage in a Nigerian population: epidemiology and population biology. PLoS One. 2012;7(1):e30548. doi: <https://dx.doi.org/10.1371/journal.pone.0030548>. PubMed PMID: 22291984.

20. Inverarity D, Diggle M, Ure R, Johnson P, Altstadt P, Mitchell T, et al. Molecular epidemiology and genetic diversity of pneumococcal carriage among children in Beni State, Bolivia. Trans R Soc Trop Med Hyg. 2011;105(8):445-51. doi: <https://dx.doi.org/10.1016/j.trstmh.2011.04.013>. PubMed PMID: 21714978.

21. Cardozo DM, Nascimento-Carvalho CM, Andrade AL, Silvany-Neto AM, Daltro CH, Brandao MA, et al. Prevalence and risk factors for nasopharyngeal carriage of *Streptococcus pneumoniae* among adolescents. J Med Microbiol. 2008;57(Pt 2):185-9. doi: <https://dx.doi.org/10.1099/jmm.0.47470-0>. PubMed PMID: 18201984.

22. Regev-Yochay G, Raz M, Dagan R, Porat N, Shainberg B, Pinco E, et al. Nasopharyngeal carriage of *Streptococcus pneumoniae* by adults and children in community and family settings. Clin Infect Dis 2004;38(5):632-9. doi: <https://dx.doi.org/10.1086/381547>. PubMed PMID: 14986245.

23. Farida H, Severin JA, Gasem MH, Keuter M, Wahyono H, van den Broek P, et al. Nasopharyngeal carriage of *Streptococcus pneumoniae* in pneumonia-prone age groups in Semarang, Java Island, Indonesia. PLoS One. 2014;9(1):e87431. doi: <https://dx.doi.org/10.1371/journal.pone.0087431>. PubMed PMID: 24498104.

24. Murad C, Dunne EM, Sudigdoadi S, Fadlyana E, Tarigan R, Pell CL, et al. Pneumococcal carriage, density, and co-colonization dynamics: A longitudinal study in Indonesian infants. IJID. 2019;86:73-81. doi: <https://dx.doi.org/10.1016/j.ijid.2019.06.024>.

25. Hu J, Sun X, Huang Z, Wagner AL, Carlson B, Yang J, et al. *Streptococcus pneumoniae* and *Haemophilus influenzae* type b carriage in Chinese children aged 12-18 months in Shanghai, China: a cross-sectional study. BMC Infect Dis. 2016;16:149. doi: <https://dx.doi.org/10.1186/s12879-016-1485-3>. PubMed PMID: 27080523.

26. Russell FM, Carapetis JR, Ketaiwai S, Kunabuli V, Taoi M, Biribo S, et al. Pneumococcal nasopharyngeal carriage and patterns of penicillin resistance in young children in Fiji. Ann Trop Paediatr 2006;26(3):187-97. doi: <https://dx.doi.org/10.1179/146532806X120273>. PubMed PMID: 16925955.

27. Dunne EM, Choummanivong M, Neal EFG, Stanhope K, Nguyen CD, Xeuatvongsa A, et al. Factors associated with pneumococcal carriage and density in infants and young children in Laos PDR. PLoS One. 2019;14(10):e0224392. doi: <https://dx.doi.org/10.1371/journal.pone.0224392>.

28. von Mollendorf C, Dunne EM, La Vincente S, Ulziibayar M, Suuri B, Luvsantseren D, et al. Pneumococcal carriage in children in Ulaanbaatar, Mongolia before and one year after the introduction of the 13-valent pneumococcal conjugate vaccine. Vaccine. 2019;37(30):4068-75. doi: <https://dx.doi.org/10.1016/j.vaccine.2019.05.078>.

29. Shiri T, Nunes MC, Adrian PV, Van Niekerk N, Klugman KP, Madhi SA. Interrelationship of *Streptococcus pneumoniae*, *Haemophilus influenzae* and *Staphylococcus aureus* colonization within and between pneumococcal-vaccine naive mother-child dyads. BMC Infect Dis. 2013;13:483. doi: <https://dx.doi.org/10.1186/1471-2334-13-483>. PubMed PMID: 24134472.

30. Vanker A, Nduru PM, Barnett W, Dube FS, Sly PD, Gie RP, et al. Indoor air pollution and tobacco smoke exposure: impact on nasopharyngeal bacterial carriage in mothers and infants in an African birth cohort study. ERJ Open Res. 2019;5(1). Epub 2019/02/12. doi: 10.1183/23120541.00052-2018. PubMed PMID: 30740462; PubMed Central PMCID: PMCPMC6360211.

31. Skosana Z, Von Gottberg A, Olorunju S, Mohale T, Du Plessis M, Adams T, et al. Non-vaccine serotype pneumococcal carriage in healthy infants in South Africa following introduction of the 13-valent pneumococcal conjugate vaccine. S Afr Med J. 2021;111(2):143-8. Epub 2021/05/05. doi: 10.7196/SAMJ.2021.v111i2.14626. PubMed PMID: 33944725.

32. Reis JN, Palma T, Ribeiro GS, Pinheiro RM, Ribeiro CT, Cordeiro SM, et al. Transmission of *Streptococcus pneumoniae* in an urban slum community. J Infect Dis. 2008;57(3):204-13. doi: <https://dx.doi.org/10.1016/j.jinf.2008.06.017>. PubMed PMID: 18672297.

33. Menezes AP, Azevedo J, Leite MC, Campos LC, Cunha M, Carvalho Mda G, et al. Nasopharyngeal carriage of *Streptococcus pneumoniae* among children in an urban setting in Brazil prior to PCV10 introduction. Vaccine. 2016;34(6):791-7. doi: <https://dx.doi.org/10.1016/j.vaccine.2015.12.042>. PubMed PMID: 26742946.

34. Neves FPG, Cardoso NT, Snyder RE, Marlow MA, Cardoso CAA, Teixeira LM, et al. Pneumococcal carriage among children after four years of routine 10-valent pneumococcal conjugate vaccine use in Brazil: The emergence of multidrug resistant serotype 6C. Vaccine. 2017;35(21):2794-800. doi: 10.1016/j.vaccine.2017.04.019. PubMed PMID: 28431817.

35. Toledo ME, Casanova MF, Linares-Perez N, Garcia-Rivera D, Torano Peraza G, Barcos Pina I, et al. Prevalence of pneumococcal nasopharyngeal carriage among children 2-18 months of age: baseline study pre-introduction of pneumococcal vaccination in Cuba. Ped Infect Dis J. 2017;36(1):e22-e8. doi: 10.1097/inf.0000000000001341. PubMed PMID: 27649366.

36. Rivera-Olivero IA, del Nogal B, Sisco MC, Bogaert D, Hermans PW, de Waard JH. Carriage and invasive isolates of *Streptococcus pneumoniae* in Caracas, Venezuela: the relative invasiveness of serotypes and vaccine coverage. Eur J Clin Microbiol Infect Dis. 2011;30(12):1489-95. doi: <https://dx.doi.org/10.1007/s10096-011-1247-5>. PubMed PMID: 21499972.

37. Verhagen LM, Hermsen M, Rivera-Olivero IA, Sisco MC, de Jonge MI, Hermans PW, et al. Nasopharyngeal carriage of respiratory pathogens in Warao Amerindians: significant relationship with stunting. Trop Med Int Health. 2017;22(4):407-14. doi: 10.1111/tmi.12835. PubMed PMID: 28072501.

38. Karami M, Hosseini SM, Hashemi SH, Ghiasvand S, Zarei O, Safari N, et al. Prevalence of nasopharyngeal carriage of Streptococcus pneumoniae in children 7 to 14 years in 2016: A survey before pneumococcal conjugate vaccine introduction in Iran. Human vaccines & immunotherapeutics. 2019;15(9):2178-82. doi: <https://dx.doi.org/10.1080/21645515.2018.1539601>.

39. Korona-Glowniak I, Malm A. Characteristics of *Streptococcus pneumoniae* strains colonizing upper respiratory tract of healthy preschool children in Poland. Sci World J. 2012.

40. Ozdemir B, Beyazova U, Camurdan AD, Sultan N, Ozkan S, Sahin F. Nasopharyngeal carriage of *Streptococcus pneumoniae* in healthy Turkish infants. J Infect Dis. 2008;56(5):332-9. doi: <https://dx.doi.org/10.1016/j.jinf.2008.02.010>. PubMed PMID: 18377994.

41. Uzuner A, Ilki A, Akman M, Gundogdu E, Erbolukbas R, Kokacya O, et al. Nasopharyngeal carriage of penicillin-resistant *Streptococcus pneumoniae* in healthy children. Turk J Pediatr 2007;49(4):370-8. Epub 2008/02/06. PubMed PMID: 18246737.

42. Ozdemir H, Ciftci E, Durmaz R, Guriz H, Aysev AD, Karbuz A, et al. Risk factors for nasopharyngeal carriage of *Streptococcus pneumoniae* in healthy Turkish children after the addition of heptavalent pneumococcal conjugate vaccine (PCV7) to the national vaccine schedule. Turk J Pediatr. 2013;55(6):575-83. Epub 2014/03/01. PubMed PMID: 24577974.

43. Arvas A, Cokugras H, Gur E, Gonullu N, Taner Z, Bahar Tokman H. Pneumococcal nasopharyngeal carriage in young healthy children after pneumococcal conjugate vaccine in Turkey. Balkan Med J. 2017. doi: 10.4274/balkanmedj.2016.1256. PubMed PMID: 28443585.

44. Neal EFG, Nguyen C, Ratu FT, Matanitobua S, Dunne EM, Reyburn R, et al. A comparison of pneumococcal nasopharyngeal carriage in very young Fijian infants born by vaginal or Cesarean delivery. JAMA Netw Open. 2019;2(10):e1913650. Epub 2019/10/19. doi: 10.1001/jamanetworkopen.2019.13650. PubMed PMID: 31626319; PubMed Central PMCID: PMCPMC6813584.

45. Neal EFG, Flasche S, Nguyen CD, Ratu FT, Dunne EM, Koyamaibole L, et al. Associations between ethnicity, social contact, and pneumococcal carriage three years post-PCV10 in Fiji. Vaccine. 2020;38(2):202-11. doi: <https://dx.doi.org/10.1016/j.vaccine.2019.10.030>.

46. Neal EFG, Nguyen CD, Ratu FT, Dunne EM, Kama M, Ortika BD, et al. Factors associated with pneumococcal carriage and density in children and adults in Fiji, using four cross-sectional surveys. PLoS One. 2020;15(4):e0231041. doi: <https://dx.doi.org/10.1371/journal.pone.0231041>.

47. Ricketson LJ, Wood ML, Vanderkooi OG, MacDonald JC, Martin IE, Demczuk WH, et al. Trends in asymptomatic nasopharyngeal colonization with *Streptococcus pneumoniae* after introduction of the 13-valent pneumococcal conjugate vaccine in Calgary, Canada. Ped Infect Dis J. 2014;33(7):724-30. doi: <https://dx.doi.org/10.1097/INF.0000000000000267>. PubMed PMID: 24463806.

48. Samore MH, Magill MK, Alder SC, Severina E, Morrison-De Boer L, Lyon JL, et al. High rates of multiple antibiotic resistance in *Streptococcus pneumoniae* from healthy children living in isolated rural communities: association with cephalosporin use and intrafamilial transmission. Pediatrics. 2001;108(4):856-65. PubMed PMID: 11581436.

49. Millar EV, O'Brien KL, Zell ER, Bronsdon MA, Reid R, Santosham M. Nasopharyngeal carriage of *Streptococcus pneumoniae* in Navajo and White Mountain Apache children before the introduction of pneumococcal conjugate vaccine. Ped Infect Dis J. 2009;28(8):711-6. Epub 2009/07/14. doi: 10.1097/INF.0b013e3181a06303. PubMed PMID: 19593248.

50. Cheng Immergluck L, Kanungo S, Schwartz A, McIntyre A, Schreckenberger PC, Diaz PS. Prevalence of *Streptococcus pneumoniae* and *Staphylococcus aureus* nasopharyngeal colonization in healthy children in the United States. Epidemiol Infect. 2004;132(2):159-66. PubMed PMID: 15061489.

51. Finkelstein JA, Huang SS, Daniel J, Rifas-Shiman SL, Kleinman K, Goldmann D, et al. Antibiotic-resistant *Streptococcus pneumoniae* in the heptavalent pneumococcal conjugate vaccine era: predictors of carriage in a multicommunity sample. Pediatrics. 2003;112(4):862-9. PubMed PMID: 14523178.

52. Huang SS, Finkelstein JA, Rifas-Shiman SL, Kleinman K, Platt R. Community-level predictors of pneumococcal carriage and resistance in young children. Am J Epidemiol. 2004;159(7):645-54. PubMed PMID: 15033642.

53. Huang SS, Hinrichsen VL, Stevenson AE, Rifas-Shiman SL, Kleinman K, Pelton SI, et al. Continued impact of pneumococcal conjugate vaccine on carriage in young children. Pediatrics. 2009;124(1):e1-11. doi: <https://dx.doi.org/10.1542/peds.2008-3099>. PubMed PMID: 19564254.

54. Moore MR, Hyde TB, Hennessy TW, Parks DJ, Reasonover AL, Harker-Jones M, et al. Impact of a conjugate vaccine on community-wide carriage of nonsusceptible *Streptococcus pneumoniae* in Alaska. J Infect Dis. 2004;190(11):2031-8. doi: <https://dx.doi.org/10.1086/425422>. PubMed PMID: 15529269.

55. Park SY, Moore MR, Bruden DL, Hyde TB, Reasonover AL, Harker-Jones M, et al. Impact of conjugate vaccine on transmission of antimicrobial-resistant *Streptococcus pneumoniae* among Alaskan children. Ped Infect Dis J. 2008;27(4):335-40. doi: <https://dx.doi.org/10.1097/INF.0b013e318161434d>. PubMed PMID: 18316986.

56. Lee GM, Kleinman K, Pelton SI, Hanage W, Huang SS, Lakoma M, et al. Impact of 13-Valent Pneumococcal Conjugate Vaccination on *Streptococcus pneumoniae* Carriage in Young Children in Massachusetts. J Pediatric Infect Dis Soc. 2014;3(1):23-32. doi: 10.1093/jpids/pit057. PubMed PMID: 24567842.

57. Hsu KK, Rifas-Shiman SL, Shea KM, Kleinman KP, Lee GM, Lakoma M, et al. Do community-level predictors of pneumococcal carriage continue to play a role in the conjugate vaccine era? Epidemiol Infect. 2014;142(2):379-87. doi: <https://dx.doi.org/10.1017/S0950268813000794>. PubMed PMID: 23731707.

58. Reisman J, Rudolph K, Bruden D, Hurlburt D, Bruce MG, Hennessy T. Risk factors for pneumococcal colonization of the nasopharynx in Alaska native adults and children. J Pediatric Infect Dis Soc. 2014;3(2):104-11. doi: 10.1093/jpids/pit069. PubMed PMID: 26625363.

59. Wroe PC, Lee GM, Finkelstein JA, Pelton SI, Hanage WP, Lipsitch M, et al. Pneumococcal carriage and antibiotic resistance in young children before 13-valent conjugate vaccine. Ped Infect Dis J. 2012;31(3):249-54. doi: <https://dx.doi.org/10.1097/INF.0b013e31824214ac>. PubMed PMID: 22173142.

60. Koliou MG, Andreou K, Lamnisos D, Lavranos G, Iakovides P, Economou C, et al. Risk factors for carriage of *Streptococcus pneumoniae* in children. BMC Pediatr. 2018;18(1):144. Epub 2018/04/28. doi: 10.1186/s12887-018-1119-6. PubMed PMID: 29699525; PubMed Central PMCID: PMCPMC5921789.

61. Memish ZA, Assiri A, Almasri M, Alhakeem RF, Turkestani A, Al Rabeeah AA, et al. Impact of the Hajj on pneumococcal transmission. Clin Microbiol Infect. 2015;21(1):77.e11-8. doi: <https://dx.doi.org/10.1016/j.cmi.2014.07.005>. PubMed PMID: 25636939.

62. Cohen R, Levy C, Bonnet E, Thollot F, Boucherat M, Fritzell B, et al. Risk factors for serotype 19A carriage after introduction of 7-valent pneumococcal vaccination. BMC Infect Dis. 2011;11:95. doi: <https://dx.doi.org/10.1186/1471-2334-11-95>. PubMed PMID: 21501471.

63. Hoang VT, Dao TL, Ly TDA, Belhouchat K, Chaht KL, Gaudart J, et al. The dynamics and interactions of respiratory pathogen carriage among French pilgrims during the 2018 Hajj. Emerg Microbes Infect. 2019;8(1):1701-10. Epub 2019/11/22. doi: 10.1080/22221751.2019.1693247. PubMed PMID: 31749410; PubMed Central PMCID: PMCPMC6882464.

64. Navne JE, Borresen ML, Slotved HC, Andersson M, Melbye M, Ladefoged K, et al. Nasopharyngeal bacterial carriage in young children in Greenland: a population at high risk of respiratory infections. Epidemiol Infect. 2016;144(15):3226-36. doi: 10.1017/s0950268816001461. PubMed PMID: 27405603.

65. Ansaldi F, de Florentiis D, Canepa P, Zancolli M, Martini M, Orsi A, et al. Carriage of *Streptococcus pneumoniae* 7 years after implementation of vaccination program in a population with very high and long-lasting coverage, Italy. Vaccine. 2012;30(13):2288-94. doi: <https://dx.doi.org/10.1016/j.vaccine.2012.01.067>. PubMed PMID: 22306795.

66. Camilli R, Daprai L, Cavrini F, Lombardo D, D'Ambrosio F, Del Grosso M, et al. Pneumococcal carriage in young children one year after introduction of the 13-valent conjugate vaccine in Italy. PLoS One. 2013;8(10):e76309. doi: <https://dx.doi.org/10.1371/journal.pone.0076309>. PubMed PMID: 24124543.

67. Camilli R, Vescio MF, Giufre M, Daprai L, Garlaschi ML, Cerquetti M, et al. Carriage of *Haemophilus influenzae* is associated with pneumococcal vaccination in Italian children. Vaccine. 2015;33(36):4559-64. doi: <https://dx.doi.org/10.1016/j.vaccine.2015.07.009>. PubMed PMID: 26190092.

68. Zuccotti G, Mameli C, Daprai L, Garlaschi ML, Dilillo D, Bedogni G, et al. Serotype distribution and antimicrobial susceptibilities of nasopharyngeal isolates of *Streptococcus pneumoniae* from healthy children in the 13-valent pneumococcal conjugate vaccine era. Vaccine. 2014;32(5):527-34. doi: 10.1016/j.vaccine.2013.12.003. PubMed PMID: 24342249.

69. Almeida ST, Nunes S, Santos Paulo AC, Valadares I, Martins S, Breia F, et al. Low prevalence of pneumococcal carriage and high serotype and genotype diversity among adults over 60 years of age living in Portugal. PLoS One. 2014;9(3):e90974. doi: <https://dx.doi.org/10.1371/journal.pone.0090974>. PubMed PMID: 24604030.

70. Alfayate Miguélez S, Yague Guirao G, Menasalvas Ruíz AI, Sanchez-Solís M, Domenech Lucas M, González Camacho F, et al. Impact of pneumococcal vaccination in the nasopharyngeal carriage of *Streptococcus pneumoniae* in healthy children of the Murcia Region in Spain. Vaccines. 2020;9(1). Epub 2021/01/01. doi: 10.3390/vaccines9010014. PubMed PMID: 33379235; PubMed Central PMCID: PMCPMC7823743.

71. Bogaert D, van Belkum A, Sluijter M, Luijendijk A, de Groot R, Rumke HC, et al. Colonisation by *Streptococcus pneumoniae* and *Staphylococcus aureus* in healthy children. Lancet. 2004;363(9424):1871-2. doi: <https://dx.doi.org/10.1016/S0140-6736(04)16357-5>. PubMed PMID: 15183627.

72. Labout JA, Duijts L, Arends LR, Jaddoe VW, Hofman A, de Groot R, et al. Factors associated with pneumococcal carriage in healthy Dutch infants: the generation R study. J Pediatr. 2008;153(6):771-6. doi: <https://dx.doi.org/10.1016/j.jpeds.2008.05.061>. PubMed PMID: 18621390.

73. Gils E, Veenhoven R, Rodenburg G, Hak E, Sanders E. Effect of 7-valent pneumococcal conjugate vaccine on nasopharyngeal carriage with *Haemophilus influenzae* and *Moraxella catarrhalis* in a randomized controlled trial. Vaccine. 2011;29(44):7595-8. doi: 10.1016/j.vaccine.2011.08.049. PubMed PMID: CN-00806054.

74. Daana M, Rahav G, Hamdan A, Thalji A, Jaar F, Abdeen Z, et al. Measuring the effects of pneumococcal conjugate vaccine (PCV7) on *Streptococcus pneumoniae* carriage and antibiotic resistance: the Palestinian-Israeli Collaborative Research (PICR). Vaccine. 2015;33(8):1021-6. doi: 10.1016/j.vaccine.2015.01.003. PubMed PMID: 25593104.

75. Mackenzie GA, Leach AJ, Carapetis JR, Fisher J, Morris PS. Epidemiology of nasopharyngeal carriage of respiratory bacterial pathogens in children and adults: cross-sectional surveys in a population with high rates of pneumococcal disease. BMC Infect Dis. 2010;10:304. doi: <https://dx.doi.org/10.1186/1471-2334-10-304>. PubMed PMID: 20969800.

76. Sung RY, Ling JM, Fung SM, Oppenheimer SJ, Crook DW, Lau JT, et al. Carriage of *Haemophilus influenzae* and *Streptococcus pneumoniae* in healthy Chinese and Vietnamese children in Hong Kong. Acta Paediatr. 1995;84(11):1262-7. PubMed PMID: 8580623.

77. Chan KC, Subramanian R, Chong P, Nelson EA, Lam HS, Li AM, et al. Pneumococcal carriage in young children after introduction of PCV13 in Hong Kong. Vaccine. 2016;34(33):3867-74. doi: 10.1016/j.vaccine.2016.05.047. PubMed PMID: 27265449.

78. Otsuka T, Chang B, Shirai T, Iwaya A, Wada A, Yamanaka N, et al. Individual risk factors associated with nasopharyngeal colonization with *Streptococcus pneumoniae* and *Haemophilus influenzae*: a Japanese birth cohort study. Ped Infect Dis J. 2013;32(7):709-14. doi: <https://dx.doi.org/10.1097/INF.0b013e31828701ea>. PubMed PMID: 23411622.

79. Ueno M, Ishii Y, Tateda K, Anahara Y, Ebata A, Iida M, et al. Prevalence and risk factors of nasopharyngeal carriage of *Streptococcus pneumoniae* in healthy children in Japan. Jpn J Infect Dis. 2013;66(1):22-5. PubMed PMID: 23429080.

80. Chang B, Akeda H, Nakamura Y, Hamabata H, Ameku K, Toma T, et al. Impact of thirteen-valent pneumococcal conjugate vaccine on nasopharyngeal carriage in healthy children under 24 months in Okinawa, Japan. JIC. 2020;26(5):465-70. doi: <https://dx.doi.org/10.1016/j.jiac.2019.12.009>.

81. Hsieh YC, Chiu CH, Chang KY, Huang YC, Chen CJ, Kuo CY, et al. The impact of the heptavalent pneumococcal conjugate vaccine on risk factors for *Streptococcus pneumoniae* carriage in children. Ped Infect Dis J. 2012;31(9):e163-8. doi: <https://dx.doi.org/10.1097/INF.0b013e31825cb9f9>. PubMed PMID: 22592521.

82. Kuo CY, Hwang KP, Hsieh YC, Cheng CH, Huang FL, Shen YH, et al. Nasopharyngeal carriage of *Streptococcus pneumoniae* in Taiwan before and after the introduction of a conjugate vaccine. Vaccine. 2011;29(32):5171-7. doi: <https://dx.doi.org/10.1016/j.vaccine.2011.05.034>. PubMed PMID: 21621578.

83. World Health Organization. WHO/Who we are/Regional offices 2020 [cited 2020 April 3]. Available from: <https://www.who.int/about/who-we-are/regional-offices>.

84. World Bank Country and Lending Groups [Internet]. World Bank. 2020 [cited 2020 Jan 26].
