# Supplementary material for: Factors associated with pneumococcal nasopharyngeal carriage: A systematic review
Source: PLOS Glob Public Health. 2022 Apr 11;2(4):e0000327. doi: 10.1371/journal.pgph.0000327 (PMC10021834; doi:10.1371/journal.pgph.0000327)
Supplement: S4 Table — (DOCX) [file pgph.0000327.s006.docx]

**S4 Table. Quality assessment of included studies.**

| **Controlled intervention studies** | | | | | | | | | | | | | | | | | | | | | | | | | | | | | | | | | | | | | | | | | | | | | | |
| --- | --- | --- | --- | --- | --- | --- | --- | --- | --- | --- | --- | --- | --- | --- | --- | --- | --- | --- | --- | --- | --- | --- | --- | --- | --- | --- | --- | --- | --- | --- | --- | --- | --- | --- | --- | --- | --- | --- | --- | --- | --- | --- | --- | --- | --- | --- |
| Ref | Described as RCT | | | Randomization adequate | | | | Allocation concealed | | | Participants and providers blinded | | | | Outcome assessors blinded | | | Baseline similarity | | | Overall dropout rate < 20% | | | Differential dropout rate < 15% points | | | High adherence | | | Other interventions avoided | | | | 2003 / 2013 WHO outcome methods [1, 2] | | | Sample size justification | | Outcomes or subgroups specified before analyses | | Intention-to-treat | | | Quality | | |
| [3] | Yes | | | Yes | | | | Yes | | | Yes | | | | Yes | | | Yes | | | Yes | | | Yes | | | Yes | | | Yes | | | | No | | | Yes | | Yes | | Yes | | | Good | | |
| **Cross-sectional and longitudinal studies** | | | | | | | | | | | | | | | | | | | | | | | | | | | | | | | | | | | | | | | | | | | | | | |
| Ref | | Objective stated clearly | | | Population defined | | Participation rate > 50% | | | Subjects from similar populations | | Subjects from similar period | | Inclusion criteria specified | | | Exclusion criteria specified | | | Sample size justification | | | Exposures measures before outcomes | | Sufficient timeframe | | | Exposure levels examined | | | Exposure measures valid | | Exposures assess more than once | | | 2003 / 2013 WHO outcome methods [1, 2] | | Outcome assessors blinded | | | Loss-to-follow < 20% | | Key potential confounding variables adjusted for | | | Quality |
| [4] | | Yes | | | Yes | | Yes | | | Yes | | Yes | | Yes | | | Yes | | | Yes | | | No | | Yes | | | Yes | | | CD | | No | | | CD | | NR | | | NA | | Yes | | | Good |
| [5] | | Yes | | | Yes | | Yes | | | Yes | | Yes | | NR | | | NR | | | No | | | No | | Yes | | | Yes | | | Yes | | Yes | | | Yes | | NR | | | Yes | | Yes | | | Good |
| [6] | | Yes | | | Yes | | Yes | | | Yes | | Yes | | NR | | | NR | | | Yes | | | No | | Yes | | | Yes | | | Yes | | No | | | Yes | | NR | | | NA | | Yes | | | Good |
| [7] | | Yes | | | Yes | | Yes | | | Yes | | Yes | | Yes | | | Yes | | | NR | | | No | | Yes | | | Yes | | | Yes | | Yes | | | No | | NR | | | Yes | | Yes | | | Good |
| [8] | | Yes | | | Yes | | Yes | | | No | | Yes | | Yes | | | NR | | | Yes | | | No | | Yes | | | Yes | | | Yes | | No | | | No | | NR | | | NA | | No | | | Fair |
| [9] | | Yes | | | Yes | | Yes | | | Yes | | Yes | | Yes | | | Yes | | | Yes | | | No | | Yes | | | Yes | | | Yes | | No | | | CD | | NR | | | NA | | CD | | | Good |
| [10] | | Yes | | | Yes | | CD | | | Yes | | Yes | | Yes | | | Yes | | | No | | | Yes | | Yes | | | Yes | | | Yes | | No | | | CD | | NR | | | NA | | Yes | | | Good |
| [11] | | Yes | | | Yes | | NR | | | Yes | | Yes | | Yes | | | NA | | | NR | | | No | | Yes | | | Yes | | | Yes | | No | | | No | | NR | | | NA | | Yes | | | Fair |
| [12] | | Yes | | | Yes | | NR | | | Yes | | Yes | | NR | | | NR | | | NR | | | No | | Yes | | | Yes | | | Yes | | No | | | CD | | NR | | | NA | | CD | | | Fair |
| [13] | | Yes | | | Yes | | Yes | | | Yes | | Yes | | Yes | | | Yes | | | No | | | Yes | | Yes | | | Yes | | | Yes | | No | | | Yes | | NR | | | NR | | Yes | | | Good |
| [14] | | Yes | | | Yes | | CD | | | No | | Yes | | Yes | | | Yes | | | No | | | No | | Yes | | | Yes | | | Yes | | No | | | Yes | | NR | | | NA | | Yes | | | Fair |
| [15] | | Yes | | | Yes | | NR | | | No | | Yes | | Yes | | | Yes | | | NR | | | No | | Yes | | | Yes | | | Yes | | No | | | CD | | NR | | | NA | | Yes | | | Fair |
| [16] | | Yes | | | Yes | | Yes | | | Yes | | Yes | | Yes | | | Yes | | | Yes | | | No | | Yes | | | Yes | | | Yes | | No | | | No | | NR | | | NA | | Yes | | | Good |
| [17] | | Yes | | | Yes | | Yes | | | Yes | | Yes | | Yes | | | Yes | | | Yes | | | No | | Yes | | | Yes | | | Yes | | No | | | Yes | | NR | | | NA | | Yes | | | Good |
| [18] | | Yes | | | Yes | | Yes | | | Yes | | Yes | | NR | | | NR | | | NR | | | No | | Yes | | | Yes | | | Yes | | No | | | No | | NR | | | NA | | CD | | | Fair |
| [19] | | Yes | | | Yes | | Yes | | | Yes | | Yes | | Yes | | | Yes | | | Yes | | | No | | Yes | | | Yes | | | Yes | | No | | | No | | NR | | | NA | | CD | | | Good |
| [20] | | Yes | | | Yes | | NR | | | Yes | | Yes | | Yes | | | Yes | | | No | | | Yes | | Yes | | | Yes | | | Yes | | No | | | No | | NR | | | NA | | Yes | | | Good |
| [21] | | Yes | | | Yes | | Yes | | | Yes | | Yes | | Yes | | | No | | | No | | | No | | Yes | | | No | | | CD | | CD | | | CD | | NR | | | No | | Yes | | | Fair |
| [22] | | Yes | | | Yes | | Yes | | | Yes | | Yes | | NR | | | NR | | | NR | | | No | | Yes | | | Yes | | | Yes | | No | | | CD | | NR | | | NA | | Yes | | | Fair |
| [23] | | Yes | | | Yes | | Yes | | | Yes | | Yes | | Yes | | | Yes | | | NR | | | No | | Yes | | | Yes | | | Yes | | No | | | Yes | | NR | | | NA | | Yes | | | Good |
| [24] | | Yes | | | Yes | | CD | | | Yes | | Yes | | Yes | | | Yes | | | No | | | Yes | | Yes | | | Yes | | | Yes | | No | | | CD | | NR | | | NA | | Yes | | | Good |
| [25] | | Yes | | | Yes | | CD | | | No | | Yes | | Yes | | | Yes | | | No | | | No | | Yes | | | Yes | | | Yes | | No | | | CD | | NR | | | NA | | Yes | | | Fair |
| [26] | | Yes | | | Yes | | Yes | | | Yes | | Yes | | Yes | | | Yes | | | Yes | | | No | | Yes | | | Yes | | | Yes | | No | | | Yes | | NR | | | NA | | Yes | | | Good |
| [27] | | Yes | | | Yes | | Yes | | | Yes | | Yes | | Yes | | | Yes | | | Yes | | | No | | Yes | | | Yes | | | Yes | | Yes | | | CD | | NR | | | NA | | Yes | | | Good |
| [28] | | Yes | | | Yes | | Yes | | | Yes | | Yes | | NR | | | NR | | | No | | | No | | Yes | | | Yes | | | Yes | | No | | | CD | | NR | | | NA | | Yes | | | Fair |
| [29] | | Yes | | | Yes | | Yes | | | Yes | | Yes | | Yes | | | Yes | | | Yes | | | No | | Yes | | | Yes | | | Yes | | Yes | | | CD | | NR | | | NA | | Yes | | | Fair |
| [30] | | Yes | | | Yes | | Yes | | | Yes | | Yes | | Yes | | | Yes | | | No | | | Yes | | Yes | | | Yes | | | Yes | | No | | | CD | | NR | | | NA | | Yes | | | Good |
| [31] | | Yes | | | Yes | | Yes | | | No | | Yes | | CD | | | CD | | | No | | | No | | Yes | | | Yes | | | CD | | No | | | CD | | NR | | | NA | | Yes | | | Fair |
| [32] | | Yes | | | Yes | | Yes | | | Yes | | Yes | | Yes | | | Yes | | | No | | | No | | Yes | | | Yes | | | Yes | | No | | | Yes | | NR | | | NA | | Yes | | | Good |
| [33] | | Yes | | | Yes | | Yes | | | Yes | | Yes | | Yes | | | Yes | | | No | | | No | | CD | | | Yes | | | Yes | | No | | | CD | | NR | | | NA | | Yes | | | Fair |
| [34] | | Yes | | | Yes | | Yes | | | No | | Yes | | Yes | | | Yes | | | No | | | No | | Yes | | | Yes | | | Yes | | Yes | | | CD | | NR | | | NA | | Yes | | | Good |
| [35] | | No | | | No | | NR | | | No | | Yes | | NR | | | NR | | | No | | | No | | Yes | | | Yes | | | Yes | | No | | | Yes | | NR | | | NA | | No | | | Poor |
| [36] | | Yes | | | Yes | | Yes | | | Yes | | Yes | | NR | | | NR | | | NR | | | No | | Yes | | | Yes | | | Yes | | No | | | CD | | NR | | | NA | | CD | | | Fair |
| [37] | | Yes | | | Yes | | CD | | | No | | Yes | | Yes | | | Yes | | | No | | | No | | Yes | | | Yes | | | Yes | | No | | | CD | | NR | | | NA | | Yes | | | Fair |
| [38] | | Yes | | | Yes | | Yes | | | No | | Yes | | Yes | | | Yes | | | No | | | No | | Yes | | | CD | | | Yes | | Yes | | | CD | | NR | | | Yes | | No | | | Fair |
| [39] | | Yes | | | Yes | | Yes | | | No | | Yes | | Yes | | | Yes | | | No | | | No | | Yes | | | CD | | | Yes | | No | | | CD | | NR | | | NA | | Yes | | | Fair |
| [40] | | Yes | | | Yes | | Yes | | | Yes | | Yes | | Yes | | | Yes | | | No | | | Yes | | Yes | | | Yes | | | Yes | | Yes | | | CD | | NR | | | Yes | | Yes | | | Good |
| [41] | | Yes | | | Yes | | CD | | | CD | | Yes | | CD | | | CD | | | No | | | No | | Yes | | | Yes | | | Yes | | Yes | | | CD | | NR | | | NA | | No | | | Fair |
| [42] | | Yes | | | Yes | | Yes | | | Yes | | Yes | | Yes | | | Yes | | | No | | | No | | Yes | | | Yes | | | CD | | No | | | Yes | | NR | | | NA | | CD | | | Fair |
| [43] | | Yes | | | Yes | | Yes | | | No | | No | | No | | | No | | | Yes | | | No | | Yes | | | Yes | | | CD | | Yes | | | Yes | | NR | | | NA | | Yes | | | Fair |
| [44] | | Yes | | | Yes | | Yes | | | Yes | | No | | Yes | | | Yes | | | Yes | | | No | | Yes | | | Yes | | | Yes | | Yes | | | No | | NR | | | CD | | No | | | Good |
| [45] | | Yes | | | Yes | | Yes | | | Yes | | Yes | | Yes | | | Yes | | | Yes | | | No | | Yes | | | CD | | | Yes | | Yes | | | Yes | | NR | | | NA | | Yes | | | Good |
| [46] | | Yes | | | Yes | | CD | | | Yes | | Yes | | Yes | | | Yes | | | No | | | Yes | | Yes | | | Yes | | | Yes | | No | | | No | | NR | | | No | | Yes | | | Good |
| [47] | | Yes | | | Yes | | CD | | | Yes | | No | | Yes | | | Yes | | | No | | | Yes | | Yes | | | Yes | | | Yes | | No | | | Yes | | NR | | | Yes | | Yes | | | Good |
| [48] | | Yes | | | Yes | | Yes | | | Yes | | Yes | | NR | | | NR | | | No | | | No | | Yes | | | Yes | | | Yes | | No | | | CD | | NR | | | NA | | CD | | | Fair |
| [49] | | Yes | | | Yes | | Yes | | | Yes | | Yes | | Yes | | | Yes | | | Yes | | | Yes | | Yes | | | Yes | | | Yes | | Yes | | | Yes | | NR | | | Yes | | Yes | | | Good |
| [50] | | Yes | | | Yes | | Yes | | | Yes | | Yes | | NR | | | NR | | | Yes | | | No | | Yes | | | CD | | | Yes | | No | | | Yes | | NR | | | NA | | CD | | | Fair |
| [51] | | Yes | | | Yes | | Yes | | | Yes | | Yes | | NR | | | NR | | | NR | | | No | | Yes | | | Yes | | | Yes | | No | | | Yes | | NR | | | NA | | Yes | | | Fair |
| [52] | | Yes | | | Yes | | Yes | | | Yes | | Yes | | Yes | | | Yes | | | Yes | | | Yes | | Yes | | | Yes | | | Yes | | No | | | Yes | | NR | | | NA | | Yes | | | Good |
| [53] | | Yes | | | Yes | | Yes | | | Yes | | Yes | | Yes | | | Yes | | | Yes | | | No | | Yes | | | Yes | | | Yes | | No | | | Yes | | Yes | | | NA | | Yes | | | Good |
| [54] | | Yes | | | Yes | | Yes | | | Yes | | Yes | | Yes | | | Yes | | | Yes | | | No | | Yes | | | Yes | | | Yes | | Yes | | | Yes | | Yes | | | NA | | Yes | | | Good |
| [55] | | Yes | | | Yes | | Yes | | | Yes | | Yes | | NR | | | NR | | | No | | | No | | Yes | | | Yes | | | Yes | | No | | | Yes | | NR | | | NA | | Yes | | | Fair |
| [56] | | Yes | | | Yes | | Yes | | | Yes | | Yes | | Yes | | | Yes | | | No | | | No | | Yes | | | Yes | | | Yes | | No | | | CD | | NR | | | NA | | Yes | | | Good |
| [57] | | Yes | | | Yes | | Yes | | | Yes | | Yes | | Yes | | | Yes | | | No | | | Yes | | Yes | | | Yes | | | Yes | | Yes | | | CD | | NR | | | Yes | | Yes | | | Good |
| [58] | | Yes | | | Yes | | CD | | | Yes | | Yes | | Yes | | | Yes | | | No | | | No | | Yes | | | Yes | | | Yes | | No | | | No | | NR | | | NA | | Yes | | | Fair |
| [59] | | Yes | | | Yes | | CD | | | Yes | | Yes | | Yes | | | Yes | | | No | | | No | | Yes | | | Yes | | | Yes | | No | | | No | | NR | | | NA | | CD | | | Fair |
| [60] | | Yes | | | Yes | | CD | | | Yes | | Yes | | Yes | | | Yes | | | No | | | No | | Yes | | | Yes | | | Yes | | No | | | CD | | NR | | | NA | | CD | | | Fair |
| [61] | | Yes | | | Yes | | CD | | | Yes | | Yes | | NR | | | NR | | | No | | | No | | Yes | | | Yes | | | Yes | | Yes | | | CD | | NR | | | NA | | CD | | | Fair |
| [62] | | Yes | | | Yes | | NR | | | Yes | | Yes | | Yes | | | Yes | | | No | | | No | | Yes | | | Yes | | | Yes | | No | | | CD | | NR | | | NA | | Yes | | | Fair |
| [63] | | Yes | | | Yes | | Yes | | | Yes | | Yes | | NR | | | NR | | | No | | | No | | Yes | | | Yes | | | Yes | | No | | | CD | | NR | | | NA | | Yes | | | Fair |
| [64] | | Yes | | | Yes | | Yes | | | Yes | | Yes | | CD | | | CD | | | No | | | No | | Yes | | | Yes | | | Yes | | No | | | CD | | NR | | | NA | | Yes | | | Fair |
| [65] | | Yes | | | Yes | | Yes | | | Yes | | Yes | | Yes | | | Yes | | | No | | | No | | Yes | | | Yes | | | Yes | | Yes | | | No | | NR | | | NA | | Yes | | | Good |
| [66] | | Yes | | | Yes | | CD | | | Yes | | Yes | | Yes | | | Yes | | | No | | | No | | Yes | | | Yes | | | Yes | | No | | | CD | | NR | | | NA | | CD | | | Fair |
| [67] | | Yes | | | Yes | | Yes | | | Yes | | Yes | | NR | | | NR | | | NR | | | No | | Yes | | | Yes | | | Yes | | No | | | No | | NR | | | NA | | Yes | | | Fair |
| [68] | | Yes | | | Yes | | Yes | | | Yes | | Yes | | NR | | | NR | | | No | | | No | | Yes | | | Yes | | | Yes | | No | | | No | | NR | | | NA | | Yes | | | Fair |
| [69] | | Yes | | | Yes | | NR | | | Yes | | Yes | | Yes | | | Yes | | | No | | | Yes | | Yes | | | Yes | | | Yes | | Yes | | | CD | | NR | | | CD | | Yes | | | Good |
| [70] | | Yes | | | Yes | | Yes | | | Yes | | Yes | | NR | | | NR | | | NR | | | No | | Yes | | | Yes | | | Yes | | No | | | CD | | NR | | | Yes | | Yes | | | Good |
| [71] | | Yes | | | Yes | | Yes | | | Yes | | Yes | | NR | | | Yes | | | No | | | No | | Yes | | | Yes | | | Yes | | No | | | CD | | NR | | | NA | | CD | | | Poor |
| [72] | | Yes | | | Yes | | Yes | | | Yes | | Yes | | Yes | | | Yes | | | No | | | No | | Yes | | | Yes | | | Yes | | No | | | Yes | | NR | | | NA | | Yes | | | Good |
| [73] | | Yes | | | Yes | | Yes | | | No | | Yes | | NR | | | NR | | | NR | | | No | | Yes | | | Yes | | | Yes | | No | | | Yes | | NR | | | Yes | | Yes | | | Fair |
| [74] | | Yes | | | Yes | | NR | | | Yes | | Yes | | Yes | | | CD | | | No | | | No | | Yes | | | Yes | | | Yes | | No | | | CD | | NR | | | NA | | Yes | | | Fair |
| [75] | | Yes | | | Yes | | Yes | | | Yes | | Yes | | Yes | | | Yes | | | No | | | No | | Yes | | | Yes | | | Yes | | No | | | Yes | | NR | | | NA | | Yes | | | Good |
| [76] | | Yes | | | Yes | | Yes | | | Yes | | Yes | | Yes | | | Yes | | | No | | | Yes | | Yes | | | Yes | | | Yes | | No | | | CD | | NR | | | Yes | | Yes | | | Good |
| [77] | | Yes | | | Yes | | Yes | | | Yes | | Yes | | Yes | | | NR | | | Yes | | | No | | Yes | | | Yes | | | Yes | | No | | | No | | NR | | | NA | | Yes | | | Good |
| [78] | | Yes | | | Yes | | Yes | | | Yes | | Yes | | Yes | | | Yes | | | No | | | No | | Yes | | | Yes | | | Yes | | No | | | CD | | NR | | | NA | | Yes | | | Good |
| [79] | | Yes | | | Yes | | Yes | | | Yes | | Yes | | Yes | | | Yes | | | NR | | | Yes | | Yes | | | Yes | | | Yes | | Yes | | | Yes | | NR | | | Yes | | Yes | | | Good |
| [80] | | Yes | | | Yes | | Yes | | | Yes | | Yes | | Yes | | | Yes | | | Yes | | | No | | Yes | | | Yes | | | Yes | | No | | | Yes | | NR | | | Yes | | Yes | | | Good |
| [81] | | Yes | | | Yes | | Yes | | | Yes | | Yes | | Yes | | | Yes | | | Yes | | | Yes | | Yes | | | Yes | | | Yes | | No | | | No | | NR | | | NA | | No | | | Fair |
| [82] | | Yes | | | Yes | | Yes | | | Yes | | Yes | | NR | | | NR | | | No | | | No | | Yes | | | Yes | | | Yes | | Yes | | | CD | | NR | | | NA | | CD | | | Fair |
| [83] | | Yes | | | Yes | | Yes | | | Yes | | Yes | | Yes | | | Yes | | | Yes | | | No | | Yes | | | Yes | | | Yes | | No | | | Yes | | NR | | | NA | | Yes | | | Good |
| **Case-control studies** | | | | | | | | | | | | | | | | | | | | | | | | | | | | | | | | | | | | | | | | | | | | | | |
| Ref | | | Objective stated clearly | | | Population defined | | | Sample size justification | | | | Controls/ cases from same population | | | Inclusion / exclusion criteria defined | | | Cases defined and differentiated from controls | | | 100% eligible cases/ controls selected, or cases/ controls selected randomly | | | | Concurrent controls | | | Exposure/risk occurred prior to the development of case defining condition | | | Exposure measures valid | | | Outcomes assessors blinded | | | | | 2003 / 2013 WHO outcome methods 24, 25) | | Key potential confounding variables/matching adjusted for | | | Quality | |
| [84] | | | No | | | Yes | | | No | | | | Yes | | | Yes | | | Yes | | | Yes | | | | Yes | | | Yes | | | Yes | | | NR | | | | | Yes | | Yes | | | Good | |

Abbreviations: CD - cannot determine; NA – not applicable; NR – not reported – RCT – randomized controlled trial; WHO – World Health Organization

# References

1. Satzke C, Turner P, Virolainen-Julkunen A, Adrian PV, Antonio M, Hare KM, et al. Standard method for detecting upper respiratory carriage of *Streptococcus pneumoniae*: updated recommendations from the World Health Organization Pneumococcal Carriage Working Group. Vaccine. 2013;32(1):165-79. doi: 10.1016/j.vaccine.2013.08.062. PubMed PMID: 24331112.

2. O'Brien KL, Nohynek H. Report from a WHO Working Group: standard method for detecting upper respiratory carriage of *Streptococcus pneumoniae*. Ped Infect Dis J. 2003;22(2):e1-11. Epub 2003/02/15. doi: 10.1097/01.inf.0000049347.42983.77. PubMed PMID: 12586987.

3. Gils E, Veenhoven R, Rodenburg G, Hak E, Sanders E. Effect of 7-valent pneumococcal conjugate vaccine on nasopharyngeal carriage with *Haemophilus influenzae* and *Moraxella catarrhalis* in a randomized controlled trial. Vaccine. 2011;29(44):7595-8. doi: 10.1016/j.vaccine.2011.08.049. PubMed PMID: CN-00806054.

4. Abdullahi O, Karani A, Tigoi CC, Mugo D, Kungu S, Wanjiru E, et al. The prevalence and risk factors for pneumococcal colonization of the nasopharynx among children in Kilifi District, Kenya. PLoS One. 2012;7(2):e30787. doi: <https://dx.doi.org/10.1371/journal.pone.0030787>. PubMed PMID: 22363489.

5. Abdullahi O, Nyiro J, Lewa P, Slack M, Scott JA. The descriptive epidemiology of *Streptococcus pneumoniae* and *Haemophilus influenzae* nasopharyngeal carriage in children and adults in Kilifi district, Kenya. Ped Infect Dis J. 2008;27(1):59-64. doi: <https://dx.doi.org/10.1097/INF.0b013e31814da70c>. PubMed PMID: 18162940.

6. Adetifa IM, Antonio M, Okoromah CA, Ebruke C, Inem V, Nsekpong D, et al. Pre-vaccination nasopharyngeal pneumococcal carriage in a Nigerian population: epidemiology and population biology. PLoS One. 2012;7(1):e30548. doi: <https://dx.doi.org/10.1371/journal.pone.0030548>. PubMed PMID: 22291984.

7. Alfayate Miguélez S, Yague Guirao G, Menasalvas Ruíz AI, Sanchez-Solís M, Domenech Lucas M, González Camacho F, et al. Impact of pneumococcal vaccination in the nasopharyngeal carriage of *Streptococcus pneumoniae* in healthy children of the Murcia Region in Spain. Vaccines. 2020;9(1). Epub 2021/01/01. doi: 10.3390/vaccines9010014. PubMed PMID: 33379235; PubMed Central PMCID: PMCPMC7823743.

8. Almeida ST, Nunes S, Santos Paulo AC, Valadares I, Martins S, Breia F, et al. Low prevalence of pneumococcal carriage and high serotype and genotype diversity among adults over 60 years of age living in Portugal. PLoS One. 2014;9(3):e90974. doi: <https://dx.doi.org/10.1371/journal.pone.0090974>. PubMed PMID: 24604030.

9. Ansaldi F, de Florentiis D, Canepa P, Zancolli M, Martini M, Orsi A, et al. Carriage of *Streptococcus pneumoniae* 7 years after implementation of vaccination program in a population with very high and long-lasting coverage, Italy. Vaccine. 2012;30(13):2288-94. doi: <https://dx.doi.org/10.1016/j.vaccine.2012.01.067>. PubMed PMID: 22306795.

10. Arvas A, Cokugras H, Gur E, Gonullu N, Taner Z, Bahar Tokman H. Pneumococcal nasopharyngeal carriage in young healthy children after pneumococcal conjugate vaccine in Turkey. Balkan Med J. 2017. doi: 10.4274/balkanmedj.2016.1256. PubMed PMID: 28443585.

11. Assefa A, Gelaw B, Shiferaw Y, Tigabu Z. Nasopharyngeal carriage and antimicrobial susceptibility pattern of *Streptococcus pneumoniae* among pediatric outpatients at Gondar University Hospital, North West Ethiopia. PEDN. 2013;54(5):315-21. doi: <https://dx.doi.org/10.1016/j.pedneo.2013.03.017>. PubMed PMID: 23680262.

12. Bogaert D, van Belkum A, Sluijter M, Luijendijk A, de Groot R, Rumke HC, et al. Colonisation by *Streptococcus pneumoniae* and *Staphylococcus aureus* in healthy children. Lancet. 2004;363(9424):1871-2. doi: <https://dx.doi.org/10.1016/S0140-6736(04)16357-5>. PubMed PMID: 15183627.

13. Bojang A, Jafali J, Egere U, Hill P, Antonio M, Jeffries D. Seasonality of pneumococcal nasopharyngeal carriage in rural Gambia determined within the context of a cluster randomized pneumococcal vaccine trial. PLoS One. 2015;10(7):13. PubMed PMID: CN-01130937.

14. Camilli R, Daprai L, Cavrini F, Lombardo D, D'Ambrosio F, Del Grosso M, et al. Pneumococcal carriage in young children one year after introduction of the 13-valent conjugate vaccine in Italy. PLoS One. 2013;8(10):e76309. doi: <https://dx.doi.org/10.1371/journal.pone.0076309>. PubMed PMID: 24124543.

15. Camilli R, Vescio MF, Giufre M, Daprai L, Garlaschi ML, Cerquetti M, et al. Carriage of *Haemophilus influenzae* is associated with pneumococcal vaccination in Italian children. Vaccine. 2015;33(36):4559-64. doi: <https://dx.doi.org/10.1016/j.vaccine.2015.07.009>. PubMed PMID: 26190092.

16. Cardozo DM, Nascimento-Carvalho CM, Andrade AL, Silvany-Neto AM, Daltro CH, Brandao MA, et al. Prevalence and risk factors for nasopharyngeal carriage of *Streptococcus pneumoniae* among adolescents. J Med Microbiol. 2008;57(Pt 2):185-9. doi: <https://dx.doi.org/10.1099/jmm.0.47470-0>. PubMed PMID: 18201984.

17. Chan KC, Subramanian R, Chong P, Nelson EA, Lam HS, Li AM, et al. Pneumococcal carriage in young children after introduction of PCV13 in Hong Kong. Vaccine. 2016;34(33):3867-74. doi: 10.1016/j.vaccine.2016.05.047. PubMed PMID: 27265449.

18. Chang B, Akeda H, Nakamura Y, Hamabata H, Ameku K, Toma T, et al. Impact of thirteen-valent pneumococcal conjugate vaccine on nasopharyngeal carriage in healthy children under 24 months in Okinawa, Japan. JIC. 2020;26(5):465-70. doi: <https://dx.doi.org/10.1016/j.jiac.2019.12.009>.

19. Cheng Immergluck L, Kanungo S, Schwartz A, McIntyre A, Schreckenberger PC, Diaz PS. Prevalence of *Streptococcus pneumoniae* and *Staphylococcus aureus* nasopharyngeal colonization in healthy children in the United States. Epidemiol Infect. 2004;132(2):159-66. PubMed PMID: 15061489.

20. Cohen R, Levy C, Bonnet E, Thollot F, Boucherat M, Fritzell B, et al. Risk factors for serotype 19A carriage after introduction of 7-valent pneumococcal vaccination. BMC Infect Dis. 2011;11:95. doi: <https://dx.doi.org/10.1186/1471-2334-11-95>. PubMed PMID: 21501471.

21. Coles CL, Kanungo R, Rahmathullah L, Thulasiraj RD, Katz J, Santosham M, et al. Pneumococcal nasopharyngeal colonization in young South Indian infants. Ped Infect Dis J. 2001;20(3):289-95. PubMed PMID: 11303832.

22. Daana M, Rahav G, Hamdan A, Thalji A, Jaar F, Abdeen Z, et al. Measuring the effects of pneumococcal conjugate vaccine (PCV7) on *Streptococcus pneumoniae* carriage and antibiotic resistance: the Palestinian-Israeli Collaborative Research (PICR). Vaccine. 2015;33(8):1021-6. doi: 10.1016/j.vaccine.2015.01.003. PubMed PMID: 25593104.

23. Dunne EM, Choummanivong M, Neal EFG, Stanhope K, Nguyen CD, Xeuatvongsa A, et al. Factors associated with pneumococcal carriage and density in infants and young children in Laos PDR. PLoS One. 2019;14(10):e0224392. doi: <https://dx.doi.org/10.1371/journal.pone.0224392>.

24. Farida H, Severin JA, Gasem MH, Keuter M, Wahyono H, van den Broek P, et al. Nasopharyngeal carriage of *Streptococcus pneumoniae* in pneumonia-prone age groups in Semarang, Java Island, Indonesia. PLoS One. 2014;9(1):e87431. doi: <https://dx.doi.org/10.1371/journal.pone.0087431>. PubMed PMID: 24498104.

25. Finkelstein JA, Huang SS, Daniel J, Rifas-Shiman SL, Kleinman K, Goldmann D, et al. Antibiotic-resistant *Streptococcus pneumoniae* in the heptavalent pneumococcal conjugate vaccine era: predictors of carriage in a multicommunity sample. Pediatrics. 2003;112(4):862-9. PubMed PMID: 14523178.

26. Gebre T, Tadesse M, Aragaw D, Feye D, Beyene HB, Seyoum D, et al. Nasopharyngeal carriage and antimicrobial susceptibility patterns of *Streptococcus pneumoniae* among children under five in Southwest Ethiopia. Children. 2017;4(4). doi: 10.3390/children4040027. PubMed PMID: 28422083.

27. Haile AA, Gidebo DD, Ali MM. Colonization rate of Streptococcus pneumoniae, its associated factors and antimicrobial susceptibility pattern among children attending kindergarten school in Hawassa, southern Ethiopia. BMC Res Notes. 2019;12(1):344. Epub 2019/06/19. doi: 10.1186/s13104-019-4376-z. PubMed PMID: 31208447; PubMed Central PMCID: PMCPMC6580519.

28. Hill PC, Akisanya A, Sankareh K, Cheung YB, Saaka M, Lahai G, et al. Nasopharyngeal carriage of *Streptococcus pneumoniae* in Gambian villagers. Clin Infect Dis 2006;43(6):673-9. doi: <https://dx.doi.org/10.1086/506941>. PubMed PMID: 16912937.

29. Hoang VT, Dao TL, Ly TDA, Belhouchat K, Chaht KL, Gaudart J, et al. The dynamics and interactions of respiratory pathogen carriage among French pilgrims during the 2018 Hajj. Emerg Microbes Infect. 2019;8(1):1701-10. Epub 2019/11/22. doi: 10.1080/22221751.2019.1693247. PubMed PMID: 31749410; PubMed Central PMCID: PMCPMC6882464.

30. Hsieh YC, Chiu CH, Chang KY, Huang YC, Chen CJ, Kuo CY, et al. The impact of the heptavalent pneumococcal conjugate vaccine on risk factors for *Streptococcus pneumoniae* carriage in children. Ped Infect Dis J. 2012;31(9):e163-8. doi: <https://dx.doi.org/10.1097/INF.0b013e31825cb9f9>. PubMed PMID: 22592521.

31. Hsu KK, Rifas-Shiman SL, Shea KM, Kleinman KP, Lee GM, Lakoma M, et al. Do community-level predictors of pneumococcal carriage continue to play a role in the conjugate vaccine era? Epidemiol Infect. 2014;142(2):379-87. doi: <https://dx.doi.org/10.1017/S0950268813000794>. PubMed PMID: 23731707.

32. Hu J, Sun X, Huang Z, Wagner AL, Carlson B, Yang J, et al. *Streptococcus pneumoniae* and *Haemophilus influenzae* type b carriage in Chinese children aged 12-18 months in Shanghai, China: a cross-sectional study. BMC Infect Dis. 2016;16:149. doi: <https://dx.doi.org/10.1186/s12879-016-1485-3>. PubMed PMID: 27080523.

33. Huang SS, Finkelstein JA, Rifas-Shiman SL, Kleinman K, Platt R. Community-level predictors of pneumococcal carriage and resistance in young children. Am J Epidemiol. 2004;159(7):645-54. PubMed PMID: 15033642.

34. Huang SS, Hinrichsen VL, Stevenson AE, Rifas-Shiman SL, Kleinman K, Pelton SI, et al. Continued impact of pneumococcal conjugate vaccine on carriage in young children. Pediatrics. 2009;124(1):e1-11. doi: <https://dx.doi.org/10.1542/peds.2008-3099>. PubMed PMID: 19564254.

35. Inverarity D, Diggle M, Ure R, Johnson P, Altstadt P, Mitchell T, et al. Molecular epidemiology and genetic diversity of pneumococcal carriage among children in Beni State, Bolivia. Trans R Soc Trop Med Hyg. 2011;105(8):445-51. doi: <https://dx.doi.org/10.1016/j.trstmh.2011.04.013>. PubMed PMID: 21714978.

36. Karami M, Hosseini SM, Hashemi SH, Ghiasvand S, Zarei O, Safari N, et al. Prevalence of nasopharyngeal carriage of Streptococcus pneumoniae in children 7 to 14 years in 2016: A survey before pneumococcal conjugate vaccine introduction in Iran. Human vaccines & immunotherapeutics. 2019;15(9):2178-82. doi: <https://dx.doi.org/10.1080/21645515.2018.1539601>.

37. Koliou MG, Andreou K, Lamnisos D, Lavranos G, Iakovides P, Economou C, et al. Risk factors for carriage of *Streptococcus pneumoniae* in children. BMC Pediatr. 2018;18(1):144. Epub 2018/04/28. doi: 10.1186/s12887-018-1119-6. PubMed PMID: 29699525; PubMed Central PMCID: PMCPMC5921789.

38. Korona-Glowniak I, Malm A. Characteristics of *Streptococcus pneumoniae* strains colonizing upper respiratory tract of healthy preschool children in Poland. Sci World J. 2012.

39. Kuo CY, Hwang KP, Hsieh YC, Cheng CH, Huang FL, Shen YH, et al. Nasopharyngeal carriage of *Streptococcus pneumoniae* in Taiwan before and after the introduction of a conjugate vaccine. Vaccine. 2011;29(32):5171-7. doi: <https://dx.doi.org/10.1016/j.vaccine.2011.05.034>. PubMed PMID: 21621578.

40. Labout JA, Duijts L, Arends LR, Jaddoe VW, Hofman A, de Groot R, et al. Factors associated with pneumococcal carriage in healthy Dutch infants: the generation R study. J Pediatr. 2008;153(6):771-6. doi: <https://dx.doi.org/10.1016/j.jpeds.2008.05.061>. PubMed PMID: 18621390.

41. Lee GM, Kleinman K, Pelton SI, Hanage W, Huang SS, Lakoma M, et al. Impact of 13-Valent Pneumococcal Conjugate Vaccination on *Streptococcus pneumoniae* Carriage in Young Children in Massachusetts. J Pediatric Infect Dis Soc. 2014;3(1):23-32. doi: 10.1093/jpids/pit057. PubMed PMID: 24567842.

42. le Polain de Waroux O, Flasche S, Kucharski AJ, Langendorf C, Ndazima D, Mwanga-Amumpaire J, et al. Identifying human encounters that shape the transmission of *Streptococcus pneumoniae* and other acute respiratory infections. Epidemics. 2018;25:72-9. Epub 2018/07/29. doi: 10.1016/j.epidem.2018.05.008. PubMed PMID: 30054196; PubMed Central PMCID: PMCPMC6227246.

43. Lindstrand A, Kalyango J, Alfven T, Darenberg J, Kadobera D, Bwanga F, et al. Pneumococcal carriage in children under five years in Uganda-will present pneumococcal conjugate vaccines be appropriate? PLoS One. 2016;11(11):e0166018. doi: 10.1371/journal.pone.0166018. PubMed PMID: 27829063.

44. Mackenzie GA, Leach AJ, Carapetis JR, Fisher J, Morris PS. Epidemiology of nasopharyngeal carriage of respiratory bacterial pathogens in children and adults: cross-sectional surveys in a population with high rates of pneumococcal disease. BMC Infect Dis. 2010;10:304. doi: <https://dx.doi.org/10.1186/1471-2334-10-304>. PubMed PMID: 20969800.

45. Memish ZA, Assiri A, Almasri M, Alhakeem RF, Turkestani A, Al Rabeeah AA, et al. Impact of the Hajj on pneumococcal transmission. Clin Microbiol Infect. 2015;21(1):77.e11-8. doi: <https://dx.doi.org/10.1016/j.cmi.2014.07.005>. PubMed PMID: 25636939.

46. Menezes AP, Azevedo J, Leite MC, Campos LC, Cunha M, Carvalho Mda G, et al. Nasopharyngeal carriage of *Streptococcus pneumoniae* among children in an urban setting in Brazil prior to PCV10 introduction. Vaccine. 2016;34(6):791-7. doi: <https://dx.doi.org/10.1016/j.vaccine.2015.12.042>. PubMed PMID: 26742946.

47. Millar EV, O'Brien KL, Zell ER, Bronsdon MA, Reid R, Santosham M. Nasopharyngeal carriage of *Streptococcus pneumoniae* in Navajo and White Mountain Apache children before the introduction of pneumococcal conjugate vaccine. Ped Infect Dis J. 2009;28(8):711-6. Epub 2009/07/14. doi: 10.1097/INF.0b013e3181a06303. PubMed PMID: 19593248.

48. Moore MR, Hyde TB, Hennessy TW, Parks DJ, Reasonover AL, Harker-Jones M, et al. Impact of a conjugate vaccine on community-wide carriage of nonsusceptible *Streptococcus pneumoniae* in Alaska. J Infect Dis. 2004;190(11):2031-8. doi: <https://dx.doi.org/10.1086/425422>. PubMed PMID: 15529269.

49. Murad C, Dunne EM, Sudigdoadi S, Fadlyana E, Tarigan R, Pell CL, et al. Pneumococcal carriage, density, and co-colonization dynamics: A longitudinal study in Indonesian infants. IJID. 2019;86:73-81. doi: <https://dx.doi.org/10.1016/j.ijid.2019.06.024>.

50. Nackers F, Cohuet S, le Polain de Waroux O, Langendorf C, Nyehangane D, Ndazima D, et al. Carriage prevalence and serotype distribution of *Streptococcus pneumoniae* prior to 10-valent pneumococcal vaccine introduction: A population-based cross-sectional study in South Western Uganda, 2014. Vaccine. 2017;35(39):5271-7. Epub 2017/08/09. doi: 10.1016/j.vaccine.2017.07.081. PubMed PMID: 28784282; PubMed Central PMCID: PMCPMC6616034.

51. Navne JE, Borresen ML, Slotved HC, Andersson M, Melbye M, Ladefoged K, et al. Nasopharyngeal bacterial carriage in young children in Greenland: a population at high risk of respiratory infections. Epidemiol Infect. 2016;144(15):3226-36. doi: 10.1017/s0950268816001461. PubMed PMID: 27405603.

52. Neal EFG, Nguyen C, Ratu FT, Matanitobua S, Dunne EM, Reyburn R, et al. A Comparison of Pneumococcal Nasopharyngeal Carriage in Very Young Fijian Infants Born by Vaginal or Cesarean Delivery. JAMA network open. 2019;2(10):e1913650. doi: <https://dx.doi.org/10.1001/jamanetworkopen.2019.13650>.

53. Neal EFG, Flasche S, Nguyen CD, Ratu FT, Dunne EM, Koyamaibole L, et al. Associations between ethnicity, social contact, and pneumococcal carriage three years post-PCV10 in Fiji. Vaccine. 2020;38(2):202-11. doi: <https://dx.doi.org/10.1016/j.vaccine.2019.10.030>.

54. Neal EFG, Nguyen CD, Ratu FT, Dunne EM, Kama M, Ortika BD, et al. Factors associated with pneumococcal carriage and density in children and adults in Fiji, using four cross-sectional surveys. PLoS One. 2020;15(4):e0231041. doi: <https://dx.doi.org/10.1371/journal.pone.0231041>.

55. Neves FPG, Cardoso NT, Snyder RE, Marlow MA, Cardoso CAA, Teixeira LM, et al. Pneumococcal carriage among children after four years of routine 10-valent pneumococcal conjugate vaccine use in Brazil: The emergence of multidrug resistant serotype 6C. Vaccine. 2017;35(21):2794-800. doi: 10.1016/j.vaccine.2017.04.019. PubMed PMID: 28431817.

56. Nguyen HAT, Fujii H, Vu HTT, Parry CM, Dang AD, Ariyoshi K, et al. An alarmingly high nasal carriage rate of Streptococcus pneumoniae serotype 19F non-susceptible to multiple beta-lactam antimicrobials among Vietnamese children. BMC Infect Dis. 2019;19(1):241. Epub 2019/03/15. doi: 10.1186/s12879-019-3861-2. PubMed PMID: 30866853; PubMed Central PMCID: PMCPMC6416861.

57. Otsuka T, Chang B, Shirai T, Iwaya A, Wada A, Yamanaka N, et al. Individual risk factors associated with nasopharyngeal colonization with *Streptococcus pneumoniae* and *Haemophilus influenzae*: a Japanese birth cohort study. Ped Infect Dis J. 2013;32(7):709-14. doi: <https://dx.doi.org/10.1097/INF.0b013e31828701ea>. PubMed PMID: 23411622.

58. Ousmane S, Diallo BA, Ouedraogo R, Sanda AA, Soussou AM, Collard JM. Serotype distribution and antimicrobial sensitivity profile of *Streptococcus pneumoniae c*arried in healthy toddlers before PCV13 introduction in Niamey, Niger. PLoS One. 2017;12(1):e0169547. doi: 10.1371/journal.pone.0169547. PubMed PMID: 28103262.

59. Ozdemir B, Beyazova U, Camurdan AD, Sultan N, Ozkan S, Sahin F. Nasopharyngeal carriage of *Streptococcus pneumoniae* in healthy Turkish infants. J Infect Dis. 2008;56(5):332-9. doi: <https://dx.doi.org/10.1016/j.jinf.2008.02.010>. PubMed PMID: 18377994.

60. Ozdemir H, Ciftci E, Durmaz R, Guriz H, Aysev AD, Karbuz A, et al. Risk factors for nasopharyngeal carriage of *Streptococcus pneumoniae* in healthy Turkish children after the addition of heptavalent pneumococcal conjugate vaccine (PCV7) to the national vaccine schedule. Turk J Pediatr. 2013;55(6):575-83. Epub 2014/03/01. PubMed PMID: 24577974.

61. Park SY, Moore MR, Bruden DL, Hyde TB, Reasonover AL, Harker-Jones M, et al. Impact of conjugate vaccine on transmission of antimicrobial-resistant *Streptococcus pneumoniae* among Alaskan children. Ped Infect Dis J. 2008;27(4):335-40. doi: <https://dx.doi.org/10.1097/INF.0b013e318161434d>. PubMed PMID: 18316986.

62. Regev-Yochay G, Raz M, Dagan R, Porat N, Shainberg B, Pinco E, et al. Nasopharyngeal carriage of *Streptococcus pneumoniae* by adults and children in community and family settings. Clin Infect Dis 2004;38(5):632-9. doi: <https://dx.doi.org/10.1086/381547>. PubMed PMID: 14986245.

63. Reis JN, Palma T, Ribeiro GS, Pinheiro RM, Ribeiro CT, Cordeiro SM, et al. Transmission of *Streptococcus pneumoniae* in an urban slum community. J Infect Dis. 2008;57(3):204-13. doi: <https://dx.doi.org/10.1016/j.jinf.2008.06.017>. PubMed PMID: 18672297.

64. Reisman J, Rudolph K, Bruden D, Hurlburt D, Bruce MG, Hennessy T. Risk factors for pneumococcal colonization of the nasopharynx in Alaska native adults and children. J Pediatric Infect Dis Soc. 2014;3(2):104-11. doi: 10.1093/jpids/pit069. PubMed PMID: 26625363.

65. Ricketson LJ, Wood ML, Vanderkooi OG, MacDonald JC, Martin IE, Demczuk WH, et al. Trends in asymptomatic nasopharyngeal colonization with *Streptococcus pneumoniae* after introduction of the 13-valent pneumococcal conjugate vaccine in Calgary, Canada. Ped Infect Dis J. 2014;33(7):724-30. doi: <https://dx.doi.org/10.1097/INF.0000000000000267>. PubMed PMID: 24463806.

66. Rivera-Olivero IA, del Nogal B, Sisco MC, Bogaert D, Hermans PW, de Waard JH. Carriage and invasive isolates of *Streptococcus pneumoniae* in Caracas, Venezuela: the relative invasiveness of serotypes and vaccine coverage. Eur J Clin Microbiol Infect Dis. 2011;30(12):1489-95. doi: <https://dx.doi.org/10.1007/s10096-011-1247-5>. PubMed PMID: 21499972.

67. Russell FM, Carapetis JR, Ketaiwai S, Kunabuli V, Taoi M, Biribo S, et al. Pneumococcal nasopharyngeal carriage and patterns of penicillin resistance in young children in Fiji. Ann Trop Paediatr 2006;26(3):187-97. doi: <https://dx.doi.org/10.1179/146532806X120273>. PubMed PMID: 16925955.

68. Samore MH, Magill MK, Alder SC, Severina E, Morrison-De Boer L, Lyon JL, et al. High rates of multiple antibiotic resistance in *Streptococcus pneumoniae* from healthy children living in isolated rural communities: association with cephalosporin use and intrafamilial transmission. Pediatrics. 2001;108(4):856-65. PubMed PMID: 11581436.

69. Shiri T, Nunes MC, Adrian PV, Van Niekerk N, Klugman KP, Madhi SA. Interrelationship of *Streptococcus pneumoniae*, *Haemophilus influenzae* and *Staphylococcus aureus* colonization within and between pneumococcal-vaccine naive mother-child dyads. BMC Infect Dis. 2013;13:483. doi: <https://dx.doi.org/10.1186/1471-2334-13-483>. PubMed PMID: 24134472.

70. Skosana Z, Von Gottberg A, Olorunju S, Mohale T, Du Plessis M, Adams T, et al. Non-vaccine serotype pneumococcal carriage in healthy infants in South Africa following introduction of the 13-valent pneumococcal conjugate vaccine. S Afr Med J. 2021;111(2):143-8. Epub 2021/05/05. doi: 10.7196/SAMJ.2021.v111i2.14626. PubMed PMID: 33944725.

71. Sung RY, Ling JM, Fung SM, Oppenheimer SJ, Crook DW, Lau JT, et al. Carriage of *Haemophilus influenzae* and *Streptococcus pneumoniae* in healthy Chinese and Vietnamese children in Hong Kong. Acta Paediatr. 1995;84(11):1262-7. PubMed PMID: 8580623.

72. Toledo ME, Casanova MF, Linares-Perez N, Garcia-Rivera D, Torano Peraza G, Barcos Pina I, et al. Prevalence of pneumococcal nasopharyngeal carriage among children 2-18 months of age: baseline study pre-introduction of pneumococcal vaccination in Cuba. Ped Infect Dis J. 2017;36(1):e22-e8. doi: 10.1097/inf.0000000000001341. PubMed PMID: 27649366.

73. Uddén F, Filipe M, Slotved HC, Yamba-Yamba L, Fuursted K, Pintar Kuatoko P, et al. Pneumococcal carriage among children aged 4 - 12 years in Angola 4 years after the introduction of a pneumococcal conjugate vaccine. Vaccine. 2020;38(50):7928-37. Epub 2020/11/05. doi: 10.1016/j.vaccine.2020.10.060. PubMed PMID: 33143954.

74. Ueno M, Ishii Y, Tateda K, Anahara Y, Ebata A, Iida M, et al. Prevalence and risk factors of nasopharyngeal carriage of *Streptococcus pneumoniae* in healthy children in Japan. Jpn J Infect Dis. 2013;66(1):22-5. PubMed PMID: 23429080.

75. Usuf E, Badji H, Bojang A, Jarju S, Ikumapayi UN, Antonio M, et al. Pneumococcal carriage in rural Gambia prior to the introduction of pneumococcal conjugate vaccine: a population-based survey. Trop Med Int Health. 2015;20(7):871-9. doi: <https://dx.doi.org/10.1111/tmi.12505>. PubMed PMID: 25778937.

76. Usuf E, Bojang A, Camara B, Jagne I, Oluwalana C, Bottomley C, et al. Maternal pneumococcal nasopharyngeal carriage and risk factors for neonatal carriage after the introduction of pneumococcal conjugate vaccines in The Gambia. Clin Microbiol Infect. 2018;24(4):389-95. Epub 2017/07/27. doi: 10.1016/j.cmi.2017.07.018. PubMed PMID: 28743545.

77. Uzuner A, Ilki A, Akman M, Gundogdu E, Erbolukbas R, Kokacya O, et al. Nasopharyngeal carriage of penicillin-resistant *Streptococcus pneumoniae* in healthy children. Turk J Pediatr 2007;49(4):370-8. Epub 2008/02/06. PubMed PMID: 18246737.

78. Verhagen LM, Hermsen M, Rivera-Olivero IA, Sisco MC, de Jonge MI, Hermans PW, et al. Nasopharyngeal carriage of respiratory pathogens in Warao Amerindians: significant relationship with stunting. Trop Med Int Health. 2017;22(4):407-14. doi: 10.1111/tmi.12835. PubMed PMID: 28072501.

79. Vanker A, Nduru PM, Barnett W, Dube FS, Sly PD, Gie RP, et al. Indoor air pollution and tobacco smoke exposure: impact on nasopharyngeal bacterial carriage in mothers and infants in an African birth cohort study. ERJ Open Res. 2019;5(1). Epub 2019/02/12. doi: 10.1183/23120541.00052-2018. PubMed PMID: 30740462; PubMed Central PMCID: PMCPMC6360211.

80. von Mollendorf C, Dunne EM, La Vincente S, Ulziibayar M, Suuri B, Luvsantseren D, et al. Pneumococcal carriage in children in Ulaanbaatar, Mongolia before and one year after the introduction of the 13-valent pneumococcal conjugate vaccine. Vaccine. 2019;37(30):4068-75. doi: <https://dx.doi.org/10.1016/j.vaccine.2019.05.078>.

81. Wada FW, Tufa EG, Berheto TM, Solomon FB. Nasopharyngeal carriage of Streptococcus pneumoniae and antimicrobial susceptibility pattern among school children in South Ethiopia: post-vaccination era. BMC research notes. 2019;12(1):306. doi: <https://dx.doi.org/10.1186/s13104-019-4330-0>.

82. Wroe PC, Lee GM, Finkelstein JA, Pelton SI, Hanage WP, Lipsitch M, et al. Pneumococcal carriage and antibiotic resistance in young children before 13-valent conjugate vaccine. Ped Infect Dis J. 2012;31(3):249-54. doi: <https://dx.doi.org/10.1097/INF.0b013e31824214ac>. PubMed PMID: 22173142.

83. Zuccotti G, Mameli C, Daprai L, Garlaschi ML, Dilillo D, Bedogni G, et al. Serotype distribution and antimicrobial susceptibilities of nasopharyngeal isolates of *Streptococcus pneumoniae* from healthy children in the 13-valent pneumococcal conjugate vaccine era. Vaccine. 2014;32(5):527-34. doi: 10.1016/j.vaccine.2013.12.003. PubMed PMID: 24342249.

84. Coles CL, Sherchand JB, Khatry SK, Katz J, Leclerq SC, Mullany LC, et al. Nasopharyngeal carriage of *S. pneumoniae* among young children in rural Nepal. Trop Med Int Health. 2009;14(9):1025-33. Epub 2009/07/01. doi: 10.1111/j.1365-3156.2009.02331.x. PubMed PMID: 19563428; PubMed Central PMCID: PMCPMC2770711.
