# Supplementary material for: Factors associated with pneumococcal nasopharyngeal carriage: A systematic review
Source: PLOS Glob Public Health. 2022 Apr 11;2(4):e0000327. doi: 10.1371/journal.pgph.0000327 (PMC10021834; doi:10.1371/journal.pgph.0000327)
Supplement: S5 Table — (DOCX) [file pgph.0000327.s007.docx]

**S5 Table. Factors assessed for association with pneumococcal nasopharyngeal carriage, stratified by World Bank income status, WHO region, and country.**

| **WHO region^a^** | **Country** | **Ref** | **Age group** | **Carriage rates**  **% (95% CI)^b^** | **Risk factors** | **Estimate of association (95% CI) *P*-value ^b^** | |
| --- | --- | --- | --- | --- | --- | --- | --- |
| **Low-income countries^c^ (n = 17)** | | | | | | | |
| Africa | Ethiopia | [1] | ≤ 10 years | 41.03% | Participant sex  Male  Female  Age (years)  8–10  < 3  3–5  5-< 8  Residence  Urban  Rural  Family size  5 +  < 5  Siblings < 5 years  No  Yes  Siblings > 5 years  No  Yes  Sleeping with parents  No  Yes  Number of rooms in the house  2+  1  Recent antibiotic use  No  Yes  Otitis media  No  Yes  Upper respiratory tract infection  No  Tonsillopharyngitis  Sinusitis  Lower respiratory tract infection  No  Tuberculosis  Pneumonia | *ref*  0.92 (0.51–1.7) *P* = 0.783  *ref*  aOR 3.5 (1.4-8.7) *P* = 0.007  aOR 1.6 (0.68–3.9) *P* = 0.275  aOR 1.8 (0.75–4.4) *P* = 0.183  *ref*  aOR 0.69 (0.33–1.44) *P* = 0.324  *ref*  aOR 1.38 (0.69–2.74) *P* = 0.358  *ref*  aOR 2.84 (1.40–5.76) *P* = 0.004  *ref*  aOR 0.70 (0.33–1.51) *P* =0.362  *ref*  aOR 0.72 (0.22–2.42) *P* = 0.600  *ref*  aOR 2.96 (1.46-5.99) *P* = 0.003  *ref*  aOR (0.89 (0.48–1.64) *P* = 0.701  *ref*  aOR 2.20 (0.58–8.31) P = 0.246  *ref*  aOR 2.84 (0.82–9.86) P = 0.101  aOR 1.92 (0.71–5.15) P 0.198  *ref*  aOR 1.60 (0.64–4.03) *P* = 0.316  aOR 3.55 (1.37-9.20) *P* = 0.009 | |
|  |  | [2] | < 5 years | 43.8% | Attending school  No  Yes  Having siblings  No  Yes  Sibling(s) < 5 years old  No  Yes  PCV vaccination status  Unvaccinated (0 doses)  Partially (vaccinated with 1 or 2 doses)  Fully (vaccinated with 3 doses)  Malnutrition  No  Yes  Sinusitis  No  Yes  Otitis media  No  Yes | *ref*  aOR 2.12 (0.72–6.28) P = 0.18  *ref*  aOR 1.16 (0.66–2.03) P = 0.61  *ref*  aOR 1.80 (1.17-2.77) *P* = 0.08  *ref*  aOR 1.07 (0.60–1.89) P = 0.82  aOR 1.06 (0.40–2.83) P = 0.90  *ref*  aOR 2.07 (1.24-3.44) *P* = 0.05  *ref*  aOR 2.02 (0.77–5.30) *P*= 0.15  *ref*  aOR 3.15 (0.09–103.9) *P* = 0.07 | |
|  |  | [3] | 3–5 years | 62.5% | Age < 5 years  Co-sleeping with siblings | aOR 2.09 (1.13–3.87) *P* = 0.02  aOR 1.89 (1.15–3.09) *P* = 0.01 | |
|  |  |  | 6–13 years | 38.6% |  |  |  |
|  |  |  | Total | 43.8% |  |  |  |
|  |  |  | 4 years | 12.2% |  |  |  |
|  |  |  | 5 years | 17.6% |  |  |  |
|  |  |  | 6 years | 22.1% |  |  |  |
|  |  | [4] | 3–4 years | 34.5% | Age (years)  3–4  5–6  Siblings < 5 years old  No  Yes  Childcare attendance  No  Yes  Smoker in a house  No  Yes  Sharing bed with parents  No  Yes  Cooking in bedroom  No  Yes  Previous hospitalization  No  Yes | *ref*  aOR 3.1 (1.3–7.6) *P* = 0.010  *ref*  aOR 2.9 (1.3–6.8) *P* = 0.011  *ref*  aOR 0.7 (0.3–1.5) *P* = 0.345  *ref*  aOR 2.6 (0.6–12.3) *P* = 0.225  *ref*  aOR 3.5 (1.4–8.4) *P* = 0.005  *ref*  aOR 3.6 (1.6–7.9) *P* = 0.002  *ref*  aOR 3.5 (1.5–8.2) *P* = 0.004 | |
|  |  |  | 5–6 years | 18.7% |  |  |  |
|  | Kenya | [5] | < 1 year | 59% (49–69) | Rainy season  Coryza  Culture of non-capsulate *H. influenzae*  Taken amoxicillin/ampicillin in last 2 weeks | aOR 1.65 (1.11-2.43)  aOR 2.29 (1.48-3.56)  aOR 7.46 (3.94-14.1)  aOR 0.15 (0.03-0.76) | |
|  |  |  | 1–2 years | 61% (52–69) |  |  |  |
|  |  |  | 3–4 years | 50% (41–60) |  |  |  |
|  |  |  | 5–9 years | 41% (32–51) |  |  |  |
|  |  |  | 10–19 years | 9.6% (4.7–17) |  |  |  |
|  |  |  | 20–29 years | 7.8% (3.4–15) |  |  |  |
|  |  |  | 30–49 years | 3.2% (0.7–9.1) |  |  |  |
|  |  |  | > 50 years | 4.7% (1.5–11) |  |  |  |
|  |  | [6] | 3-59 months | 65.8% (64.0–67.5) | Cough in the last 2 weeks  Coryza, in the last 2 weeks  Antibiotics in last 2 weeks  Study time, by month | aOR 1.55 (1.26–1.91)  aOR 2.62 (2.12–3.34)  aOR 0.53 (0.34–0.81)  aOR 0.98 (0.96–0.99) | |
|  | Niger | [7] | 0-2 years | 54.5% | Age > 3 months  Breastfeeding  > 1 sibling < 6 years  Smoking environment  Antibiotic treatment 3 months to 7 days prior to enrolment  History of infection in previous 3 months  Childcare attendance  Participant sex  Female  Male  Prematurity  Ethnicity  Zarna-Sonrai  Hausa  Kanouri  Burkinabe  Gurmantche  Fulan  Togolese  Tuareg  Beninoise | aOR 2.3 (1.4-2.9) *P* < 0.001  aOR 1.5 (0.8–2.8) *P* 0.172  aOR 2.1 (1.5–2.9) *P* < 0.001  aOR 1.2 (0.9–1.7) *P* = 0.203  aOR 0.9 (0.5–1.5) *P* = 0.9  aOR 0.9 (0.6–1.4) *P* = 0.577  aOR 1.1 (0.7–1.8) *P*= 0.665  ref  aOR 0.8 (0.6–1.1) *P* = 0.684  aOR 1.0 (0.6 -14.7) *P* = 0.928  aOR 0.4 (0–1.6) P = 0.255  aOR 0.3 (0–1.4) *P* = 0.166  aOR 0.04 (0–0.03) *P* = 0.001  aOR 1.2 (0.3–5.4) *P* = 0.807  aOR 0.2 (0–1.8) *P* = 0.175  aOR 0.8 (0.1–4.8) *P* = 0.850  aOR 0.6 (0.1–2.9) *P* 0.527  aOR 1.0 (0.2–4.1) *P* = 0.974  aOR 2.1 (0.5–10.3) *P* = 0.354 | |
|  | The Gambia | [8] | > 30 months | 55.0% | Season  Rainy  Dry  Age in July 2006, years  2.5-<5  5-< 15  > 15  Participant sex  Male  Female | *ref*  aOR 1.87 (1.58-2.21) *P* < 0.001  *ref*  aOR 0.34 (0.25-0.45) *P* < 0.001  aOR 0.06 (0.04-0.08) *P* < 0.01  *ref*  aOR 0.84 (0.67–1.06) *P* = 0.150 | |
|  |  | [9] | ≥ 18 years | 13.5% | Individual level  Age (years)  18–24  25- 39  > 40  Participant sex  Male  Female  Smoke  No  Yes  Smoke (only males smoked)  No  Cigarettes  Tobacco  Another smoker in the household  No  Cigarettes  Tobacco  Both  Occupation  Farmer  Other  Schooling (attends/attended primary, secondary or tertiary)  No  Yes  Antibiotic  No  Yes  Common cold  No  Yes  Number of times per day sits at open fire  0  1–2  > 3 | *ref*  1.0 (0.67-1.52)  0.6 (0.37-1.09)  global *P* = 0.04  *ref*  1.1 (0.70-1.75) *P* = 0.44  *ref*  0.9 (0.40-1.94) *P* = 0.75  *ref*  0.6 (0.2-1.6)  3.0 (1.2-7.6)  global *P* = 0.0006  *ref*  1.8 (1.06-329)  1.6 (0.90-2.76)  2.6 (0.50-13.7)  global *P* = 0.06  *ref*  0.9 (0.65-1.31) *P* = 0.67  *ref*  1.1 (0.61-1.89) *P* = 0.80  *ref*  0.51(0.14-1.88) *P* = 0.01  *ref*  1.4 (0.98-2.06) *P* = 0.07  *ref*  0.7 (0.56-1.01)  1.1 (0.79-1.52)  global *P* = 0.04 | |
|  |  |  |  |  | Household-level  Number of pneumococcal vaccine trial children per household  0  1  > 2  Number of pneumococcal conjugate vaccine children per household  0  > 1  Number of placebo children per household  0  > 1  Number of individuals per household  ≤15  16-30  >30  Number of children < 5 years in the household  < 3  3–5  > 5  Household cooking place  Inside  Outside  A child with a runny nose in household  No  Yes  No. of children on the bed  0  1  > 2  Average age (years) of children on the bed  < 5  5–9  > 10 | *ref*  0.55 (0.31-0.97)  0.97 (0.57-1.34)  global *P* = 0.06  *ref*  0.91 (0.48-1.72) *P* = 0.78  *ref*  2.07 (1.32-3.51) *P* = 0.007  *ref*  1.50 (0.62-3.53)  1.60 (0.77-3.31)  global *P* = 0.06  *ref*  0.96 (0.51-1.80)  1.30 (0.7-2.6)  global *P* = 0.07  *ref*  0.62 (0.37-1.10) *P* =0.09  *ref*  1.29 (0.75-2.22) *P* = 0.03  *ref*  1.04 (0.73-1.49)  1.19 (0.741-1.9)  global *P* = 0.12  *ref*  0.71 (0.36-1.40)  0.73 (0.32-1.66)  global *P* = 0.16 | |
|  |  | [10] | 0-28 days | 0.3% at day 0  37.2% at day 28 | Birthweight  < 2.5 kgs  > 2.5 kgs  Mother carrier any time during the neonatal period  No  Yes  Other children in the household  No  Yes  Season  Rainy  Dry | *ref*  aOR 3.461 (0.91–13.11) P = 0.068  *ref*  aOR 2.82 (1.77-4.80) *P* < 0.001  *ref*  aOR 4.06 (1.90-8.86) *P* < 0.001  *ref*  aOR 1.98 (1.15-3.43) *P* = 0.014 | |
|  |  | [11] | < 1 month | 93% | Ethnicity  Jola  Mandinka  Fula  Other  House floor material  Natural material  Cement/concrete  Number of luxury items owned  < 4 items  > 4 items  Smoker in household  No  Yes  Child attending school  No  Yes  Number of other children sleeping in the room  < 3  > 3  BMI quartile (among children aged 0–5 years)  1  2  3  4  Breastfed  No  Yes | *ref*  aOR 0.86 (0.63–1.18) *P* = 0.34  aOR 0.91 (0.58–1.41) P = 0.67  aOR 0.66 (0.45-0.98) *P* = 0.04  *ref*  aOR 0.91 (0.68–1.23) *P* = 0.55  *ref*  aOR 0.89 (0.69–1.15) *P* = 0.37  *ref*  0.91 (0.74–1.12) *P* = 0.36  *ref*  aOR 0.82 (0.66–1.03) *P* = 0.09  *ref*  aOR 0.97 (0.70–1.33) *P* = 0.83  *ref*  aOR 1.32 (0.50–3.46) *P* 0.58  aOR 0.76 (0.28–2.04) *P* = 0.59  aOR 0.85 (0.41–1.76) *P* = 0.66  *ref*  aOR 0.83 (0.38–1.80) *P* = 0.64 | |
|  |  |  | < 3 months | 96% |  |  |  |
|  |  |  | < 1 year | 97% |  |  |  |
|  |  |  | 1-4 years | ~90% (estimated from bar graph) |  |  |  |
|  |  |  | 5-14 years | ~83% (estimated from bar graph) |  |  |  |
|  |  |  | 15-39 years | ~63% (estimated from bar graph) |  |  |  |
|  |  |  | > 40 years | 51% |  |  |  |
|  |  |  | All ages | 72% |  |  |  |
|  | Uganda | [12] | < 5 years | 75.1% | All contacts  Physical contacts  Non-physical contacts  Household contacts  Non-household contacts  Contacts > 1 hour  Contacts < 1 hour | aRR 1.03 (1.00–1.07)  aRR 1.06 (1.02–1.09)  aRR 0.97 (0.91–1.05)  aRR 1.04 (0.99–1.10)  aRR 1.03 (0.98–1.09)  aRR 1.06 (1.02–1.10)  aRR 0.94 (0.85–1.03) | |
|  |  |  | 5–9 years | 45.9% |  |  |  |
|  |  |  | 10–19 years | 19.3% |  |  |  |
|  |  |  | 20–39 years | 6.1% |  |  |  |
|  |  |  | > 40 years | 4.8% |  |  |  |
|  |  | [13] | < 5 years | 56% in 2009  55% in 2011 | Increasing age, months  Participant sex  Male  Female  Ill in 2 weeks before the survey  No  Yes  Ill on the day of the interview  No  Yes | aOR 0.99 (0.99–1.00) *P* = 0.46  *ref*  aOR 0.99 (0.71–1.38) *P* = 0.94  *ref*  aOR 0.90 (0.54–1.51) *P* = 0.69  *ref*  aOR 1.50 (1.03-2.19) *P* = 0.04 | |
|  |  | [14] | < 2 years | 77.0% (72.5–81.0) | Participant sex  Male  Female  History of oral antibiotic treatment  No  Yes  Respiratory symptoms  No  Yes | *ref*  aOR 0.83 (0.63–1.09)  ref  aOR 1.09 (0.68–1.76)  *ref*  1.17 (0.77–1.77) | |
|  |  |  | 2–4 years | 71.4% (63.8–78.0) |  |  |  |
|  |  |  | 5–15 years | 38.7% (33.5–44.3) |  |  |  |
|  |  |  | > 15 years | 8.5% (5.7–12.4) |  |  |  |
| South-East Asia | India | [15] | 2-2.5 months | 54% | Participant sex  Male  Female  Infant fed colostrum  No  Yes  History of night blindness during pregnancy  No  Yes  Total number of cigarettes smoked per day  < 20  ⩾ 20  Fuel  Wood  Other  Season  Dry/cool  Humid/rainy  Mother’s years of education  > 1  0  Conveyance  Bicycle  None  Siblings < 5 years  < 1  > 2 | *ref*  aOR 1.73 (1.17-2.56)  *ref*  aOR 2.14 (1.30-3.56)  *ref*  aOR 1.09 (0.54–2.17)  *ref*  aOR 1.91 (1.0-3.68)  *ref*  aOR 0.66 (0.27–1.59)  *ref*  aOR 1.38 (0.94–2.04)  *ref*  aOR 1.72 (1.15-2.57)  *ref*  aOR 1.48 (0.99-2.20)  *ref*  aOR 1.06 (0.61–1.85) | |
|  |  |  | 4 months | 64.1% | Participant sex  Male  Female  Infant fed colostrum  No  Yes  History of night blindness during pregnancy  No  Yes  Total number of cigarettes smoked per day  < 20  ⩾ 20  Fuel  Wood  Other  Season  Dry/cool  Humid/rainy  Mother’s years of education  > 1  0  Conveyance  Bicycle  None  Siblings < 5 years  < 1  > 2 | *ref*  aOR 1.47 (0.96–2.25)  *ref*  aOR 1.09 (0.64–1.86)  *ref*  aOR 0.69 (0.34–1.39)  *ref*  aOR 1.51 (0.75–3.03)  *ref*  aOR 0.98 (0.39–2.45)  *ref*  aOR 1.14 (0.72–1.78)  *ref*  aOR 1.10 (0.72–1.71)  *ref*  aOR 1.20 (0.78–1.84)  *ref*  aOR 2.39 (1.21–4.72) | |
|  |  |  | 6 months | 70.2% | Participant sex  Male  Female  Infant fed colostrum  No  Yes  History of night blindness during pregnancy  No  Yes  Total number of cigarettes smoked per day  < 20  ⩾ 20  Fuel  Wood  Other  Season  Dry/cool  Humid/rainy  Mother’s years of education  > 1  0  Conveyance  Bicycle  None  Siblings < 5 years  < 1  > 2 | *ref*  aOR 1.36 (0.38–2.73)  *ref*  aOR 0.83 (0.34–2.03)  *ref*  aOR 3.04 (0.61–15.13)  *ref*  aOR 0.63 (0.20–1.98)  *ref*  aOR 0.029 (0.07–1.28)  *ref*  aOR 1.25 (0.57–2.75)  *ref*  aOR 1.35 (0.67–2.76)  *ref*  aOR 1.28 (0.63–2.60)  *ref*  aOR 1.02 (0.37–2.86) | |
|  | Nepal | [16] | 1-35 months (Healthy controls) | 82.0% | Muslim religion  Latrine distance from household  Treated for illness 1-7 days before nasopharyngeal swab | aOR 2.93 (1.03-8.39)  aOR 2.41 (1.31-3.44)  aOR 0.37 (0.21-0.64) | |
| Western Pacific | Vietnam | [17] | 6 months-< 5 years | 33.6% (30.6–36.8) | Participant sex  Female  Male  Age (years)  < 1  > 1  Childcare attendance  No  Yes  Situation  Healthy children  Acute respiratory infection  Prior antibiotic use  No or unknown  Yes | *ref*  aOR 0.85 (0.63–1.14) *P* = 0.2725  *ref*  aOR 0.63 (0.43-0.93) *P* = 0.0203  *ref*  aOR 1.56 (1.16-2.17) *P* = 0.0073  *ref*  aOR 1.33 (0.93–1.90) *P* = 0.1137  *ref*  aOR 1.42 (0.98–2.05) *P* = 0.0621 | |
| **Lower-middle-income countries^c^ (n = 11)** | | | | | | | |
| Africa | Angola | [18] | 4–12 years | 35.3% | Age (each extra year as a continuous variable)  Female sex  BMI < 5^th^ percentile  Site of inclusion  Luanda (community)  Luanda (hospital)  Saurimo (community)  Chronic ear / auditory or respiratory tract symptoms  Ongoing infectious symptoms  Antibiotic treatment last month  Vaccinated according to schedule  > 5 children in the household  Smoker in the household  Access to grid electricity  *H. influenzae* colonization  *M. catarrhalis* colonization | aOR 0.95 (0.88–1.02)  aOR 1.06 (0.77–1.46)  aOR 1.10 (0.69–1.76)  *ref*  aOR 0.43 (0.17–1.07)  aOR 0.61 (0.30–1.24)  aOR 1.59 (0.77–3.29)  aOR 1.14 (0.48–2.72)  aOR 0.27 (0.09–0.83)  aOR 1.09 (0.73–1.61)  aOR 0.90 (0.65–1.24)  aOR 1.55 (0.96–2.51)  aOR 0.97 (0.52–1.83)  aOR 2.37 (1.51–3.72)  aOR 1.43 (0.92–2.20) | |
|  | Nigeria | [19] | <2.9 months | 47.8% (95% CI not reported) | Age group (years)  > 40  15–39  5–14  <5  Participant sex  Female  Male  Predominant cooking fuel  Kerosene  Other  Overcrowding index  < 3  > 4  Number of residents under five years old  0  > 1 | *ref*  aOR 1.1 (0.65–1.91) *P* = 0.70  aOR 2.9 (1.63-5.0) *P* < 0.001  aOR 7.4 (4.60-11.85) *P* < 0.001  *ref*  aOR 1.1 (0.81–1.48) *P* = 0.57  *ref*  aOR 0.5 (0.18–1.48) *P* = 0.10  *ref*  aOR 1.3 (0.97–1.78) *P* 0.07  *ref*  aOR 0.08 (0.61–1.11) *P* = 0.20 | |
|  |  |  | 6-9 months | 89.6% (95% CI not reported) |  |  |  |
|  |  |  | < 2 years | 74.4% (69.3-79.0) |  |  |  |
|  |  |  | < 18 years | 67.4% (63.3-71.0) |  |  |  |
|  |  |  | > 18 years | 26% (21.6-30.9) |  |  |  |
|  |  |  | All ages | 52.5% (49.4-55.7) |  |  |  |
| Americas | Bolivia | [20] | Children (ages not specified) mean 9.5 years (SD 3.5) | 34% | Age in Trinidad (years)  0–5  > 6  Age in Riberalta (years)  0–5  > 6  Participant sex  Female  Male  Residence  Trinidad  Riberalta | *ref*  aOR 0.7 (0.4–1.3)  *ref*  aOR 9.4 (1.2-76.9)  *ref*  aOR 1.6 (1.1-2.4)  *ref*  aOR 15.2 (1.6-146.3) | |
|  | Brazil | [21] | 10-19 years | 8.2% (6.6-10.0) | Age (years, as continuous variable)  Male sex  Passive exposure to cigarette smoke  Upper respiratory tract infection during recruitment  Episode of acute asthma | aOR 0.85 (0.77–0.94) *P* < 0.05  aOR 1.78 (1.11–2.85) *P* < 0.05  aOR 1.76 (1.10–2.79) *P* < 0.05  aOR 2.67 (1.67–4.28) *P* < 0.001  aOR (2.89 (1.18–7.08) *P* < 0.05 | |
| Eastern Mediterranean | West Bank and Gaza | [22] | < 5.5 years | 50% | Age  < 6 months  6–11 months  1 year  2 years  3–5.5 years  Parental pneumococcal carriage  No  Yes  Childcare attendance  No  Yes  Received antibiotics in past 6 months  No  Yes  Each extra household member | *ref*  aOR 0.36 (0.14-0.92) *P* = 0.003  aOR 0.81 (0.35–1.90) *P* = 0.63  aOR 0.53 (0.22–1.28) *P* = 0.16  aOR 0.47 (0.20–1.12) *P* = 0.09  *ref*  aOR 1.31 (0.59–2.94) *P* = 0.51  *ref*  aOR 3.72 (0.98-14.17) *P* = 0.05  *ref*  aOR 0.86 (0.55–1.33) *P* = 0.49  aOR 1.08 (1.01-1.14) *P* = 0.02 | |
| South East Asia | Indonesia | [23] | 6-60 months | 43% (32–50) | Being a child  Passive smoking  Toddlers at home | aOR 7.7 (4.5-13.0)  aOR 2.1 (1.4-3.4)  aOR 3.0 (1.9-4.7) | |
|  |  |  | 45-70 years | 11% (5–15) |  |  |  |
|  |  |  | Overall | 27% (20–32) |  |  |  |
|  |  | [24] | 2 months | 22% | Antibiotic exposure (current and/or within past 14 days)  Upper respiratory tract infection symptoms  Age (months) | aOR 0.44 (0.25–0.78) *P* = 0.004  aOR 1.21 (1.01–1.46) *P* = 0.041  aOR 1.22 (1.17–1.27) *P* < 0.001 | |
|  |  |  | 12 months | 68.4% |  |  |  |
| Western Pacific | China | [25] | 12-18 months | 16.6% | Season  Autumn  Spring  Age of child (months)  17–18  12  13–14  15–16  Residency  Non-local (relocated to Shanghai)  Local (from Shanghai)  Urbanicity (residential location)  Rural  Urban  Suburban  Father’s education  College or more  Primary school or less  Junior high  High school/vocational | *ref*  aOR 0.46 (0.27-0.78) *P* = 0.0044  *ref*  aOR 1.46 (0.69–3.09)  aOR 1.34 (0.64–2.84)  aOR 0.94 (0.42–2.09)  global *P* = 0.4703  *ref*  aOR 0.30 (0.13-0.69) *P* = 0.0048  *ref*  aOR 0.66 (0.34–1.25)  aOR 1.06 (0.46–2.44)  global *P* = 0.1773  *ref*  aOR 0.79 (0.21–2.90)  aOR 1.10 (0.52–2.34)  aOR 0.66 (0.29–1.52)  global *P* = 0.4037 | |
|  | Fiji | [26] | 3-13 months | 44.3% | Ethnicity  Fijian of Indian Descent  Indigenous iTaukei  Rural residence  Low birthweight  > 2 children aged < 5 years living in the household  Indoor cooking with wood  Hay fever symptoms  Acute respiratory tract infection symptoms  Breastfeeding | *ref*  aOR 2.81 (1.76-4.49) *P* < 0.001  aOR 1.64 (0.96–2.80) *P* = 0.073  aOR 0.61 (0.29–1.24) *P =* 0.172  aOR 1.49 (0.98–2.25) *P* = 0.061  aOR 1.43 (0.91–2.24) *P* = 0.123  aOR 1.15 (0.68–1.92) *P* = 0.605  aOR 1.93 (1.27-2.91) *P* = 0.002  aOR 0.74 (0.45–1.23) *P* = 0.244 | |
|  | Lao People’s Democratic Republic | [27] | 5–8 weeks | 15.7% | Ethnicity  Lao Loum  Other (Lao Thung, Hmong, and other)  Residence type  Urban  Rural  Upper respiratory tract infection symptoms  No  Yes  Two or more children < 5 years in the household  No  Yes  Family income  Above poverty line  Below poverty line  Mode of delivery  Vaginal  Caesarean | *ref*  aOR 1.65 (0.72–3.76) *P* = 0.233  *ref*  aOR 1.56 (0.85–2.84) *P* = 0.152  *ref*  aOR 1.17 (0.74–1.85) *P* = 0.493  *ref*  aOR 1.97 (1.39–2.79) *P* < 0.001  *ref*  aOR 1.64 (0.99–2.72) *P* = 0.055  *ref*  aOR 0.69 (0.44–1.09) *P* = 0.108 | |
|  |  |  | 12–23 months | 50.6% | Participant sex  Female  Male  Ethnicity  Lao Loum  Other (Lao Thung, Hmong, and other)  Residence type  Urban  Rural  Upper respiratory tract infection symptoms  No  Yes  Antibiotic use in the previous 2 weeks  No  Yes  Exposure to household cigarette smoke  No  Yes  Primary household fuel source  Non-biofuel (gas, kerosene, or electricity)  Biofuel (wood or charcoal)  Children < 5 years in the household  1  2 or more  Family income  Above poverty line  Below poverty line  Mode of delivery  Vaginal  Caesarean  PCV13 vaccination history  0 or 1 dose  2 or 3 doses | *ref*  aOR 0.79 (0.60–1.03) *P* = 0.080  *ref*  aOR 1.07 (0.54–2.11) *P* = 0.852  *ref*  aOR 1.84 (1.35–2.50) *P* < 0.001  *ref*  aOR 2.64 (1.97–3.53) *P* < 0.001  *ref*  aOR 0.91 (0.69–1.21) *P* = 0.523  *ref*  aOR 0.90 (0.68–1.20) *P* = 0.474  *ref*  aOR (1.24 (0.88–1.73) *P* = 0.214  *ref*  aOR 2.40 (1.80–3.20) *P* < 0.001  *ref*  aOR 0.96 (0.56–1.64) *P* 0.887  *ref*  aOR 0.80 (0.56–1.15) *P* = 0.227  *ref*  aOR 0.82 (0.62–1.09) *P* = 0.168 | |
|  | Mongolia | [28] | 5- 8 weeks | 28.4% (24.3–32.8) pre-PCV13 | Mother completed university  Yes  No  Number of children < 5 years in the household  One  Two or more  Housing type  Formal  Informal (ger) | *ref*  aOR 0.53 (0.34–0.83)  *ref*  aOR 1.64 (1.07–2.52)  *ref*  aOR 2.05 (1.30–3.24) | |
|  |  |  |  | 24.0% (20.3–28.0) post-PCV13 | Mother completed university  Yes  No  Number of children < 5 years in the household  One  Two or more  Exposure to household cigarette smoke  No  Yes  Housing type  Formal  Informal (ger) | *ref*  aOR 0.59 (0.37–0.92)  *ref*  aOR 2.07 (1.34–3.20)  *ref*  aOR 2.61 (1.22–5.60)  *ref*  aOR 2.07 (1.31–3.28) | |
|  |  |  | 12–23 months | 60.1% (55.6–64.4) pre-PCV13 | Mother completed university  Yes  No  Number of children < 5 years in the household  One  Two or more  Housing type  Formal  Informal (ger) | *ref*  aOR 0.66 (0.44–0.99)  *ref*  aOR 1.66 (1.10–2.50)  *ref*  aOR 1.74 (1.15–2.62) | |
|  |  |  |  | 55.9% (51.4–60.3) post-PCV13 | Mother completed university  Yes  No  Number of children < 5 years in the household  One  Two or more  Exposure to household cigarette smoke  No  Yes  Housing type  Formal  Informal (ger) | *ref*  aOR 0.60 (0.39–0.91)  *ref*  aOR 1.48 (1.01–2.18)  *ref*  aOR 2.09 (1.08–4.06)  *ref*  aOR 2.24 (1.42–3.54) | |
|  | Only risk factors significant on multivariable analysis were reported; other factors assessed for association with pneumococcal carriage included: participant sex, primary caregiver, crowding, minimum income, presence of a chimney, main source of cooking fuel, infant mode of delivery, breastfeeding, and receipt of PCV13 in patient or siblings | | | | | | |
| **Upper-middle-income countries^c^ (n = 18)** | | | | | | | |
| Africa | South Africa | [29] | 6-12 weeks to 2 years | 59.8% | *H. influenzae* co-colonization  *S. aureus* co-colonization  Age of child (not defined)  Influenza season  Peri-influenza season  Influenza season  Season of the year (not defined)  Children aged 3–6 years in the household  No  Yes  Use of coal/wood for fuel  No  Yes  Childcare attendance  No  Yes  Any smoker in the household  No  Yes  Interaction between *H. influenzae* and *S. aureus* | aOR 1.75 (1.32-2.32)  aOR 0.51 (0.39-0.67)  aOR 1.09 (1.06-1.10)  *ref*  aOR 1.37 (1.06-1.77)  aOR 0.88 (0.80-0.97)  *ref*  aOR 1.19 (0.92–1.56)  *ref*  aOR 1.58 (0.98–2.54)  *ref*  aOR 0.83 (0.59–1.16)  *ref*  0.94 (0.70–1.26)  aOR 2.28 (1.31–3.97) | |
|  |  |  | Mothers of infants aged 6–12 weeks to 2 years | 18.9% | *H. influenzae* co-colonization  *S. aureus* co-colonization  Age of mother (not defined)  Influenza season  Peri-influenza season  Influenza season  Season of the year (not defined)  Children aged 3–6 years in the household  No  Yes  Use of coal/wood for fuel  No  Yes  Childcare attendance  No  Yes  Any smoker in the household  No  Yes  Interaction between *H. influenzae* and *S. aureus* | aOR 1.48 (0.74–2.97)  aOR 0.77 (0.57–1.03)  aOR 0.94 (0.93–0.95)  *ref*  aOR 1.00 (0.75–1.33)  aOR 1.16 (1.01–1.32)  *ref*  aOR 0.72 (0.51–1.02)  *ref*  aOR 1.56 (0.91–2.67)  *ref*  aOR 1.20 (0.89–1.62)  *ref*  aOR 0.99 (0.71–1.39)  aOR 1.50 (0.54–4.17) | |
|  |  | [30] | 6 months | 66% | PM_10_ (particulate matter)  6 months  12 months  NO2 (nitrogen dioxide)  6 months  12 months  Benzene  6 months  12 months  Carbon monoxide  6 months  12 months  Smoking  6 months  12 months  Toluene  6 months  12 months | Adjusted for all clinical, demographic variables  aRR 0.96 (0.79–1.16)  aRR 1.02 (0.87–1.20)  aRR 1.32 (0.97–1.81)  aRR 1.07 (0.80–1.43)  aRR 1.01 (0.87–1.17)  aRR 1.01 (0.89–1.15)  aRR 1.03 (0.88–1.21)  aRR 1.05 (0.91–1.22)  aRR 1.14 (1.00–1.30)  aRR 1.0 (0.87–1.15)  aRR 0.95 (0.74–1.22)  aRR 1.1 (0.90–1.34) | Adjusted for all clinical-demographic variables and indoor air pollutants  aRR 0.96 (0.69–1.34)  aRR 0.98 (0.72–1.35)  aRR 1.93 (0.98–1.92)  -  aRR 0.88 (0.64–1.22)  aRR 1.17 (0.90–1.51)  aRR 1.28 (0.99–1.66)  aRR 1.13 (0.89–1.44)  aRR 1.12 (0.86–1.47)  aRR 0.93 (0.74–1.18)  aRR 1.23 (0.82–1.83)  aRR 1.00 (0.70–1.43) |
|  |  |  | 12 months | 68% |  |  |  |
|  |  |  | Adults | 13% |  |  |  |
|  |  | [31] | ~6, 10, 14 weeks  9–18 months  5 years | 43.7% | Age  6–14 weeks  9–18 months  5 years  Type of dwelling  Single room  Shack (informal dwelling)  *H. influenzae* co-colonization | *ref*  aOR 3.4 (1.9–5.9) *P* < 0.001  aOR 2.5 (1.1–5.5) *P* = 0.024  aOR 8.5 (1.3–52.3) *P* = 0.025  aOR 2.4 (1.2–4.5) *P* = 0.010  aOR 5.6 (0.6–2.5) *P* = < 0.001 | |
| Americas | Brazil | [32] | < 5 years | 66.6% (51.2–78.8) | Age (years)  > 17  < 2  2–4  5-17  Participant sex  Female  Male  Upper respiratory tract infection in the last month  Breastfeeding  School attendance | *ref*  aOR 14.1 (5.2-38.2)  aOR 8.0 (3.5-18.6)  aOR 1.5 (0.6–3.8)  *ref*  aOR 1.1 (0.7–1.8)  aOR 1.6 (0.9–2.8)  aOR 0.2 (0.05-1.0)  aOR 2.7 (1.2-6.0) | |
|  |  |  | 5–17 years | 45.3 (35–55.8) |  |  |  |
|  |  |  | > 17 years | 16.2% (10.1–24.2) |  |  |  |
|  |  |  | All ages | 36% (28–44) |  |  |  |
|  |  | [33] | 1-48 months | 55% | Ethnicity  White  Mixed  Upper respiratory tract infection in month pre-survey  No  Yes  Number of children < 2 years living in the same household  0  1  2  3  Each extra resident per number of rooms  Season  Feb–June  July–January | *ref*  aOR 0.52 (0.29-0.93)  *ref*  aOR 1.38 (1.00-1.89)  *ref*  aOR 1.44 (0.99–2.20)  aOR 1.38 (0.73–2.53)  aOR 2.00 (1.33–2.89)  aOR 1.77 (1.05-3.10)  *ref*  aOR 0.53 (0.67-0.78) | |
|  |  | [34] | < 6 years attending a public clinic for well-child or sick visit | 25.7% | Childcare attendance  Rhinitis  Increasing age (years)  Co-residents  Asthma or bronchitis  Residence in urban slum  Fever, coryza/sneezing, cough/expectoration, fatigue/breathlessness, hypoactivity, vomit, and/or diarrhoea | aOR 7.39 (3.25-16.81)  aOR 0.29 (0.11-0.79)  aOR 0.84 (0.65–1.09)  aOR 1.36 (0.93–1.99)  aOR 2.23 (0.99–5.02)  aOR 1.64 (0.89–3.05)  aOR 1.91 (0.99-3.72) | |
|  |  |  | < 6 years attending a private clinic for well-child or sick visit | 19.0% | Childcare attendance  Rhinitis  Increasing age (years)  Fever, coryza/sneezing, cough/expectoration, fatigue/breathlessness, hypoactivity, vomit, and/or diarrhoea | aOR 13.73 (4.28–44.07)  aOR 2.94 (1.94–16.00)  aOR 0.70 (0.47–1.07)  aOR 2.74 (1.12-7.04) | |
|  | Cuba | [35] | 2-18 months | 22% (19.1–23.3) | Intercept  Previous hospitalisations  Previous respiratory infections  Childcare attendance  Sharing bed with parents  Living with someone > 60 years of age  Sibling < 5 years of age | aOR 0.13 (0.08–0.19) *P* < 0.001  aOR 1.62 (1.05-2.48) *P* = 0.028  aOR 1.57 (1.03-2.45) *P* = 0.039  aOR 2.36 (1.26-4.33) *P* = 0.006  aOR 1.60 (0.96–2.62) *P* = 0.067  aOR 0.58 (0.38-0.87) *P* = 0.010  aOR 2.51 (1.82-3.48) *P* < 0.001 | |
|  | Venezuela | [36] | 3-65 months | 27% | Childcare attendance  No  Yes  Siblings  No  Yes  Family size  < 5 members  > 5 members  Antibiotic use in the week preceding the survey  No  Yes  Socio-economic level  Medium-high  Low | *ref*  aOR 1.63 (1.19-2.24) *P* = 0.002  *ref*  aOR 1.45 (1.3-2.03) *P* = 0.032  *ref*  aOR 1.17 (0.85–1.62) *P* = 0.332  *ref*  aOR 0.38 (0.19-0.76) *P* = 0.006  *ref*  aOR 1.90 (0.93–3.88) *P* = 0.077 | |
|  |  | [37] | 0-10 years and caregivers | 51% | Participant sex  Male  Female  Every increase of 1 year in age  Cooking method  Wood smoke  Gas  Wall status of the house  Without walls  With walls  Tobacco smoke exposure in household  No  Yes  Every extra person or child < 5 years in the household | *ref*  aOR 0.97 (0.81–1.2)  aOR 0.90 (0.89–0.92)  *ref*  aOR 0.79 (0.48–1.3)  *ref*  aOR 0.85 (0.59–1.2)  *ref*  aOR 1.2 (0.8–1.7)  aOR 1.0 (0.97–1.1) | |
|  |  |  | 0-4 years | 73% |  |  |  |
|  |  |  | 5-10 years | 55% |  |  |  |
|  |  |  | Caregivers | 15% |  |  |  |
|  |  |  | 0-4 years | 73% | Nutritional status  Every increase of 1 unit of height for age Z score  Every increase of 1 unit of weight for age Z score | aOR 0.76 (0.70–0.83)  aOR 1.1 (0.91–1.3) | |
| Eastern Mediterranean | Iran | [38] | 7–14 years | 12.03% | Region  Sex  Age  Household  Room  Sleeping  Smoking | aOR 1.05 (0.52–2.10) *P* = 0.883  aOR 1.33 (0.69–2.58) *P* = 0.385  aOR 1.07 (0.95–1.19) *P* = 0.239  aOR 1.06 (0.80–1.40) *P* = 0.652  aOR 0.96 (0.76–1.21) *P* = 0.759  aOR 0.93 (0.51–1.68) *P* = 0.810  aOR 1.85 (1.09–3.15) *P* = 0.022 | |
| Europe | Poland | [39] | 3-5 years | 37% in Autumn | During Autumn  Childcare attendance  Type of antibiotic | aOR 2.5 (1.3-4.9) *P* = 0.005  aOR 1.0 (95% CI not reported) *P* = 0.04 | |
|  |  |  |  | 33.1% in Winter | During Winter  Age  Childcare attendance  Respiratory tract infection  Number of antibiotic courses | aOR 0.75 (0.5–1.0) *P* = 0.08  aOR 2.1 (1.1-4.0) *P* = 0.02  aOR 1.9 (1.1-3.5) *P* = 0.03  aOR 0.5 (0.3-0.8) *P* = 0.002 | |
|  |  |  |  | 44.4% in Spring | During Spring  Age  Childcare attendance | aOR 0.6 (0.5-0.8) *P* = 0.003  aOR 2.6 (1.4-4.8) *P* = 0.001 | |
|  | Turkey | [40] | 0-2 years | 22.5% | Age (months)  < 2  > 2  Presence of child in the household who attends school  Rural settlement  Smoker present in the house  Antibiotic use in the previous 30 days | *ref*  aOR 2.98 (1.41-6.29) *P* = 0.004  aOR 1.72 (1.13-2.62) *P* = 0.01  aOR 1.15 (0.74–1.77) *P* = 0.52  aOR 1.18 (0.77–1.82) *P* = 0.43  aOR 1.07 (0.69–1.66) *P* = 0.75 | |
|  |  | [41] | 9 days-67 months | 37.2% | Increasing number of rooms in house  Increasing age (months) | aOR 0.574 (0.395-0.834) *P* = 0.004  aOR 0.978 (0.959-0.998) *P* = 0.028 | |
|  |  | [42] | 1 month-18 years | 21.9% | Age (months)  > 60  > 0–24  > 25–60  Child in the family who attends childcare  Respiratory infection in month preceding survey  Increase in the number of children in family by 1  Family income < ₺200  Family income ₺ 200–400  Family income ₺400–800  Family income ₺800-2000 | *ref*  aOR 3.68 (2.42-5.59) *P* < 0.001  aOR 2.05 (1.28-3.29) *P* = 0.003  aOR 1.93 (1.26-2.95) *P* = 0.002  aOR 1.43 (1.05-1.94) *P* = 0.021  aOR 1.38 (1.13-1.68) *P* < 0.001  aOR 3.96 (0.88–17.83) *P* = 0.072  aOR 1.62 (0.43–6.09) *P* = 0.4714  aOR 2.25 (0.6–8.37) *P* = 0.226  aOR 1.11 (0.28–4.27) *P* = 0.877 | |
|  |  | [43] | 0-6 years | 14% | Age (> 24 months)  Infections on the last three months  Childcare attendance  Antibiotic usage  Sibling | aOR 1.844 (0.608–5.593) *P* = 0.618  aOR 0.317(0.033–3.026) *P* =0.378  aOR 2.638 (0.765–9.098) *P* = 0.545  aOR 0.742 (0.075–7.323) *P* = 0.545  aOR 0.729 (0.201–0.2646) *P* =0.576 | |
| Western Pacific | Fiji | [44] | 5–8 weeks | Infants born by vaginal delivery: 27.3%;  Infants born by Caesarean section: 18,1%;  Overall: 26.1% | Infant mode of delivery  Vaginal  Caesarean  Survey year  Pre-PCV10  1 year post-PCV10  2 years post-PCV10  3 years post-PCV10  Residential location  Rural  Urban  Low family income  > 2 children aged < 5 years in the household | *ref*  aOR 1.57 (1.10–2.23) *P* = 0.01  *ref*  aOR 0.94 (0.69–1.27)  aOR 0.65 (0.46–0.91)  aOR 1.48 (1.11–1.96)  global *P* < 0 001  *ref*  aOR 1.09 (0.88–1.35) *P* = 0.44  aOR 1.32 (1.06–1.64) *P* = 0.01  aOR 1.99 (1.60–2.48) *P* < 0.001 | |
|  |  | [45] | 5–8 weeks  12–23 months  2–6 years  Caregivers | 32.4% | The number of physical contacts per 24 hours with:  Infants 5–8 weeks  Toddlers 12–23 months  Young children 2–6 years  Older children 7–14 years  Adults  Ethnicity  Fijian of Indian Descent  iTaukei  Residential location  Urban  Rural  Upper respiratory tract infection symptoms  Poverty  Participant group  Toddlers 12–23 months  Infants 5–8 weeks  Young children 2–6 years  Caregivers of pediatric participants  Participant sex  Male  Female  PCV10 vaccinated  Number of people living in the household | aOR 0.94 (0.73–1.22) *P* = 0.65  aOR 1.34 (1.07–1.68) *P* < 0.01  aOR 1.12 (0.98–1.28) *P* = 0.11  aOR 1.10 (0.98–1.23) *P* = 0.10  aOR 0.99 (0.92–1.08) *P* = 0.88  *ref*  aOR 5.16 (3.95–6.75) *P* < 0.01  *ref*  aOR 0.84 (0.67–1.06) *P* = 0.14  aOR 2.00 (1.58–2.55) *P* < 0.01  aOR 1.09 (0.87–1.38) *P* = 0.45  *ref*  aOR 0.70 (0.40–1.22)  aOR 0.98 (0.62–1.55)  aOR 0.08 (0.04–0.15)  global *P* < 0.01  *ref*  aOR 1.00 (0.80–1.25) *P* = 0.98  aOR 0.97 (0.61–1.55) *P* = 0.90  aOR 1.00 (0.95–1.05) *P* = 0.92 | |
|  |  | [46] | 5–8 weeks  12–23 months  2–6 years  Caregivers | 30.5% | PCV10 vaccination status  Not vaccinated  Vaccinated  Survey year  Pre-PCV10  1 year post-PCV10  2 years post-PCV10  3 years post-PCV10  Ethnicity  Fijian of Indian Descent  iTaukei  Participant group  Caregivers of pediatric participants  Infants 5–8 weeks  Toddlers 12–23 months  Children 2–6 years  Residential location  Rural  Urban  Participant sex  Male  Female  Number of children < 5 years living in the household  Less than two  Two or more  Family income level  Not low  Low  Symptoms of upper respiratory tract infection  Not present  Present  Household cigarette smoke  No exposure  Exposure | ref  aOR 0.82 (0.66–1.01) *P* = 0.065  *ref*  aOR 0.67(0.51–0.88)  aOR 0.49 (0.36–0.66)  aOR 0.62 (0.46–0.83)  global *P* < 0.001  *ref*  aOR 2.74 (2.17–3.45) *P* < 0.001  *ref*  aOR 4.15 (3.40–5.06)  aOR 8.88 (7.13–11.07)  aOR 8.48 (6.99–10.29)  global *P* <0.001  *ref*  aOR 1.45 (1.30–2.57) *P* <0.001  *ref*  aOR 1.06 (1.30–2.57) *P* < 0.001  *ref*  aOR 1.42 (1.27–1.59) *P* < 0.001  *ref*  aOR 1.44 (1.28–1.62) *P* < 0.011  *ref*  aOR 1.77 (1.57–2.01) *P* < 0.001  *ref*  aOR 0.97 (0.87–1.08) *P* = 0.555 | |
| **High-income countries^c^ (n = 36)** | | | | | | | |
| Americas | Canada | [47] | 10 months-5 years | 17.7% | Vaccination status  0–1 dose of any vaccine  2+ doses PCV7 & 0 doses PCV13  2+ doses of PCV7 & 1 dose PCV13  2+ doses of PCV13  Age  12 months  18 months  4-6 years  Siblings  0  > 1 < 2 years of age  > 1 > 2 years of age  Childcare attendance  0 hours/week  < 10 hours / week  > 10 hours / week  Otitis media in the previous year  0 episodes  1 episode  > 2 episodes  Antibiotics at the time of the survey  No  Yes  Antibiotics in 2 months preceding the survey  No  Yes | *ref*  aOR 0.81 (0.67-0.97) *P* < 0.05  aOR 0.48 (0.33-0.70) *P* < 0.005  aOR 0.74 (0.56-0.98) *P* < 0.005  *ref*  aOR 1.00 (0.85–1.18)  aOR 0.55 (0.44-0.69) *P* < 0.005  *ref*  aOR 1.95 (1.51-2.51) *P* < 0.005  aOR 2.24 (1.89-2.65) *P* < 0.005  *ref*  aOR 1.31 (0.97–1.76)  aOR 2.33 (1.99-2.74) *P* < 0.005  *ref*  aOR 1.35 (1.11-1.64) *P* < 0.005  aOR 1.45 (1.12-1.88) *P* < 0.005  *ref*  aOR 0.29 (0.15–0.56) *P* < 0.005  *ref*  aOR 0.68 (0.55-0.84) *P* < 0.005 | |
|  | United States of America | [48] | < 2-≥ 5 years | 24% in Community A  14% in Community B | Age (years)  < 2  2–4  > 5  Number of children < 8 years in the household  1  2  3  > 4  Siblings with pneumococcal carriage  Childcare exposure  Community  B  A | *ref*  aOR 2.1 (1.2-3.7) *P* = 0.007  aOR 2.3 (1.5-3.5) *P* < 0.001  *ref*  aOR 2.2 (1.1-4.5) *P* = 0.025  aOR 2.4 (1.1-5.0) *P* = 0.022  aOR 4.0 (1.9-8.3) *P* < 0.001  aOR 3.3 (2.2-5.0) *P* < 0.001  aOR 2.4 (1.6-5.0) *P* = 0.019  *ref*  aOR 1.7 (1.1-2.6) *P* = 0.010 | |
|  |  | [49] | < 6 years | 63.4% | Age (years)  > 2  < 2  Participant sex  Female  Male  Ever breastfed  No  Yes  Current antibiotic use  No  Yes  Antibiotic use in the past month  No  Yes  Smoker lives in the household  No  Yes  Would/coal burning stove in household  Sibling colonized with pneumococcus  No  Yes | *ref*  aOR 2.22 (1.42-3.49)  *ref*  aOR 1.52 (1.05-2.21)  *ref*  aOR 0.51 (0.27-0.94)  *ref*  aOR 0.53 (0.22 -1.27)  *ref*  aOR 1.94 (1.16-3.24)  *ref*  aOR 1.14 (0.74–1.77)  aOR 1.31 (0.95–1.83)  *ref*  aOR 4.31 (2.79-6.66) | |
|  |  | [50] | ≤ 5 years | 16.2% (12.4–20.8) | Childcare attendance for five or more days  Increasing age in years  Parental response of “yes” to “Does your child have any illness?” | aOR 2.56 (1.08-6.07)  OR 1.37 (1.07-1.75)  aOR 2.27 (0.962–5.34) | |
|  |  | [51] | < 7 years | 26% | Age (months)  < 5  5-< 24  24-< 36  > 36  Recent antibiotics  Childcare attendance  Respiratory tract infection/acute otitis media  Siblings at home  0  1  >1  Breastfed | *ref*  aOR 4.5 (2.2-9.5) *P*<0.001  aOR 3.0 (1.4-6.2) *P*<0.01  aOR 3.4 (1.6-7.3) *P*<0.01  aOR 0.69 (0.45–1.0) *P* = 0.08  aOR 2.3 (1.6-3.4) *P* <0.001  aOR 2.5 (1.7-3.6) *P* < 0.001  *ref*  aOR 1.5 (1.0-2.3) *P* = 0.04  aOR 2.5 (1.5-4.2) *P* = 0.0007  aOR 0.58 (0.38-0.89) *P* = 0.013 | |
|  |  | [52] | < 7 years | 26% | Siblings at home  0  1  > 1  Age (months)  < 5  5-< 24  24-< 36  > 36  Respiratory tract infection  Recent antibiotic use  Breastfed for > 2 months  Average household size ⩾ 2.9 persons  Median household income and childcare  Non-childcare, median income > $35,000  Non-childcare, median income < $35,000  Childcare, median income ⩾ $35,000  Childcare, median income < $35,000  Median household income replaceable by  Persons in poverty > 20%  > 40% adults with < high school education  Unemployment > 6%  Owner occupancy < 45%  Lack of unit plumbing > 0.5%  Children per square mile > 7000 | *ref*  aOR 1.8 (1.2-2.7)  aOR 2.5 (1.4-4.6)  *ref*  aOR 5.6 (2.3-13.6)  aOR 4.1 (1.7-10.3)  aOR 5.0 (2.1-11.9)  aOR 2.9 (1.9-4.2)  aOR 0.6 (0.4-0.9)  aOR 0.6 (0.3-0.9)  aOR 3.0 (1.7-5.5)  *ref*  aOR 2.7 (1.7-4.3)  aOR 2.9 (1.8–4.6)  aOR 2.7 (1.3-5.5)  aOR 2.5 (1.6–4.0)  aOR 2.8 (1.7–4.5)  aOR 2.4 (1.5–3.9)  aOR 2.1 (1.3–3.4)  aOR 1.8 (1.1–2.9)  aOR 2.3 (1.3–3.9) | |
|  |  | [53] | 3 months-<7 years in 2001 | 27% | Age (months)  3-< 6  6-< 24  24-< 36  > 36  Respiratory tract infection Childcare attendance  Young siblings  0  1  > 1  Recent antibiotic use | *ref*  aOR 4.0 (1.5-11.0)  aOR 2.0 (0.7–6.0) aOR 2.6 (1.0-7.3)  aOR 2.4 (1.7-3.6)  aOR 2.4 (1.6-3.7)  *ref*  aOR 1.5 (1.0-2.2)  aOR 2.8 (1.5-5.2)  aOR 0.6 (0.4-0.9) | |
|  |  |  | 3 months to <7 years in 2004 | 23% | Age (months)  3-< 6  6-< 24  24-< 36  > 36  Respiratory tract infection Childcare attendance  Young siblings  0  1  > 1  Recent antibiotic use | *ref*  aOR 1.2 (0.6–2.6)  aOR 1.3 (0.6–2.9)  aOR 0.6 (0.3–1.2)  aOR 1.3 (1.0–1.9)  aOR 2.0 (1.4–2.9)  *ref*  aOR 1.3 (0.9–1.9)  aOR 2.0 (0.9–4.3)  aOR 0.7 (0.5–1.0) | |
|  |  |  | 3 months-<7 years, 2007 | 30% | Age (months)  3-< 6  6-< 24  24-< 36  > 36  Respiratory tract infection Childcare attendance  Young siblings  0  1  > 1  Recent antibiotic use | *ref*  aOR 1.2 (0.7–2.2)  aOR 1.1 (0.6–2.3)  aOR 0.5 (0.3–1.0) aOR 1.4 (1.0–1.9)  aOR 2.2 (1.6–3.2)  *ref*  aOR 1.3 (0.9–1.8)  aOR 1.7 (1.0–2.8)  aOR 0.7 (0.5–0.9) | |
|  |  | [54] | 3-59 months | 38% in 2000  44% in 2001  35% in 2002 | Age (years  > 2  1-< 2  Childcare attendance  No  Yes  Household contact < 5 years  No  Yes  Antibiotics in previous 90 days  No  Yes | *ref*  aOR 1.4 (1.0-1.9)  *ref*  aOR 2.1 (1.6-2.7)  *ref*  aOR 1.6 (1.3-2.1)  *ref*  aOR 0.41 (0.32-0.54) | |
|  |  | [55] | 3-59 months | 38% in 2000  44% in 2001  35% in 2002  40% in 2003  41% in 2004 | Childcare attendance  > 1 household member aged < 59 months  Antimicrobial use in previous 90 days  Hospitalization in previous 90 days  Age (months)  24–59  12–23  Number doses of PCV7 / up to date for age | Increased odds (aOR not reported)  Increased odds (aOR not reported)  Reduced odds (aOR not reported)  Reduced odds (aOR not reported)  *ref*  Reduced odds (aOR not reported)  No association (aOR not reported) | |
|  |  | [56] | 6-23 months | Estimated from line graph  ~30.0% in 2001  ~25.0% in 2004  ~29.5% in 2007  ~31.0% in 2009  ~35.5% in 2011 | Respiratory tract infection at specimen collection  Vaccinated with PCV13  Young siblings (< 6 years) in household  0  1  > 2  Childcare attendance  None or < 4 hours / week  4–20 hours / week  > 20 hours / week  Recent antibiotic use  > 8 weeks or none recorded  < 2 weeks  2–4 weeks  4-< 8 weeks | aOR 1.85 (1.42-2.41) *P* < 0.001  aOR 1.07 (0.81–1.42) *P* = 0.63  *ref*  aOR 2.31 (1.75–3.04)  aOR 3.03 (2.00–4.60)  global *P* < 0.001  *ref*  aOR 2.00 (1.33–3.01)  aOR 3.05 (2.23–4.18)  global *P* < 0.001  *ref*  aOR 0.29 (0.17–0.52)  aOR 0.63 (0.41–0.97)  aOR 0.61 (0.39–0.94)  global *P* < 0.001 | |
|  |  |  | 2-7 years | Estimated from line graph  ~28.0% in 2001  ~22.5% in 2004  ~25.5% in 2007  ~26.0% in 2009  ~28.5% in 2011 | Respiratory tract infection at specimen collection  Vaccinated with PCV13  Young siblings (< 6 years) in household  0  1  > 2  Childcare attendance  None or < 4 hours / week  4–20 hours / week  > 20 hours / week  Recent antibiotic use  > 8 weeks or none recorded  < 2 weeks  2–4 weeks  4-< 8 weeks | aOR 1.18 (0.93–1.50) *P* = 0.17  aOR 1.23 (0.85–1.78) *P* = 0.28  *ref*  aOR 0.98 (0.77–1.26)  aOR 0.86 (0.55–1.34)  global *P* = 0.001  *ref*  aOR 1.43 (1.03–1.97)  aOR 1.87 (1.36–2.55)  global *P* < 0.001  *ref*  aOR 0.25 (0.13–0.50)  aOR 0.38 (0.22–0.65)  aOR 0.65 (0.41–1.03)  global *P* < 0.001 | |
|  |  | [57] | < 7 years from urban Boston | 22.3% | Age (months)  > 36  0-< 6  6-¸24  24–36  Ethnicity  White  Black non-Hispanic  Hispanic  Other non-Hispanic  Current Respiratory tract infection  Antibiotic use within two months  Childcare attendance  Number of siblings aged < 6 years vs > 1 year  > 1  0  1  College graduate or above  No  Yes  Household income  US $ >35,000  US $ < 35,000  Number of PCV7 doses  0  > 1 | *ref*  aOR 2.72 (1.17-6.35) *P* = 0.02  aOR 1.85 (1.01-3.39) *P* = 0.05  aOR 1.24 (0.60–2.58) *P* = 0.56  *ref*  aOR 0.85 (0.39–1.86) *P* = 0.69  aOR 0.79 (0.33–1.90) *P* = 0.59  aOR 1.67 (0.58–4.87) *P* = 0.34)  aOR 3.14 (1.98-4.99) *P* < 0.0001  aOR 0.92 (0.50–1.70) *P* = 0.78  aOR 2.86 (1.66-4.92) *P* = 0.00  *ref*  aOR 0.61 (0.25–1.47) *P* = 0.26  aOR 0.69 (0.27–1.75) *P* = 0.43  *ref*  aOR 1.39 (0.77–2.50) *P* = 0.27  *ref*  aOR 1.38 (0.79–2.42) *P* = 0.26  *ref*  aOR 1.15 (0.50–2.65) *P* = 0.74 | |
|  |  |  | < 7 years from outside Boston | 30.4% | Age (months)  > 36  0-< 6  6-24  24–36  Ethnicity  White  Black non-Hispanic  Hispanic  Other non-Hispanic  Current Respiratory tract infection  Antibiotic use within two months  Childcare attendance  Number of siblings aged < 6 years vs > 1 year  > 1  0  1  College graduate or above  No  Yes  Household income  US $ >35,000  US $ < 35,000  Number of PCV7 doses  0  > 1 | *ref*  aOR 2.81 (1.34-5.89) *P* = 0.01  aOR 2.73 (1.25-3.82) *P* < 0.0001  aOR 2.19 (1.25-3.82) *P* = 0.01  *ref*  aOR 0.53 (0.22–1.23) P = 0.14  aOR 1.35 (0.69–2.63) *P* = 0.38  aOR 0.53 (0.20–1.39) *P* = 0.20  aOR 2.17 (1.47-3.20) *P* < 0.0001  aOR 0.52 (0.36-0.74) *P* = 0.00  aOR 2.45 (1.63-3.67) *P* < 0.0001  *ref*  aOR 0.58 (0.33–1.01) *P* = 0.06  aOR 0.72 (0.41–1.27) *P* = 0.26  *ref*  aOR 1.10 (0.76–1.58) *P* = 0.62  *ref*  aOR 1.06 (0.62–1.79) *P* = 0.84  *ref*  aOR 0.95 (0.22–4.09) *P* = 0.95 | |
|  |  | [58] | < 10 years in rural Alaskan villages | 62.5% | Age  < 6 months  6–11 months  1 year  2–4 years  5–9 years  Village (region)  A (Bristol Bay)  B (Bristol Bay)  C (Norton Sound)  D (Norton Sound)  E (Yukon Delta)  F (Yukon Delta)  G (Yukon Delta)  H (Yukon Delta)  In-home running water  No  Yes  Number of persons per household  > 3 children < 10 years old  No  Yes  Increase of each extra person per room  Antibiotics in previous 90 days  No  Yes  Otitis media  Strep throat  Pneumonia | *ref*  aOR 1.63 (1.01-2.62)  aOR 1.79 (1.17-2.75)  aOR 2.03 (1.38-2.99)  aOR 1.30 (0.89–1.89)  global P < 0.001  *ref*  aOR 0.76 (0.48–1.20)  aOR 0.93 (0.66–1.31)  aOR 0.50 (0.33–0.76)  aOR 1.01 (0.73–1.42)  aOR 1.43 (0.96–2.15)  aOR 1.03 (0.72–1.47)  aOR 1.02 (0.71–1.46)  global *P* < 0.001  *ref*  aOR 1.35 (1.08-1.69) *P* = 0.01  *P* = 0.30 (aOR not reported)  *ref*  aOR 1.47 (1.22-1.77) *P* < 0.001  aOR 1.11 (1.03-1.20) *P* = 0.009  *ref*  aOR 0.57 (0.47-0.68) *P* < 0.001  aOR 0.79 (0.64-0.97) *P* = 0.02  *P* = 0.98 (aOR not reported)  *P* = 0.12 (aOR not reported) | |
|  |  |  | 10–17 years in rural Alaskan villages | 40.1% | Age  10- 14 years  15–17 years  Village (region)  A (Bristol Bay)  B (Bristol Bay)  C (Norton Sound)  D (Norton Sound)  E (Yukon Delta)  F (Yukon Delta)  G (Yukon Delta)  H (Yukon Delta)  In-home running water  No  Yes  Number of persons per household  > 3 children < 10 years old  No  Yes  > 3 adolescents 10-17 years old  No  Yes  Increase of each extra person per room  Antibiotics in previous 90 days  No  Yes | *ref*  aOR 0.43 (0.36–0.51) *P* < 0.001  *ref*  aOR 0.73 (0.44–1.17)  aOR 0.80 (0.56–1.15)  aOR 0.71 (0.49–1.03)  aOR 1.36 (0.99–1.88)  aOR 1.39 (1.01–1.91)  aOR 0.97 (0.70–1.36)  aOR 1.43 (1.03–1.99)  global *P* < 0.001  ref  *P* = 0.31 (aOR not reported)  aOR 1.06 (1.02–1.10) *P* = 0.001  *ref*  *P* = 0.63 (aOR not reported)  *ref*  *P* = 0.38 (aOR not reported)  *P* = 0.37 (aOR not reported)  *ref*  aOR 0.74 (0.61–0.91) *P* = 0.005 | |
|  |  |  | > 18 years in rural Alaskan villages | 14.3% | Age  18–49 years  > 50 years  Village (region)  A (Bristol Bay)  B (Bristol Bay)  C (Norton Sound)  D (Norton Sound)  E (Yukon Delta)  F (Yukon Delta)  G (Yukon Delta)  H (Yukon Delta)  In-home running water  No  Yes  Number of persons per household  > 3 children < 10 years old  No  Yes  > 3 adolescents 10-17 years old  No  Yes  Increase of each extra person per room  Antibiotics in previous 90 days  No  Yes  Bronchitis  No  Yes  Skin infection  No  Yes | *ref*  aOR 0.70 (0.58–0.83) *P* < 0.001  *ref*  aOR 0.96 (0.64–1.43)  aOR 0.85 (0.63–1.15)  aOR 0.66 (0.47–0.91)  aOR 1.17 (0.88–1.55)  aOR 1.00 (0.76–1.32)  aOR 0.86 (0.65–1.13)  aOR 1.14 (0.85–1.52)  global *P* = 0.005  ref  *P* = 0.44 (aOR not reported)  aOR 1.05 (1.02–1.07) *P* = 0.0006  *ref*  *P* = 0.90 (aOR not reported)  *ref*  *P* = 0.73 (aOR not reported)  *P* = 0.82 (aOR not reported)  *ref*  aOR 0.68 (0.55–0.84) *P* = 0.003  *ref*  aOR 1.41 (1.02–1.96) *P* = 0.04  *ref*  aOR 1.54 (1.03–2.31) *P* = 0.04 | |
|  |  | [59] | 3 months-< 7 years | 30% in 2006–2007  29% in 2008-2009 | Age (months)  36-< 84  3 to < 6  6 to < 24  24 to 36  Group childcare  Young siblings  0  1  > 1  Respiratory tract infection at specimen collection  Recent antibiotics | *ref*  aOR 1.90 (1.22-2.98) *P* = 0.005  aOR 2.13 (1.62-2.79) *P* < 0.001  aOR 1.45 (1.03-2.05) *P* = 0.033  aOR 2.21 (1.72-2.83) *P* < 0.001  *ref*  aOR 1.51 (1.22-1.88) *P* < 0.001  aOR 1.58 (1.10-2.26) *P* = 0.013  aOR 1.44 (1.16-1.77) *P* < 0.001  aOR 0.66 (0.52-0.82) *P* < 0.001 | |
| Eastern Mediterranean | Cyprus | [60] | 6 months-5 years | 35.3% | Participant sex  Male  Female  Age (years)  < 1  1–2  > 2  Nationality  Other  Both parents Cypriots  Breastfeeding  No  Yes  Exposure to smoking  No  Yes  Childcare attendance  No  Yes  Siblings  No  1  > 2  Vaccination  No  Incomplete  Full  Original of specimen  From a public center  From a private center | *ref*  aOR 0.91 (0.57–1.47) *P* = 0.71  *ref*  aOR 0.93 (0.50–1.74) *P* = 0.83  aOR 1.31 (0.63–2.72) *P* = 0.47  *ref*  aOR 2.31 (1.29–4.21) *P* < 0.001  *ref*  aOR 1.28 (0.73–2.27) *P* = 0.40  *ref*  aOR 1.43 (0.86–2.40) *P* = 0.17  *ref*  aOR 2.88 (1.62–5.20) *P* < 0.001  *ref*  aOR 2.72 (1.55–4.86) *P* < 0.001  aOR 2.78 (1.47–5.34) *P* < 0.001  *ref*  aOR 1.64 (0.79–3.38) *P* = 0.18  aOR 0.48 (0.25–0.92) *P* = 0.03  *ref*  aOR 1.05 (0.62–1.77) *P* = 0.85 | |
|  | Kingdom of Saudi Arabia | [61] | > 18 years, at the beginning of Hajj | 4.4% | Beginning of Hajj  Participant sex  Male  Female  Primary or lower formal education  > 50 years of age  Resident in Africa  Current tobacco cigarette smoking  Previous influenza or pneumococcal vaccination  Chronic respiratory disease | *ref*  aOR 0.81 (0.46–1.4)  aOR 1.2 (0.67–2.1)  aOR 0.66 (0.38–1.1)  aOR 1.0 (0.54–1.8)  aOR 1.7 (0.74–3.8)  aOR 0.44 (0.22–0.88)  aOR 1.5 (0.49–4.8) | |
|  |  |  | > 18 years, at the end of Hajj | 7.5% | End of Hajj  Participant sex  Male  Female  Primary or lower formal education  > 50 years of age  Resident in Africa  Current tobacco cigarette smoking  Previous influenza or pneumococcal vaccination  Chronic respiratory disease  Upper respiratory infection during Hajj  Used antibiotics during Hajj  Shared room with > 1 person with upper respiratory infection | *ref*  aOR 0.89 (0.51–1.6)  aOR 1.1 (0.68–1.9)  aOR 1.6 (0.92–2.7)  aOR 1.2 (0.68–2.1)  aOR 0.96 (0.52–1.8)  aOR 1.1 (0.59–1.9)  aOR 0.79 (0.35–1.8)  aOR 1.2 (0.69–2.0)  aOR 0.96 (0.52–1.8)  aOR 1.2 (0.69–2.2) | |
| Europe | France | [62] | 6-24 months | 50.8% | Acute otitis media Childcare attendance  Siblings  Carriage of *M. catarrhalis*  Age < 12 months  Carriage of *H. influenzae*  Recent antibiotic use  Carriage of *S. aureus*  Partial vaccination | aOR 2.98 (2.49-3.56) *P* < 0.0001  aOR 1.55 (1.32-1.82) *P* < 0.0001  aOR 1.63 (1.41-1.89) *P* < 0.0001  aOR 1.46 (1.27-1.69) *P* < 0.0001  aOR 1.12 (0.96–1.30) *P* = 0.16  aOR 0.81 (0.69-0.94) *P* = 0.006  aOR 0.75 (0.65-0.97) *P* = 0.002  aOR 0.62 (0.46-0.83) *P* = 0.001  aOR 1.25 (0.70–2.20) *P* = 0.45 | |
|  | France and the Kingdom of Saudi Arabia | [63] | 26–83 years | 7.4% | Participant sex  Female  Male  Chronic respiratory disease  *H. influenzae–M. catarrhalis* co-colonization | *ref*  aOR 4.14 (1.67–10.32)  aOR 3.22 (1.09–9.50)  aOR 6.22 (2.04–19.01) | |
|  | Greenland | [64] | 0-6 years | 50.6% | Age (years)  Participant sex  Female  Male  Ethnicity  Inuit  Mixed / other  Number of PCV13 doses  0  > 1  Region  West  East  Childcare attendance  No  Yes  Siblings in childcare  No  Yes  Persons/room  < 2  > 2  Tobacco exposure  No  Yes | aOR 0.8 (0.69-0.9) *P* < 0.01  *ref*  aOR 1.3 (0.8–2.0) P = 0.26  *ref*  aOR 0.7 (0.3–1.5) *P* = 0.32  *ref*  aOR 0.5 (0.3–1.1) *P* = 0.07  *ref*  aOR 0.9 (0.6–1.6) *P* = 0.94  *ref*  aOR 1.0 (0.5–1.9) *P* = 0.96  *ref*  *aOR* 1.6 (1.1-2.2) *P* = 0.04  *ref*  aOR 1.7 (1.0-2.9) *P* = 0.05  *ref*  aOR 1.3 (0.7–2.2) *P* = 0.39 | |
|  | Italy | [65] | 0-12 months | 22% (13–31) | Each month of life adjusted  Childcare attendance  Each sibling | aPR 1.02 (1.01-1.03) *P* = 0.006  aPR 1.51 (1.16-1.97) *P* = 0.001  aPR 1.55 (1.19-2.01) *P* = 0.002 | |
|  |  |  | 13-24 months | 48.6% (36.3–60.9) |  |  |  |
|  |  |  | 25-59 months | 60% (49.6–70.3) |  |  |  |
|  |  |  | 0-59 months | 50.1% (43–57.2) |  |  |  |
|  |  | [66] | 0-5 months | 7.14% | Age (months)  0–5  6–23  24–35  36–71  Childcare attendance  No  Yes  Young siblings  0  > 1  Smoking  No  Yes  Vaccination status  Unvaccinated  PCV7  PCV7/13 + PCV13  Partially vaccinated | *ref*  aOR 3.75 (2.19-6.43) *P* < 0.001  aOR 3.15 (2.36-4.22) *P* < 0.001  aOR 3.03 (1.00-9.21) *P* = 0.051  *ref*  aOR 2.31 (0.89–5.98) *P* = 0.084  *ref*  aOR 1.20 (0.91–1.57) *P* = 0.193  *ref*  aOR 0.70 (0.61–0.80) *P* < 0.001  *ref*  aOR 1.34 (0.97–1.87) *P* = 0.073  aOR 1.04 (0.74–1.43) *P* = 0.839  aOR 0.86 (0.72–1.03) *P* = 0.121 | |
|  |  |  | 6-23 months | 33.33% |  |  |  |
|  |  |  | 24-35 months | 33.06% |  |  |  |
|  |  |  | 36-71 months | 35.19% |  |  |  |
|  |  |  | < 5 years | 32.9% |  |  |  |
|  |  | [67] | < 6 years | 31.56% | Childcare centre attendance  Vaccination status  PCV7  PCV7/PCV13 + PCV13 | SEM analysis coefficients reported  0.54 (0.14-0.94) *P* = 0.009  0.09 (-0.027–0.45) *P* = 0.43  -0.02 (-0.40–0.35) *P* = 0.909 | |
|  |  | [68] | 3-12 months | 22% (19–26) | Age (months)  3-12  13-24  25-59  Participant sex  Female  Male  Caucasian  Siblings  None  1  > 2  Childcare attendance  Indirect smoke  Respiratory tract infection in the previous 90 days  Antibiotics in the last 90 days  PCV vaccination | *ref*  aPR 1.06 (0.85–1.32)  aPR 0.74 (0.58–0.94)  *ref*  aPR 0.91 (0.77–1.09)  aOR 1.17 (0.88–1.56)  *ref*  aPR 1.79 (1.46-2.19)  aPR 2.23 (1.72-2.88)  aPR 2.27 (1.85-2.80)  aPR 1.04 (0.87–1.24)  aPR 1.39 (1.15-1.68)  aPR 0.67 (0.52-0.86)  aPR 1.03 (0.82–1.13) | |
|  |  |  | 13-24 months | 31% (26–36) |  |  |  |
|  |  |  | 25-59 months | 32% (27–37) |  |  |  |
|  |  |  | 3–59 months | 27% (25–30) |  |  |  |
|  | Portugal | [69] | > 60 years | 2.2% (1.6–3.2) in 2010  2.6% (1.8–3.6) in 2011  1.6% (0.9–2.8) in 2012 | Area  Urban  Rural  Housing  Family home  Retirement home  Smoker  Chronic disease  Chronic obstructive pulmonary disease  Asthma  Respiratory infection in the previous year  Asthma/bronchitis  Cold/Flu  Mild symptoms of respiratory illness at sampling  Sputum  Cough  Shortness of breath | *ref*  aOR 2.0 (1.2-3.5)  *ref*  aOR 2.0 (1.1-3.6)  aOR 4.4 (1.9-9.2)  aOR 0.9 (0.5–1.7)  aOR 1.2 (0.5–2.5)  aOR 1.8 (0.8–4.0)  aOR 1.3 (0.8–2.1)  aOR 1.3 (0.6–2.5)  aOR1.3 (0.7–2.3)  aOR 1.8 (0.9–3.5) | |
|  | Spain | [70] | 1 year | 18.9% | Age (years)  1  4  Participant sex  Male  Female  Vaccination  Full (yes)  Full (no)  At least one dose (yes)  At least one dose (no)  Childcare / school attendance  No  Yes  Breastfeeding, number of months | *ref*  aOR 0.73 (0.52–1.03) *P* = 0.28  *ref*  aOR 0.98 (0.78–1.25) *P* = 0.78  *ref*  aOR 0.77 (0.58–1.02) *P* = 0.26  *ref*  aOR 0.85 (0.66–1.11) *P* = 0.27  *ref*  aOR 1.78 (1.24–1.53) *P* = 0.005  aOR 1.0 (0.99–1.00) *P* = 0.9 | |
|  |  |  | 4 years | 21% |  |  |  |
|  | The Netherlands | [71] | 1-19 years | 19% | Age and childcare attendance (reported together as a single aOR) | aOR 2.14 (1.44–3.18) | |
|  |  | [72] | 1.5 months | 8.3% | Increasing birth weight (grams)  Increasing gestational age (weeks)  Increasing parity  Participant sex  Male  Female  Maternal smoking  Education level mother  > 1 sibling | aOR 2.67 (1.22–5.86) *P* < 0.05  aOR 1.07 (0.86–1.34)  aOR 6.85 (1.96–23.92) *P* < 0.01  *ref*  aOR 1.27 (0.62–2.61)  aOR 0.24 (0.03–1.77)  aOR 1.35 (0.62–2.97)  aOR 4.33 (1.22–15.35) *P* < 0.05 | |
|  |  |  | 6 months | 31.3% | Increasing birth weight (grams)  Increasing gestational age (weeks)  Increasing parity  Participant sex  Male  Female  Maternal smoking  Education level mother  > 1 sibling  Childcare attendance  Duration of breastfeeding  As a continuous variable  Never  < 3 months  3–6 months  > 6 months  Exclusive breastfeeding  Formula-fed only  Partially breastfed  Exclusively breastfed for > 3 months  Pneumococcus at 1.5 months | aOR 1.30 (0.84–2.01)  aOR 1.05 (0.91–1.21)  aOR 1.87 (0.70–5.00)  *ref*  aOR 1.18 (0.78–5.00)  aOR 1.22 (0.65–2.30)  aOR 1.31 (0.82–2.08)  aOR 1.37 (0.50–3.73)  aOR 3.05 (1.88–4.95) *P* < 0.001  aOR 1.14 (0.93–1.40)  *ref*  aOR 1.42 (0.63–3.20)  aOR 1.82 (0.82–4.04)  aOR 1.46 (0.67–3.20)  *ref*  aOR 1.76 (0.84–3.68)  aOR 1.19 (0.54–2.65)  aOR 1.26 (0.50–3.14) | |
|  |  |  | 14 months | 44.5% | Increasing birth weight (grams)  Increasing gestational age (weeks)  Increasing parity  Participant sex  Male  Female  Maternal smoking  Education level mother  > 1 sibling  Childcare attendance  Duration of breastfeeding  As a continuous variable  Never  < 3 months  3–6 months  > 6 months  Exclusive breastfeeding  Formula-fed only  Partially breastfed  Exclusively breastfed for > 3 months  Pneumococcus at 1.5 months  Pneumococcus at 6 months | aOR 1.09 (0.70–1.71)  aOR 0.96 (0.84–1.10)  aOR 1.19 (0.43–3.26)  *ref*  aOR 1.04 (0.68–1.58)  aOR 0.79 (0.40–1.56)  aOR 0.95 (0.59–1.53)  aOR 0.81 (0.29–2.25)  aOR 2.78 (1.70–4.55) *P* < 0.001  aOR 1.11 (0.91–1.37)  *ref*  aOR 0.50 (0.21–1.19)  aOR 1.10 (0.47–2.55)  aOR 1.23 (0.55–2.74)  *ref*  aOR 0.91 (0.42–1.98)  aOR 0.95 (0.42–2.14)  aOR 0.55 (0.23–1.30)  aOR 2.43 (1.50–3.94) *P* < 0.001 | |
|  |  | [73] | 6-24 months | 66% (61-71) | Sex of participant  Female  Male  Age (months)  6  12  18  24  Exclusive breastfeeding for the first 6 weeks of life  Childcare attendance  Period of sampling  April-September  October-March  Presence of siblings in the household  Current upper respiratory tract infection symptoms  Prescription of antibiotics within two months  Regular use of a pacifier | *ref*  aOR 0.98 (0.74–1.28)  *ref*  aOR 2.38 (1.74-3.24)  aOR 2.31 (1.65-3.23)  aOR 1.72 (1.23-2.40)  aOR 0.90 (0.68–1.19)  aOR 3.26 (2.45-4.32)  *ref*  aOR 1.08 (0.86–1.35)  aOR 2.26 (1.69-3.02)  aOR 1.53 (1.14-2.06)  aOR 0.27 (0.18-0.40)  aOR 1.04 (0.79–1.36) | |
| Europe and Eastern Mediterranean | Israel and West Bank and Gaza^d^ | [74] | < 5 years | 28.5% in East Jerusalem  32.9% in West Bank and Gaza  28.5% overall | Childcare attendance  No  Yes  Number of household members  2–3  4–6  > 7  Age (months)  < 6  6–23  24–60  Region  Palestinian Authority  East Jerusalem  Year  2009  2010  2011  PCV7 effect (2010)  PCV7 effect (2011) | *ref*  aOR 1.24 (0.94–1.64) *P* = 0.132  *ref*  aOR 1.53 (1.24-1.89) *P* < 0.001  aOR 1.74 (1.35-2.24) *P* < 0.001  *ref*  aOR 2.40 (1.90-3.02) *P* < 0.001  aOR 1.29 (1.00-1.66) *P* = 0.052  *ref*  aOR 0.76 (0.57–1.03) *P* = 0.075  *ref*  aOR 0.98 (0.77–1.26) *P* = 0.895  aOR 0.80 (0.62–1.03) *P* 0.079  aOR 1.05 (0.69–1.60) *P* = 0.829  aOR 1.07 (0.70–1.65) *P* = 0.753 | |
| Western Pacific | Australia | [75] | Indigenous Australian children | | Age (years)  2–4  5–8  9–12  13–15  Runny nose in the previous week  No  Yes  *H. influenzae* detected  No  Yes  *M. catarrhalis* detected  No  Yes | *ref*  aOR 0.65 (0.28–1.54)  aOR 0.43 (0.17–1.08)  aOR 0.13 (0.03–0.56)  *ref*  aOR 1.80 (0.98–3.29)  *ref*  aOR 9.43 (4.71-18.5)  *ref*  aOR 2.67 (1.34-5.33) | |
|  |  |  | 2–15 years | 67.1% |  |  |  |
|  |  |  | 2–4 years | 82.4% |  |  |  |
|  |  |  | 5–8 years | 72.7% |  |  |  |
|  |  |  | 9–12 years | 52% |  |  |  |
|  |  |  | 13–15 years | 30% |  |  |  |
|  |  |  | Indigenous Australian adolescents and adults | | Age (years)  16–24  25–34  35–44  45–54  > 55  Participant sex  Female  Male  Chest infection in the previous month  No  Yes  Runny nose in the previous week  No  Yes  Frequency sitting at an outside fire  Never/monthly  Most days/weekly  Every day  *H. influenzae* detected  No  Yes  *M. catarrhalis* detected  No  Yes | *ref*  aOR 1.13 (0.52–2.49)  aOR 2.04 (0.92–4.54)  aOR 1.90 (0.83–4.34)  aOR 2.19 (0.88–5.50)  *ref*  aOR 2.21 (1.31–3.73)  *ref*  aOR 1.62 (0.98–2.67)  *ref*  aOR 1.66 (1.05-2.64)  *ref*  aOR 1.23 (0.74–2.05)  aOR 6.89 (1.87-25.4)  *ref*  aOR 6.74 (4.06-11.2)  *ref*  aOR 3.27 (1.97-5.45) | |
|  |  |  | > 16 years | 26.0% |  |  |  |
|  |  |  | 16–24 years | 17.3% |  |  |  |
|  |  |  | 25–34 years | 22.2% |  |  |  |
|  |  |  | 35–44 years | 30.8% |  |  |  |
|  |  |  | 45–54 years | 31% |  |  |  |
|  |  |  | > 55 years | 38% |  |  |  |
|  | Hong Kong | [76] | < 5 years community based Chinese children | 10.8% (8.3–13.2) | Ethnicity  Chinese (community-based)  Vietnamese (refugee camp based)  Age > 3 months  Smoking family member(s)  Smaller household area per person  > 2 siblings | *ref*  aOR 3.52 *P* < 0.0001  aOR 3.5 *P* < 0.0001  aOR 1.7 *P* = 0.0031  aOR 1.11 *P* = 0.005  aOR 2.8 *P* = 0.0045 | |
|  |  |  | < 5 years Vietnamese refugees in detention | 55.7% (50.1–61.3) |  |  |  |
|  |  | [77] | 2 months | 2.3% | Age (months)  2 months  12 months  18 months  Siblings < 6 years old  Respiratory symptoms in recent three days  Respiratory symptoms in recent month  Doctor visit in recent in month preceding survey  Respiratory symptoms in household members in recent one month | *ref*  aOR 2.88 (1.41-5.87) *P* = 0.004  aOR 2.19 (1.05-4.57) *P* = 0.04  aOR 3.90 (2.44-6.23) *P* < 0.001  aOR 2.13 (1.31-3.47) *P* = 0.002  aOR 1.71 (1.07-2.73) *P* = 0.03  aOR 1.17 (0.65–2.10) *p* = 0.60  aOR 1.00 (0.60–1.65) *P* = 0.98 | |
|  |  |  | 12 months | 7.9% |  |  |  |
|  |  |  | 18 months | 5.9% |  |  |  |
|  |  |  | Total | 5.5% |  |  |  |
|  | Japan | [78] | 0-36 months | 33.3% | Start of childcare attendance (months of age)  > 24  12–23  < 12  Older Siblings and their childcare attendance  No siblings  Siblings not attending childcare  Siblings attending childcare  Clinic visits  < 20  > 21  Breastfeeding at six months of age  Exclusively  Partial  Bottle milk | *ref*  aHR 1.549 (1.126-2.132) *P* = 0.007  aHR 2.124 (1.488-3.032) *P* <0.001  *ref*  aHR 1.735 (1.199-2.511) *P* = 0.003  aHR 3.463 (2.616-4.584) *P* < 0.001  *ref*  aHR 1.089 (0.817–1.451) *P* = 0.562  *ref*  aHR 0.808 (0.590–1.106) *P* *=* 0.183  aHR 0.623 (0.447-0.868) *P* = 0.005 | |
|  |  | [79] | 2 months-6 years | 22% | Age (years)  < 1  1–2  3-6  Childcare attendance  No  Yes  Presence of older siblings  No  Yes  Treatment with antibiotics during previous three months  No  Yes  Season  Winter (January–March)  Spring (April–June)  Summer (July–September  Autumn (October–December) | *ref*  aOR 0.89 (0.38–2.09) P = 0.783  aOR 0.74 (0.22–2.54) *P* = 0.633  *ref*  aOR 3.11 (1.00-09.65) *P* = 0.049  *ref*  aOR 2.64 (1.32-5.27) *P* = 0.006  *ref*  aOR 1.54 (0.73–3.25) *P* = 0.253  *ref*  aOR 1.56 (0.60–4.02) P = 0.358  aOR 0.36 (0.14-0.90) *P* = 0.028  aOR 0.82 (0.33–2.02) *P* = 0.664 | |
|  |  | [80] | 2–24 months | 25,2% | Age (months)  > 12 months  < 12 months  Participant sex  Female  Male  Childcare attendance  No  Yes  Siblings  No  Yes  Inoculation times  1-2  None  3-4  None | *ref*  1.60 (0.93–2.76) *P* = 0.09  *ref*  aOR 0.92 (0.63–1.33) *P* = 0.66  *ref*  aOR 6.21 (4.00–9.65) *P* < 0.001  *ref*  aOR 2.26 (1.53–3.35) *P* < 0.001  *ref*  aOR 2.19 (0.95–5.02) *P* = 0.07  *ref*  aOR 1.59 (0.65–3.86) *P* = 0.31 | |
|  | Taiwan (China) | [81] | 2-5 years | 13.6% | Age (months)  0-< 12  12-< 24  24-< 60  Siblings  0  1  2  > 2  Childcare attendance  History of acute otitis media  Upper respiratory tract infection within two weeks  Antibiotic usage within 2 weeks  Breastfeeding  0  1 month  2 months  > 2 months  Household exposure to smoking | *ref*  aOR 1.29 (1.06–1.57) *P* < 0.05  aOR 1.04 (0.85–1.27)  *ref*  aOR 1.62 (1.36–1.92) *P* < 0.005  aOR 2.27 (1.83–2.81) *P* < 0.005  aOR 2.68 (2.02–3.55) *P* < 0.005  aOR 2.77 (2.32–3.3) *P* < 0.005  aOR 1.43 (1.17–1.75) *P* < 0.005  aOR 1.74 (1.51–2.01) *P* < 0.005  aOR 1.18 (0.92–1.51)  *ref*  aOR 0.91 (0.71–1.16)  aOR 0.93 (0.75–1.15)  aOR 1.02 (0.87–1.19)  aOR 1.18 (1.03–1.35) *P* < 0.05 | |
|  |  | [82] | >2–6 months | 5.5% | Age > 2 years  History of being breastfed  Having at least one sibling  Childcare attendance  Influenza vaccination  PCV7 vaccination  History of otitis media  Upper respiratory tract infection in last 2 weeks  Antibiotic use in the last 2 weeks  Influenza virus infection  *S. aureus* colonization | aOR 0.96 (0.79–1.16) *P* = 0.664  aOR 0.96 (0.82–1.13) *P* = 0.608  aOR 1.81 (1.51-2.16) *P* = <0.001  aOR 2.99 (2.46-3.65) *P* < 0.001  aOR 1.07 (0.91–1.25) *P* = 0.404  aOR 0.75 (0.56–1.02 *P* = 0.068  aOR 1.38 (1.11-1.72) *P* = 0.004  aOR 1.72 (1.47-2.01) *P* < 0.001  aOR 1.11 (0.85–1.46) *P* = 0.438  aOR 2.82 (0.93–8.57) *P* = 0.068  aOR 0.48 (0.39-0.58) *P* < 0.001 | |
|  |  |  | >6–12 months | 12.8% |  |  |  |
|  |  |  | >12–18 months | 12.1% |  |  |  |
|  |  |  | >18–24 months | 11.5% |  |  |  |
|  |  |  | >2–5 years | 19% |  |  |  |
|  |  |  | 2 months–5 years | 14.1% |  |  |  |

Abbreviations: 95% CI–95% confidence interval; aHR–adjusted hazard ratio; aOR–adjusted odds ratio; aPR–adjusted prevalence ratio; aRR–adjusted rate ratio; BMI–body mass index; PCV–pneumococcal conjugate vaccine; PCV7–seven-valent pneumococcal conjugate vaccine; PCV10–ten-valent pneumococcal conjugate vaccine; PCV13–thirteen-valent pneumococcal conjugate vaccine; SD–standard deviation; SEM–structural equation modeling; WHO–World Health Organization. Footnotes: ^a^ As per countries listed under WHO regional offices[83]; **^b^** As available in individual studies; **^c^** World Bank Income status at the time the study was undertaken[84]; ^d^ This study was conducted in high-income Israel (WHO European region) and lower-middle-income West Bank and Gaza (WHO Eastern Mediterranean regions)[74, 83, 84]

# References

1. Assefa A, Gelaw B, Shiferaw Y, Tigabu Z. Nasopharyngeal carriage and antimicrobial susceptibility pattern of *Streptococcus pneumoniae* among pediatric outpatients at Gondar University Hospital, North West Ethiopia. PEDN. 2013;54(5):315-21. doi: <https://dx.doi.org/10.1016/j.pedneo.2013.03.017>. PubMed PMID: 23680262.

2. Gebre T, Tadesse M, Aragaw D, Feye D, Beyene HB, Seyoum D, et al. Nasopharyngeal carriage and antimicrobial susceptibility patterns of *Streptococcus pneumoniae* among children under five in Southwest Ethiopia. Children. 2017;4(4). doi: 10.3390/children4040027. PubMed PMID: 28422083.

3. Wada FW, Tufa EG, Berheto TM, Solomon FB. Nasopharyngeal carriage of Streptococcus pneumoniae and antimicrobial susceptibility pattern among school children in South Ethiopia: post-vaccination era. BMC research notes. 2019;12(1):306. doi: <https://dx.doi.org/10.1186/s13104-019-4330-0>.

4. Haile AA, Gidebo DD, Ali MM. Colonization rate of Streptococcus pneumoniae, its associated factors and antimicrobial susceptibility pattern among children attending kindergarten school in Hawassa, southern Ethiopia. BMC Res Notes. 2019;12(1):344. Epub 2019/06/19. doi: 10.1186/s13104-019-4376-z. PubMed PMID: 31208447; PubMed Central PMCID: PMCPMC6580519.

5. Abdullahi O, Nyiro J, Lewa P, Slack M, Scott JA. The descriptive epidemiology of *Streptococcus pneumoniae* and *Haemophilus influenzae* nasopharyngeal carriage in children and adults in Kilifi district, Kenya. Ped Infect Dis J. 2008;27(1):59-64. doi: <https://dx.doi.org/10.1097/INF.0b013e31814da70c>. PubMed PMID: 18162940.

6. Abdullahi O, Karani A, Tigoi CC, Mugo D, Kungu S, Wanjiru E, et al. The prevalence and risk factors for pneumococcal colonization of the nasopharynx among children in Kilifi District, Kenya. PLoS One. 2012;7(2):e30787. doi: <https://dx.doi.org/10.1371/journal.pone.0030787>. PubMed PMID: 22363489.

7. Ousmane S, Diallo BA, Ouedraogo R, Sanda AA, Soussou AM, Collard JM. Serotype distribution and antimicrobial sensitivity profile of *Streptococcus pneumoniae c*arried in healthy toddlers before PCV13 introduction in Niamey, Niger. PLoS One. 2017;12(1):e0169547. doi: 10.1371/journal.pone.0169547. PubMed PMID: 28103262.

8. Bojang A, Jafali J, Egere U, Hill P, Antonio M, Jeffries D. Seasonality of pneumococcal nasopharyngeal carriage in rural Gambia determined within the context of a cluster randomized pneumococcal vaccine trial. PLoS One. 2015;10(7):13. PubMed PMID: CN-01130937.

9. Usuf E, Badji H, Bojang A, Jarju S, Ikumapayi UN, Antonio M, et al. Pneumococcal carriage in rural Gambia prior to the introduction of pneumococcal conjugate vaccine: a population-based survey. Trop Med Int Health. 2015;20(7):871-9. doi: <https://dx.doi.org/10.1111/tmi.12505>. PubMed PMID: 25778937.

10. Usuf E, Bojang A, Camara B, Jagne I, Oluwalana C, Bottomley C, et al. Maternal pneumococcal nasopharyngeal carriage and risk factors for neonatal carriage after the introduction of pneumococcal conjugate vaccines in The Gambia. Clin Microbiol Infect. 2018;24(4):389-95. Epub 2017/07/27. doi: 10.1016/j.cmi.2017.07.018. PubMed PMID: 28743545.

11. Hill PC, Akisanya A, Sankareh K, Cheung YB, Saaka M, Lahai G, et al. Nasopharyngeal carriage of *Streptococcus pneumoniae* in Gambian villagers. Clin Infect Dis 2006;43(6):673-9. doi: <https://dx.doi.org/10.1086/506941>. PubMed PMID: 16912937.

12. le Polain de Waroux O, Flasche S, Kucharski AJ, Langendorf C, Ndazima D, Mwanga-Amumpaire J, et al. Identifying human encounters that shape the transmission of *Streptococcus pneumoniae* and other acute respiratory infections. Epidemics. 2018;25:72-9. Epub 2018/07/29. doi: 10.1016/j.epidem.2018.05.008. PubMed PMID: 30054196; PubMed Central PMCID: PMCPMC6227246.

13. Lindstrand A, Kalyango J, Alfven T, Darenberg J, Kadobera D, Bwanga F, et al. Pneumococcal carriage in children under five years in Uganda-will present pneumococcal conjugate vaccines be appropriate? PLoS One. 2016;11(11):e0166018. doi: 10.1371/journal.pone.0166018. PubMed PMID: 27829063.

14. Nackers F, Cohuet S, le Polain de Waroux O, Langendorf C, Nyehangane D, Ndazima D, et al. Carriage prevalence and serotype distribution of *Streptococcus pneumoniae* prior to 10-valent pneumococcal vaccine introduction: A population-based cross-sectional study in South Western Uganda, 2014. Vaccine. 2017;35(39):5271-7. Epub 2017/08/09. doi: 10.1016/j.vaccine.2017.07.081. PubMed PMID: 28784282; PubMed Central PMCID: PMCPMC6616034.

15. Coles CL, Kanungo R, Rahmathullah L, Thulasiraj RD, Katz J, Santosham M, et al. Pneumococcal nasopharyngeal colonization in young South Indian infants. Ped Infect Dis J. 2001;20(3):289-95. PubMed PMID: 11303832.

16. Coles CL, Sherchand JB, Khatry SK, Katz J, Leclerq SC, Mullany LC, et al. Nasopharyngeal carriage of *S. pneumoniae* among young children in rural Nepal. Trop Med Int Health. 2009;14(9):1025-33. Epub 2009/07/01. doi: 10.1111/j.1365-3156.2009.02331.x. PubMed PMID: 19563428; PubMed Central PMCID: PMCPMC2770711.

17. Nguyen HAT, Fujii H, Vu HTT, Parry CM, Dang AD, Ariyoshi K, et al. An alarmingly high nasal carriage rate of Streptococcus pneumoniae serotype 19F non-susceptible to multiple beta-lactam antimicrobials among Vietnamese children. BMC Infect Dis. 2019;19(1):241. Epub 2019/03/15. doi: 10.1186/s12879-019-3861-2. PubMed PMID: 30866853; PubMed Central PMCID: PMCPMC6416861.

18. Uddén F, Filipe M, Slotved HC, Yamba-Yamba L, Fuursted K, Pintar Kuatoko P, et al. Pneumococcal carriage among children aged 4 - 12 years in Angola 4 years after the introduction of a pneumococcal conjugate vaccine. Vaccine. 2020;38(50):7928-37. Epub 2020/11/05. doi: 10.1016/j.vaccine.2020.10.060. PubMed PMID: 33143954.

19. Adetifa IM, Antonio M, Okoromah CA, Ebruke C, Inem V, Nsekpong D, et al. Pre-vaccination nasopharyngeal pneumococcal carriage in a Nigerian population: epidemiology and population biology. PLoS One. 2012;7(1):e30548. doi: <https://dx.doi.org/10.1371/journal.pone.0030548>. PubMed PMID: 22291984.

20. Inverarity D, Diggle M, Ure R, Johnson P, Altstadt P, Mitchell T, et al. Molecular epidemiology and genetic diversity of pneumococcal carriage among children in Beni State, Bolivia. Trans R Soc Trop Med Hyg. 2011;105(8):445-51. doi: <https://dx.doi.org/10.1016/j.trstmh.2011.04.013>. PubMed PMID: 21714978.

21. Cardozo DM, Nascimento-Carvalho CM, Andrade AL, Silvany-Neto AM, Daltro CH, Brandao MA, et al. Prevalence and risk factors for nasopharyngeal carriage of Streptococcus pneumoniae among adolescents. Journal of Medical Microbiology. 2008;57(Pt 2):185-9. doi: <https://dx.doi.org/10.1099/jmm.0.47470-0>. PubMed PMID: 18201984.

22. Regev-Yochay G, Raz M, Dagan R, Porat N, Shainberg B, Pinco E, et al. Nasopharyngeal carriage of *Streptococcus pneumoniae* by adults and children in community and family settings. Clin Infect Dis 2004;38(5):632-9. doi: <https://dx.doi.org/10.1086/381547>. PubMed PMID: 14986245.

23. Farida H, Severin JA, Gasem MH, Keuter M, Wahyono H, van den Broek P, et al. Nasopharyngeal carriage of *Streptococcus pneumoniae* in pneumonia-prone age groups in Semarang, Java Island, Indonesia. PLoS One. 2014;9(1):e87431. doi: <https://dx.doi.org/10.1371/journal.pone.0087431>. PubMed PMID: 24498104.

24. Murad C, Dunne EM, Sudigdoadi S, Fadlyana E, Tarigan R, Pell CL, et al. Pneumococcal carriage, density, and co-colonization dynamics: A longitudinal study in Indonesian infants. International journal of infectious diseases : IJID : official publication of the International Society for Infectious Diseases. 2019;86:73-81. doi: <https://dx.doi.org/10.1016/j.ijid.2019.06.024>.

25. Hu J, Sun X, Huang Z, Wagner AL, Carlson B, Yang J, et al. *Streptococcus pneumoniae* and *Haemophilus influenzae* type b carriage in Chinese children aged 12-18 months in Shanghai, China: a cross-sectional study. BMC Infect Dis. 2016;16:149. doi: <https://dx.doi.org/10.1186/s12879-016-1485-3>. PubMed PMID: 27080523.

26. Russell FM, Carapetis JR, Ketaiwai S, Kunabuli V, Taoi M, Biribo S, et al. Pneumococcal nasopharyngeal carriage and patterns of penicillin resistance in young children in Fiji. Ann Trop Paediatr 2006;26(3):187-97. doi: <https://dx.doi.org/10.1179/146532806X120273>. PubMed PMID: 16925955.

27. Dunne EM, Choummanivong M, Neal EFG, Stanhope K, Nguyen CD, Xeuatvongsa A, et al. Factors associated with pneumococcal carriage and density in infants and young children in Laos PDR. PLoS One. 2019;14(10):e0224392. doi: <https://dx.doi.org/10.1371/journal.pone.0224392>.

28. von Mollendorf C, Dunne EM, La Vincente S, Ulziibayar M, Suuri B, Luvsantseren D, et al. Pneumococcal carriage in children in Ulaanbaatar, Mongolia before and one year after the introduction of the 13-valent pneumococcal conjugate vaccine. Vaccine. 2019;37(30):4068-75. doi: <https://dx.doi.org/10.1016/j.vaccine.2019.05.078>.

29. Shiri T, Nunes MC, Adrian PV, Van Niekerk N, Klugman KP, Madhi SA. Interrelationship of *Streptococcus pneumoniae*, *Haemophilus influenzae* and *Staphylococcus aureus* colonization within and between pneumococcal-vaccine naive mother-child dyads. BMC Infect Dis. 2013;13:483. doi: <https://dx.doi.org/10.1186/1471-2334-13-483>. PubMed PMID: 24134472.

30. Vanker A, Nduru PM, Barnett W, Dube FS, Sly PD, Gie RP, et al. Indoor air pollution and tobacco smoke exposure: impact on nasopharyngeal bacterial carriage in mothers and infants in an African birth cohort study. ERJ Open Res. 2019;5(1). Epub 2019/02/12. doi: 10.1183/23120541.00052-2018. PubMed PMID: 30740462; PubMed Central PMCID: PMCPMC6360211.

31. Skosana Z, Von Gottberg A, Olorunju S, Mohale T, Du Plessis M, Adams T, et al. Non-vaccine serotype pneumococcal carriage in healthy infants in South Africa following introduction of the 13-valent pneumococcal conjugate vaccine. S Afr Med J. 2021;111(2):143-8. Epub 2021/05/05. doi: 10.7196/SAMJ.2021.v111i2.14626. PubMed PMID: 33944725.

32. Reis JN, Palma T, Ribeiro GS, Pinheiro RM, Ribeiro CT, Cordeiro SM, et al. Transmission of Streptococcus pneumoniae in an urban slum community. Journal of Infection. 2008;57(3):204-13. doi: <https://dx.doi.org/10.1016/j.jinf.2008.06.017>. PubMed PMID: 18672297.

33. Menezes AP, Azevedo J, Leite MC, Campos LC, Cunha M, Carvalho Mda G, et al. Nasopharyngeal carriage of *Streptococcus pneumoniae* among children in an urban setting in Brazil prior to PCV10 introduction. Vaccine. 2016;34(6):791-7. doi: <https://dx.doi.org/10.1016/j.vaccine.2015.12.042>. PubMed PMID: 26742946.

34. Neves FPG, Cardoso NT, Snyder RE, Marlow MA, Cardoso CAA, Teixeira LM, et al. Pneumococcal carriage among children after four years of routine 10-valent pneumococcal conjugate vaccine use in Brazil: The emergence of multidrug resistant serotype 6C. Vaccine. 2017;35(21):2794-800. doi: 10.1016/j.vaccine.2017.04.019. PubMed PMID: 28431817.

35. Toledo ME, Casanova MF, Linares-Perez N, Garcia-Rivera D, Torano Peraza G, Barcos Pina I, et al. Prevalence of pneumococcal nasopharyngeal carriage among children 2-18 months of age: baseline study pre-introduction of pneumococcal vaccination in Cuba. Ped Infect Dis J. 2017;36(1):e22-e8. doi: 10.1097/inf.0000000000001341. PubMed PMID: 27649366.

36. Rivera-Olivero IA, del Nogal B, Sisco MC, Bogaert D, Hermans PW, de Waard JH. Carriage and invasive isolates of *Streptococcus pneumoniae* in Caracas, Venezuela: the relative invasiveness of serotypes and vaccine coverage. Eur J Clin Microbiol Infect Dis. 2011;30(12):1489-95. doi: <https://dx.doi.org/10.1007/s10096-011-1247-5>. PubMed PMID: 21499972.

37. Verhagen LM, Hermsen M, Rivera-Olivero IA, Sisco MC, de Jonge MI, Hermans PW, et al. Nasopharyngeal carriage of respiratory pathogens in Warao Amerindians: significant relationship with stunting. Trop Med Int Health. 2017;22(4):407-14. doi: 10.1111/tmi.12835. PubMed PMID: 28072501.

38. Karami M, Hosseini SM, Hashemi SH, Ghiasvand S, Zarei O, Safari N, et al. Prevalence of nasopharyngeal carriage of Streptococcus pneumoniae in children 7 to 14 years in 2016: A survey before pneumococcal conjugate vaccine introduction in Iran. Human vaccines & immunotherapeutics. 2019;15(9):2178-82. doi: <https://dx.doi.org/10.1080/21645515.2018.1539601>.

39. Korona-Glowniak I, Malm A. Characteristics of *Streptococcus pneumoniae* strains colonizing upper respiratory tract of healthy preschool children in Poland. ScientificWorldJournal. 2012.

40. Ozdemir B, Beyazova U, Camurdan AD, Sultan N, Ozkan S, Sahin F. Nasopharyngeal carriage of Streptococcus pneumoniae in healthy Turkish infants. Journal of Infection. 2008;56(5):332-9. doi: <https://dx.doi.org/10.1016/j.jinf.2008.02.010>. PubMed PMID: 18377994.

41. Uzuner A, Ilki A, Akman M, Gundogdu E, Erbolukbas R, Kokacya O, et al. Nasopharyngeal carriage of penicillin-resistant *Streptococcus pneumoniae* in healthy children. Turk J Pediatr 2007;49(4):370-8. Epub 2008/02/06. PubMed PMID: 18246737.

42. Ozdemir H, Ciftci E, Durmaz R, Guriz H, Aysev AD, Karbuz A, et al. Risk factors for nasopharyngeal carriage of *Streptococcus pneumoniae* in healthy Turkish children after the addition of heptavalent pneumococcal conjugate vaccine (PCV7) to the national vaccine schedule. Turk J Pediatr. 2013;55(6):575-83. Epub 2014/03/01. PubMed PMID: 24577974.

43. Arvas A, Cokugras H, Gur E, Gonullu N, Taner Z, Bahar Tokman H. Pneumococcal nasopharyngeal carriage in young healthy children after pneumococcal conjugate vaccine in Turkey. Balkan Med J. 2017. doi: 10.4274/balkanmedj.2016.1256. PubMed PMID: 28443585.

44. Neal EFG, Nguyen C, Ratu FT, Matanitobua S, Dunne EM, Reyburn R, et al. A comparison of pneumococcal nasopharyngeal carriage in very young Fijian infants born by vaginal or Cesarean delivery. JAMA Netw Open. 2019;2(10):e1913650. Epub 2019/10/19. doi: 10.1001/jamanetworkopen.2019.13650. PubMed PMID: 31626319; PubMed Central PMCID: PMCPMC6813584.

45. Neal EFG, Flasche S, Nguyen CD, Ratu FT, Dunne EM, Koyamaibole L, et al. Associations between ethnicity, social contact, and pneumococcal carriage three years post-PCV10 in Fiji. Vaccine. 2020;38(2):202-11. doi: <https://dx.doi.org/10.1016/j.vaccine.2019.10.030>.

46. Neal EFG, Nguyen CD, Ratu FT, Dunne EM, Kama M, Ortika BD, et al. Factors associated with pneumococcal carriage and density in children and adults in Fiji, using four cross-sectional surveys. PLoS One. 2020;15(4):e0231041. doi: <https://dx.doi.org/10.1371/journal.pone.0231041>.

47. Ricketson LJ, Wood ML, Vanderkooi OG, MacDonald JC, Martin IE, Demczuk WH, et al. Trends in asymptomatic nasopharyngeal colonization with *Streptococcus pneumoniae* after introduction of the 13-valent pneumococcal conjugate vaccine in Calgary, Canada. Ped Infect Dis J. 2014;33(7):724-30. doi: <https://dx.doi.org/10.1097/INF.0000000000000267>. PubMed PMID: 24463806.

48. Samore MH, Magill MK, Alder SC, Severina E, Morrison-De Boer L, Lyon JL, et al. High rates of multiple antibiotic resistance in *Streptococcus pneumoniae* from healthy children living in isolated rural communities: association with cephalosporin use and intrafamilial transmission. Pediatrics. 2001;108(4):856-65. PubMed PMID: 11581436.

49. Millar EV, O'Brien KL, Zell ER, Bronsdon MA, Reid R, Santosham M. Nasopharyngeal carriage of *Streptococcus pneumoniae* in Navajo and White Mountain Apache children before the introduction of pneumococcal conjugate vaccine. Ped Infect Dis J. 2009;28(8):711-6. Epub 2009/07/14. doi: 10.1097/INF.0b013e3181a06303. PubMed PMID: 19593248.

50. Cheng Immergluck L, Kanungo S, Schwartz A, McIntyre A, Schreckenberger PC, Diaz PS. Prevalence of *Streptococcus pneumoniae* and *Staphylococcus aureus* nasopharyngeal colonization in healthy children in the United States. Epidemiol Infect. 2004;132(2):159-66. PubMed PMID: 15061489.

51. Finkelstein JA, Huang SS, Daniel J, Rifas-Shiman SL, Kleinman K, Goldmann D, et al. Antibiotic-resistant *Streptococcus pneumoniae* in the heptavalent pneumococcal conjugate vaccine era: predictors of carriage in a multicommunity sample. Pediatrics. 2003;112(4):862-9. PubMed PMID: 14523178.

52. Huang SS, Finkelstein JA, Rifas-Shiman SL, Kleinman K, Platt R. Community-level predictors of pneumococcal carriage and resistance in young children. Am J Epidemiol. 2004;159(7):645-54. PubMed PMID: 15033642.

53. Huang SS, Hinrichsen VL, Stevenson AE, Rifas-Shiman SL, Kleinman K, Pelton SI, et al. Continued impact of pneumococcal conjugate vaccine on carriage in young children. Pediatrics. 2009;124(1):e1-11. doi: <https://dx.doi.org/10.1542/peds.2008-3099>. PubMed PMID: 19564254.

54. Moore MR, Hyde TB, Hennessy TW, Parks DJ, Reasonover AL, Harker-Jones M, et al. Impact of a conjugate vaccine on community-wide carriage of nonsusceptible Streptococcus pneumoniae in Alaska. Journal of Infectious Diseases. 2004;190(11):2031-8. doi: <https://dx.doi.org/10.1086/425422>. PubMed PMID: 15529269.

55. Park SY, Moore MR, Bruden DL, Hyde TB, Reasonover AL, Harker-Jones M, et al. Impact of conjugate vaccine on transmission of antimicrobial-resistant *Streptococcus pneumoniae* among Alaskan children. Ped Infect Dis J. 2008;27(4):335-40. doi: <https://dx.doi.org/10.1097/INF.0b013e318161434d>. PubMed PMID: 18316986.

56. Lee GM, Kleinman K, Pelton SI, Hanage W, Huang SS, Lakoma M, et al. Impact of 13-Valent Pneumococcal Conjugate Vaccination on *Streptococcus pneumoniae* Carriage in Young Children in Massachusetts. J Pediatric Infect Dis Soc. 2014;3(1):23-32. doi: 10.1093/jpids/pit057. PubMed PMID: 24567842.

57. Hsu KK, Rifas-Shiman SL, Shea KM, Kleinman KP, Lee GM, Lakoma M, et al. Do community-level predictors of pneumococcal carriage continue to play a role in the conjugate vaccine era? Epidemiol Infect. 2014;142(2):379-87. doi: <https://dx.doi.org/10.1017/S0950268813000794>. PubMed PMID: 23731707.

58. Reisman J, Rudolph K, Bruden D, Hurlburt D, Bruce MG, Hennessy T. Risk factors for pneumococcal colonization of the nasopharynx in Alaska native adults and children. J Pediatric Infect Dis Soc. 2014;3(2):104-11. doi: 10.1093/jpids/pit069. PubMed PMID: 26625363.

59. Wroe PC, Lee GM, Finkelstein JA, Pelton SI, Hanage WP, Lipsitch M, et al. Pneumococcal carriage and antibiotic resistance in young children before 13-valent conjugate vaccine. Ped Infect Dis J. 2012;31(3):249-54. doi: <https://dx.doi.org/10.1097/INF.0b013e31824214ac>. PubMed PMID: 22173142.

60. Koliou MG, Andreou K, Lamnisos D, Lavranos G, Iakovides P, Economou C, et al. Risk factors for carriage of *Streptococcus pneumoniae* in children. BMC Pediatr. 2018;18(1):144. Epub 2018/04/28. doi: 10.1186/s12887-018-1119-6. PubMed PMID: 29699525; PubMed Central PMCID: PMCPMC5921789.

61. Memish ZA, Assiri A, Almasri M, Alhakeem RF, Turkestani A, Al Rabeeah AA, et al. Impact of the Hajj on pneumococcal transmission. Clin Microbiol Infect. 2015;21(1):77.e11-8. doi: <https://dx.doi.org/10.1016/j.cmi.2014.07.005>. PubMed PMID: 25636939.

62. Cohen R, Levy C, Bonnet E, Thollot F, Boucherat M, Fritzell B, et al. Risk factors for serotype 19A carriage after introduction of 7-valent pneumococcal vaccination. BMC Infect Dis. 2011;11:95. doi: <https://dx.doi.org/10.1186/1471-2334-11-95>. PubMed PMID: 21501471.

63. Hoang VT, Dao TL, Ly TDA, Belhouchat K, Chaht KL, Gaudart J, et al. The dynamics and interactions of respiratory pathogen carriage among French pilgrims during the 2018 Hajj. Emerg Microbes Infect. 2019;8(1):1701-10. Epub 2019/11/22. doi: 10.1080/22221751.2019.1693247. PubMed PMID: 31749410; PubMed Central PMCID: PMCPMC6882464.

64. Navne JE, Borresen ML, Slotved HC, Andersson M, Melbye M, Ladefoged K, et al. Nasopharyngeal bacterial carriage in young children in Greenland: a population at high risk of respiratory infections. Epidemiol Infect. 2016;144(15):3226-36. doi: 10.1017/s0950268816001461. PubMed PMID: 27405603.

65. Ansaldi F, de Florentiis D, Canepa P, Zancolli M, Martini M, Orsi A, et al. Carriage of *Streptococcus pneumoniae* 7 years after implementation of vaccination program in a population with very high and long-lasting coverage, Italy. Vaccine. 2012;30(13):2288-94. doi: <https://dx.doi.org/10.1016/j.vaccine.2012.01.067>. PubMed PMID: 22306795.

66. Camilli R, Daprai L, Cavrini F, Lombardo D, D'Ambrosio F, Del Grosso M, et al. Pneumococcal carriage in young children one year after introduction of the 13-valent conjugate vaccine in Italy. PLoS One. 2013;8(10):e76309. doi: <https://dx.doi.org/10.1371/journal.pone.0076309>. PubMed PMID: 24124543.

67. Camilli R, Vescio MF, Giufre M, Daprai L, Garlaschi ML, Cerquetti M, et al. Carriage of *Haemophilus influenzae* is associated with pneumococcal vaccination in Italian children. Vaccine. 2015;33(36):4559-64. doi: <https://dx.doi.org/10.1016/j.vaccine.2015.07.009>. PubMed PMID: 26190092.

68. Zuccotti G, Mameli C, Daprai L, Garlaschi ML, Dilillo D, Bedogni G, et al. Serotype distribution and antimicrobial susceptibilities of nasopharyngeal isolates of *Streptococcus pneumoniae* from healthy children in the 13-valent pneumococcal conjugate vaccine era. Vaccine. 2014;32(5):527-34. doi: 10.1016/j.vaccine.2013.12.003. PubMed PMID: 24342249.

69. Almeida ST, Nunes S, Santos Paulo AC, Valadares I, Martins S, Breia F, et al. Low prevalence of pneumococcal carriage and high serotype and genotype diversity among adults over 60 years of age living in Portugal. PLoS One. 2014;9(3):e90974. doi: <https://dx.doi.org/10.1371/journal.pone.0090974>. PubMed PMID: 24604030.

70. Alfayate Miguélez S, Yague Guirao G, Menasalvas Ruíz AI, Sanchez-Solís M, Domenech Lucas M, González Camacho F, et al. Impact of Pneumococcal Vaccination in the Nasopharyngeal Carriage of Streptococcus pneumoniae in Healthy Children of the Murcia Region in Spain. Vaccines (Basel). 2020;9(1). Epub 2021/01/01. doi: 10.3390/vaccines9010014. PubMed PMID: 33379235; PubMed Central PMCID: PMCPMC7823743.

71. Bogaert D, van Belkum A, Sluijter M, Luijendijk A, de Groot R, Rumke HC, et al. Colonisation by *Streptococcus pneumoniae* and *Staphylococcus aureus* in healthy children. Lancet. 2004;363(9424):1871-2. doi: <https://dx.doi.org/10.1016/S0140-6736(04)16357-5>. PubMed PMID: 15183627.

72. Labout JA, Duijts L, Arends LR, Jaddoe VW, Hofman A, de Groot R, et al. Factors associated with pneumococcal carriage in healthy Dutch infants: the generation R study. Journal of Pediatrics. 2008;153(6):771-6. doi: <https://dx.doi.org/10.1016/j.jpeds.2008.05.061>. PubMed PMID: 18621390.

73. Gils E, Veenhoven R, Rodenburg G, Hak E, Sanders E. Effect of 7-valent pneumococcal conjugate vaccine on nasopharyngeal carriage with *Haemophilus influenzae* and *Moraxella catarrhalis* in a randomized controlled trial. Vaccine. 2011;29(44):7595-8. doi: 10.1016/j.vaccine.2011.08.049. PubMed PMID: CN-00806054.

74. Daana M, Rahav G, Hamdan A, Thalji A, Jaar F, Abdeen Z, et al. Measuring the effects of pneumococcal conjugate vaccine (PCV7) on *Streptococcus pneumoniae* carriage and antibiotic resistance: the Palestinian-Israeli Collaborative Research (PICR). Vaccine. 2015;33(8):1021-6. doi: 10.1016/j.vaccine.2015.01.003. PubMed PMID: 25593104.

75. Mackenzie GA, Leach AJ, Carapetis JR, Fisher J, Morris PS. Epidemiology of nasopharyngeal carriage of respiratory bacterial pathogens in children and adults: cross-sectional surveys in a population with high rates of pneumococcal disease. BMC Infect Dis. 2010;10:304. doi: <https://dx.doi.org/10.1186/1471-2334-10-304>. PubMed PMID: 20969800.

76. Sung RY, Ling JM, Fung SM, Oppenheimer SJ, Crook DW, Lau JT, et al. Carriage of *Haemophilus influenzae* and *Streptococcus pneumoniae* in healthy Chinese and Vietnamese children in Hong Kong. Acta Paediatr. 1995;84(11):1262-7. PubMed PMID: 8580623.

77. Chan KC, Subramanian R, Chong P, Nelson EA, Lam HS, Li AM, et al. Pneumococcal carriage in young children after introduction of PCV13 in Hong Kong. Vaccine. 2016;34(33):3867-74. doi: 10.1016/j.vaccine.2016.05.047. PubMed PMID: 27265449.

78. Otsuka T, Chang B, Shirai T, Iwaya A, Wada A, Yamanaka N, et al. Individual risk factors associated with nasopharyngeal colonization with *Streptococcus pneumoniae* and *Haemophilus influenzae*: a Japanese birth cohort study. Ped Infect Dis J. 2013;32(7):709-14. doi: <https://dx.doi.org/10.1097/INF.0b013e31828701ea>. PubMed PMID: 23411622.

79. Ueno M, Ishii Y, Tateda K, Anahara Y, Ebata A, Iida M, et al. Prevalence and risk factors of nasopharyngeal carriage of *Streptococcus pneumoniae* in healthy children in Japan. Jpn J Infect Dis. 2013;66(1):22-5. PubMed PMID: 23429080.

80. Chang B, Akeda H, Nakamura Y, Hamabata H, Ameku K, Toma T, et al. Impact of thirteen-valent pneumococcal conjugate vaccine on nasopharyngeal carriage in healthy children under 24 months in Okinawa, Japan. Journal of infection and chemotherapy : official journal of the Japan Society of Chemotherapy. 2020;26(5):465-70. doi: <https://dx.doi.org/10.1016/j.jiac.2019.12.009>.

81. Hsieh YC, Chiu CH, Chang KY, Huang YC, Chen CJ, Kuo CY, et al. The impact of the heptavalent pneumococcal conjugate vaccine on risk factors for *Streptococcus pneumoniae* carriage in children. Ped Infect Dis J. 2012;31(9):e163-8. doi: <https://dx.doi.org/10.1097/INF.0b013e31825cb9f9>. PubMed PMID: 22592521.

82. Kuo CY, Hwang KP, Hsieh YC, Cheng CH, Huang FL, Shen YH, et al. Nasopharyngeal carriage of *Streptococcus pneumoniae* in Taiwan before and after the introduction of a conjugate vaccine. Vaccine. 2011;29(32):5171-7. doi: <https://dx.doi.org/10.1016/j.vaccine.2011.05.034>. PubMed PMID: 21621578.

83. World Health Organization. WHO/Who we are/Regional offices 2020 [cited 2020 April 3]. Available from: <https://www.who.int/about/who-we-are/regional-offices>.

84. World Bank Country and Lending Groups [Internet]. World Bank. 2020 [cited 2020 Jan 26].
