# Supplementary material for: Factors associated with pneumococcal nasopharyngeal carriage: A systematic review
Source: PLOS Glob Public Health. 2022 Apr 11;2(4):e0000327. doi: 10.1371/journal.pgph.0000327 (PMC10021834; doi:10.1371/journal.pgph.0000327)
Supplement: Attachment — Submitted filename: Author response PLoS Glob Health.pdf [file pgph.0000327.s010.pdf]

03 March 2022

Dear Dr Homaira,

We thank the Editor and the reviewers for their thorough assessment of our manuscript and the detailed and insightful comments. A point-by-point response to editorial requirements, comments, and reviewer comments is below.

Journal requirements:

While revising your submission, please upload your figure files to the Preflight Analysis and Conversion Engine (PACE) digital diagnostic tool, https://pacev2.apexcovantage.com/. PACE helps ensure that figures meet PLOS requirements. To use PACE, you must first register as a user. Registration is free. Then, login and navigate to the UPLOAD tab, where you will find detailed instructions on how to use the tool. If you encounter any issues or have any questions when using PACE, please email PLOS at figures@plos.org. Please note that Supporting Information files do not need this step.

Authors' response

We thank the Editor for this and confirm that we have uploaded our figure files to the Preflight Analysis and Conversion Engine.

Reviewer #1: Manuscript Number: PGPH-D-21-00990

Thank you for inviting me to review this paper. A well written and comprehensive review. However, I suggest minor revision for this review. Find below my comments:
1. Line 48: Write out the full meaning of HIV

Authors' response

We thank Reviewer 1 for their time and consideration of our manuscript. We have amended the manuscript accordingly.

2. Line 62: Any reason why facility-based studies were not included?

Authors' response

To minimise the non-comparability of included studies, we restricted studies to those of community-based populations. Inclusion of hospital or facility-based studies may introduce bias. Firstly, pneumococcal carriage rates among community-based populations and those attending health care facilities (particularly to treat respiratory infections) have previously been found to differ (Coles CL et al. Trop Med Int Health 2009; Kotler L et al. Vaccine. 2021). Secondly, although there are approximately 100 distinct pneumococcal serotypes, a relatively small proportion of the serotypes is responsible for most invasive pneumococcal disease (Hausdorf WP et al. Clin Infect Dis. 2000). As such, there may be a bias towards more invasive vaccine-serotypes among hospitalised populations than community-based populations (Hausdorf WP et al. Clin Infect Dis. 2000). Compared with community-based populations, hospital/facility-based populations have higher rates of exposure to factors that are relevant to facility-based populations, but less so to community-based populations, such as length of stay and receipt of antibiotics (Fortinksky RH et al. J Appl Gerontol.2014; Grant CC et al. J Paediatr Child Health. 2012). Additionally, hospitalised populations have higher rates of exposure to risk factors found to be associated with pneumococcal carriage risk factors, including crowding, exposure to cigarette smoke, comorbidities, receipt of antibiotics, and co-colonisation with other pathogens (e.g., Haemophilus influenzae and influenza type A and B) (Fortinksky RH et al. J

Appl Gerontol.2014; Grant CC et al. J Paediatr Child Health. 2012; Grijalva CG et al. Clin Infect Dis. 2014; Morris DE et al. Front Microbiol. 2017; Shrestha S et al. Sci Transl Med. 2013; Siegel SJ et al. Cell Host Microbe.2014).

3. Line 73: Any reason for not including grey literatures?

Authors' response

We appreciate the inclusion of grey literature, but this may introduce bias. Studies in grey literature may be of lower methodological quality than peer-reviewed and published studies. Further, identified grey literature studies may be an unrepresentative sample of unpublished studies.

4. Line 88: Full meaning of WHO.

Authors' response

We have amended our manuscript accordingly.

5. Line 97: Give the basis for the use Kruskal-Wallis method for this analysis.

Authors' response

We have amended the Methods section to clarify that
"We used a Kruskal-Wallis test to compare overall pneumococcal nasopharyngeal carriage rates by income classification (low, lower-middle, upper-middle, and high), as this is a rank-based, non-parametric method for testing differences between two or more categorical, independent groups on a continuous (or ordinal) outcome (where study-level carriage rates were treated as continuous)."

6. Line 120: In the discussion section can you give likely reasons for having just only one RCT in this study.

Authors' response

RCTs are considered the gold standard for investigating causal relationships between exposures/interventions and outcomes. However, this review focuses on factors associated with pneumococcal carriage rather than interventions to reduce pneumococcal carriage. As such, RCTs are less relevant to our research question.

7. Line 147: Any statement on the poor-quality studies? Were they included in the final analysis? Reasons for inclusion or non-inclusion.

Authors' response

In our original submission, supplementary file S Table 4 (Quality assessment of included studies) included a statement on poor-quality studies. In our revised manuscript, we have moved the statement on poor-quality studies to the Results section under the heading Quality Assessment. It reads, "Underlying reasons for poor quality related to a lack of sufficient detail in study methods. A cross-sectional study conducted in Hong Kong was considered poor quality as the methods were unclear, including a lack of clarity around the inclusion criteria and insufficient descriptions of pneumococcal carriage detection and statistical methods(Sung RY et al. Acta Paediatr. 1995). In this study, it was unclear how multivariable logistic regression models were built, there was no discussion regarding variable selection, and the only indication that a multivariable model had been used was

in the abstract (Sung RY et al. Acta Paediatr. 1995). A cross-sectional study from Bolivia was considered poor quality, as it lacked a clear research question, the inclusion and exclusion criteria were unclear, no sample size calculation was included, and the description of statistical methods was insufficient (Inverarity D et al. Trans R Soc Trop Med Hyg. 2011). Studies were not excluded based on poor quality to ensure transparency and completeness of reporting from all studies identified as relevant to the review (Shea BJ et al. BMJ. 2017)." As a narrative summary has been presented rather than a meta-analysis, and the limitations have been outlined, no sensitivity analyses have been undertaken.

8. Discussion – Need to make specific public health recommendations based on the findings of this review.

Authors' response

In our revised Discussion, we suggest, we suggest "Identifying factors associated with pneumococcal carriage in certain settings may help inform other public health interventions that may be needed. Some risk factors are not modifiable, such as age, living with young children (however this is most likely due to increased viral transmission in this age group), and ethnicity. However, the risk of pneumococcal carriage, transmission, and disease may be reduced by public health programs and policies that target particular age groups (Berical AC et al Ann Am Thorac Soc.2016; Weinberger DM et al. Am J Epidemiol. 2018), to reduce transmission, such as , such as increased access to improved sanitation and hygiene (Mattos KJ et al. Env Eng Sci. 2021; Gudnason T et al. Scand J Infect Dis. 2014), or that are tailored to address socio-economic differences and social determinants of health which promote tranmission (Dunne EM et al. Pneumonia. 2016). Reducing environmental risk factors for pneumococcal carriage and viral transmission includes improving breastfeeding, reducing malnutrition, preventing overcrowding, enhancing respiratory etiquette, and reducing smoke exposure. Public health programs that promote birth spacing (which may reduce the number of young siblings living in the same household), breastfeeding, interventions to reduce poverty, and which ensure high coverage of infant vaccination, may reduce the risk of pneumococcal disease (Danino D et al. Clin Infect Dis. 2021; Rybak A et al. Pathogens. 2021; Kim DH et al. Int J Environ Res Pub Health. 2021). Many of these modifiable factors are included in the WHO integrated Global Action Plan for the Prevention and Control of Pneumonia and Diarrhoea (World Health Organisation. GAPPD. 2013). Having programs to address these factors would also help prevent other infectious diseases that are a common cause of child morbidity and mortality in LMICs.

Recognising the importance of viral respiratory pathogens with regard to pneumococcal disease, we have included the following in our revised manuscript: "Vaccines and other public health interventions that modify viral respiratory pathogen infection are also likely to be important in preventing pneumococcal disease. In our review, we found that having symptoms of an acute respiratory tract infection (which are mostly viral in origin) was a risk factor for pneumococcal carriage in most studies where it was measured. Co-colonisation with respiratory viruses is associated with increased pneumococcal density, which increases the risk of acute respiratory infections and in some settings severe pneumonia (Howard LM et al. Emerg Infect Dis, 2019; Carr OJJ et al. J Infect Dis. 2021). In Israel, France, and South Korea, pneumococcal disease and community-acquired pneumonia declined during the public health measures used to control SARS-CoV-2 transmission (Danino D et al. Clin Infect Dis. 2021; Rybak A et al. Pathogens. 2021; Kim DH et al. Int J Environ Res Pub Health. 2021). Many countries reported declines in respiratory syncytial virus (RSV) during this period (Gastaldi A et al. Children. 2021; Di Mattia G et al. Pediatr Pulmonol. 2021). In Israel, pneumonia admissions declined despite pneumococcal carriage and density remaining unchanged. However, the circulation of other co-colonizing viruses which are known to increase the virulence of pneumococci declined substantially during the lockdown periods (Danino D et al. Clin Infect Dis. 2021; Smith CM et al. Am J Respir Crit Care Med. 2014). Although not assessed in primary studies in this review, pneumococcal carriage is more frequent in young children during infection with RSV than with other viruses (Brealey JC et al. Respirology. 2018; Sender V et al. Front Cell Infect

Microbiol. 2021). Further, RSV stimulates substantial growth of pneumococci, and co-colonization with RSV is associated with increased pneumococcal density and severity of acute respiratory tract infections (Brealey JC et al. Respirology. 2018; Weinberger DM et al. PLoS Med. 2015; Morpeth SC et al. Sci Rep. 2018). Additionally, co-colonization with influenza and parainfluenza have been found to increase the probability of pneumococcal acquisition (Grijalva CG et al. Clin Infect Dis. 2014). This suggests that public health interventions that modify the transmission of viral respiratory pathogens are also very important in preventing pneumococcal disease, including vaccines against RSV and influenza, and other interventions that reduce viral pathogen transmission. Co-colonization with H. influenzae and M. catarrhalis were also found to be risk factors for pneumococcal carriage in this review."

9. Line 384: There is need to include the "References" heading.

Authors' response

We have amended our manuscript accordingly.

10. Figure 1 – This is blur. Include a clearer picture.

Authors' response

We have uploaded our figure files to the Preflight Analysis and Conversion Engine to ensure figures meet PLOS requirements.

Reviewer 2: This systematic review describes some factors associated with pneumococcal carriage in children by classification of the settings on the income of country. It also describes carriage rates in children across those country level income categories. The manuscript is well structured and clearly written. The methods are acceptable and clearly described in adequate detail. I have the following comments to make.

A systematic review to answer this question need to rely on what RFs are assessed in the primary studies and that will not obviously be similar across studies. This needs to be properly highlighted and needs to be addressed in the methods with mention of any attempt to select homogenous studies in this regard somehow (noting studies in LMIC are few).

Authors' response

We thank Reviewer 2 for their time and review of our manuscript. An important point has been raised regarding the comparability of risk factors across studies. Due to the observational nature of all but one of the included studies, variables collected for assessment with pneumococcal carriage were dictated by primary study focus, design, and context. Comparability of risk factors across studies was low, such that meta-analysis was not appropriate. In our revised manuscript, we have amended the Eligibility criteria of the Methods section to include

"As risk factors assessed in primary studies were unlikely to be similar across all pneumococcal carriage studies, we limited our review to studies of healthy, community-based populations in an attempt to select studies with as similar as possible exposures. For transparency and completeness, we also present the results for all factors assessed for association with pneumococcal carriage by each included primary study."

How representative are these studies for each income category setting in particular in the low income settings. Even within these countries there could be variation between regions and population groups.

Authors' response

This review included primary studies which were most frequently based in high-income countries. Low and lower-middle-income countries were less represented. We agree with Reviewer 2 that there is likely variation between regions and within population groups. It is also very likely that there is underrepresentation from low- and middle-income countries, limiting the representativeness of these studies for these income settings. In addition, most studies used convenience sampling, and therefore their study populations may not be representative of their general populations. In our revised manuscript, we have amended the Limitations sections to include: "Although articles from low- and lower-middle-income countries were included in this review, most primary studies were conducted in high-income countries. Low- and lower-middle-income countries were proportionally underrepresented, limiting the potential representativeness of studies for these income settings. Further, most studies used convenience sampling. For these reasons, the studies for which pneumococcal nasopharyngeal carriage rates were available may not be representative of regional, country, or within-country populations. Therefore, we caution against using the reported rates by income classification as population or sub-population rates."

I get the sense that authors somewhat downplay the impact of PCV use in changing the pneumococcal carriage levels. It is true that in some settings NVTs have replaced VTs in carriage with not a substantial decline overall. However, there is overwhelming evidence that in all settings there is large reductions in disease particularly that of severe end of the spectrum (IPD). The classification of these study settings needs to be considered in terms of PCV use, schedule and duration of program.

Authors' response

Pneumococcal conjugate vaccines should be adopted in all countries, as they are highly effective against preventing invasive and non-invasive vaccine-type pneumococcal disease. However, vaccine-serotype circulation has been sustained in some settings post-PCV introduction (Swarthout TD et al. Not Commun 2020; Britton KJ et al. Vaccine. 2021). Additionally, in some settings where PCV has led to a decline in vaccine serotypes, concomitant increases in non-vaccine serotypes have been observed (Rose MA et al. Front. Med. 2021). Although non-vaccine serotypes tend to have lower intrinsic virulence than vaccine serotypes, increases in non-vaccine serotype disease post-PCV introduction has also been observed (Hausdorf WP et al. Clin Infect Dis. 2000; Weinberger DM et al. Lancet 2011; Feikin DR et al. PLoS Med. 2013). PCV is administered to infants and contact with young children is a risk factor for pneumococcal carriage (Neal EFG et al. Vaccine 2020). Our previous research also found that contact with older, unvaccinated children was a risk factor for vaccine-serotype carriage (Neal EFG et al. Vaccine 2020). While it is well known that vaccine-serotype carriage is modifiable by PCV, they can continue to circulate post-PCV introduction, and serotype replacement can also occur. Therefore, the focus of this review was to identify potentially modifiable risk factors in addition to PCV vaccination, in particular the importance of co-infection with viruses and the need to prevent viral transmission.

In our revised manuscript, we have ensured clarity around this topic by reordering the Discussion to highlight the importance and value of PCV. Further, we have added to the Discussion that

"PCVs are the primary intervention to control pneumococcal disease (van Gils EG et al. Jama. 2009; Mulholland EK & Satzke C. Lancet 2012; Lee GM et al. J Pediatric Infect Dis Soc. 2014; van Hoek AJ et al. Vaccine. 2014; Desai AP et al. Pediatr Infect Dis. 2015; Lindstrand A et al. Vaccine. 2016). Acknowledging PCVs are the best method of pneumococcal disease prevention, introducing PCV into national immunisation programs and ensuring high uptake is important."

Stratifying results by PCV use, schedule, and duration of the PCV program is beyond this review's scope. However, we are undertaking a separate systematic review on that topic.

Authors in the discussion highlight the importance on non-vaccine interventions to reduce carriage rates. I think these are all secondary. Besides are there sufficient evidence to suggest a significant reduction in carriage that causes most disease as a result of these possible 'other' interventions (noting also that most risk factors identified are not modifiable). This needs to be addressed in the discussion.

Authors' response

We have reordered and revised the Discussion section of our manuscript for clarity, highlighting that PCVs are the primary intervention against pneumococcal disease prevention. Few studies investigate the impact of other public health interventions on pneumococcal carriage in community-based populations.

Nonetheless, evidence exists supporting other interventions to protect against pneumococcal disease, for which pneumococcal carriage is a prerequisite. Public health interventions introduced in response to COVID-19 have reduced pneumococcal disease and community-acquired pneumonia. In Israel, reductions in hospital visits for community-alveolar pneumonia and bacteremic pneumococcal pneumonia were not associated with reductions in carriage or density. Instead, they were due to the lack of circulation of respiratory viruses, which are known to increase the virulence of pneumococci, highlighting how important it is to reduce transmission of viral respiratory pathogens through vaccination and other public health measures (Danino D et al. Clin Infect Dis. 2021; Smith CM et al., Am J Respir Crit Care Med 2014).

Our manuscript has been revised to accordingly – please refer to the response to Reviewer 1, Point 8.

The reason for excluding studies assessing RFs associated with VT and non-VT type carriage is not clear.

Authors' response

For clarity, we have amended the Introduction to include the following background: "PCV is the most effective measure to prevent pneumococcal disease and is an intervention known to reduce carriage of vaccine serotypes (Hammit LL et al. Lancet. 2019; Roca A et al. PLoS Med. 2011; Dunne EM et al Lancet Glob Health. 2018). In some settings, such as Malawi and Papua New Guinea, vaccine-serotypes have continued to circulate post-PCV introduction (Swarthout TD et al. Nat Commun. 2020; Britton KJ et al. Vaccine. 2021). Additionally, replacement with non-vaccine serotypes in carriage, and to a lesser extent disease, has occurred in some settings post-PCV introduction (Weinberger DM et al. Lancet. 2011; Feikin DR et al. PLoS Med. 2013; Rose MA et al. Front Med. 2021). In addition to introducing PCV, other interventions can also reduce pneumococcal disease (Danino D et al. Clin Infect Dis. 2021; Rybak A et al. Pathogens. 2021; Kim DH et al. Int J Environ Res Pub Health. 2021).

This study aims to identify risk factors for overall pneumococcal carriage to determine if there are other potentially modifiable exposures, in addition to PCV vaccination."

Need some mention of carriage in First Nations/Indigenous population in high income countries some of whom might have living conditions somewhat similar to LMIC settings.

Authors' response

In our revised manuscript, we have amended the Discussion to include, "Indigenous ethnicity was identified as a risk factor for pneumococcal carriage (Neal EFG et al. Vaccine. 2020 and Neal EFG et al. PLoS One. 2020). Further, notably high carriage rates were identified in Indigenous Australian and Navajo, and White Mountain Apache children in the United States of America (Millar EV et al. Ped Infect Dis J. 2009 and Mackenzie GA et al. BMC Infect Dis. 2010). Social determinants of health likely affect differential pneumococcal carriage (and disease) burden within countries, however comprehensive analysis of factors predisposing towards differences in pneumococcal carriage between indigenous and non-indigenous populations living in the same settings remain largely unqualified and unquantified."

It is unknown if the First Nations/Indigenous populations in primary studies included in this review were living in conditions "somewhat similar to LMIC settings." Due to heterogeneity of participant characteristics and study contexts, it has not been possible to compare study populations by income classification.

There are some conclusions drawn regarding the differences in carriage rates across income settings. The key question addressed in the review is risk factors associated with carriage primarily at an individual level. Drawing conclusions on overall carriage rates would need to consider the prevalence of risk factors in respective population. This needs to be addressed.

Authors' response

This is an important point, but unfortunately, it was not possible to compare rates of risk factors in respective study populations, or indeed by income classification. In our revised manuscript, we note in the Discussion that "This systematic review has brought together diverse studies from around the globe. For example, diversity is evident in the quality, inclusion criteria, study duration, and methods of pneumococcal detection and risk factor assessment. A meta-regression to understand drivers of variation in carriage across studies would have been possible had we comparable population-level information on studies. However, few studies measured the same exposure variables or measured them with similar definitions, and population-level risk factors were not documented, preventing comparison of risk factor rates by income classifications and limiting conclusions that could be drawn regarding differences in overall carriage rates across populations."

We hope that the revised version is now suitable for publication, and we look forward to hearing from you in due course.

Yours sincerely,

Eleanor Neal, on behalf of all co-authors
